# Boron, Aluminum, and Gallium Fluorides as Catalysts for the Defluorofunctionalization of Electron-Deficient Arenes: The Role of NaBAr^F^_4_ Promoters

**DOI:** 10.1021/acs.inorgchem.4c05381

**Published:** 2025-03-21

**Authors:** Wenbang Yang, Andrew J. P. White, Mark R. Crimmin

**Affiliations:** Molecular Sciences Research Hub, Imperial College London, 82 Wood Lane, White City, London W12 0BZ, U.K.

## Abstract

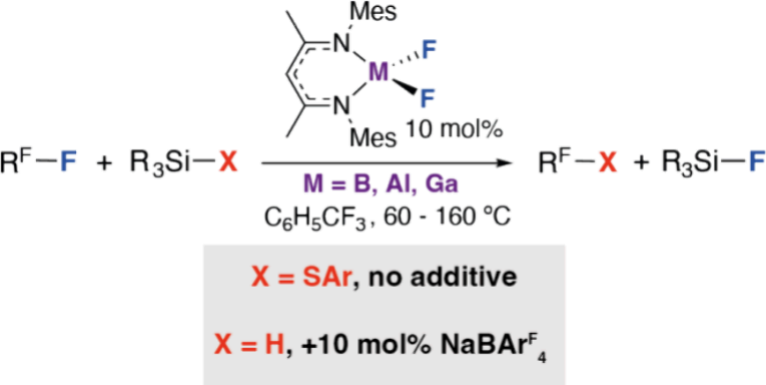

A series of boron, aluminum, and gallium difluoride complexes [{(ArNCMe)_2_CH}MF_2_] (M = B, Al, Ga) are reported as catalysts for the defluorofunctionalization of electron-deficient arenes. Thiodefluorination reactions between TMS–SPh and poly(fluorinated aromatics) proceed under forcing conditions. Evidence is presented for the fluoride entering the catalytic cycle through a metathesis reaction with TMS–SPh to form metal thiolate intermediates, e.g., [{(ArNCMe)_2_CH}MF(SPh)], which are then nucleophiles for addition to the aromatic substrate, likely through a concerted S_N_Ar mechanism. Attempts to expand the scope of reactivity to include the hydrodefluorination of electron-deficient arenes met with limited success. Activity could, however, be recovered through the addition of NaBAr^F^_4_ as a catalytic additive (Ar^F^ = 3,5-C_6_H_3_(CF_3_)_2_). NMR titrations suggest that NaBAr^F^_4_ is capable of coordinating with aluminum and gallium fluoride complexes, most likely through weak M–F---Na interactions (M = Al, Ga), and can play a role in lowering the barrier of metathesis between [{(ArNCMe)_2_CH}MF_2_] and Et_3_SiH to form the group 13 hydrido fluoride [{(ArNCMe)_2_CH}M(H)F], facilitating catalytic turnover. DFT calculations indicate that this weak interaction leads to a polarization of the M–F bond. The discovery of this additive effect has potentially broad implications in developing new reactivity and applications of thermodynamically stable metal fluorides.

## Abstract 2

A series of boron, aluminum, and gallium difluoride complexes [{(ArNCMe)2CH}MF2] (M = B, Al, Ga) are reported as catalysts for the defluorofunctionalization of electron-deficient arenes.

## Introduction

Compounds of the group 13 elements boron, aluminum, and gallium are routinely employed as Lewis acids in catalysis. The high fluoride-ion affinities of three-coordinate group 13 compounds underpin numerous applications that break and functionalize strong carbon–fluorine bonds.^1^ For example, BF_3_–OEt_2_ and AlCl_3_ have been reported as catalysts for the Friedel–Crafts alkylation of arenes with fluoroalkanes.^2^ B(C_6_F_5_)_3_ has been shown to catalyze the hydrodefluorination of fluoroalkanes using Et_3_SiH as a terminal reductant.^3^ Similar reactivity has been achieved using aluminum chloride fluoride (ACF), a heterogeneous catalyst proposed to contain highly Lewis acid active sites based on aluminum.^4−8^ Aluminum compounds are also competent reagents^9,10^ and catalysts^11,12^ for carbon–heteroatom and carbon–carbon from fluoroalkanes. In the past few years, Frustrated Lewis Pair (FLP) catalysts based on group 13 compounds have been applied to highly selective catalytic transformations that allow the controlled functionalization of a single carbon–fluorine bond of sp^3^ CF_3_ or sp^2^ CF_2_H groups.^13−18^

**Figure 1:**
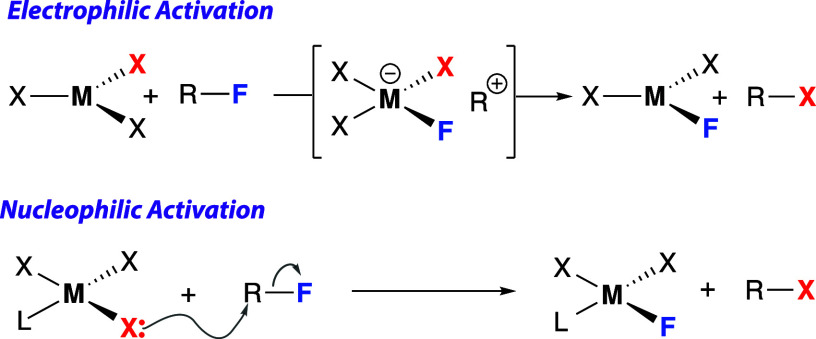
Selected reaction modes of group 13 compounds with carbon–fluorine bonds.

In contrast, the use of nucleophilic group 13 compounds as the catalyst for carbon–fluorine bond functionalization is less common. It might be expected that by increasing the coordination number of a group 13 compound from three- to four- coordinate groups, its Lewis acid behavior could be tempered and the nucleophilic reactivity of the coordinated ligands could be exposed (Figure 1).^19^ For example, we can consider the complementary reactivity profiles of i-Bu_2_AlH and LiAlH_4_, with the latter hydride complex often considered to be the more nucleophilic reagent. The switch in electronic behavior might be expected to expand the scope of accessible substrates for catalytic transformations, as Lewis acid catalysts operate primarily on sp^3^ C–F bonds, whereas nucleophilic species would be capable of addition to sp^2^ C–F bonds. This hypothesis is largely untested. Examples of nucleophilic catalytic behavior are, however, more widespread for the transition metals.^20−22^

In the past 10 years, our research group have developed several new reactions that led to the generation of well-defined four-coordinate aluminum fluoride complexes as products.^23−25^ More recently, we became interested in the use of these complexes as potential catalysts for the functionalization of carbon–fluorine bonds. Here, we show that molecular fluoride complexes of boron, aluminum, and gallium are competent catalysts for the thiodefluorination and hydrodefluorination of electron-deficient arenes and alkenes. We report two defined approaches to catalysis: (i) an additive-free reaction and (ii) a catalytic protocol that relies on the use of NaBAr^F^_4_ (Ar^F^ = 3,5-C_6_H_3_(CF_3_)_2_) as an additive. Experimental and computational data are provided to support the formation of nucleophilic four-coordinate complexes as key intermediates and metal fluoride species as on-cycle species. Furthermore, we suggest that the additive plays a unique role in polarizing and activating metal–fluorine intermediates through the formation of weak M–F----Na interactions. Our findings complement existing uses of compounds based on main group elements such as tetrabutylammonium difluorophenylsilicate (TBAT), tetrabutylammonium fluoride (TBAF), or CsF as initiators for carbon–fluorine bond functionalization.^26^

## Results and Discussion

### Additive-Free Thiodefluorination of Arenes

**Figure 2:**
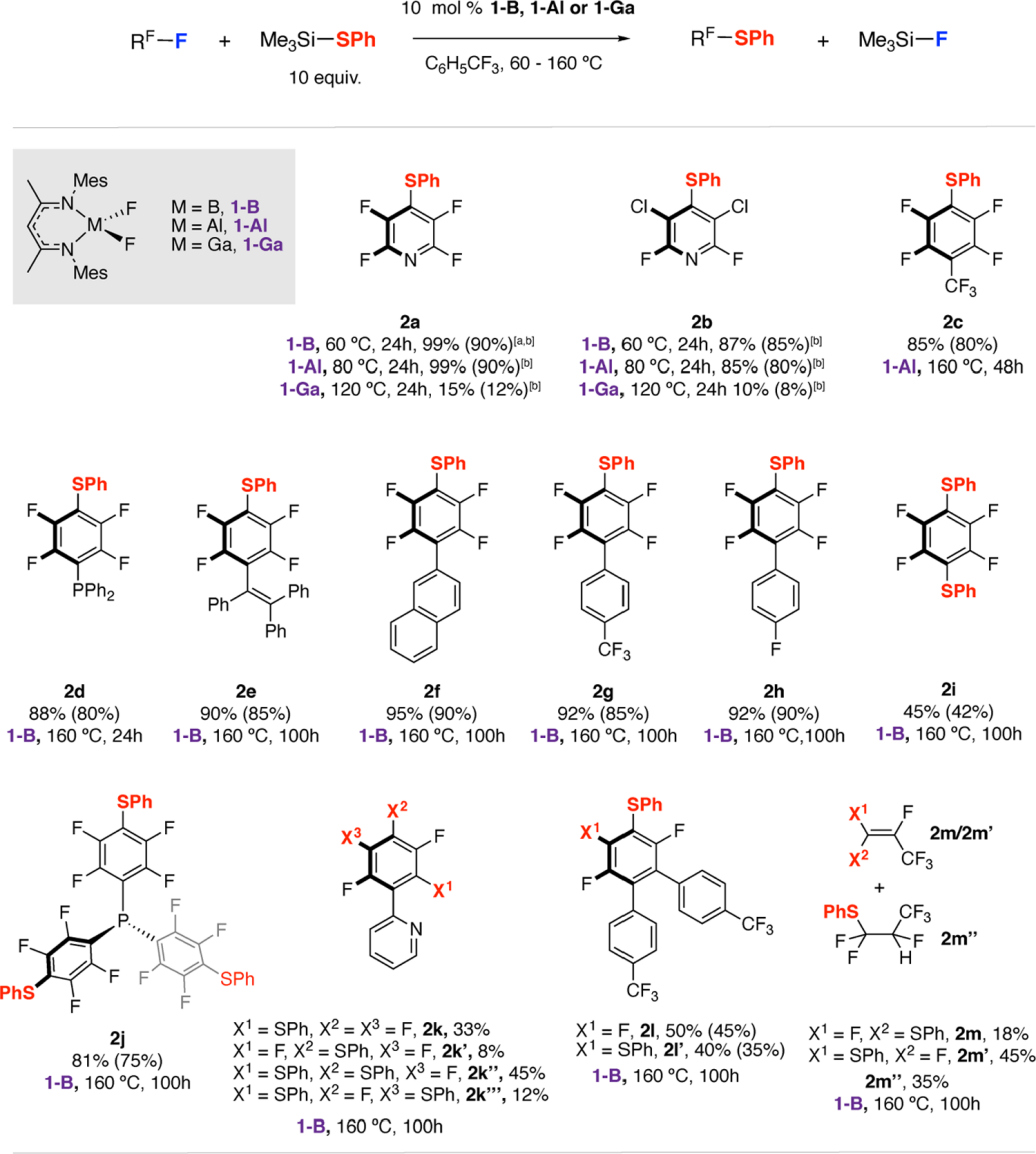
Scope of group 13 fluoride catalyzed defluorothioylation of fluorinated arenes and hexafluoropropene. ^a^NMR yields were measured by ^19^F NMR spectroscopy using 1,2-difluorobenzene as an internal standard. Isolated yields in parentheses. ^b^Reactions run using 3 equiv of Me_3_Si–SPh.

**Figure 3:**
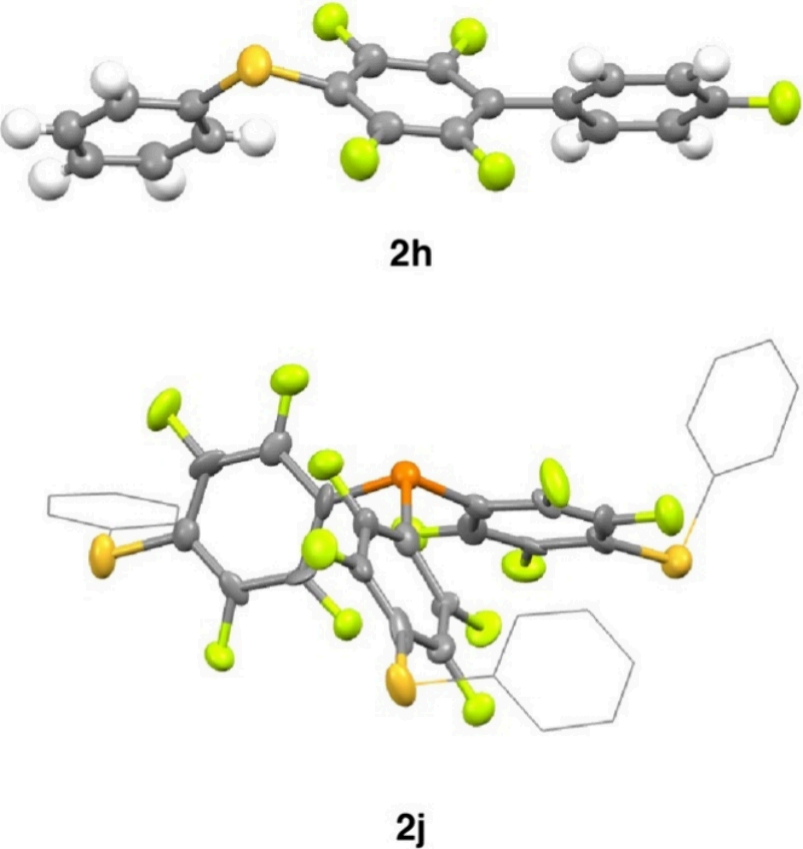
Crystal structures of **2h** and **2j**. 50% probability ellipsoids, and selected hydrogen atoms are omitted for clarity.

A series of four-coordinate group 13 difluoride complexes **1-B**, **1-Al**, and **1-Ga** supported by a sterically demanding β-diketiminate ligand were prepared. Initial experiments were conducted to screen a series of conditions for the reaction of pentafluoropyridine with group 13 catalysts using silanes as the terminal reductant. **1-B**, **1-Al**, and **1-Ga** were found to be active catalysts for the thiodefluorination of electron-deficient arenes with silicon-based reagents at 10–20 mol % loading, between 60 and 160 °C, using α,α,α-trifluorotoluene as a solvent (Figure 2). Using Me_3_Si–SPh as a terminal reagent, we could selectively convert pentafluoropyridine into **2a**. A control reaction showed a maximum of 10% conversion for this transformation after 24 h at 100 °C, when carried out in the absence of a catalyst. α,α,α-Trifluorotoluene was used as a solvent due to its high boiling point and its modest dielectric constant. Focusing on thiodefluorination, a scope of electron-deficient fluorinated aromatics was investigated. A series of substituted perfluoroarenes could be selectively converted to monosubstituted products **2b**–**2h**. Under more forcing conditions, higher levels of substitution of pentafluoropyridine and perfluorotoluene could be achieved (see the Supporting Information for details). Hexafluorobenzene reacted selectively, giving **2i** the product of 1,4-disubstitution. Similarly, P(C_6_F_5_)_3_ underwent a selective trisubstitution under catalytic conditions to yield **2j**, which could be crystallographically characterized (Figure 3). **2j** holds promise as a novel ligand for applications in catalysis. 2-(Perfluorophenyl)pyridine underwent nonselective thioylation to form isomers of **2k**, primarily at the 2- and 4-position of the perfluorophenyl ring. This finding suggests that the pyridyl-directing group has little control on the reaction, arguing against the coordination of the substrate to the catalyst expected during Lewis acid catalysis. An electron-deficient polyfluorinated terphenyl underwent nonselective single and double substitution to form **2l** and **2l**′****. Hexafluoropropene is also reactive under catalytic conditions, providing a mixture of stereoisomers **2m:2m′** from thioylation of the terminal sp^2^C–F bonds along with a side product **2m″** derived from protonation, likely from adventitious water.

For comparison, sodium thiolate anions are competent nucleophiles in S_N_Ar reactions with a range of fluoroarenes in polar solvents (e.g., ethylene glycol, pyridine, THF, DMF) under mild conditions.^27−30^ Similar reactions are reported to occur between fluoroalkenes or fluoroarenes and aryl thiols in the presence of K_2_CO_3_ as a base.^31,32^ Group 13 thiolate nucleophiles have also been reported for the defluorofunctionalization of alkyl fluorides.^9^ Catalytic approaches to thiodefluorination are less common but include fluoride metathesis reactions between thioesters and fluoroarenes catalyzed by either [RhH(PPh_3_)_4_] or 4-dimethylaminopyridine (DMAP).^33−35^

Qualitatively, for the synthesis of **2a** and **2b**, it was found that the catalysts were more active across the series **1-B** > **1-Al** > **1-Ga**. The heavier group 13 catalysts require more forcing conditions and show more limited scope than the lightest boron analogue. Reaction trends are consistent with a nucleophilic mechanism with regioselectivity determined by the most electrophilic position of the aromatic ring. In contrast to known Lewis acid catalysts based on group 13, these reactions are selective for sp^2^C–F over sp^3^C–F bonds with CF_3_ groups being tolerated under catalytic conditions. Less electron-deficient arenes, i.e., those with lower fluorine content, do not react under optimized conditions.

**Scheme 1:**
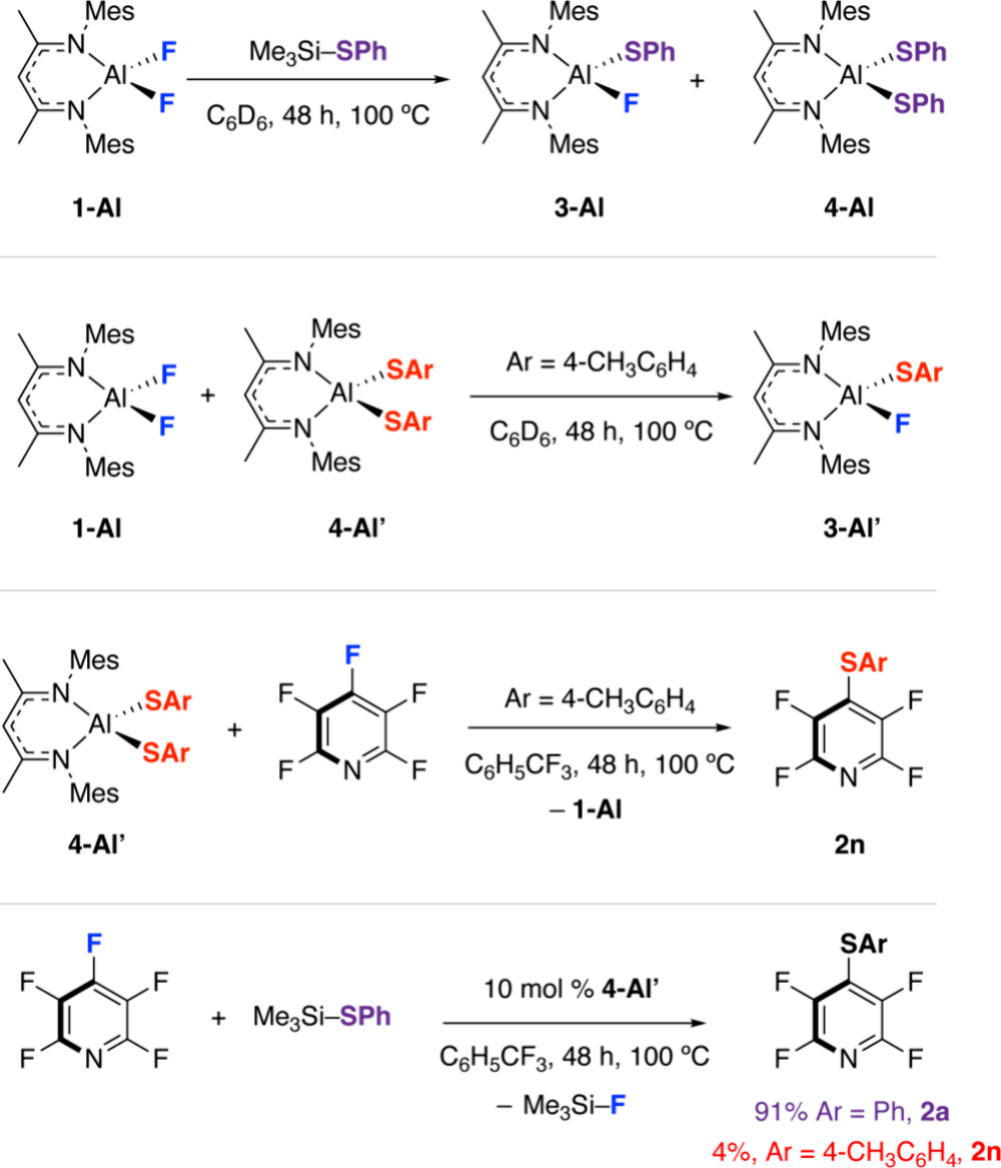
Catalytic Relevance of Group 13 Thiolate Complexes Stoichiometric reaction between **1-Al** and Me_3_Si–SPh. Reaction of an isolated dithiolate complex with **1-Al** and pentafluoropyridine under stoichiometric and catalytic conditions.

To better understand the role of the catalyst, in two separate experiments, **1-B** and **1-Al** were reacted with 3 equiv of Me_3_Si–SPh. In the case of **1-B**, no apparent reaction occurred,^36^ and **1-Al** reacted with Me_3_Si–SPh after 48h at 100 °C in C_6_D_6_ to produce a 3:1 mixture of **3-Al** and **4-Al** along with Me_3_Si–F as a side product (Scheme 1). The latter was readily apparent from ^19^F NMR resonance at δ = −148.5 ppm. **4-Al** could be crystallized from the mixture, and the structure was determined by single-crystal X-ray diffraction. It is likely that ligand exchange between fluoride and thiolate ligands is facile under the conditions of catalysis. A reaction between an independently prepared sample of the dithiolate complex **4-Al′** and **1-Al** at 100 °C in C_6_D_6_ led to an intermolecular scrambling of these ligands with the formation of the cross-product **3-Al’**, as evidenced by a broad singlet also found at δ = −148.5 ppm in the ^19^F NMR spectrum (Scheme 1). **4-Al**′**** proved to be a competent nucleophile, as the reaction with pentafluoropyridine led to the formation of the defluorothioylation product **2n**. **4-Al**′**** was also shown to be catalytically active for the reaction of Me_3_Si–SPh with pentafluoropyridine under similar conditions as **1-Al**, in this case, forming **2a** as the major product along with small amounts of **2n** derived from the catalytic loading of **4-Al**′****.

### NaBAr^F^_4_ Promoted Hydrodefluorination of Arenes

Attempts to extend the catalytic methodology to the hydrodefluorination of arenes met with limited success. **1-B**, **1-Al**, and **1-Ga** were not active catalysts for the reaction of Et_3_SiH with pentafluoropyridine under catalytic conditions established for Me_3_Si–SPh. Following an extensive screen of conditions and additives, it was found that 10 mol % NaBAr^F^ could be used as an additive to effectively promote the reaction. Several silanes, including Et_3_SiH, Ph_2_SiH_2_, PhSiH_3_, Me_2_PhSiH, (MeO)_3_SiH, and (Me_2_SiH)_2_O, could be used as effective hydride sources with the reaction occurring in noncoordinating solvents, such as fluorobenzene or α,α,α-trifluorotoluene. α,α,α-Trifluorotoluene and fluorobenzene were used as solvents due to their high boiling points, modest dielectrics, and a known ability to solvate charge-separated species.

**Figure 4:**
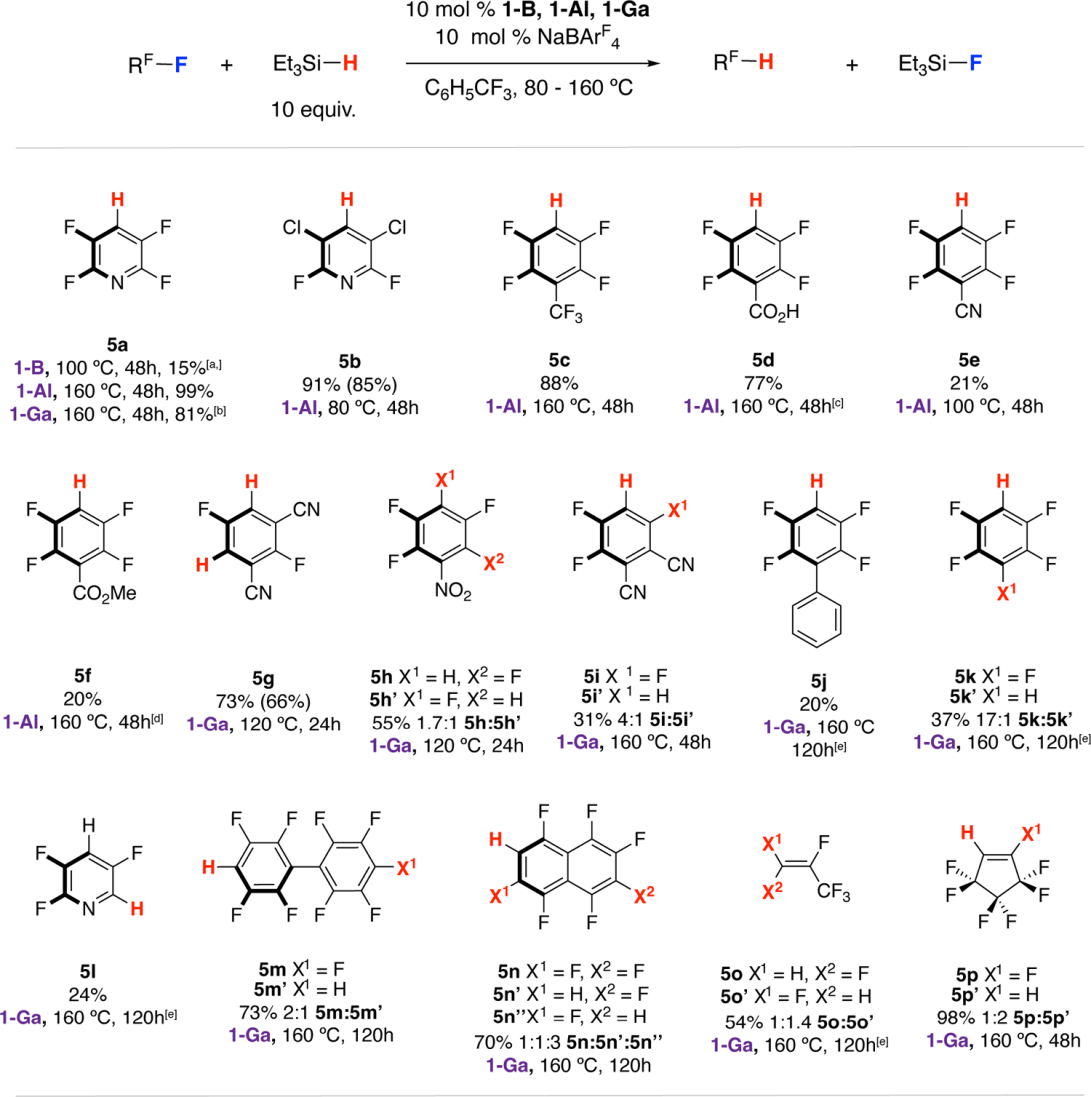
Scope of group 13 fluoride catalyzed hydrodefluorination of fluorinated arenes and alkenes. ^a^NMR yields measured by ^19^F NMR spectroscopy using 1,2-difluorobenzene as an internal standard. Isolated yields are in parentheses. ^b^Product formed alongside 12% **5l**. ^c^Me_2_PhSiH was used as a terminal reductant. ^d^Ph_3_SiH used as terminal reductant. ^e^20 mol % catalyst loading used.

Under optimized conditions, pentafluoropyridine could be converted into 2,3,5,6-tetrafluoropyridine **5a** in 99% using 10 mol % **1-Al** + 10 mol % NaBAr^F^_4_ as a precatalyst mixture and 10 equiv of Et_3_SiH as the terminal reductant. Control reactions using solely **1-Al** or NaBAr^F^_4_ led to minimal conversion; similarly, NaBPh_4_ was not an active additive under these conditions. Qualitatively, **1-Al** and **1-Ga** appeared to be more active catalysts than **1-B**. The scope of hydrodefluorination was investigated. A series of electron-deficient aromatics, including nitro, nitrile, ester, and acid functional groups, underwent hydrodefluorination (**5b**–**5n**) with more forcing conditions and higher loadings required for less electron-deficient substrates (e.g., **5j,k, 5m,n**). Fluorinated alkenes, such as perfluorocyclopentene and hexafluoropropene, were also reactive and could be transformed into hydrodefluorination products **5o:5o′** and **5p:5p**′****, respectively, in reasonable yields (Figure 4).

For comparison, contemporary approaches in main-group-catalyzed hydrodefluorination of fluoroarenes include catalysts based on P(III)/P(V) and Bi(I)/Bi(III) redox couples.^37−40^ The nucleophilic silicate complex TBAT has also been reported as a catalyst for hydrodefluorination of fluoroarenes with loadings as low as 0.1 mol % at 60 °C.^26^ Transition metal catalysts typically operate with a broader substrate range and improved efficiencies.^41^

**Scheme 2:**
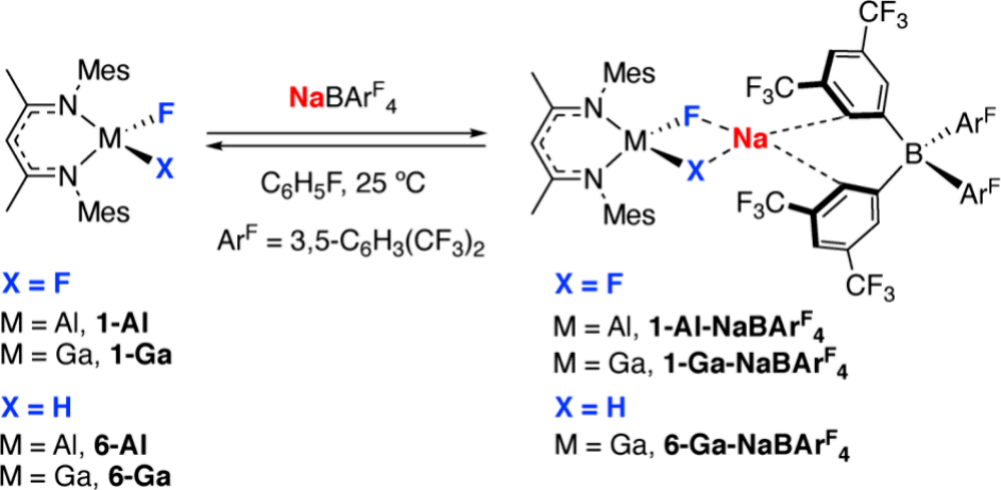
Proposed Reversible Reaction between **1-Al** or **1-Ga** and NaBAr^F^_4_

**Figure 5:**
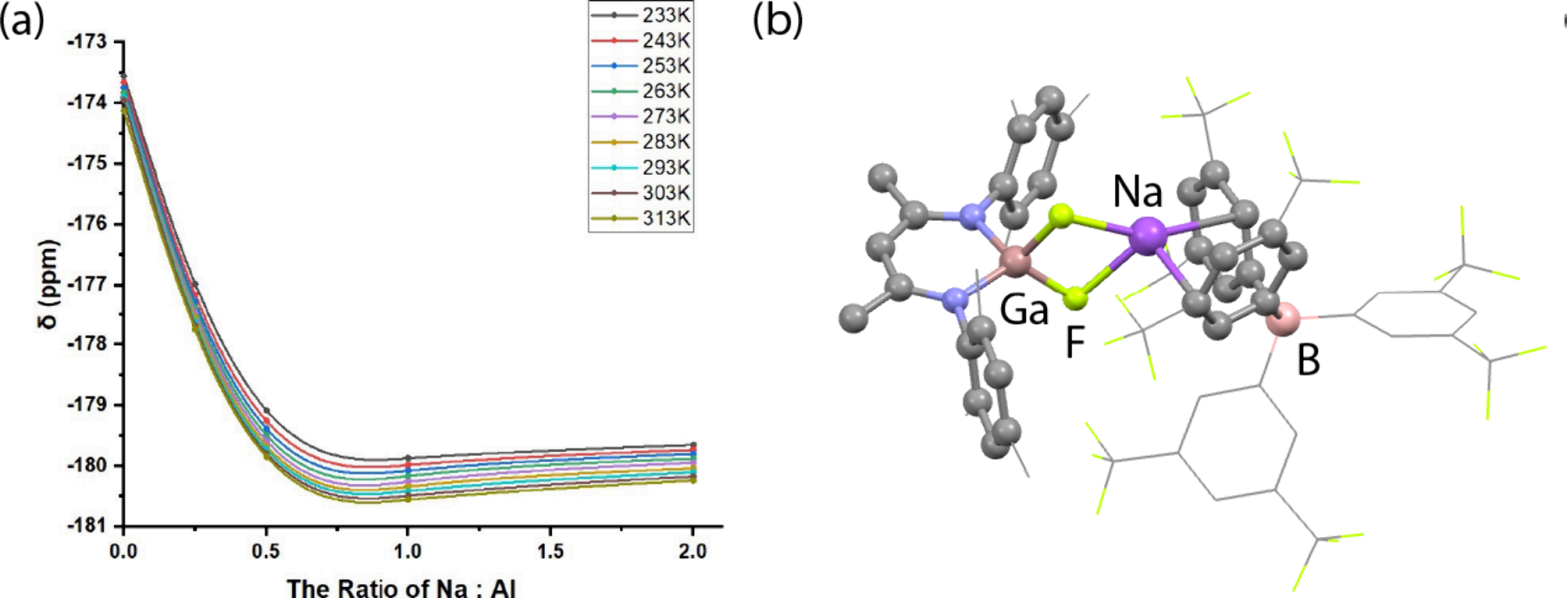
(a) VT ^19^F NMR spectroscopic data from the reaction of **1-Al** and NaBAr^F^_4_ at different Al:Na ratios. (b) Calculated structure of **1-Ga-NaBAr**^**F**^_**4**_.

The addition of **1-Al** to NaBAr^F^_4_ in fluorobenzenes suggests an interaction between the two precatalyst components (Scheme 2). Hence, at 298 K, a 50 mM solution of a 1:1 mixture of **1-Al** and NaBAr^F^_4_ displayed a defined and sharp ^19^F NMR resonance at δ = −180.2 ppm. **1-Al** alone demonstrates a resonance at δ = −174.5 ppm at the same concentration. Varying the ratio of NaBAr^F^_4_:**1-Al** from 0.5:1 to 2:1 suggests that the binding event is 1:1 as the maximum Δδ ≈ 6 ppm is observed at this ratio with only small changes in chemical shift occurring at higher ratios. The reaction between **1-Al** and NaBAr^F^_4_ was investigated at nine different temperatures across a 233–313 K temperature range, with consistent results supporting 1:1 binding at all temperatures (Figure 5a). No attempts were made to quantify these data, as it is likely that the equilibria at play are complicated by the potential for the solvent, fluorobenzene, to act as a competitive ligand for Na^+^. A similar reaction between a 1:1 mixture of **1-Ga** and NaBAr^F^_4_ at 298 K in fluorobenzene displayed an upfield shift of the ^19^F NMR resonance Δδ ≈ 2.5 ppm relative to **1-Ga**.

While the ^19^F NMR spectroscopic data support changes to the magnetic environment at fluorine upon the reaction of **1-Al** or **1-Ga** with NaBAr^F^_4_, these data in isolation do not give definitive information on the location of the Na^+^ ion within **1-Al-NaBAr**^**F**^_**4**_ or **1-Ga-NaBAr**^**F**^_**4**_. To probe the binding event, gallium hydride fluoride complex **6-Ga** was prepared and reacted with NaBAr^F^_4_ in the hope that the ^2^*J*_H–F_ coupling constant may shed light on changes to the Ga–F bonding on coordination to Na^+^. **6-Ga** displays a doublet resonance at δ = −182.0 ppm (d, ^2^*J*_H–F_ = 91.1 Hz) in the ^19^F NMR spectrum. The reaction with NaBAr^F^_4_ generates a new species assumed to be **6-Ga-NaBAr**^**F**^_**4**_ with δ = −197.3 ppm (d, ^2^*J*_H–F_ = 102.6 Hz). The latter demonstrates the expected upfield shift of the fluoride ligand Δδ ≈ 15 ppm, along with an increase of the ^2^*J*_H–F_ coupling constant by more than 10 Hz, consistent with changes to bonding of the {GaHF} motif on association with Na^+^. Transition metal fluoride complexes are known to be effective hydrogen bond (and halogen bond) donors.^42−44^ Examples of coordination of metal fluorides to s-block cations are, however, less common. DFT calculations were undertaken to understand the nature of the binding event. Coordination of NaBAr^F^_4_ to **1-Al** was calculated to be exergonic by ΔG°_(298 K)_ = −1.4 kcal mol^–1^, consistent with a reversible binding event. A similar coordination geometry and an exergonic reaction ΔG°_(298 K)_ = −2.9 kcal mol^–1^ was calculated for the reaction of NaBAr^F^_4_ with **1-Ga** (Figure 5b). NBO and AIM calculations support the characterization event as a weak electrostatic interaction between the fluorine atoms and Na^+^ center (see the Supporting Information, Section 6.4).

A plausible reaction mechanism for the hydrodefluorination of fluoroarenes involves (i) σ-bond metathesis between Et_3_SiH and the group 13 fluoride **1-M** forming the group 13 hydride complex and (ii) nucleophilic attack of the hydride on the fluorinated arene by a concerted S_N_Ar mechanism, liberating the hydrodefluorinated product and regenerating the group 13 fluoride (M= Al, Ga). We have previously shown that a β-diketiminate-stabilized aluminum dihydride complex is an effective reagent for the hydrodefluorination of electron-deficient arenes under forcing conditions.^23^

**Figure 6:**
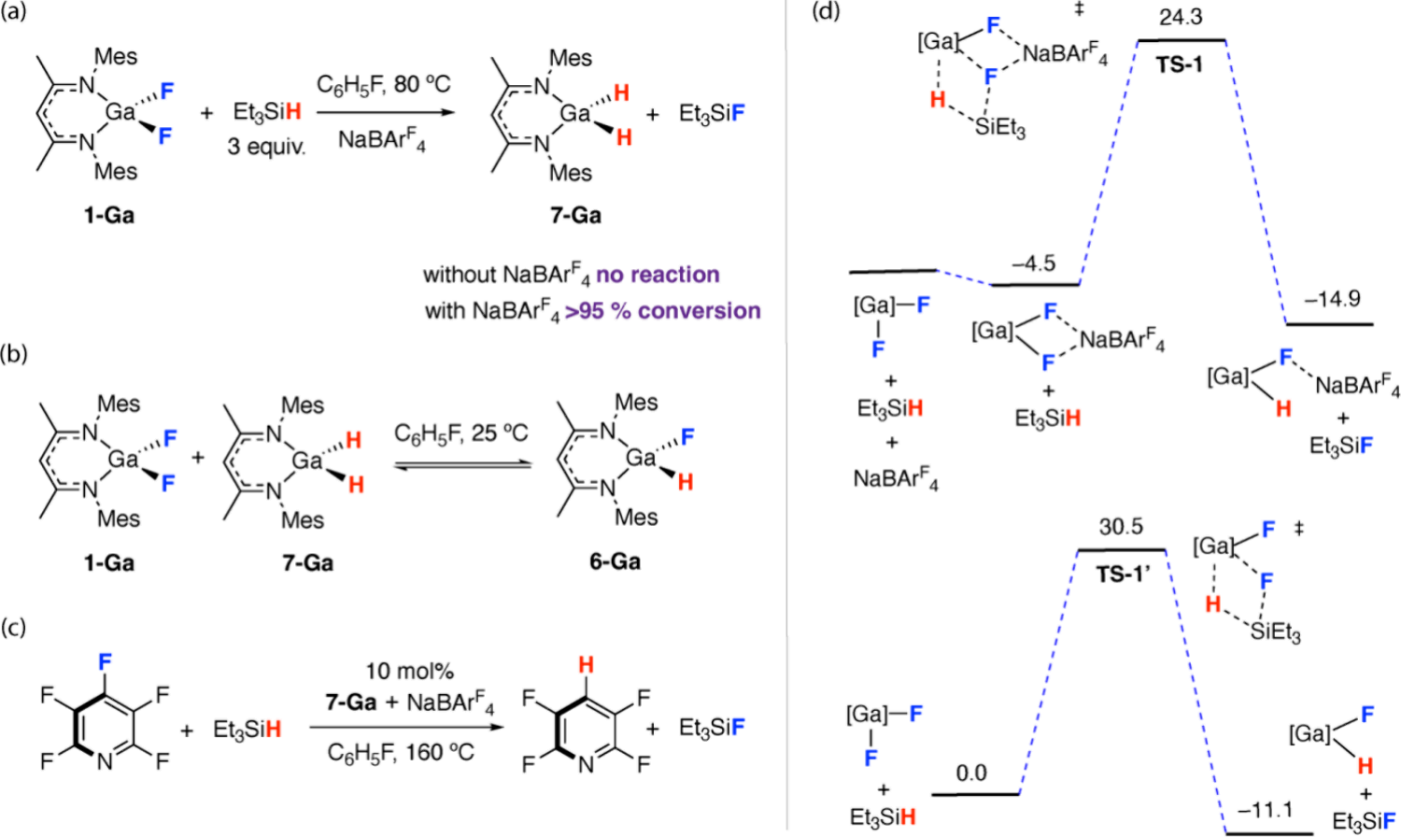
(a) Stoichiometric reactions between **1-Ga** and Et_3_SiH with and without NaBAr^F^_4_. (b) Lithium-based exchange between **1-Ga**, **7-Ga**, and **6-Ga**. (c) Hydrodefluorination of pentafluorobenzene catalyzed by **7-Ga** and NaBAr^F^_4_. (d) DFT calculated barriers for fluoride to hydride ligand metathesis for **1-Ga** with and without NaBAr^F^_4_. wB97xD/def2TZVPP/PCM(fluorobenzene) // wB97xD/6–31G**/6–311+G*/SDDAll/PCM(fluorobenzene). Gibbs energies are given in kcal mol^–1^.

Further experiments and calculations were undertaken to shed light on the role of NaBAr^F^_4_ during catalytic hydrodefluorination. **1-Ga** and **1-Ga-NaBAr**^**F**^_**4**_ show different reactivities with Et_3_SiH. While no apparent reaction is observed between **1-Ga** and Et_3_SiH up to 80 °C in fluorobenzene, the addition of 1 equiv of NaBAr^F^_4_ promotes the formation of **7-Ga** and Et_3_SiF (δ = – 176.1 ppm) after 6 h at 80 °C (Figure 6a). The expected kinetic product of this reaction, **6-Ga**, was shown to be in equilibrium with **1-Ga** and **7-Ga** at 25 °C in fluorobenzene (Figure 6b). DFT calculations support the role of NaBAr^F^_4_ in lowering the barrier for metathesis through fluoride coordination (Figure 6d). The reaction of **1-Ga-NaBAr**^**F**^_**4**_ with Et_3_SiH was calculated to occur through **TS-1** (ΔG^‡^_298 K_ = 28.8 kcal mol^–1^), leading to the direct formation of **6-Ga-NaBAr**^**F**^_**4**_. In the absence of the promoter, **1-Ga** was calculated to react with Et_3_SiH by a higher barrier through **TS-1’** (ΔG^‡^_298 K_ = 30.5 kcal mol^–1^) to form **6-Ga**. The lower energy of **TS-1** relative to **TS-1’** is explained through Na^+^ playing a role in charge stabilization during the polarization and breaking of the Ga---F bond.

Experimentally, it was found that **7-Ga** is an effective nucleophile for the hydrodefuorination of pentafluoropyridine at 100 °C and that 10 mol % **7-Ga+** NaBAr^F^_4_ can be used as a precatalyst for the hydrodefluorination of the same substrate with Et_3_SiH at 160 °C (Figure 6c).^45^ These findings suggest a synergistic role for Na^+^ and the group 13 complex in catalysis and are reminiscent of cooperative effects in not only the stoichiometric activation of fluoroarenes with heterometallic complexes but also catalytic applications of s/p heterobimetallic complexes in hydrogenation, hydroamination, hydrophosphination, and hydroboration of unsaturated substrates.^46,47^ Based on the current data, however, we cannot exclude alternative roles for NaBAr^F^_4_ in catalysis, including modification of the solvent polarity, or in the S_N_Ar step, due to coordination to the substrate and polarization of the reacting carbon–fluorine bond.

## Conclusions and Summary

In summary, a series of four-coordinate group 13 fluoride complexes (M = B, Al, Ga) are reported as catalysts for the thiodefluorination and hydrodefluorination of poly- and perfluoroarenes using silanes as terminal reagents. The scope of reactivity in the electrophile is broad, provided that the aromatic ring is electron-deficient. For thiodefluorination, mechanistic studies support a plausible role for group 13 thiolate intermediates, which are capable of C–S bond formation through nucleophilic attack on the aromatic ring. Similarly, for hydrodefluorination, group 13 hydrides are likely key catalytic intermediates. In both cases, catalytic turnover requires metathesis (ligand exchange) between the group 13 complex and silicon reagent; a step that requires breaking of strong group 13 metal–fluoride bonds. In the case of thiodefluorination, RS^–^-to-F^–^ metathesis occurs in the absence of an additive, while in the case of hydrodefluorination, H^–^-to-F^–^ exchange requires the use of NaBAr^F^_4_ as an additive. Stoichiometric experiments and DFT calculations are consistent with NaBAr^F^_4_ playing a role in activating and weakening the group 13 metal–fluoride bond through M–F---Na^+^ interactions, facilitating metathesis with silane reagents. This additive effect may have important implications for future catalyst design.^48^

## Experimental Section

### General Procedure for Thiodefluorination of Arenes

In a glovebox, the fluoroarene (0.05 mmol), Me_3_Si-X (Me_3_Si = trimethylsilane, X = SPh) reagent (0.15–0.5 mmol), catalyst (10–20 mol % **1-B**, **1-Al**, or **1-Ga**), and PhCF_3_ or PhF (dry and degassed) were added to a J. Young’s NMR tube and sealed. The total volume of the PhCF_3_ and C_6_D_6_ (5:1) or PhF and C_6_D_6_ (5:1) solution was made up of 0.6 mL. The reaction was monitored by ^19^F NMR spectroscopy and, depending on the substrate, heated between 60 and 160 °C (Figure 2). 1,2-Difluorobenzene in a sealed glass capillary containing C_6_D_6_ (δ = 138.3 ppm) was added as an internal standard, and the reaction mixture was analyzed by quantitative ^19^F NMR spectroscopy. Nonvolatile products were isolated and purified by column chromatography. For full details and characterization data on **2a**–**m**, see the Supporting Information.

### General Procedure for Hydrodefluorination of Fluoroarenes

In a glovebox, the fluoroarene (0.05 mmol), silane reagent (0.15–0.5 mmol), catalyst mixture (10–20 mol % **1-B**, **1-Al**, or **1-Ga** + 10–20 mol % NaBAr^F^_4_), and PhCF_3_ or PhF (dry and degassed) were added to a J-Young’s NMR tube and sealed. The total volume of the PhCF_3_ and C_6_D_6_ (5:1) or PhF and C_6_D_6_ (5:1) solution was made up to 0.6 mL. The reaction was monitored by ^19^F NMR spectroscopy and, depending on the substrate, heated between 80 and 160 °C (Figure 4). 1,2-Difluorobenzene in a sealed glass capillary containing C_6_D_6_ (δ = 138.3 ppm) was added as an internal standard, and the reaction mixture was analyzed by quantitative ^19^F NMR spectroscopy. Nonvolatile products were isolated and purified by column chromatography. For full details and characterization data on **5a**–**p**, see the Supporting Information.

### Computational Methods

DFT calculations were performed using Gaussian 09 (Revision D.01) using an ultrafine integration grid (int = ultrafine). Geometry optimizations and frequency calculations were performed using the ωB97xD density functional, including solvent corrections (PCM, fluorobenzene) with SDDAll (Na, Ga), 6-31G** (C, H) and 6-311+G* (N, F, Si) basis sets. Frequency analyses for all stationary points were performed using the enhanced criteria to confirm the nature of the structures as either minima (no imaginary frequency) or transition states (only one imaginary frequency). The electronic energies of the optimized geometries were calculated using the ωB97xD functional with solvent corrections (PCM, fluorobenzene) using the def2TZVPP basis sets for all atoms. The Gibbs free energy correction from the frequency calculation was added to this electronic energy to generate Gibbs free energy values (calculated at 298.15 K, 1 atm) for the stationary points. Intrinsic reaction coordinate calculations were used to connect transition states and minima located on the potential energy surface. NBO analysis was performed at the ωB97xD/def2TZVPP level using NBO 6.0 and is stated as such. QTAIM analysis was conducted by using the AIMAll package.

## Supporting Information Available

Synthetic procedures; kinetic experiments; NMR spectra of all compounds; crystallographic data; and computational methods (PDF)
Cartesian coordinates of the DFT-optimized structures (XYZ)

## Supplementary Material

ic4c05381_si_001.pdf

ic4c05381_si_002.xyz
