# Supplementary material for: Boron, Aluminum, and Gallium Fluorides as Catalysts for the Defluorofunctionalization of Electron-Deficient Arenes: The Role of NaBArF4 Promoters
Source: Inorg Chem. 2025 Mar 21;64(12):6092–9. doi: 10.1021/acs.inorgchem.4c05381 (PMC11962835; doi:10.1021/acs.inorgchem.4c05381)
Supplement: Supplementary file 1 — ic4c05381_si_001.pdf [file ic4c05381_si_001.pdf]

Supporting Information

Boron, Aluminium, and Gallium Fluorides as Catalysts for the Defluorofunctionalisation of Electron-Deficient Arenes: The Role of $NaBAr^F_4$ Promoters

Wenbang Yang, Andrew J. P. White and Mark R. Crimmin*

m.crimmin@imperial.ac.uk

Molecular Sciences Research Hub, Imperial College London, 82 Wood Lane, Shepherds Bush, W12 0BZ, UK.

Table of Contents

1) General experimental **S3**

2) Synthesis and Characterisation Data of Group 13 Catalysts **S4**

3) Group 13-catalyzed Defluorofunctionalisation Reactions **S10**

3.1) Optimization of the Reaction Conditions S10

3.2) General Procedure for Defluorothioylation S12

3.3) General Procedure for Hydrodefluorination S32

4) Single Crystal X-ray Diffraction Data **S51**

5) Mechanistic Studies of Group 13-catalyzed Defluorofunctionalisation Reactions **S55**

5.1) Mechanistic Proposal of Group 13 Catalytic Cycle: Fluoride Metathesis (Defluorothioylation) S55

5.2) Mechanistic Proposal of Group 13 Catalytic Cycle: Fluoride Metathesis (Hydrodefluorination) S61

6) DFT Studies **S73**

6.1) Computational methods S73

6.2) Calculated Reaction Pathway: Hydrodefluorination S74

6.3) Optimised structures S76

6.4) NBO Analysis S79

6.5) QTAIM Analysis S87

7) NMR Spectra **S94**

7.1) NMR Spectra of Group 13 complexes and the Precursors S94

7.2) NMR Spectra of the Isolated Products S101

7.3) ^{19}F-NMR Spectra for Determining the NMR Yields of Defluorofunctionalisation Reactions S129

8) Selected IR spectra **S142**

9) XYZ Coordinates **S143**

10) References **S161**

1) General experimental

Standard Schlenk line and glovebox techniques were used for all manipulations under an inert atmosphere of dinitrogen or argon unless otherwise stated. NMR scale reactions were performed in J. Young NMR tubes equipped with internal standard capillaries of ferrocene (^{1}H NMR spectroscopy) and prepared in a glovebox. An MBraun Labmaster glovebox was used, operating at <0.1 ppm H_2O and <0.1 ppm O_2.

Instruments: ^{1}H, ^{13}C, ^{19}F NMR spectra were recorded on BRUKER 400 MHz or 500 MHz machines, and referenced against $SiMe_4$ (^{1}H, ^{13}C), $CFCl_3$ (^{19}F)). All peaks are referenced against residual solvent and values are quoted in ppm. Data were processed using the MestReNova software package. The coupling constants (J) are reported in hertz (Hz). The following abbreviations are used to define multiplicities: s (singlet), d (doublet), t(triplet), q (quadruplet), sept. (septet), dd (doublet of doublets), ddd (doublet of doublets of doublets), dt (doublet of triplets), td (triplet of doublets), m (multiplet), dm (doublet of multiplets), br s (broad signal).

^{19}F NMR yields were determined by using 1,2-difluorobenzene as internal standard. (δ = 138.3 ppm in C_6D_6)

Single crystal X-ray data was obtained on Agilent Diffraction Xcalibur PX Ultra A and Xcalibur 3 E diffractometers, and the structures were refined using the SHELXTL, SHELX-97, and SHELX-2013 program systems.

Product isolation was performed using an automated flash column: Biotage® Selekt, Two Channel, single collection bed with UV Detector. Biotage® Sfär Silica D Duo 60 µm 50 g columns were used. Product mixtures were dried on silica (230-400 mesh) and loaded prior to loading. Isolations were performed using a 10:1 ratio of n-pentane: ethyl acetate. Alternative chromatography was conducted by manual columns on silica gel (technical grade, 230–400 mesh particle size, Sigma-Aldrich).

Chemicals: Solvents were dried over activated alumina from a solvent purification system (SPS) based upon the Grubbs design and de-gassed before use. Glassware was dried for >6 h prior to use at 120 °C. Benzene-d_6, fluorobenzene and trifluoro-toluene were de-gassed and stored over 3 Å molecular sieves before use. All reagents were acquired from Sigma Aldrich (Merck), Honeywell or Fluorochem and used without further purification unless specified. $LiAlH_4$ was purified by extraction in to Et_2O (5g in approximately 100 ml), followed by filtration and removal of the solvent under reduced pressure. The mixture was removed the last traces of solvent and the Colourless solid isolated proved soluble in Et_2O. $^{Mes}BDIBF_2$[S1] (^{Mes}BDI = {MesNC(Me)}$_2$CH), $^{Mes}BDIAlF_2$[S2], $^{Mes}BDIGaF_2$[S3], $^{Mes}BDIAlH_2$[S2] and $^{Mes}BDIGaH_2$[S3] were prepared by literature procedures.

2) Synthesis and Characterisation Data of Group 13 Catalysts

2.1. Starting Material Synthesis and Characterisation

2.1.1 Synthesis of ^{Mes}BDIBF$_2$

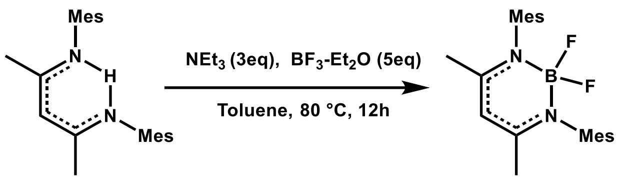

Procedure: ^{Mes}BDIH (1.67 g, 5.0 mmol) was dissolved in 100 ml of dry toluene. Triethylamine (1.52 g, 2.10 ml, 15.0 mmol) was slowly added, and then the solution was allowed to stir for over 30 min. After which, BF_3•OEt_2 (3.54 g, 3.10 ml, 25.0 mmol) was added to the solution, and the solution was heated at 80 °C for 14 hours. After cooling to 25 °C 20 ml of H_2O was added to quench excess BF_3•OEt_2. The organic and aqueous phases were separated, the light-yellow organic phase was then dried over $MgSO_4$, filtered under gravity and the solvent under reduced pressure. The crude product was obtained as a colourless solid which was then crystallized from 20 ml methanol. Yield = 1.15 g, 2.87 mmol, 60%.

^{1}H-NMR ($CDCl_3$, 298 K, 400 MHz) δ 6.89 (s, 4H, Ar***H***), 5.26 (s, 1H, C***H***), 2.26 (s, 12 H, o-Ar***Me***), 2.20 (s, 6H, p-Ar***Me***), 1.75 (s, 6H, C***Me***).

^{19}F-NMR ($CDCl_3$, 298 K, 376.5 MHz) δ -131.22 (q, $J_{B–F}$ = 19 Hz).

^{11}B-NMR ($CDCl_3$, 298 K, 128.0 MHz) δ 1.28 (t, $J_{B–F}$ = 19 Hz).

2.1.2 Synthesis of $^{Mes}BDIAlH_2$

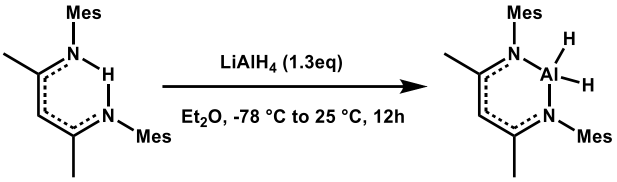

Procedure: In a glovebox, $LiAlH_4$ (0.56 g, 15.6 mmol.) and the $^{Mes}BDIH$ (4.01g, 12.0 mmol) pro-ligand were weighed in to separate schlenks. The schlenks were sealed, removed from the glovebox, and attached to a vacuum manifold. Dry Et_2O 24 ml was added to each schlenk to make up ≈ 0.5 M solutions of the reagents. The solution of the pro-ligand was cooled to -78 °C and the solution of $LiAlH_4$ added portion-wise over 5 minutes. The mixture was then allowed to warm to 25 °C, slightly below 25 °C H_2 evolution was observed along with formation of a heavy colourless precipitate assumed to be LiH. The mixture was stirred for 12 h at 25 °C, the precipitate allowed to settle, and the mixture filtered via a cannula. Concentration of the filtrate to approximately half the volume, followed by storage at -20 °C, filtration $^{Mes}BDIAlH_2$ as a colourless crystalline solid. $^{Mes}BDIAlH_2$ was isolated as a single crop of colourless crystals and dried under vacuum (2.17 g, 6.0 mmol, 50%).

^{1}H-NMR (C_6D_6, 298 K, 400 MHz) δ 6.75 (s, 4H, Ar***H***), 4.79 (s, 1H, C***H***), 2.33 (s, 12H, Ar***Me***), 2.10 (s, 6H, Ar***Me***), 1.48 (s, 6H, C***Me***).

2.1.3 Synthesis of $^{Mes}BDIAlF_2$

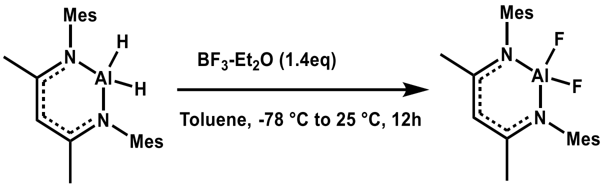

Procedure: In a glovebox, $^{Mes}BDIAlH_2$ (1.20 g, 3.3 mmol) was weighed and transferred to a schlenk. The schlenk was sealed removed from the box and attached to a vacuum manifold. Toluene (30 ml) was added via cannula and the reaction mixture cooled to -78 °C. BF_3•OEt_2 (0.6 ml, 4.7 mmol, 1.4 equiv.) was added via syringe and the mixture stirred for 30 min at this temperature and then warmed to 25 °C and stirred for 14 hours. The reaction mixture was filtered, and the toluene removed under reduced pressure. The crude reaction product was washed with n-pentane (20 ml) and dried under vacuum to give $^{Mes}BDIAlF_2$ as a colourless powder (0.98 g, 2.47 mmol, 75 %).

^{1}H-NMR (C_6D_6, 298 K, 400 MHz) 6.71 (s, 4H, Ar***H***), 4.88 (s, 1H, C***H***), 2.28 (s, 12 H, o-Ar***Me***), 2.07 (s, 6H, p-Ar***Me***), 1.44 (s, 6H, C***Me***).

^{19}F-NMR (C_6D_6, 298 K, 376.5 MHz) δ -174.5 (Al***F***).

2.1.4 Synthesis of ^{Mes}BDIGaH$_2$

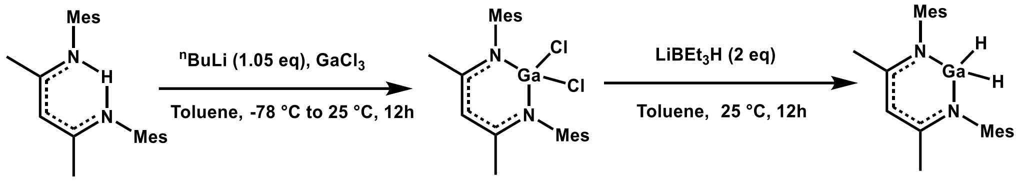

Procedure: At -78 °C a solution of ^{Mes}BDIH (1.67 g, 5 mmol) in toluene (30 ml) was treated dropwise with ^{n}BuLi (1.6 M in n-hexane, 3.28 ml, 5.25 mmol) and the reaction mixture was allowed to warm up to 25 °C for 3 hours. To the pale-yellow reaction mixture, a solution of GaCl$_3$ (0.92 g, 5.25 mmol) in toluene (30 ml) was slowly added at –78 °C and allowed to warm to 25 °C and stirred for 14 hours. The reaction mixture was filtered, and the filtrate was dried in vacuo to obtain ^{Mes}BDIGaCl$_2$ as a colourless solid. Further purification can be achieved by washing the crude product with n-pentane (20 ml). Yield: 1.23 g, 2.6 mmol, 52%.

A solution of ^{Mes}BDIGaCl$_2$ (944 mg, 2 mmol) in toluene (20 ml) was treated dropwise with LiEt$_3$BH (1.0 m in THF, 4 ml, 4 mmol) at 25 °C and stirred overnight. The reaction mixture was filtered, washed with n-pentane (10 ml) and the filtrate was dried in vacuo and the crude solid crystallized in 2 ml dry Et$_2$O to obtain ^{Mes}BDIGaH$_2$ as a colourless solid. Yield: 0.49 g, 1.2 mmol, 60%.

^{Mes}BDIGaCl$_2$:

^{1}H-NMR (C$_6$D$_6$, 298 K, 400 MHz) δ 6.73 (s, 4H, Ar***H***), 4.76 (s, 1H, C***H***), 2.35 (s, 12H, Ar***Me***), 2.06 (s, 6H, Ar***Me***), 1.42 (s, 6H, C***Me***).

^{Mes}BDIGaH$_2$:

^{1}H-NMR (C$_6$D$_6$, 298 K, 400 MHz) δ 6.75 (s, 4H, Ar***H***), 5.28 (bs, H, Ga***H***), 4.70 (s, 1H, C***H***), 2.31 (s, 12H, Ar***Me***), 2.11 (s, 6H, Ar***Me***), 1.52 (s, 6H, C***Me***).

2.1.5 Synthesis of ^{Mes}BDIGaF$_2$

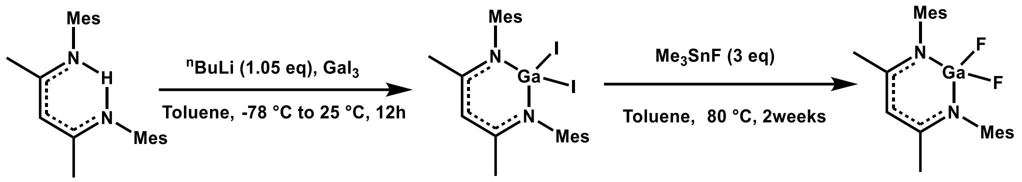

Procedure: At -78 °C a solution of ^{Mes}BDIH (1.67 g, 5 mmol) in toluene (30 ml) was treated dropwise with ^{n}BuLi (1.6 m in n-hexane, 3.28 ml, 5.25 mmol) and the reaction mixture was allowed to warm to 25 °C then stirred for 3 h at this temperature. To the pale-yellow mixture, a solution of GaI_3 (2.36 g, 5.25 mmol) in toluene (30 ml) was slowly added at -78 °C and allowed to warm to 25 °C and stirred for 14 hours. The reaction mixture was filtered, and the filtrate was dried in vacuo to obtain ^{Mes}BDIGaI$_2$ as a colourless solid. Further purification can be achieved by washing the crude product with n-pentane (20 ml). Yield 1.97 g, 3.0 mmol, 60%.

A solution of ^{Mes}BDIGaI$_2$ (657mg, 1 mmol) and Me_3SnF (548 mg, 3 mmol) in toluene (40 ml) was heated at 70 °C for 2 weeks. The reaction mixture was filtered, and the solvent removed under reduced pressure, the crude product was washed with n-pentane (10 ml) and then crystallized in dry Et_2O (5 ml) to obtain ^{Mes}BDIGaF$_2$ as a colourless solid. Yield: 44.2 mg, 0.1 mmol, 10%.

^{Mes}BDIGaI$_2$:

^{1}H-NMR (C_6D_6, 298 K, 400 MHz) δ 6.68 (s, 4H, Ar***H***), 4.92 (s, 1H, C***H***), 2.43 (s, 12 H, o-Ar***Me***), 2.05 (s, 6H, p-Ar***Me***), 1.43 (s, 6H, C***Me***).

^{13}C-NMR (C_6D_6, 100 MHz, 298 K) δ 170.0 (s, Ar***C***H), 139.0 (s, Ar***C***H), 135.7 (s, Ar***C***), 132.6 (s, Ar***C***), 129.9 (s, Ar***C***), 97.2 (s, Ar***C***H), 22.6 (s, p-Mes***Me***) , 20.5 (s, NC***Me***), 17.5 (s, o-Mes***Me***).

^{Mes}BDIGaF$_2$:

^{1}H-NMR (C_6D_6, 298 K, 400 MHz) δ 6.69 (s, 4H, Ar***H***), 4.75 (s, 1H, C***H***), 2.31 (s, 12 H, o-Ar***Me***), 2.07 (s, 6H, p-Ar***Me***), 1.43 (s, 6H, C***Me***).

^{19}F-NMR (C_6D_6, 298 K, 376.5 MHz) δ -200.7 (Ga***F***).

^{13}C-NMR (100 MHz, 298 K, C_6D_6) δ 172.0 (s, Ar***C***H), 139.5 (s, Ar***C***H), 136.3 (s, Ar***C***), 133.1 (s, Ar***C***), 129.6 (s, Ar***C***), 94.6 (s, Ar***C***H), 21.9 (s, p-Mes***Me***), 20.5 (s, NC***Me***), 17.9 (s, o-Mes***Me***).

2.1.6 Synthesis of ^{Mes}BDIAl(S-C_6H_4Me)$_2$

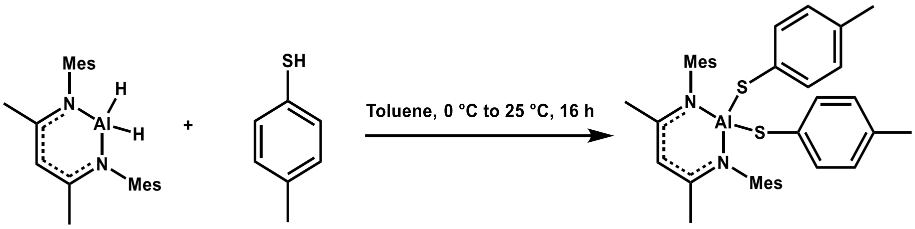

Procedure: In a glovebox, ^{Mes}BDIAlH_2 (1.20 g, 3.3 mmol) was weighed and transferred to a schlenk. The schlenk was sealed, removed from the box and attached to a vacuum manifold. Toluene (30 ml) was added via cannula. After which, 4-methylbenzenethiol (0.82 g, 6.6 mmol) was weighed and transferred to a schlenk. Toluene (30 ml) was added via cannula and the solution cooled to 0 °C. The solution of ^{Mes}BDIAlH_2 was transferred into the solution of 4-methylbenzenethiol via cannula at 0 °C and the mixture stirred for 30 min at this temperature and then warmed to 25 °C and stirred for 16 hours. The reaction mixture was filtered and then concentrated to 5 ml under reduced pressure. 20 ml n-pentane was slowly added, and the crude product crystallized by storage in a -20°C freezer for 2 days to obtain ^{Mes}BDIAl(S-C_6H_4Me)$_2$ as a colourless solid. (0.91 mg, 1.5 mmol, 45 %).[4]

^{1}H-NMR (C_6D_6, 298 K, 400 MHz) δ 7.04 – 7.00 (m, 4H, Ar***H***), 6.77 (s, 4H, Ar***H***), 6.69 (d, *J* = 7.9 Hz, 4H, Ar***H***), 5.12 (s, 1H, C***H***), 2.37 (s, 12H, p-Ar***Me***), 2.11 (s, 6H, C***Me***), 1.97 (s, 6H, C***Me***), 1.49 (s, 6H, o-Ar***Me***).

^{13}C-NMR (C_6D_6, 100 MHz, 298 K) δ = 171.6 (s, Ar***C***H), 139.8 (s, Ar***C***H), 136.3(s, Ar***C***), 134.3 (s, Ar***C***H), 133.7(s, Ar***C***), 132.8 (s, Ar***C***H), 130.1 (s, Ar***C***H), 129.0 (s, Ar***C***H), 128.3 (s, Ar***C***), 98.7 (s, Ar***C***H), 23.0 (s, p-Mes***Me***), 21.3 (s, Ar***Me***), 20.8 (s, NC***Me***), 19.4 (s, o-Mes***Me***).

[Since the PhSH, thiophenol, is volatile and highly toxic, 4-methylbenzenethiol was used as a suitable replacement to access mechanistically relevant aluminium(III) thiolate complexes]

3) Group 13-catalyzed Reactions

3.1. Optimization of the Reaction Conditions

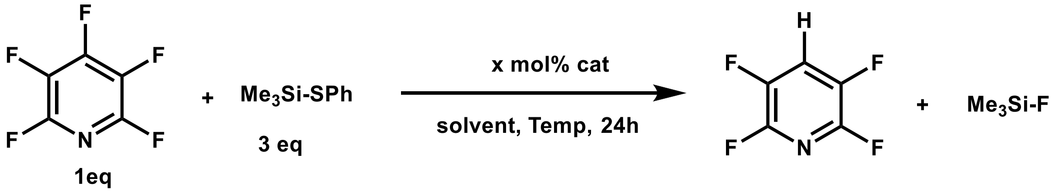

Table S1: Defluorothiolation of pentafluoropyridine using different $^{Mes}BDIMF_2$ and solvents[a]

	Solvent	Cat%	Tem	Time	Yield[b]
Me_3Si-SPh	$C_6H_5CF_3$	10% $^{Mes}BDIBF_2$	60 °C	24h	99%
Me_3Si-SPh	C_6H_5F	10% $^{Mes}BDIBF_2$	60 °C	24h	99%
Me_3Si-SPh	$C_6H_5CF_3$	10% $^{Mes}BDIAlF_2$	80 °C	24h	99%
Me_3Si-SPh	$C_6H_5CF_3$	10%$^{Mes}BDIAlF_2$	80 °C	24h	23%
Me_3Si-SPh	$C_6H_5CF_3$	5% $^{Mes}BDIAlF_2$	80 °C	24h	55%
Me_3Si-SPh	$C_6H_5CF_3$	10% $^{Mes}BDIGaF_2$	100 °C	24h	15%
Me_3Si-SPh	$C_6H_5CF_3$	no	100 °C	24h	10%

a: pentafluoropyridine (0.05 mmol), 0.6 mL solvent, con = 0.083 M;
b: Yields calculated by quantitative ^{19}F-NMR using 1,2-difluorobenzene as internal standard.

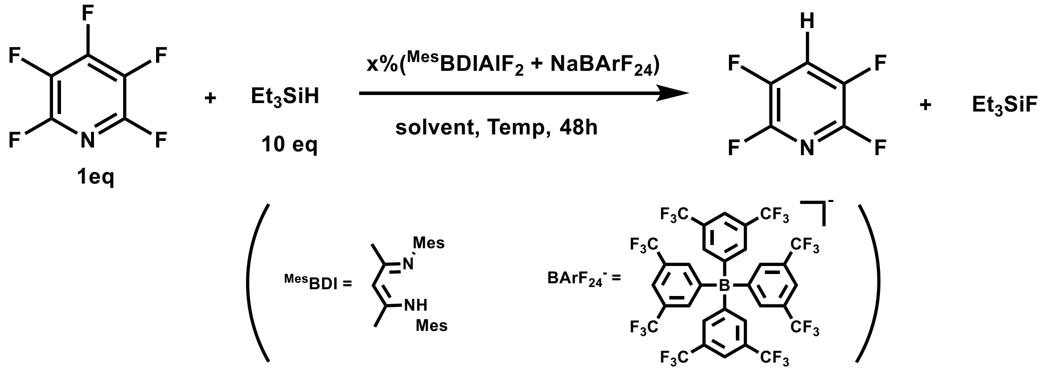

Table S2: Hydrodefluorination of pentafluoropyridine using different $^{Mes}BDIAlF_2$ and solvents[a]

	Solvent	Cat%	Temp	Yield[b]
Et_3SiH	C_6D_6	20%	100°C	10%
Et_3SiH	THF	20%	100°C	35%
Et_3SiH	1,4-dioxane	20%	100°C	12%
Et_3SiH	C_6H_5F	20%	100°C	100%
Et_3SiH	C_6H_5F	10%	100°C	40%
Et_3SiH	C_6H_5F	10%	160°C	96%
Et_3SiH	$C_6H_5CF_3$	10%	160°C	99%

a: pentafluoropyridine (0.05 mmol), 0.6 mL solvent, con = 0.083 M;
b: Yields calculated by quantitative ^{19}F-NMR using 1,2-difluorobenzene as internal standard.

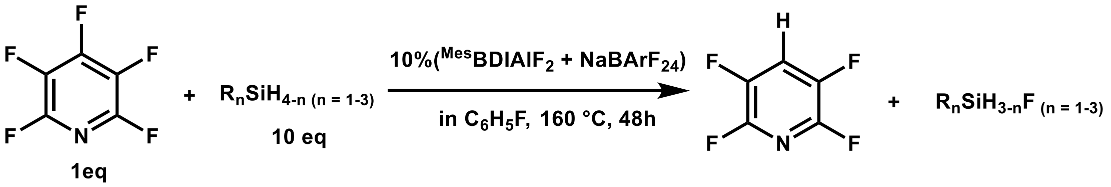

Table S3. Hydrodefluorination of pentafluoropyridine using different hydrosilanes[a]

	Conversion	Yield[b]
Et_3SiH	100%	96%
Et_2SiH_2	100%	87%
Me_2PhSiH	100%	96%
$PhSiH_3$	37%	37%
Ph_2SiH_2	47%	47%
Ph_3SiH	24%	9%
$(MeO)_3SiH$	22%	23%
$(EtO)_3SiH$	100%	95%
$(Me_2SiH)_2O$	100%	92%
$(iPr)_3SiH$	0%	0%

a: pentafluoropyridine (0.05 mmol), 0.6 mL solvent, con = 0.083 M; ;
b: Yields calculated by quantitative ^{19}F-NMR using 1,2-difluorobenzene as internal standard.

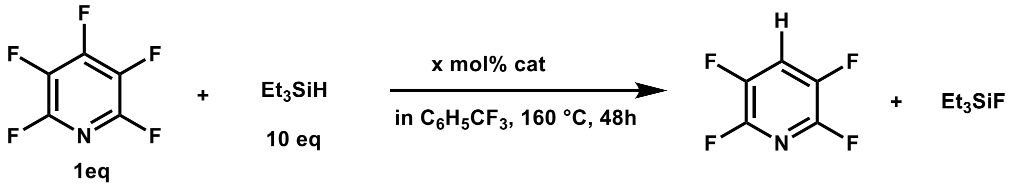

Table S4. Hydrodefluorination of pentafluoropyridine using different ^{Mes}BDIMF$_2$ catalysts[a]

Cat	Yield[b]
10% (^{Mes}BDIBF$_2$+NaBArF$_{24}$)	15%
10% (^{Mes}BDIAlF$_2$+NaBArF$_{24}$)	99%
10% (^{Mes}BDIGaF$_2$+NaBArF$_{24}$)	99%
20% NaBArF$_{24}$	0%
100% NaBArF$_{24}$	30%
20% ^{Mes}BDIBF$_2$	0%
20% ^{Mes}BDIAlF$_2$	0%
20% ^{Mes}BDIGaF$_2$	0%
50% LiF	0%
50% NaF	0%
5% (^{Mes}BDIAlF$_2$+NaBArF$_{24}$)	32%
10% (^{Mes}BDIAlF$_2$+NaBPh$_4$)	0%

a: pentafluoropyridine (0.05 mmol), 0.6 mL solvent, con = 0.083 M;
b: Yields calculated by quantitative ^{19}F-NMR using 1,2-difluorobenzene as internal standard.

3.2. General Procedure for Defluorofunctionalisation

In a glovebox, fluoroarene (0.05 mmol), Me_3Si-X (Me_3Si = trimethylsilane, X = SPh) reagent (0.15-0.5 mmol), cat. (10-20% ^{Mes}BDIM-F_2, M = B, Al, Ga) and $PhCF_3$ or PhF (dry and degassed) were added to a J-Young's NMR tube and sealed. The total volume of the $PhCF_3$ and C_6D_6 (5:1) or PhF and C_6D_6 (5:1) solution was made up to 0.6 ml. After reaction at room temperature or 160 °C, the hydrodefluorination product was purified by column chromatography. If the product was volatile, 1,2-difluorobenzene in a sealed glass capillary containing C_6D_6 (δ = 138.3 ppm) was added as internal standard and the reaction mixture was analyzed by quantitative ^{19}F-NMR spectroscopy.

- **Control experiment:**

A series of control experiments were conducted to ensure the internal standards were stable under the reaction conditions.

A sealed glass capillary containing C_6D_6 was placed in a J. Young NMR tube. PhF (0.6ml) or $PhCF_3$ (0.6ml), and Me_3Si-X reagent (0.50 mmol) were added to the J. Young NMR tube. The reaction mixture was heated to 160 °C for 48 h. The thiodefluorination products were not observed by either ^{1}H or ^{19}F NMR spectroscopy.

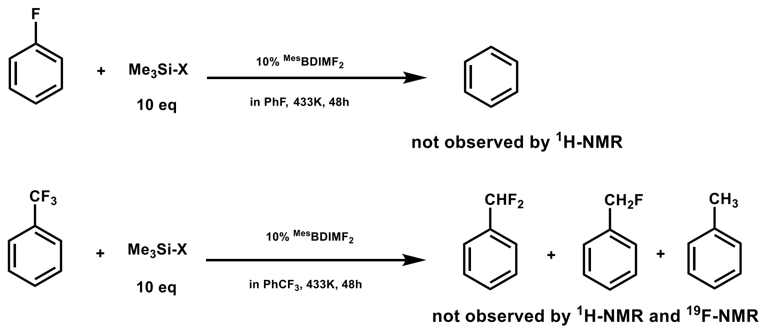

3.2.1. Results and Characterisation Data of Defluorofunctionalisation Products

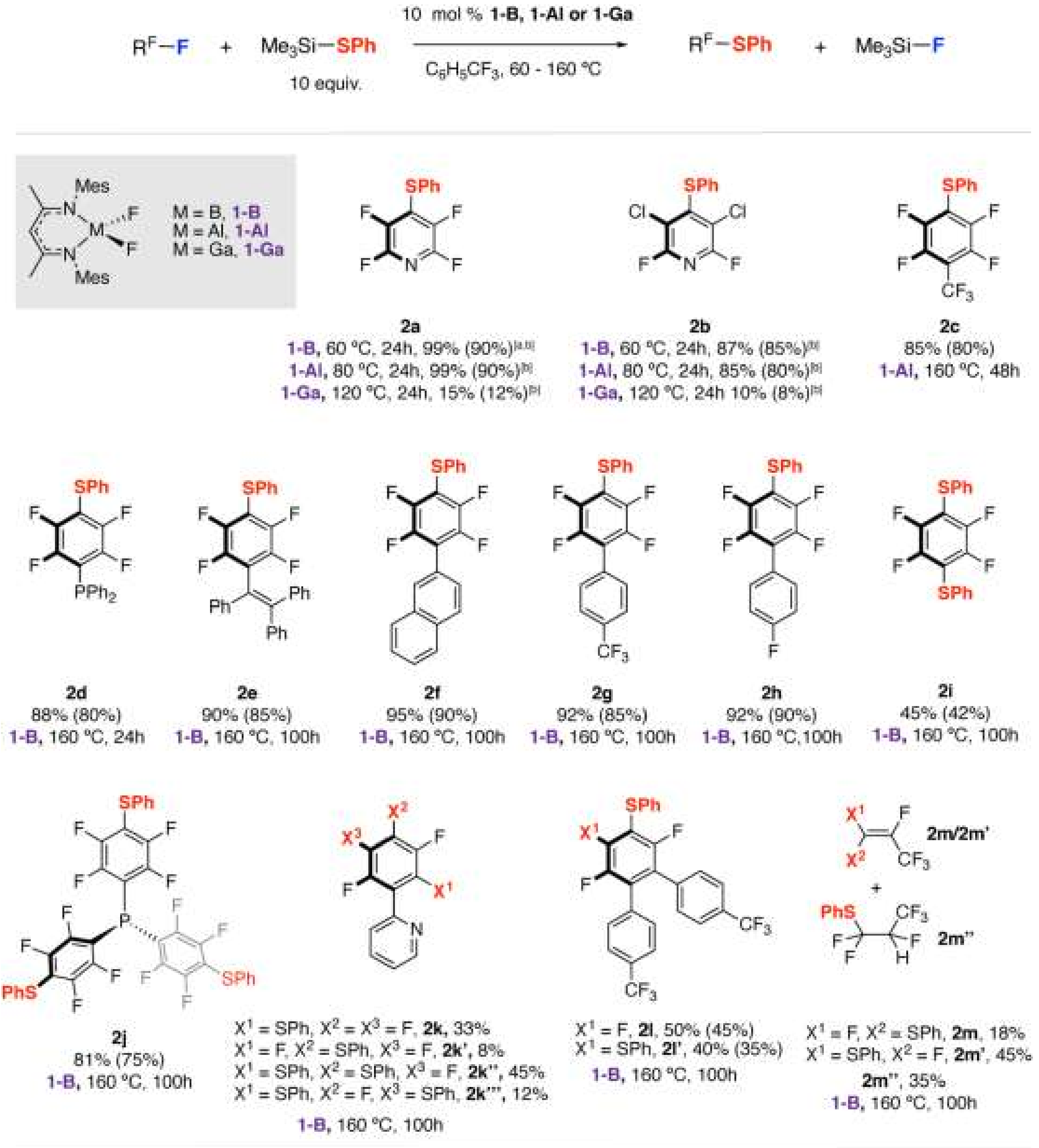

Figure S1. Scope of group 13 fluoride catalysed defluorothioylation of fluorinated arenes and hexafluoropropene. [a] NMR yields measured by ^{19}F NMR spectroscopy using 1,2-difluorobenzene as an internal standard. Isolated yields in parantheses. [b] Reactions run using 3 equiv. of Me_3Si–SPh

- Thiodefluorination of pentafluoropyridine

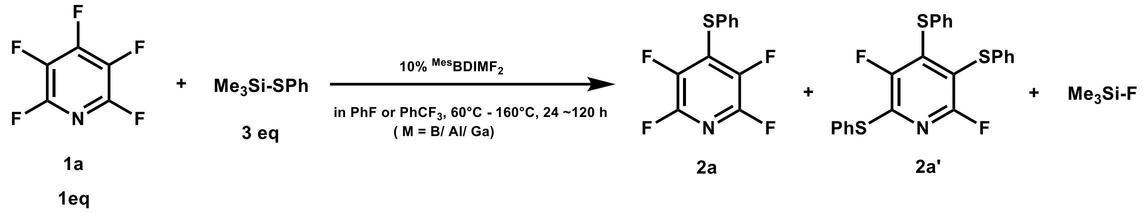

Following the general procedure, the reaction was performed with pentafluoropyridine (42.4 mg, 0.25 mmol), Me_3Si-SPh (142 mg, 0.75 mmol, 3 equiv.) and (^{Mes}BDIMF$_2$, 25 µmol, 10 mol%) at 60 – 120 °C for 24 h. Quantitative ^{19}F-NMR revealed that 2,3,5,6-tetrafluoro-4-(phenylthio)pyridine (**2a**) was formed as shown in the table below. 2,3,5,6-tetrafluoro-4-(phenylthio)pyridine (**2a**) was obtained as a colourless crystalline solid after purification by column chromatography on silica gel (n-pentane).

Catalyst	Mass (mg)	Temp (℃)	2a NMR yield (%)	2a isolated yield (%)
^{Mes}BDIBF$_2$	9.5	60	99	90
^{Mes}BDIAlF$_2$	10.0	80	99	90
^{Mes}BDIGaF$_2$	11.3	120	15	12

2,3,5,6-Tetrafluoropyridine (**2a**)

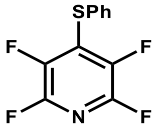

^{1}H NMR (400 MHz, $CDCl_3$): 7.55 (dt, $^3J_{H\text{–}H}$ = 7.7 Hz, 1.7 Hz, 2H, Ar–***H***), 7.47 – 7.36 (m, 3H, Ar–***H***).

^{13}C NMR (101 MHz, $CDCl_3$): 145.2 – 144.4 (m, Ar***C***), 142.62 – 141.90 (m, Ar***C***), 140.05 – 139.30 (m, Ar***C***), 132.88 (s, Ar***C***H), 131.25 – 130.69 (m, Ar***C***), 129.60 (s, Ar***C***H), 129.44 (s, Ar***C***H), 129.10 (s, Ar***C***H).

^{19}F NMR (376.5 MHz, $CDCl_3$): δ -90.2 – -90.8 (m, 2F, o-F), -136.0 – -136.9 (m)

^{19}F NMR (376.5 MHz, in $PhCF_3$ with a sealed glass capillary containing 1,2-difluorobenzene in C_6D_6): δ -91.3 – -91.7 (m, 2F, o-F), -137.0 – -137.3 (m, 2F).

Following the general procedure, the reaction was performed with pentafluoropyridine (42.4 mg, 0.25 mmol), Me_3Si-SPh (473.4 mg, 2.5 mmol, 10 equiv.) and (^{Mes}BDIBF$_2$, 25 µmol, 10 mol%) at 160 °C for 120 h. Quantitative ^{19}F-NMR revealed that 2,5-difluoro-3,4,6-tris(phenylthio)pyridine (**2a'**) was formed as shown in the table below. 2,5-difluoro-3,4,6-tris(phenylthio)pyridine (**2a'**) was obtained as a colourless crystalline solid after purification by column chromatography on silica gel (n-pentane/ CH_2Cl_2 = 20:1, v/v).

Catalyst	Mass (mg)	Temp (℃)	2a NMR yield (%)	2a' NMR yield (%)	2a' isolated yield (%)
^{Mes}BDIBF$_2$	9.5	160	6	91	85

2,5-difluoro-3,4,6-tris(phenylthio)pyridine (**2a'**)

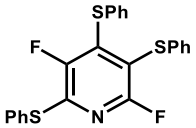

^{1}H NMR (400 MHz, $CDCl_3$): 7.57 (m, 2H, Ar–***H***), 7.48 – 7.38 (m, 5H, Ar–***H***), 7.34 (m, 3H, Ar–***H***), 7.32 – 7.29 (m, 3H, Ar–***H***), 7.27 – 7.21 (m, 2H, Ar–***H***).

^{13}C NMR (101 MHz, $CDCl_3$): 158.3 (d, $^1J_{C\text{–}F}$ = 237.0 Hz, Ar***C***), 151.9 (dd, $^1J_{C\text{–}F}$ = 254.0 Hz, $^3J_{C\text{–}F}$ = 5.0 Hz, Ar***C***), 146.7 (dd, $^2J_{C\text{–}F}$ = 22.0, $^3J_{C\text{–}F}$ = 15.0 Hz, Ar***C***), 142.4 (dd, $^2J_{C\text{–}F}$ = 14.0 Hz, $^3J_{C\text{–}F}$ = 1.0 Hz, Ar***C***), 135.4 (s, Ar***C***), 134.5 (s, Ar***C***), 131.9 (s Ar***C***), 131.8 (s Ar***C***), 129.6 (s, Ar***C***), 129.4 (s, Ar***C***), 129.3 (s, Ar***C***), 129.2 (s, Ar***C***), 128.5 (s, Ar***C***), 127.2 (s, Ar***C***), 127.0 (s, Ar***C***),127.0 (s, Ar***C***), 113.7 (m, Ar***C***). Some Ar***C*** resonances are overlapping.

^{19}F NMR (376.5 MHz, $CDCl_3$): δ -63.07 (d, $^3J_{F\text{–}F}$ = 27.4 Hz, 1F), -115.92 (d, $^3J_{F\text{–}F}$ = 28.0 Hz, 1F).

^{19}F NMR (376.5 MHz, in $PhCF_3$ with a sealed glass capillary containing 1,2-difluorobenzene in C_6D_6): δ -63.62 (d, $^3J_{F\text{–}F}$ = 27.6 Hz, 1F), -116.45 (d, $^3J_{F\text{–}F}$ = 27.6 Hz, 1F).

The spectral data matched with those reported in the literature.[S4-S6]

- Thiodefluorination of 3,5-dichloro-2,4,6-trifluoropyridine

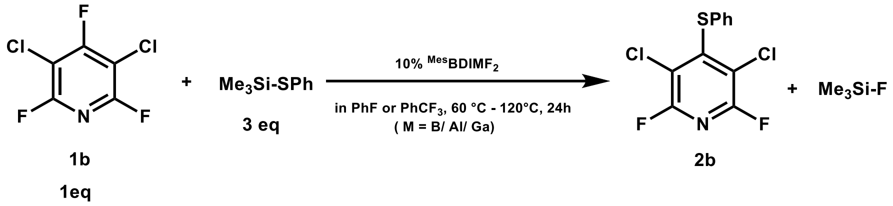

Following the general procedure, the reaction was performed with 3,5-dichloro-2,4,6-trifluoropyridine (40.5 mg, 0.25 mmol), Me_3Si-SPh (142 mg, 0.75 mmol, 3 equiv.) and $^{Mes}BDIMF_2$ (25 µmol, 10 mol%) at 60 – 120 °C for 24 h. Quantitative ^{19}F-NMR revealed that 2,3,5,6-tetrafluoro-4-(phenylthio)pyridine (**2b**) was formed as shown in the table below. 2,3,5,6-tetrafluoro-4-(phenylthio)pyridine (**2b**) was obtained as a colourless crystalline solid after purification by column chromatography on silica gel (n-pentane).

**Catalyst**	**Mass (mg)**	**Temp (℃)**	**2b NMR yield (%)**	**2b isolated yield (%)**
$^{Mes}BDIBF_2$	9.5	60	87	85
$^{Mes}BDIAlF_2$	10.0	80	85	80
$^{Mes}BDIGaF_2$	11.3	120	10	8

3,5-dichloro-2,6-difluoro-4-(phenylthio)pyridine (**2b**)

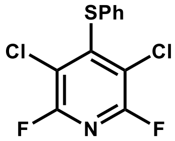

^{1}H NMR (400 MHz, $CDCl_3$): 7.38 – 7.29 (overlapping signals, 5H, Ar–***H***).

^{13}C NMR (101 MHz, $CDCl_3$): 155.0 (dd, $^1J_{C\text{–}F}$ = 247.7 Hz, $^2J_{C\text{–}F}$ = 14.8 Hz, Ar***C***), 151.0 (s, Ar***C***), 131.5 (s, Ar***C***), 131.0 (s, Ar***C***), 129.6 (s, Ar***C***H), 128.4 (s, Ar***C***H), 118.1 (m, Ar***C***).

^{19}F NMR (376.5 MHz, $CDCl_3$): δ -69.41 (s, 2F).

^{19}F NMR (376.5 MHz, in $PhCF_3$ with a sealed glass capillary containing 1,2-difluorobenzene in C_6D_6): δ -70.41 (s, 2F).

HRMS (APCL) m/z: $[M + H]^+$ Calcd for $C_{11}H_6F_2NS$ 291.9561; Found 291.9565.

- Thiodefluorination of octafluorotoluene

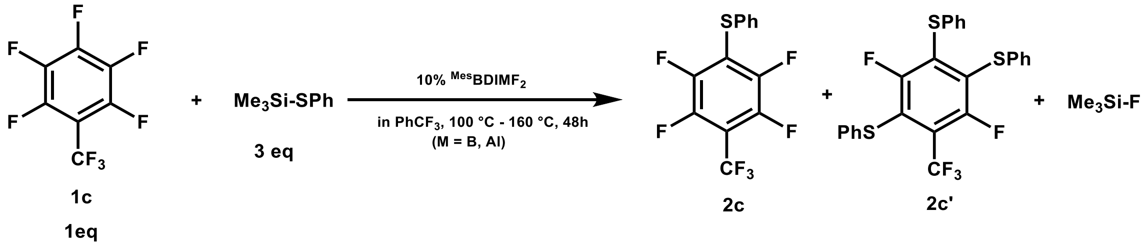

Following the general procedure, the reaction was performed with octafluorotoluene (59.0 mg, 0.25 mmol), Me_3Si-SPh (473.4 mg, 2.5 mmol, 10 equiv.) and $^{Mes}BDIMF_2$ (25 µmol, 10 mol%) at 100 – 160 °C for 48 h. Quantitative ^{19}F-NMR revealed that phenyl(2,3,5,6-tetrafluoro-4-(trifluoromethyl)phenyl)sulfane (**2c**) and (3,6-difluoro-5-(trifluoromethyl)benzene-1,2,4-triyl)tris(phenylsulfane) (**2c'**) were obtained as shown in the table below. Phenyl(2,3,5,6-tetrafluoro-4-(trifluoromethyl)phenyl)sulfane (**2c**) was obtained as a colourless crystalline solid after purification by column chromatography on silica gel (n-pentane). (3,6-difluoro-5-(trifluoromethyl)benzene-1,2,4-triyl)tris(phenylsulfane) (**2c'**) was obtained as a colourless crystalline solid after purification by column chromatography on silica gel (n-pentane/ CH_2Cl_2 = 10:1, v/v).

**Catalyst**	**Mass (mg)**	**Temp (°C)**	**2c NMR yield (%)**	**2c' NMR yield (%)**	**2c isolated yield (%)**	**2c' isolated yield (%)**
$^{Mes}BDIBF_2$	9.5	100	90	0	0	0
$^{Mes}BDIBF_2$	9.5	160	60	30	55	25
$^{Mes}BDIAlF_2$	10.0	160	85	0	0	0

phenyl(2,3,5,6-tetrafluoro-4-(trifluoromethyl)phenyl)sulfane (**2c**)

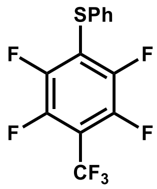

^{1}H NMR (400 MHz, $CDCl_3$): δ 7.52 – 7.48 (m, 2H, Ar–***H***), 7.39 – 7.37 (m, 3H, Ar–***H***).

^{13}C NMR (101 MHz, $CDCl_3$): δ 146.9 (dm, $^1J_{C\text{–}F}$ = 251.0 Hz, Ar***C***), 144.1 (dm, $^1J_{C\text{–}F}$ = 258 Hz, Ar***C***), 131.9 (s, Ar***C***H), 131.2 (s, Ar***C***), 129.5 (s, Ar***C***H), 128.8 (s, Ar***C***H), 120.6 (q, $^1J_{C\text{–}F}$ = 275.7 Hz, ***C***F_3), 120.4 (t, $^2J_{C\text{–}F}$ = 19.5 Hz, Ar***C***), 109.6 (m, Ar***C***).

^{19}F NMR (376.5 MHz, $CDCl_3$): δ -56.4 – -56.6 (m, 3F), -137.6 – -137.8 (m, 2F), -140.4 – -140.8 (m, 2F).

^{19}F NMR (376.5 MHz, in $PhCF_3$ with a sealed glass capillary containing 1,2-difluorobenzene in C_6D_6): δ -56.5 (t, $^3J_{F\text{–}F}$ = 21.7 Hz, 3F), -131.1 – -131.2 (m, 2F), -140.0 (m, 2F).

The spectral data matched with those reported in the literature.[S6]

(3,6-difluoro-5-(trifluoromethyl)benzene-1,2,4-triyl)tris(phenylsulfane) (**2c'**)

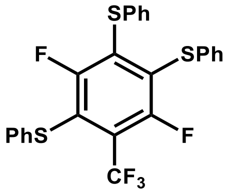

^{1}H NMR (400 MHz, $CDCl_3$): δ 7.36 – 7.11 (overlapping signals, 15H, Ar–***H***).

^{13}C NMR (101 MHz, $CDCl_3$): δ 157.9 (dd, $^1J_{C–F}$ = 248.0 Hz, $^3J_{C–F}$ = 3.4 Hz, Ar***C***), 154.8 (d, $^1J_{C–F}$ = 260.0 Hz, Ar***C***), 134.3 (s, Ar***C***), 134.2 (s, Ar***C***), 134.0 (s, Ar***C***), 133.5 (d, J = 3.0 Hz, Ar***C***), 129.9 (s, Ar***C***H), 129.7 (s, Ar***C***H), 129.3 (s, Ar***C***H), 129.1 (s, Ar***C***H), 127.5 (s, Ar***C***), 127.4 (s, Ar***C***), 124.5 (d, J = 25.0 Hz), 122.1 (q, J = 273 Hz, ***C***F_3), 120.7 (m, Ar***C***). Some Ar***C*** resonances are overlapping or cannot be observed.

^{19}F NMR (376.5 MHz, $CDCl_3$): δ -53.85 (d, $^2J_{F–F}$ = 35.0 Hz, 3F), -89.0 (d, $^4J_{F–F}$ = 16.0 Hz, 1F), -102.3 – -102.6 (qd, $^5J_{F–F}$ = 35.0, 16.0 Hz, 1F).

^{19}F NMR (376.5 MHz, in $PhCF_3$ with a sealed glass capillary containing 1,2-difluorobenzene in C_6D_6): δ -54.1 (d, $^2J_{F–F}$ = 35.2 Hz, 2F), -88.9 (d, $^4J_{F–F}$ = 16.3 Hz, 1F), -103.0 – -103.8 (qd, $^5J_{F–F}$ = 16.2 Hz, 1F).

The spectral data matched with those reported in the literature. [S6]

- Thiodefluorination of (perfluorophenyl)diphenylphosphane

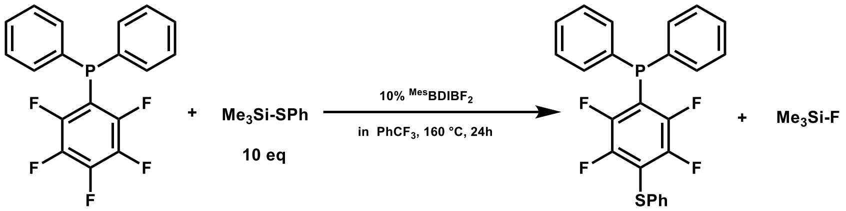

1d
1eq
2d

Following the general procedure, the reaction was performed with (perfluorophenyl)diphenylphosphane (88.1 mg, 0.25 mmol), Me_3Si-SPh (473.4 mg, 2.5 mmol, 10 equiv.) and $^{Mes}BDIBF_2$ (9.5 mg, 25 µmol, 10 mol%) at 160 °C for 24 h. Quantitative ^{19}F-NMR spectroscopy revealed that diphenyl(2,3,5,6-tetrafluoro-4-(phenylthio)phenyl)phosphane (**3r**) was formed in 88% yield. Diphenyl(2,3,5,6-tetrafluoro-4-(phenylthio)phenyl)phosphane (**3r**) (89.0 mg, 0.20 mmol, 80%) was obtained as a colourless crystalline solid after purification by column chromatography on silica gel (n-pentane: DCM = 10: 1).

diphenyl(2,3,5,6-tetrafluoro-4-(phenylthio)phenyl)phosphane (**2d**)

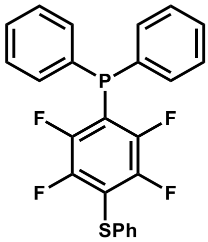

^{1}H NMR (400 MHz, C_6D_6): δ 7.47 (m, 2H, Ar–***H***), 7.44 (m, 6H, Ar–***H***), 7.40 (m, 3H, Ar–***H***), 7.33–7.32 (m, 4H, Ar–***H***).

^{13}C NMR (101 MHz, C_6D_6): δ 146.6 (dm, $^1J_{C\text{–}F}$ = 250.0 Hz, Ar***C***), 145.6 (ddm, $^1J_{C\text{–}F}$ = 248.0 Hz, $^2J_{C\text{–}F}$ =17.0 Hz, Ar***C***), 133.4 (m), 133.1 (d, $^2J_{C\text{–}F}$ = 21.4 Hz, Ar***C***H), 132.5 (m, Ar***C***H), 131.0 (m, Ar***C***H), 129.4 (d, $^3J_{C\text{–}F}$ = 3.0 Hz, Ar***C***H), 128.7 (d, $^3J_{C\text{–}F}$ = 7.3 Hz, Ar***C***H), 128.0 (s, Ar***C***H), 117.4 (dt, $^2J_{C\text{–}F}$ = 41.3 Hz, $^3J_{C\text{–}F}$ = 21.4 Hz, Ar***C***), 116.4 (t, $^3J_{C\text{–}F}$ = 20.1 Hz, Ar***C***). Some Ar***C***H resonances are overlapping and cannot be observed.

^{19}F NMR (376.5 MHz, C_6D_6): δ -127.4 (m, 2F), -132.3 (dd, $^3J_{F\text{–}F}$ = 12.9, 24.9 Hz, 2F).

^{31}P NMR (162 MHz, $CDCl_3$): δ -23.6 (t, $^3J_{P\text{–}H}$ = 36.9 Hz, 1P).

^{19}F NMR (376.5 MHz, in $PhCF_3$ with a sealed glass capillary containing 1,2-difluorobenzene in C_6D_6): δ –127.75 (ddd, $^3J_{F\text{–}F}$ = 36.9 Hz, $^4J_{F\text{–}F}$=24.4 Hz, $^5J_{F\text{–}F}$=13.0 Hz, 2F), –132.84 (dd, $^3J_{F\text{–}F}$ = 24.3 Hz, $^4J_{F\text{–}F}$ = 13.0 Hz, 2F).

^{31}P NMR (162 MHz, in $PhCF_3$ with a sealed glass capillary containing 1,2-difluorobenzene in C_6D_6): δ –23.1 (t, $^3J_{P\text{–}H}$ = 37.1 Hz, 1P).

HRMS (APCL) m/z: $[M + H]^+$ Calcd for $C_{24}H_{16}F_4PS$ 443.0641; Found 443.0635.

- Thiodefluorination of (2-(perfluorophenyl)ethene-1,1,2-triyl)tribenzene

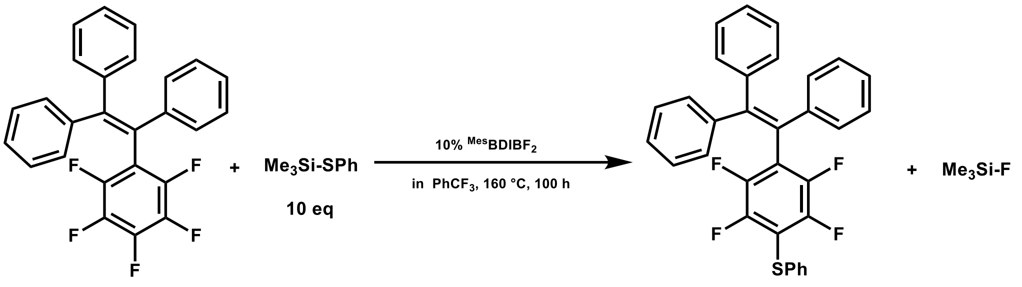

1e 2e

1eq

Following the general procedure, the reaction was performed with (2-(perfluorophenyl)ethene-1,1,2-triyl)tribenzene (105.5 mg, 0.25 mmol), Me_3Si-SPh (473.4 mg, 2.5 mmol, 10 equiv.) and $^{Mes}BDIBF_2$ (9.5 mg, 25 µmol, 10 mol%) at 160 °C for 100 h. Quantitative ^{19}F-NMR spectroscopy revealed that phenyl(2,3,5,6-tetrafluoro-4-(1,2,2-triphenylvinyl)phenyl)sulfane (**2e**) was formed in 90% yield. phenyl(2,3,5,6-tetrafluoro-4-(1,2,2-triphenylvinyl)phenyl)sulfane (**2e**) (108.8 mg, 0.212 mmol, 85%) was obtained as a colourless crystalline solid after purification by column chromatography on silica gel (n-pentane: EtOAc = 10:1).

phenyl(2,3,5,6-tetrafluoro-4-(1,2,2-triphenylvinyl)phenyl)sulfane (**2e**)

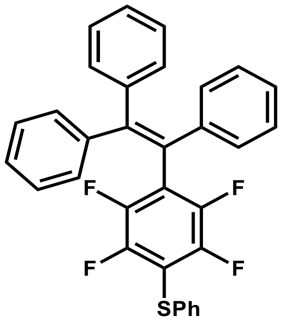

^{1}H NMR (400 MHz, C_6D_6): δ 7.35 – 7.29 (m, 4H, Ar–***H***), 7.27 – 7.20 (m, 12H, Ar–***H***), 7.15 – 7.06 (m, 4H, Ar–***H***).

^{13}C NMR (101 MHz, C_6D_6): δ 146.9 (ddm, $^1J_{C\text{-}F}$ = 246.0 Hz, $^2J_{C\text{-}F}$ =15.0 Hz), 147.8 (s, Ar***C***), 144.2 (dm, $^1J_{C\text{-}F}$ = 246 Hz), 142.3 (s, Ar***C***), 140.8 (s, Ar***C***), 139.6 (s, Ar***C***), 133.7 (s, Ar***C***), 130.9 (s, Ar***C***), 129.8 (s, Ar***C***), 129.3 (s, Ar***C***), 129.2 (s, Ar***C***), 129.1 (s, Ar***C***), 128.2 (s, Ar***C***), 127.9 (s, Ar***C***), 127.9 (s, Ar***C***), 127.8 (s, Ar***C***), 127.5 (s, Ar***C***), 127.3 (s, Ar***C***), 127.3 (s, Ar***C***), 125.7 (s, Ar***C***), 124.6 (t, $^2J_{C\text{-}F}$ = 18.8 Hz, Ar***C***H), 112.0 (t, $^2J_{C\text{-}F}$ = 20.5 Hz, Ar***C***H). Some Ar***C*** resonances are overlapping.

^{19}F NMR (376.5 MHz, C_6D_6): δ -133.4 (dd, $^3J_{F\text{–}F}$ = 24.5 Hz, $^4J_{F\text{–}F}$ = 11.9 Hz, 2F), -139.3 (dd, $^3J_{F\text{–}F}$ = 24.7 Hz, $^4J_{F\text{–}F}$ = 11.9 Hz, 2F).

^{19}F NMR (376.5 MHz, in $PhCF_3$ with a sealed glass capillary containing 1,2-difluorobenzene in C_6D_6): δ -133.6 (dd, $^3J_{F\text{–}F}$ = 24.8 Hz, $^4J_{F\text{–}F}$ = 12.1 Hz, 2F), -139.4 (dd, $^3J_{F\text{–}F}$ = 24.7 Hz, $^4J_{F\text{–}F}$ = 12.1 Hz, 2F).

HRMS (APCL) m/z: $[M + H]^+$ Calcd for $C_{32}H_{21}F_4S$ 513.1295; Found 513.1292.

The spectral data matched with those reported in the literature. [S7]

- Thiodefluorination of 2-(perfluorophenyl)naphthalene

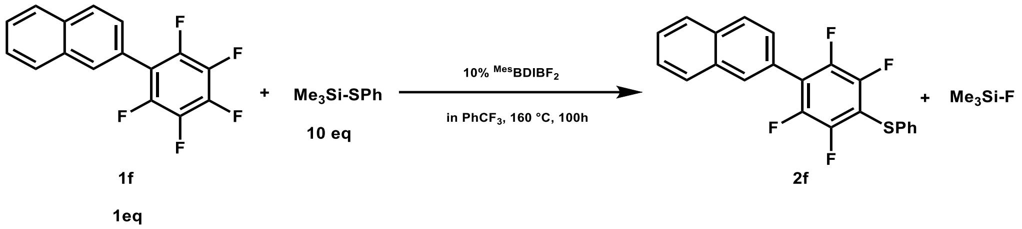

Following the general procedure, the reaction was performed with 2-(perfluorophenyl)naphthalene (73.55 mg, 0.25 mmol), Me_3Si-SPh (473.4 mg, 2.5 mmol, 10 equiv.) and $^{Mes}BDIBF_2$ (9.5 mg, 25 µmol, 10 mol%) at 160 ℃ for 100 h. Quantitative ^{19}F-NMR spectroscopy revealed that phenyl(2,3,5,6-tetrafluoro-4-(naphthalen-2-yl)phenyl)sulfane (**2f**) was formed in 95% yield. Phenyl(2,3,5,6-tetrafluoro-4-(naphthalen-2-yl)phenyl)sulfane (**2f**) (86.5 mg, 0.225mmol, 90%) was obtained as a colourless crystalline solid after purification by column chromatography on silica gel (n-pentane).

phenyl(2,3,5,6-tetrafluoro-4-(naphthalen-2-yl)phenyl)sulfane (**2f**)

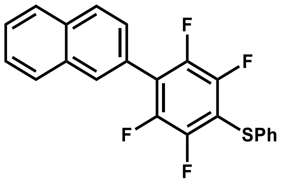

^{1}H NMR (400 MHz, C_6D_6): δ 8.04 – 7.96 (m, 2H, Ar–***H***), 7.96 – 7.90 (m, 2H, Ar–***H***), 7.65 – 7.52 (m, 3H, Ar–***H***), 7.51 – 7.44 (m, 2H, Ar–***H***), 7.40 – 7.29 (m, 3H, Ar–***H***).

^{13}C NMR (101 MHz, C_6D_6): δ 147.5 (ddm, $^1J_{C\text{–}F}$ = 245.0 Hz, $^2J_{C\text{–}F}$ = 15.0 Hz, Ar***C***), 144.2 (ddm, $^1J_{C\text{–}F}$ = 248.0 Hz, $^2J_{C\text{–}F}$ = 15.0 Hz, Ar***C***), 133.4 (s, Ar***C***), 133.3 (s, Ar***C***), 133.0 (s, Ar***C***), 130.6 (s, Ar***C***H), 130.2 (ds, Ar***C***H), 129.4 (s, Ar***C***H), 128.3 (s, Ar***C***H), 128.4 (s, Ar***C***H), 127.87 (s, Ar***C***H), 127.8 (s, Ar***C***H), 127.3 (s, Ar***C***H), 127.0 (s, Ar***C***H), 126.7 (s, Ar***C***H), 124.5 (s, Ar***C***), 121.9 (t, $^2J_{C\text{–}F}$ = 16.8 Hz, Ar***C***H), 112.7 (t, $^2J_{C\text{–}F}$ = 20.6 Hz, Ar***C***H).

^{19}F NMR (376.5 MHz, C_6D_6): δ -133.1 (dd, $^3J_{F\text{–}F}$ = 24.2 Hz, $^4J_{F\text{–}F}$ =11.9 Hz, 2F), -142.45 (dd, $^3J_{F\text{–}F}$ = 24.3 Hz, $^4J_{F\text{–}F}$ = 12.2 Hz, 2F).

^{19}F NMR (376.5 MHz, in $PhCF_3$ with a sealed glass capillary containing 1,2-difluorobenzene in C_6D_6): δ -133.4 (dd, $^3J_{F\text{–}F}$ = 24.3 Hz, $^4J_{F\text{–}F}$ = 12.2 Hz, 2F), -142.7 (dd, $^3J_{F\text{–}F}$ = 24.5 Hz, $^4J_{F\text{–}F}$ = 12.1 Hz, 2F).

HRMS (APCL) m/z: $[M + H]^+$ Calcd for $C_{22}H_{13}F_4S$ 385.0669; Found 385.0662.

- Thiodefluorination of 2,3,4,5,6-pentafluoro-4'-(trifluoromethyl)-1,1'-biphenyl

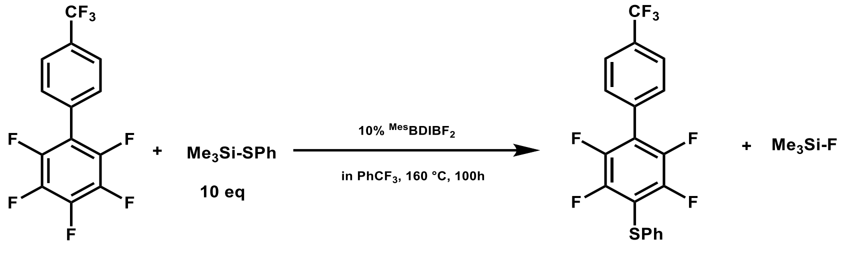

1g

1eq

2g

Following the general procedure, the reaction was performed with 2,3,4,5,6-pentafluoro-4'-(trifluoromethyl)-1,1'-biphenyl (78.0 mg, 0.25 mmol), Me_3Si-SPh (473.4 mg, 2.5 mmol, 10 equiv.) and ^{Mes}BDIBF_2 (9.5 mg, 25 µmol, 10 mol%) at 160 °C for 100 h. Quantitative ^{19}F-NMR spectroscopy revealed that phenyl(2,3,5,6-tetrafluoro-4'-(trifluoromethyl)-[1,1'-biphenyl]-4-yl)sulfane (**2g**) was formed in 95% yield. Phenyl(2,3,5,6-tetrafluoro-4'-(trifluoromethyl)-[1,1'-biphenyl]-4-yl) sulfane (**2g**) (85.4 mg, 0.212mmol, 85%) was obtained as a colourless crystalline solid after purification by column chromatography on silica gel (n-pentane).

Phenyl(2,3,5,6-tetrafluoro-4'-(trifluoromethyl)-[1,1'-biphenyl]-4-yl) sulfane (**2g**)

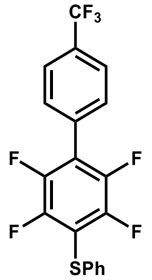

^{1}H NMR (400 MHz, C_6D_6): δ 7.80 (d, $^3J_{H\text{–}H}$ = 8.1 Hz, 2H, Ar–***H***), 7.63 (d, $^3J_{H\text{–}H}$ = 8.0 Hz, 2H, Ar–***H***), 7.47 (dd, $^3J_{H\text{–}H}$ = 7.8, 1.8, 2H, Ar–***H***), 7.40 – 7.30 (m, 3H, Ar–***H***).

^{13}C NMR (101 MHz, C_6D_6): δ 147.4 (ddm, $^1J_{C\text{–}F}$ = 247.0 Hz, $^2J_{C\text{–}F}$ = 15.0 Hz, Ar***C***), 143.8 (ddm, $^1J_{C\text{–}F}$ = 250 Hz, $^2J_{C\text{–}F}$ = 16.0 Hz, Ar***C***), 131.6 (q, $^2J_{C\text{–}F}$ = 32.0 Hz, Ar***C***) 130.9 (s, Ar***C***), 130.6 (s, Ar***C***), 129.4 (s, Ar***C***), 129.0 (s, Ar***C***), 128.1 (s, Ar***C***), 127.5 (s, Ar***C***), 125.7 (q, Hz, $^3J_{C\text{–}F}$ = 3.8 Hz), 123.8 (q, $^1J_{C\text{–}F}$ = 272.4 Hz, ***C***F_3), 120.5 – 119.4 (m, Ar***C***), 114.2 (t, $^2J_{C\text{–}F}$ = 21.0 Hz, Ar***C***).

^{19}F NMR (376.5 MHz, C_6D_6): δ -62.9 (s, 3F), -132.6 (dd, $^3J_{F\text{–}F}$ = 24.0 Hz, $^4J_{F\text{–}F}$ = 12.4 Hz, 2F), -142.6 (dd, $^3J_{F\text{–}F}$ = 24.0 Hz, $^4J_{F\text{–}F}$ = 12.4 Hz, 2F).

^{19}F NMR (376.5 MHz, in PhCF_3 with a sealed glass capillary containing 1,2-difluorobenzene in C_6D_6): δ -63.0 (s, 3F), -133.0 (dd, $^3J_{F\text{–}F}$ = 24.1 Hz, $^4J_{F\text{–}F}$ = 12.3 Hz, 2F), -142.9 (dd, $^3J_{F\text{–}F}$ = 24.0 Hz, $^4J_{F\text{–}F}$ = 12.1 Hz, 2F).

HRMS (APCL) m/z: $[M + H]^+$ Calcd for $C_{19}H_{10}F_7S$ 402.0308; Found 402.0305.

- Thiodefluorination of 2,3,4,4',5,6-hexafluoro-1,1'-biphenyl

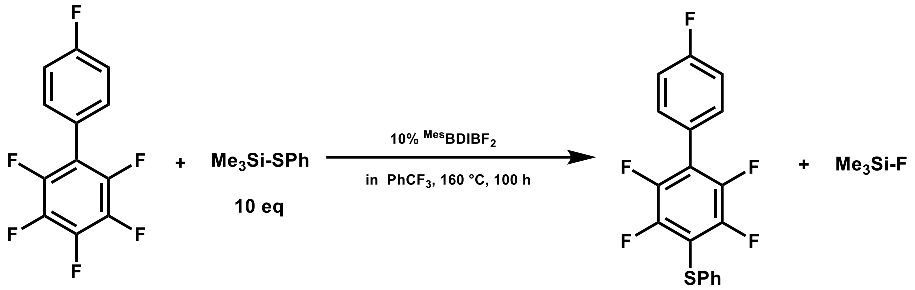

1h

1eq

2h

Following the general procedure, the reaction was performed with 2,3,4,4',5,6-hexafluoro-1,1'-biphenyl (65.6 mg, 0.25 mmol), Me_3Si-SPh (473 mg, 2.5 mmol, 10 equiv.) and $^{Mes}BDIBF_2$ (9.5 mg, 25 µmol, 10 mol%) at 160 °C for 100 h. Quantitative ^{19}F-NMR spectroscopy revealed that (2,3,4',5,6-pentafluoro-[1,1'-biphenyl]-4-yl)(phenyl)sulfane (**2h**) was formed in 92% yield. (2,3,4',5,6-pentafluoro-[1,1'-biphenyl]-4-yl)(phenyl)sulfane (**2h**) (79 mg, 0.225 mmol, 90%) was obtained as a colourless crystalline solid after purification by column chromatography on silica gel (n-pentane).

(2,3,4',5,6-pentafluoro-[1,1'-biphenyl]-4-yl)(phenyl)sulfane (**2h**)

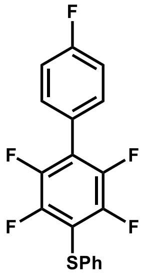

^{1}H NMR (400 MHz, C_6D_6): δ 7.54 – 7.41 (m, 4H, Ar–***H***), 7.38 – 7.30 (m, 3H, Ar–***H***), 7.26 – 7.18 (m, 2H, Ar–***H***).

^{13}C NMR (101 MHz, C_6D_6): δ 163.2 (d, $^1J_{C\text{–}F}$ = 249.0 Hz, Ar***C***), 147.4 (ddm, $^1J_{C\text{–}F}$ = 246.0 Hz, $^2J_{C\text{–}F}$ =14.0 Hz, Ar***C***), 144.0 (ddm, $^1J_{C\text{–}F}$ = 248, $^2J_{C\text{–}F}$ =15.0 Hz, Ar***C***), 133.1 (s, Ar***C***), 132.1 (d, $^3J_{C\text{–}F}$ = 8.4, Ar***C***H), 130.7 (s, Ar***C***H), 129.4 (s, Ar***C***H), 127.5 (s, Ar***C***H), 123.0 (s, Ar***C***), 120.8 (t, $^2J_{C\text{–}F}$ = 16.6 Hz, Ar***C***), 115.9 (d, $^2J_{C\text{–}F}$ = 21.9 Hz, Ar***C***H), 113.0 (t, $^2J_{C\text{–}F}$ = 20.4 Hz, Ar***C***).

^{19}F NMR (376.5 MHz, C_6D_6): δ -111.0 (p, $^3J_{H\text{–}F}$ = 6.3 Hz, 1F), -133.0 (dd, $^3J_{F\text{–}F}$ = 24.3, $^4J_{F\text{–}F}$ = 12.2 Hz, 2F), -142.8 (dd, $^3J_{F\text{–}F}$ = 24.4, $^4J_{F\text{–}F}$ = 12.1 Hz, 2F).

^{19}F NMR (376.5 MHz, in $PhCF_3$ with a sealed glass capillary containing 1,2-difluorobenzene in C_6D_6): δ -111.23 (t, $^3J_{F\text{–}H}$ = 7.9 Hz, 1F), -133.59 (dd, $^3J_{F\text{–}F}$ = 24.2, $^4J_{F\text{–}F}$ = 12.2 Hz, 2F), -143.33 (dd, $^3J_{F\text{–}F}$ = 24.1, $^4J_{F\text{–}F}$ = 11.9 Hz, 2F).

HRMS (APCL) m/z: $[M + H]^+$ Calcd for $C_{18}H_{10}F_5S$ 353.0418; Found 353.0412.

- Thiodefluorination of hexafluorobenzezne

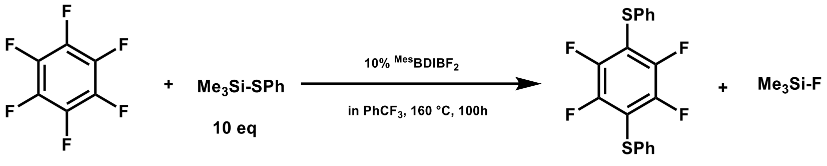

Following the general procedure, the reaction was performed with hexafluorobenzezne (47.0 mg, 0.25 mmol), Me_3Si-SPh (473.4 mg, 2.5 mmol, 10 equiv.) and $^{Mes}BDIBF_2$ (9.5 mg, 25 µmol, 10 mol%) at 160 °C for 100 h. Quantitative ^{19}F-NMR spectroscopy revealed that (perfluoro-1,4-phenylene)bis(phenylsulfane) (**2i**) was formed in 45% yield. p(perfluoro-1,4-phenylene)bis(phenylsulfane) (**2i**) (38.0 mg, 0.105 mmol, 42%) was obtained as a colourless crystalline solid after purification by column chromatography on silica gel (n-pentane).

(perfluoro-1,4-phenylene)bis(phenylsulfane) (**2i**)

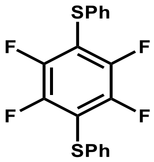

^{1}H NMR (400 MHz, C_6D_6): δ δ 7.21 (m, 4H, Ar–***H***), 6.86 (m, 6H, Ar–***H***).

^{13}C NMR (101 MHz, C_6D_6): δ 145.9 (dm, $^1J_{C\text{–}F}$ = 250 Hz, Ar***C***), 132.6 (s, Ar***C***H), 130.9 (s, Ar***C***H), 129.3 (s, Ar***C***H), 127.8 (s, Ar***C***H), 115.4 (m, Ar***C***).

^{19}F NMR (376.5 MHz, C_6D_6): δ -132.2 (s, 4F).

^{19}F NMR (376.5 MHz, in $PhCF_3$ with a sealed glass capillary containing 1,2-difluorobenzene in C_6D_6): δ -133.6 (m, 4F).

The spectral data matched with those reported in the literature.[S8]

- Thiodefluorination of tris(perfluorophenyl)phosphane

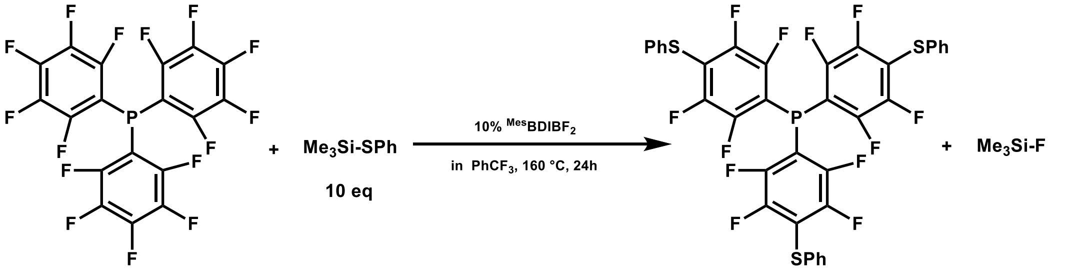

1j

1eq

2j

Following

Following the general procedure, the reaction was performed with tris(perfluorophenyl)phosphane (133 mg, 0.25 mmol), Me_3Si-SPh (473 mg, 2.5 mmol, 10 equiv.) and $^{Mes}BDIBF_2$ (9.5 mg, 25 µmol, 10 mol%) at 160 °C for 24 h. Quantitative ^{19}F-NMR spectroscopy revealed that tris(2,3,5,6-tetrafluoro-4-(phenylthio)phenyl)phosphane (**2j**) was formed in 81% yield. tris(2,3,5,6-tetrafluoro-4-(phenylthio)phenyl)phosphane (**2j**) (151 mg, 0.188 mmol, 75%) was obtained as a colourless crystalline solid after purification by column chromatography on silica gel (n-pentane： DCM = 5: 1).

tris(2,3,5,6-tetrafluoro-4-(phenylthio)phenyl)phosphane (**2j**)

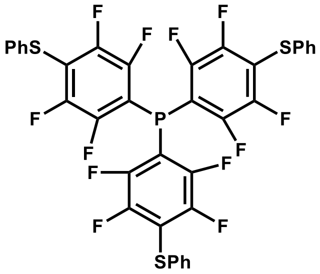

^{1}H NMR (400 MHz, C_6D_6): δ 7.46 – 7.40 (m, 6H, Ar–***H***), 7.37 – 7.31 (m, 9H, Ar–***H***).

^{13}C NMR (101 MHz, C_6D_6): δ 147.4 (dm, $^1J_{C\text{–}F}$ = 250.0 Hz, Ar***C***), 146.6 (dd, $^1J_{C\text{–}F}$ = 249.0 Hz, $^2J_{C\text{–}F}$ = 15.0 Hz, Ar***C***), 131.9 (s, Ar***C***H), 131.4 (s, Ar***C***H), 129.5 (s, Ar***C***H), 128.4 (s, Ar***C***H), 118.5 (t, $^2J_{C\text{–}F}$ = 19.8 Hz, Ar***C***), 111.0 – 109.8 (m, Ar***C***).

^{19}F NMR (376.5 MHz, C_6D_6): δ -129.7 (ddd, $^3J_{F\text{–}F}$ = 35.7 Hz, $^4J_{F\text{–}F}$ = 23.8 Hz, $^5J_{F\text{–}F}$ =12.1 Hz, 6F), -131.8 (dd, $^3J_{F\text{–}F}$ = 25.1 Hz, $^4J_{F\text{–}F}$ = 11.85 Hz, 6F).

^{19}F NMR (376.5 MHz, in $PhCF_3$ with a sealed glass capillary containing 1,2-difluorobenzene in C_6D_6): δ -130.3 (ddd, $^3J_{F\text{–}F}$ = 35.7 Hz, $^4J_{F\text{–}F}$ = 23.9 Hz, $^5J_{F\text{–}F}$ = 12.1 Hz, 6F), -132.0 (dd, $^3J_{F\text{–}F}$ = 25.1 Hz, $^4J_{F\text{–}F}$ = 11.9 Hz, 6F).

^{31}P NMR (162 MHz, $CDCl_3$): δ -72.2 (hept, $^3J_{P\text{–}F}$ = 34.4 Hz, 1P).

^{31}P NMR (162 MHz, in $PhCF_3$ with a sealed glass capillary containing 1,2-difluorobenzene in C_6D_6): δ -72.6 (hept, $^3J_{P\text{–}F}$ = 34.4 Hz, 1P).

HRMS (APCL) m/z: $[M + H]^+$ Calcd for $C_{36}H_{16}F_{12}PS_3$ 802.9955; Found 802.9943.

- Thiodefluorination of 2-(perfluorophenyl)pyridine

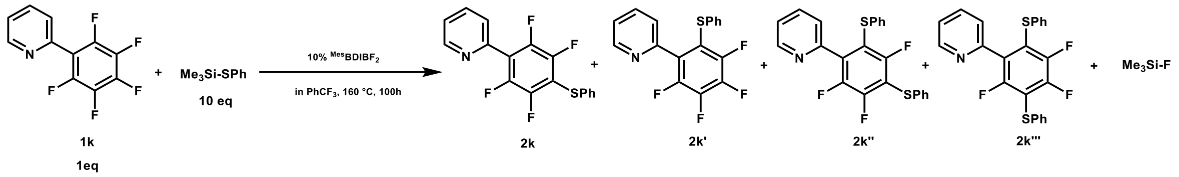

Following the general procedure, the reaction was performed with 2-(perfluorophenyl)pyridine (61.3 mg, 0.25 mmol), Me_3Si-SPh (473.4 mg, 2.5 mmol, 10 equiv.) and $^{Mes}BDIBF_2$ (9.5 mg, 25 µmol, 10 mol%) at 160 °C for 100 h. Quantitative ^{19}F-NMR spectroscopy revealed that 2-2-(2,3,5,6-tetrafluoro-4-(phenylthio)phenyl)pyridine (**2k**), 2-(2,3,4,5-tetrafluoro-6-(phenylthio)phenyl)pyridine (**2k'**), 2-(2,3,5-trifluoro-4,6-bis(phenylthio)phenyl)pyridine (**2k''**), 2-(2,4,5-trifluoro-3,6-bis(phenylthio)phenyl)pyridine (**2k'''**) were formed as shown in the table below.

2-2-(2,3,5,6-tetrafluoro-4-(phenylthio)phenyl)pyridine (**2k**) and 2-(2,3,4,5-tetrafluoro-6-(phenylthio)phenyl)pyridine (**2k'**) (25.2 mg in total, 25% (**2k**) + 5% (**2k'**)) were isolated as a mixture because these two compounds had similar polarity and were obtained as a colourless oil by column chromatography on silica gel (n-pentane: EtOAc = 10:1).

2-(2,3,5-trifluoro-4,6-bis(phenylthio)phenyl)pyridine (**2k''**) and 2-(2,4,5-trifluoro-3,6-bis(phenylthio)phenyl)pyridine (**2k'''**) (53.2 mg, 40% (**2k''**) + 10% (**2k'''**)) were isolated as a mixture because these two compounds had similar polarity and were obtained as a colourless oil after purification by column chromatography on silica gel (n-pentane: EtOAc = 5:1).

Catalyst	Mass (mg)	Temp (℃)	2k NMR yield (%)	2k' NMR yield (%)	2k'' NMR yield (%)	2k''' NMR yield (%)	2k +2k' isolated yield (%)		2k'' +2k''' isolated yield (%)	
$^{Mes}BDIBF_2$	19.0	100	33	8	45	12	25	5	40	10

2-2-(2,3,5,6-tetrafluoro-4-(phenylthio)phenyl)pyridine (**2k**)

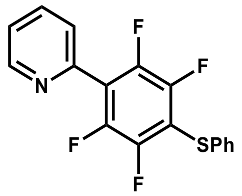

^{1}H NMR (400 MHz, C_6D_6): δ 8.86 – 8.76 (m, 1H, Py–***H***), 7.87 (td, $^{3}J_{H\text{–}H}$ = 7.7, 1.8 Hz, 1H, Py–***H***), 7.54 (dt, $^{3}J_{H\text{–}H}$ = 7.9, 1.3 Hz, 1H, Ar–***H***), 7.46 – 7.37 (m, 3H, Ar–***H***), 7.34 – 7.30 (m, 2H, Ar–***H***), 7.25 – 7.14 (m, 1H, Py–***H***).

^{19}F NMR (376.5 MHz, C_6D_6): δ -132.8 (dd, $^{3}J_{F\text{–}F}$ = 24.0 Hz, $^{4}J_{F\text{–}F}$ = 12.5 Hz, 2F), -142.8 (dd, $^{3}J_{F\text{–}F}$ = 22.9 Hz, $^{4}J_{F\text{–}F}$ = 11.2 Hz, 2F).

^{19}F NMR (376.5 MHz, in $PhCF_3$ with a sealed glass capillary containing 1,2-difluorobenzene in C_6D_6): δ -133.8 (dd, $^{3}J_{F\text{–}F}$ = 23.7 Hz, $^{4}J_{F\text{–}F}$ = 12.3 Hz, 2F), -143.2 (dd, $^{3}J_{F\text{–}F}$ = 23.2 Hz, $^{4}J_{F\text{–}F}$ = 12.6 Hz, 2F).

HRMS (APCL) m/z: $[M + H]^+$ Calcd for $C_{17}H_{10}F_4NS$ 336.0465; Found 336.0460.

2-(2,3,4,5-tetrafluoro-6-(phenylthio)phenyl)pyridine (**2k'**)

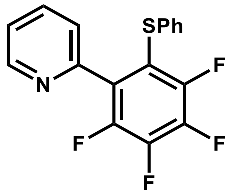

^{1}H NMR (400 MHz, C_6D_6): δ 8.74 – 8.68 (m, 1H, Py–***H***), 7.75 (td, $^{3}J_{H\text{–}H}$ = 7.7, 1.8 Hz, 1H, Py–***H***), 7.40 (d, $^{3}J_{H\text{–}H}$ = 2.2 Hz, 1H, Ar–***H***), 7.35 (t, $^{3}J_{H\text{–}H}$ = 2.4 Hz, 2H, Ar–***H***), 7.30 (s, 1H, Ar–***H***), 7.23 – 7.20 (m, 1H, Ar–***H***), 7.11 (dd, $^{3}J_{H\text{–}H}$ = 7.6, 2.1 Hz, 2H, Py–***H***).

^{19}F NMR (376.5 MHz, C_6D_6): δ -128.1 (ddd, $^{3}J_{F\text{–}F}$ = 23.4 Hz, $^{4}J_{F\text{–}F}$ = 12.14 Hz, $^{5}J_{F\text{–}F}$ = 5.2 Hz, 1F), -139.5 (dd, $^{3}J_{F\text{–}F}$ = 22.2 Hz, $^{4}J_{F\text{–}F}$ = 11.9 Hz, 1F), 153.0 (td, $^{3}J_{F\text{–}F}$ = 22.6 Hz, $^{4}J_{F\text{–}F}$ = 21.5 Hz, $^{5}J_{F\text{–}F}$ = 5.4 Hz, 1F), 153.6 (t, $^{3}J_{F\text{–}F}$ = 21.8 Hz, 1F).

^{19}F NMR (376.5 MHz, in $PhCF_3$ with a sealed glass capillary containing 1,2-difluorobenzene in C_6D_6): δ -129.2 (ddd, $^{3}J_{F\text{–}F}$ = 23.2 Hz, $^{4}J_{F\text{–}F}$ = 12.0 Hz, $^{5}J_{F\text{–}F}$ = 5.3 Hz, 1F). -140.5 (dd, $^{3}J_{F\text{–}F}$ = 22.2 Hz, $^{4}J_{F\text{–}F}$ = 12.0 Hz, 1F), 154.4 (td, $^{3}J_{F\text{–}F}$ = 22.4 Hz, $^{4}J_{F\text{–}F}$ = 21.5 Hz, $^{5}J_{F\text{–}F}$ = 5.4 Hz, 1F), 155.3 (t, $^{3}J_{F\text{–}F}$ = 21.2 Hz, 1F).

HRMS (APCL) m/z: $[M + H]^+$ Calcd for $C_{17}H_{10}F_4NS$ 336.0465; Found 336.0460.

2-(2,3,5-trifluoro-4,6-bis(phenylthio)phenyl)pyridine (**2k''**)

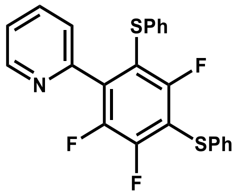

^{1}H NMR (400 MHz, C_6D_6): δ 8.70 (dd, $^{3}J_{H\text{–}H}$ = 5.5, 1.9 Hz, 1H, Py–***H***), 7.73 (td, $^{3}J_{H\text{–}H}$ = 7.7 Hz, 1.9 Hz, 1H, Py–***H***), 7.44 – 7.39 (m, 2H, Ar–***H***), 7.31 (dd, $^{3}J_{H\text{–}H}$ = 7.7, 1.8 Hz, 4H, Ar–***H***), 7.26 (d, J = 7.5 Hz, 1H, Ar–***H***), 7.19 (dd, $^{3}J_{H\text{–}H}$ = 3.5, 2.9 Hz, 4H, Ar–***H***), 7.10 – 7.04 (m, 1H, Py–***H***).

^{19}F NMR (376.5 MHz, C_6D_6): δ -110.0 (d, $^{3}J_{F\text{–}F}$ = 14.0 Hz, 1F), -124.8 (d, $^{3}J_{F\text{–}F}$ = 25.1 Hz, 1F), -130.0 (dd, $^{3}J_{F\text{–}F}$ = 25.0 Hz, $^{4}J_{F\text{–}F}$ = 14.2 Hz, 1F).

^{19}F NMR (376.5 MHz, in $PhCF_3$ with a sealed glass capillary containing 1,2-difluorobenzene in C_6D_6): δ -110.1 (d, $^{3}J_{F\text{–}F}$ = 14.7 Hz, 1F), -126.1 (d, $^{3}J_{F\text{–}F}$ = 24.4 Hz, 1F), -130.5 (dd, $^{3}J_{F\text{–}F}$ = 24.8 Hz, $^{4}J_{F\text{–}F}$ = 14.3 Hz, 1F).

2-(2,4,5-trifluoro-3,6-bis(phenylthio)phenyl)pyridine (**2k'''**)

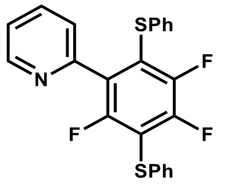

^{1}H NMR (400 MHz, C_6D_6): δ 8.73 (dd, $^{3}J_{H\text{–}H}$ = 5.4, 2.0 Hz, 1H, Py–***H***), 7.78 (td, J = 7.6, 1.9 Hz, 1H, Py–***H***), 7.46 – 7.42 (m, 2H, Ar–***H***), 7.35 (dd, $^{3}J_{H\text{–}H}$ = 7.7, 1.8 Hz, 4H, Ar–***H***), 7.30 (d, $^{3}J_{H\text{–}H}$ = 7.5 Hz, 1H, Ar–***H***), 7.25 (dd, $^{3}J_{H\text{–}H}$ = 3.5, 2.9 Hz, 4H, Ar–***H***), 7.08 (m, 1H, Py–***H***).

^{19}F NMR (376.5 MHz, C_6D_6): δ -97.2 (d, $^{3}J_{F\text{–}F}$ = 14.6 Hz, 1F), -123.7 (d, $^{3}J_{F\text{–}F}$ = 24.3 Hz, 1F), -139.9 (dd, $^{3}J_{F\text{–}F}$ = 24.0 Hz, $^{4}J_{F\text{–}F}$ =14.8 Hz, 1F).

^{19}F NMR (376.5 MHz, in $PhCF_3$ with a sealed glass capillary containing 1,2-difluorobenzene in C_6D_6): δ -96.91 (d, $^{3}J_{F\text{–}F}$ = 14.9 Hz, 1F), -124.93 (d, $^{3}J_{F\text{–}F}$ = 24.2 Hz,1F), -140.71 (dd, $^{3}J_{F\text{–}F}$ = 24.0 Hz, $^{4}J_{F\text{–}F}$ = 14.6 Hz,1F).

HRMS (APCL) m/z: $[M + H]^+$ Calcd for $C_{23}H_{15}F_3NS_2$ 426.0593; Found 426.0587.

- Thiodefluorination of 3',4',5',6'-tetrafluoro-4,4''-bis(trifluoromethyl)-1,1':2',1''-terphenyl

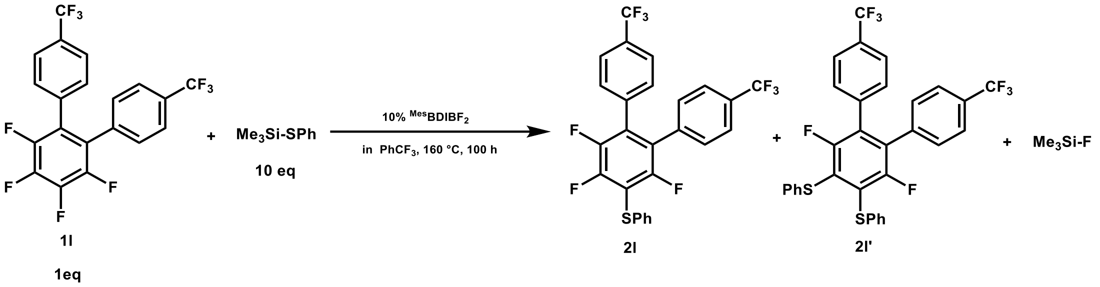

Following the general procedure, the reaction was performed with 3',4',5',6'-tetrafluoro-4,4''-bis(trifluoromethyl)-1,1':2',1''-terphenyl (**1l**, 109.6 mg, 0.25 mmol), Me_3Si-SPh (473.4 mg, 2.5 mmol, 10 equiv.) and $^{Mes}BDIBF_2$ (9.5 mg, 25 µmol, 10 mol%) at 160 °C for 100 h. Quantitative ^{19}F-NMR spectroscopy revealed that phenyl(3',5',6'-trifluoro-4,4''-bis(trifluoromethyl)-[1,1':2',1''-terphenyl]-4'-yl)sulfane (**2l**) and (3',6'-difluoro-4,4''-bis(trifluoromethyl)-[1,1':2',1''-terphenyl]-4',5'-diyl)bis(phenylsulfane) (**2l'**) were formed as shown in the table below.

Phenyl(3',5',6'-trifluoro-4,4''-bis(trifluoromethyl)-[1,1':2',1''-terphenyl]-4'-yl)sulfane (**2l**, 59.1 mg, 0.113 mmol, 45%) was obtained as a colourless crystalline oil after purification by column chromatography on silica gel (n-pentane: DCM = 10:1). (3',6'-difluoro-4,4''-bis(trifluoromethyl)-[1,1':2',1''-terphenyl]-4',5'-diyl)bis(phenylsulfane) (**2l'**, 59.0 mg, 0.09 mmol, 35%) was obtained as a colourless oil after purification by column chromatography on silica gel (n-pentane).

Catalyst	Mass (mg)	Temp (℃)	2l NMR yield (%)	2l' NMR yield (%)	2l isolated yield (%)	2l' isolated yield (%)
$^{Mes}BDIBF_2$	19.0	160	50	40	45	35

phenyl(3',5',6'-trifluoro-4,4''-bis(trifluoromethyl)-[1,1':2',1''-terphenyl]-4'-yl)sulfane (**2l**)

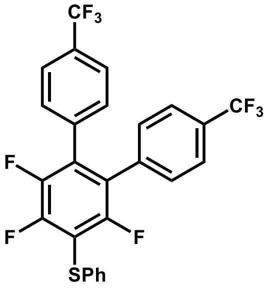

^{1}H NMR (400 MHz, C_6D_6): δ 7.58 – 7.45 (m, 6H, Ar–***H***), 7.38 – 7.30 (m, 3H, Ar–***H***), 7.20 (m, 4H, Ar–***H***).

^{13}C NMR (101 MHz, C_6D_6): δ 155.3 (d, $^1J_{C-F}$ = 246.6 Hz, Ar***C***), 150.8 (dd, $^1J_{C-F}$ = 251.6 Hz, $^2J_{C-F}$ = 20.1 Hz, Ar***C***), 150.6 (dd, $^1J_{C-F}$ = 251.6 Hz, $^2J_{C-F}$ =20.1 Hz, Ar***C***), 144.8 (dd, $^1J_{C-F}$ = 246.6 Hz, Ar***C***), 144.6 (dd, $^1J_{C-F}$ = 246.6 Hz, Ar***C***), 135.5 (s, Ar***C***), 135.1 (s, Ar***C***), 133.3 (s, Ar***C***), 131.1 (s, Ar***C***H), 130.9 (s, Ar***C***H), 130.7 (s, Ar***C***H), 130.3 (d, $^2J_{C-F}$ = 29.4 Hz, Ar***C***), 129.3 (s, Ar***C***H), 127.8 (s, Ar***C***H), 125.2 (q, $^3J_{C-F}$ = 8.0 Hz, Ar***C***H), 125.1 (q, $^3J_{C-F}$ = 8.0 Hz, Ar***C***H), 123.8 (d, $^2J_{C-F}$ = 18.8 Hz, Ar***C***), 123.7 (q, $^1J_{C-F}$ = 270 Hz, Ar***C***F_3), 123.6 (q, $^1J_{C-F}$ = 268 Hz, Ar***C***F_3), 113.3 (m, Ar***C***),

^{19}F NMR (376.5 MHz, C_6D_6): δ -62.7 (s, 3F), -62.8 (s, 3F), -109.4 (d, $^3J_{F-F}$ = 14.8 Hz, 1F), -125.5 (d,

$^{3}J_{F\text{–}F}$ = 24.2 Hz, 1F), -141.1 (dd, $^{3}J_{F\text{–}F}$ = 24.3 Hz, $^{4}J_{F\text{–}F}$ = 14.8 Hz, 1F).

^{19}F NMR (376.5 MHz, in $PhCF_3$ with a sealed glass capillary containing 1,2–difluorobenzene in C_6D_6): δ -63.1 (d, $^{3}J_{F\text{–}F}$ = 17.0 Hz, 6F), -109.5 (d, $^{3}J_{F\text{–}F}$ = 15.1 Hz, 1F), -126.2 (d, $^{3}J_{F\text{–}F}$ = 24.2 Hz, 1F), -141.6 (dd, $^{3}J_{F\text{–}F}$ = 14.8 Hz, $^{4}J_{F\text{–}F}$ = 24.3 Hz, 1F).

HRMS (APCL) m/z: $[M + H]^+$ Calcd for $C_{26}H_{14}F_9S$ 529.0667; Found 529.0662.

(2,3,4',5,6-pentafluoro-[1,1'-biphenyl]-4-yl)(phenyl)sulfane (**2l’**)

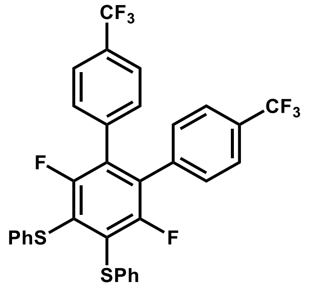

^{1}H NMR (400 MHz, C_6D_6): δ 7.52 (d, $^{3}J_{H\text{–}H}$ = 8.1 Hz, 4H, Ar–***H***), 7.42 – 7.35 (m, 4H, Ar–***H***), 7.35 – 7.29 (m, 4H, Ar–***H***), 7.28 – 7.23 (m, 2H, Ar–***H***), 7.21 (d, $^{3}J_{H\text{–}H}$ = 8.1 Hz, 4H, Ar–***H***).

^{13}C NMR (101 MHz, C_6D_6): δ 156.3 (dd, $^{1}J_{C\text{–}F}$ = 247, 4.3 Hz, Ar***C***), 135.9 (s, Ar***C***), 135.0 (s, Ar***C***), 131.0 (s, Ar***C***H), 130.3 (q, $^{2}J_{C\text{–}F}$ = 33.0 Hz, Ar***C***), 129.8 (s, Ar***C***H), 129.5 (m, Ar***C***), 129.2 (s, Ar***C***H), 128.4 (m, Ar***C***), 127.2 (s, Ar***C***H), 125.1 (q, $^{3}J_{C\text{–}F}$ = 4.0 Hz, Ar***C***H), 123.8 (q, $^{1}J_{C\text{–}F}$ = 270 Hz, Ar***C***F_3).

^{19}F NMR (376.5 MHz, C_6D_6): δ -62.8 (s, 6F), -105.0 (s, 2F).

^{19}F NMR (376.5 MHz, in $PhCF_3$ with a sealed glass capillary containing 1,2-difluorobenzene in C_6D_6): δ -63.0 (s, 6F), -105.0 (s, 2F).

HRMS (APCL) m/z: $[M + H]^+$ Calcd for $C_{32}H_{19}F_8S_2$ 619.0783; Found 619.0795.

- Thiodefluorination of perfluoroprop-1-ene

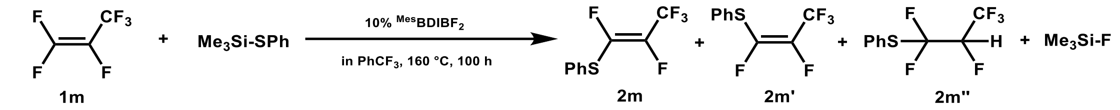

1atm, about 1eq

Following the general procedure, the reaction was performed with perfluoroprop-1-ene (1 atm. in J. Young NMR tube, approx. 0.1 mmol, 14.9 mg), Me_3Si-SPh (41.2 mg, 0.5 mmol) and $^{Mes}BDIBF_2$ (1.9 mg, 5 µmol, 10 mol%) at 160 °C for 100 h. Quantitative ^{19}F-NMR spectroscopy revealed that (Z)-(perfluoroprop-1-en-1-yl)(phenyl)sulfane (**2m**) and (E)-(perfluoroprop-1-en-1-yl)(phenyl)sulfane (**2m'**) and (1,1,2,3,3,3-hexafluoropropyl)(phenyl)sulfane (**2m''**) were formed as shown in the table below.

Catalyst	Mass (mg)	Temp (°C)	2m NMR yield (%)	2m' NMR yield (%)	2m'' NMR yield (%)
$^{Mes}BDIBF_2$	19.0	160	18	45	36

(Z)-(perfluoroprop-1-en-1-yl)(phenyl)sulfane (**2m**)

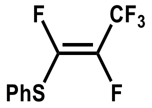

^{19}F NMR (376.5 MHz, in $PhCF_3$ with a sealed glass capillary containing 1,2-difluorobenzene in C_6D_6): δ -67.9 (dd, $^3J_{F\text{–}F}$ = 21.4, 11.7 Hz, 3F), -120.2 (dq, $^3J_{F\text{–}F}$ = 146.8, 21.0 Hz, 1F), -156.2 (dq, $^3J_{F\text{–}F}$ = 146.5, 11.7 Hz, 1F).

(E)-(perfluoroprop-1-en-1-yl)(phenyl)sulfane (**2m'**)

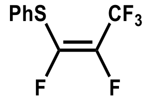

^{19}F NMR (376.5 MHz, in $PhCF_3$ with a sealed glass capillary containing 1,2-difluorobenzene in C_6D_6): δ -64.8 (dd, $^3J_{F\text{–}F}$ = 21.0, 10.5 Hz, 3F), -99.8 (dd, $^3J_{F\text{–}F}$ = 11.5, 11.0 Hz, 1F), -136.5 (q, $^3J_{F\text{–}F}$ = 12.4, 11.7 Hz, 1F).

(1,1,2,3,3,3-hexafluoropropyl)(phenyl)sulfane (**2m''**)

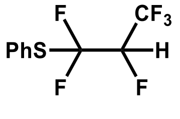

^{19}F NMR (376.5 MHz, in $PhCF_3$ with a sealed glass capillary containing 1,2-difluorobenzene in C_6D_6): δ -74.0 – -74.2 (m, 3F), -81.8 – -82.7 (m, 1F), -88.4 – -89.3 (m, 1F), -204.4 (qd, $^3J_{F\text{–}F}$ = 49.2, 11.1 Hz, 1F).

The spectral data matched with those reported in the literature. [S9]

3.3. General Procedure for Hydrodefluorination

In a glovebox, the fluoroarene (0.05 mmol), silane reagent (0.15 – 0.5 mmol), catalyst mixture (10-20 mol % $^{Mes}BDIMF_2$ + 10-20 mol % $NaBArF_{24}$) and $PhCF_3$ or PhF (dry and degassed) were added to a J-Young's NMR tube and sealed. The total volume of the $PhCF_3$ and C_6D_6 (5:1) or PhF and C_6D_6 (5:1) solution was 0.6 ml. After reaction at 25 °C or 160 °C, the HDF product was purified by column chromatography. If the HDF product was volatile, 1,2-difluorobenzene in a sealed glass capillary containing C_6D_6 (δ = 138.3 ppm) was added as an internal standard and the reaction mixture was analyzed by quantitative ^{19}F-NMR spectroscopy.

- **Control experiment:**

A series of control experiments were conducted to ensure the internal standards were stable under the reaction conditions.

A sealed glass capillary containing C_6D_6 was placed in a J. Young NMR tube. PhF (0.6ml) or $PhCF_3$ (0.6ml), and triethylsilane (80.0 µL, 0.50 mmol) were added to the J. Young NMR tube. The reaction mixture was stored at 160 °C for 48 h. However, the HDF products were not observed by ^1H or ^{19}F NMR spectroscopy.

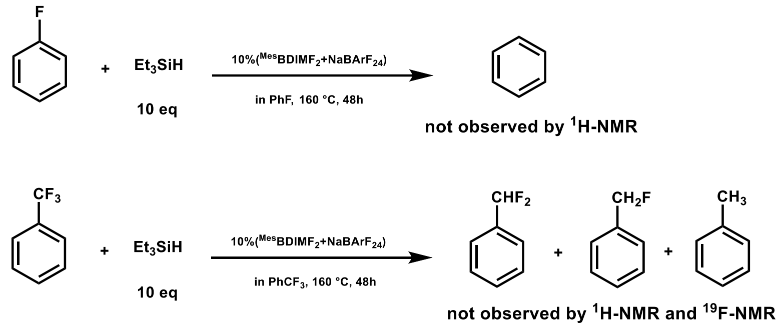

3.3.1. Results and Characterisation Data of HDF Products

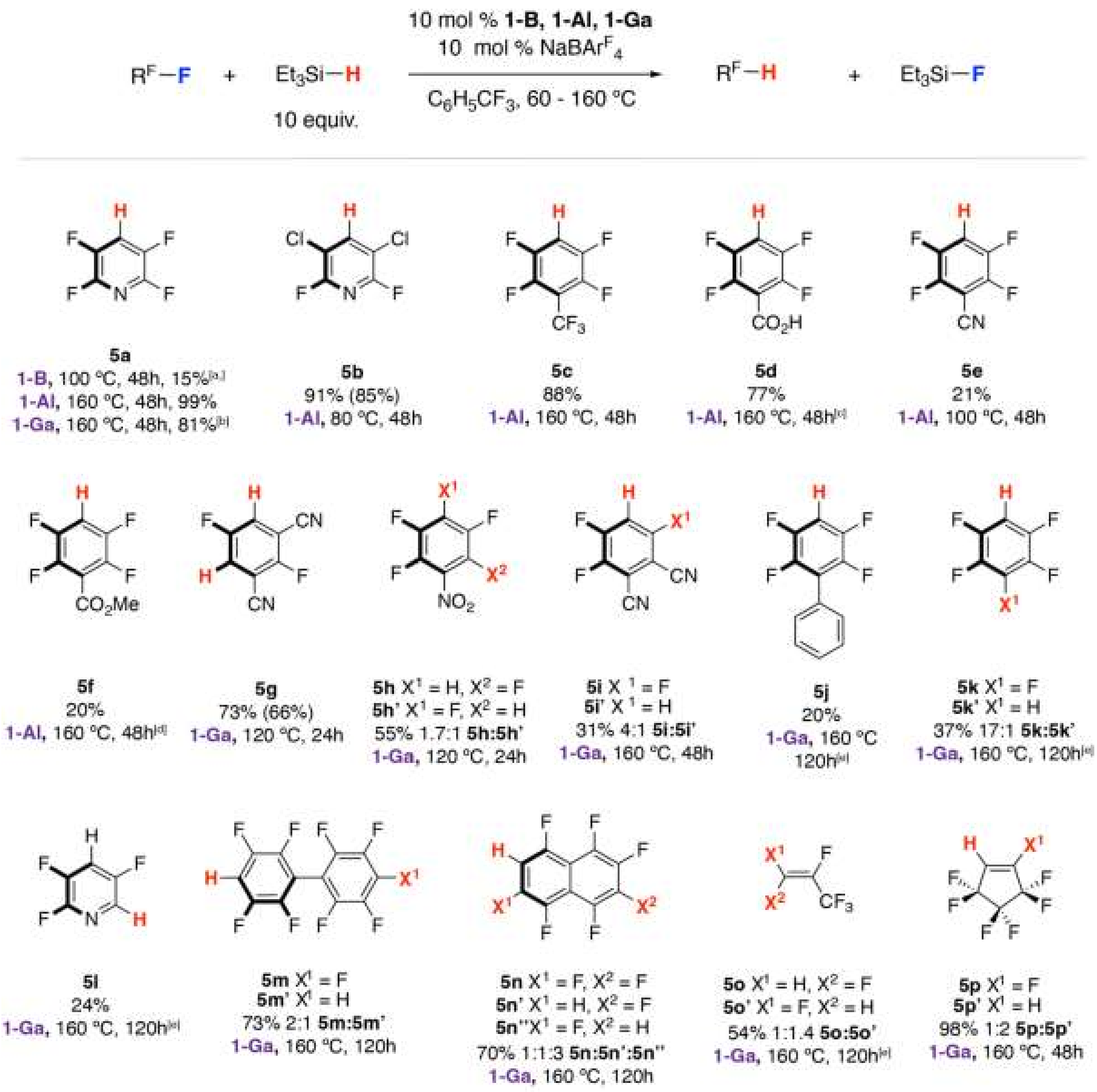

Figure S2. Scope of group 13 fluoride catalysed hydrodefluorination of fluorinated arenes and alkenes. [a] NMR yields measured by ^{19}F NMR spectroscopy using 1,2-difluorobenzene as an internal standard. Isolated yields in parentheses. [b] Product formed alongside 12% **5k**. [c] Me_2PhSiH used as terminal reductant. [d] Ph_3SiH used as terminal reductant. [e] 20 mol% catalyst loading used.

- HDF of pentafluoropyridine

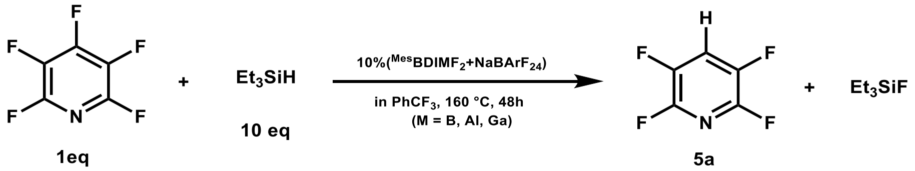

Following the general procedure, the reaction was performed with pentafluoropyridine (17.0 mg, 0.10 mmol), Et_3SiH (116.8 mg, 1.0 mmol, 10.0 equiv.), $^{Mes}BDIMF_2$ (10.0 µmol, 10 mol%) and $NaBArF_{24}$ (8.9 mg, 10.0 µmol, 10 mol%) at 160 °C for 48 h. Quantitative ^{19}F-NMR revealed that 2,3,5,6-tetrafluoropyridine (**5a**) was formed as shown in the table below.

Catalyst	Mass (mg)	Temp (°C)	5a yield (%)
$^{Mes}BDIBF_2$	3.8	160	15
$^{Mes}BDIAlF_2$	4.0	160	99
$^{Mes}BDIGaF_2$	4.5	160	81

2,3,5,6-Tetrafluoropyridine (**5a**)

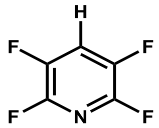

^{19}F NMR (376.5 MHz, in PhF with a sealed glass capillary containing 1,2-difluorobenzene in C_6D_6): δ -91.7 – -92.0 (m, 2F, o-F), -140.5 – -140.7 (m, 2F, m-F).

The spectral data matched with those reported in the literature.[S10]

● HDF of 3,5-dichloro-2,4,6-trifluoropyridine

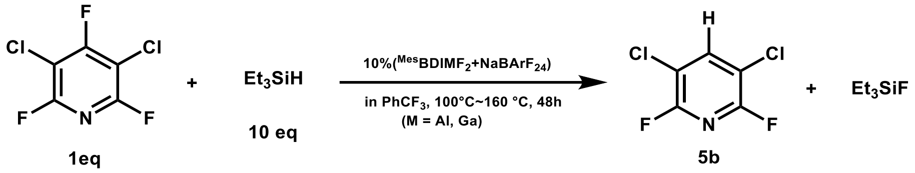

Following the general procedure, the reaction was performed with 3,5-dichloro-2,4,6-trifluoropyridine (101.0 mg, 0.5 mmol), Et_3SiH (584.0 mg, 5.0 mmol, 10.0 equiv.), $^{Mes}BDIMF_2$ (50.0 µmol, 10 mol%) and $NaBArF_{24}$ (44.5 mg, 50.0 µmol, 10 mol%) at 100 – 160 °C for 24 h. Quantitative ^{19}F-NMR spectroscopy revealed that 3,5-dichloro-2,6-difluoropyridine (**5b**) was formed as shown in the table below. 3,5-dichloro-2,6-difluoropyridine) (**5b**) was obtained as a colourless crystalline solid after purification by column chromatography on silica gel (n-pentane).

Catalyst	Mass (mg)	Temp (°C)	5b NMR yield (%)	5b isolated yield (%)
$^{Mes}BDIAlF_2$	20.0	100	91	85
$^{Mes}BDIGaF_2$	27.0	160	92	86

3,5-dichloro-2,6-difluoropyridine (**5b**)

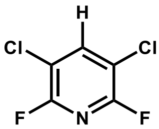

^{1}H NMR (400 MHz, $CDCl_3$): δ 7.97 (t, $^3J_{H-H}$ = 7.5 Hz, 1H, Ar–***H***).

^{13}C NMR (101 MHz, $CDCl_3$): δ 154.6 (dd, $^1J_{C-F}$ = 247.7 Hz, $^2J_{C-F}$ =13.0 Hz, Ar***C***H), 143.8 (s, Ar***C***H), 114.3 – 113.3 (m, Ar***C***H).

^{19}F NMR (376.5 MHz, $CDCl_3$): δ -72.2 (s, 1F).

^{19}F NMR (376.5 MHz, in $PhCF_3$ with a sealed glass capillary containing 1,2-difluorobenzene in C_6D_6): δ -73.4 (s, 1F)

The spectral data matched with those reported in the literature. [S10]

- HDF of octafluorotoluene

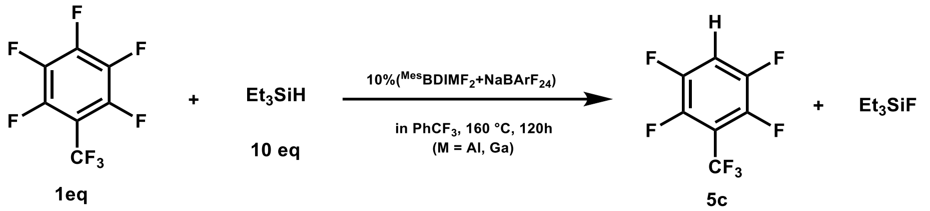

Following the general procedure, the reaction was performed with octafluorotoluene (24.0 mg, 0.10 mmol), Et_3SiH (116.8 mg, 1.0 mmol, 10.0 equiv.), $^{Mes}BDIMF_2$ (10.0 µmol, 10 mol%) and $NaBArF_{24}$ (8.9 mg, 10.0 µmol, 10 mol%) at 160 °C for 120 h. Quantitative ^{19}F-NMR spectroscopy revealed that 1,2,4,5-tetrafluoro-3-trifluoromethylbenzene (**5c**) was formed as shown in the table below.

Catalyst	Mass (mg)	Temp (°C)	5c yield (%)
$^{Mes}BDIAlF_2$	4.0	160	88
$^{Mes}BDIGaF_2$	4.5	160	90

1,2,4,5-Tetrafluoro-3-(trifluoromethyl)benzene (**5c**)

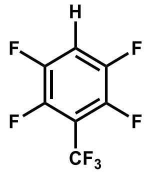

^{19}F NMR (376.5 MHz, in $PhCF_3$ with a sealed glass capillary containing 1,2-difluorobenzene in C_6D_6): δ -56.9 – -57.1 (m, 3F), -137.1 – -137.3 (m, 2F), -141.1 – -141.4 (m, 2F).

The spectral data matched with those reported in the literature.[S11]

- HDF of 2,3,4,5,6-pentafluorobenzoic acid

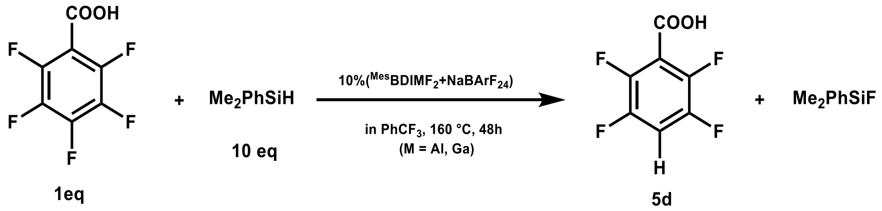

Following the general procedure, the reaction was performed with 2,3,4,5,6-pentafluorobenzoic acid (21.2 mg, 0.10 mmol), Me_2SiPhH (136.1 mg, 1.0 mmol, 10.0 equiv.), $^{Mes}BDIMF_2$ (10.0 µmol, 10 mol%) and $NaBArF_{24}$ (8.9 mg, 10.0 µmol, 10 mol%) at 160 °C for 48 h. Quantitative ^{19}F-NMR spectroscopy revealed that 2,3,5,6-tetrafluorobenzoic acid (**5d**) was formed as shown in the table below.

Catalyst	Mass (mg)	Temp (℃)	5d NMR yield (%)
$^{Mes}BDIAlF_2$	4.0	160	77
$^{Mes}BDIGaF_2$	4.5	160	80

2,3,5,6-tetrafluorobenzoic acid (**5d**)

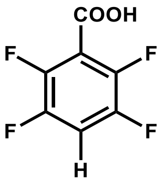

^{19}F NMR (376.5 MHz, in PhF with a sealed glass capillary containing 1,2-difluorobenzene in C_6D_6): δ -140.1 – -140.3 (m, 2F, o-F), -144.9 – -145.1 (m, 2F, m-F).

IR (cm^{-1}): 2957.45 (O-H str), 1767.50 (C=O str), 1275.11 (C-O str).

The spectral data matched with those reported in the literature.[S12]

- HDF of 2,3,4,5,6-pentafluorobenzonitrile

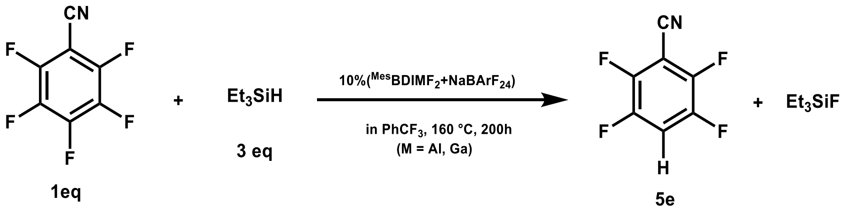

Following the general procedure, the reaction was performed with 2,3,4,5,6-pentafluorobenzonitrile (19.4 mg, 0.10 mmol), Et_3SiH (35.1 mg, 0.3 mmol, 3.0 equiv.) $^{Mes}BDIMF_2$ (10.0 µmol, 10 mol%) and $NaBArF_{24}$ (8.9 mg, 10.0 µmol, 10 mol%) at 100– 120 °C for 48 h. Quantitative ^{19}F-NMR spectroscopy revealed that 2,3,5,6-tetrafluorobenzonitrile (**5e**) was formed as shown in the table below.

**Catalyst**	**Mass (mg)**	**Temp (°C)**	**5e yield (%)**
$^{Mes}BDIAlF_2$	4.0	100	21
$^{Mes}BDIGaF_2$	4.5	120	24

2,3,5,6-tetrafluorobenzonitrile (**5e**)

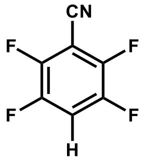

^{19}F NMR (376.5 MHz, in PhF with a sealed glass capillary containing 1,2-difluorobenzene in C_6D_6): δ -139.8– -140.0 (m, 2F, m-F), -146.1 – -146.3 (m, 2F, o-F).

The spectral data matched with those reported in the literature.[S10]

- HDF of methyl 2,3,4,5,6-pentafluorobenzoate

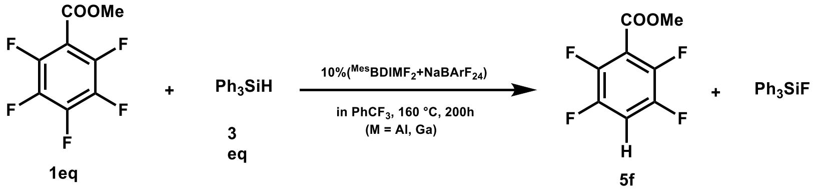

Following the general procedure, the reaction was performed with 2,3,4,5,6-pentafluorobenzoate (22.6 mg, 0.10 mmol), Ph_3SiH (260.1 mg, 1.0 mmol, 10.0 equiv.), $^{Mes}BDIMF_2$ (10.0 µmol, 10 mol%) and $NaBArF_{24}$ (8.9 mg, 10.0 µmol, 10 mol%) at 160 °C for 48 h. Quantitative ^{19}F-NMR spectroscopy revealed that methyl 2,3,5,6-tetrafluorobenzoate (**5f**) was formed as shown in the table below.

Catalyst	Mass (mg)	Temp (°C)	5f NMR yield (%)
$^{Mes}BDIAlF_2$	4.0	160	20
$^{Mes}BDIGaF_2$	4.5	160	25

2,3,5,6-tetrafluorobenzoate (**5f**)

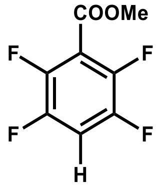

^{19}F NMR (376.5 MHz, in PhF with a sealed glass capillary containing 1,2–difluorobenzene in C_6D_6): δ -137.7 – -137.9 (m, 2F, o-F), -140.2 – -140.6 (m, 2F, m-F).

IR (cm^{-1}): 1736.73 (C=O str), 1271.69 (C-O str).

The spectral data matched with those reported in the literature.[S13]

● HDF of 2,4,5,6-tetrafluoroisophthalonitrile

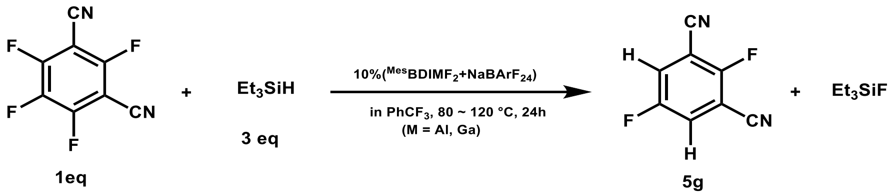

Following the general procedure, the reaction was performed with 2,4,5,6-tetrafluoroisophthalonitrile (100.0 mg, 0.5 mmol), Et_3SiH (174.0 mg, 1.5 mmol,3.0 equiv.), $^{Mes}BDIMF_2$ (50.0 µmol, 10 mol%) and $NaBArF_{24}$ (44.5 mg, 50.0 µmol, 10 mol%) at 80°C – 120 °C for 24 h. Quantitative ^{19}F-NMR spectroscopy revealed that 2,5-difluoroisophthalonitrile (**5g**) was formed as shown in the table below. 2,5-difluoroisophthalonitrile (**5g**) was obtained as a colourless crystalline solid after purification by column chromatography on silica gel on silica gel (n-pentane/DCM = 5:1, v/v).

Catalyst	Mass (mg)	Temp (°C)	5g NMR yield (%)	5g isolated yield (%)
$^{Mes}BDIAlF_2$	20.0	80	71	62
$^{Mes}BDIGaF_2$	27.0	120	73	66

2,5-difluoroisophthalonitrile (**5g**)

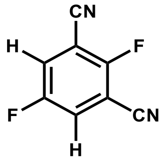

^{1}H NMR (400 MHz, $CDCl_3$): δ 7.65 (dd, $^{3}J_{H–F}$ = 6.8 Hz, $^{3}J_{H–H}$ = 4.7 Hz, 2H).

^{13}C NMR (101 MHz, $CDCl_3$): δ 161.3 (dd, $^{1}J_{C–F}$ = 266.0 Hz, $^{3}J_{C–F}$ = 3.0 Hz, Ar***C***), 156.1 (dd, $^{1}J_{C–F}$ = 253.0 Hz, $^{3}J_{C–F}$ = 4.0 Hz, Ar***C***), 125.0 (d, $^{2}J_{C–F}$ = 26.5 Hz, Ar***C***H), 110.9 (d, $^{3}J_{C–F}$ = 2.5 Hz, ***C***N), 104.6 (dd, $^{2}J_{C–F}$ = 16.8 Hz, $^{3}J_{C–F}$ = 9.8 Hz, Ar***C***).

^{19}F NMR (376.5 MHz, $CDCl_3$): δ -105.2 (d, $^{3}J_{F–H}$ = 16.4 Hz, 1F), -111.3 (d, $^{3}J_{F–H}$ = 16.4 Hz, 1F).

^{19}F NMR (376.5 MHz, in PhF with a sealed glass capillary containing 1,2-difluorobenzene in C_6D_6): δ –107.0 (d, $^{3}J_{F–H}$ = 16.0 Hz, 1F), –111.8 (d, $^{3}J_{F–H}$ = 16.0 Hz, 1F).

IR (cm^{-1}): 2239 (CN str).

The spectral data matched with those reported in the literature.[S10]

- HDF of 1,2,3,4,5-pentafluoro-6-nitrobenzene

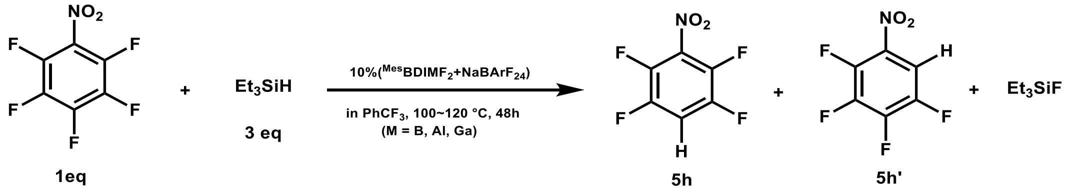

Following the general procedure, the reaction was performed with 1,2,3,4,5-pentafluoro-6-nitrobenzene (21.2 mg, 0.1 mmol), Et_3SiH (35.1 mg, 0.3 mmol, 3.0 equiv.), $^{Mes}BDIMF_2$ (10.0 μmol, 10 mol%) and $NaBArF_{24}$ (8.9 mg, 10.0 μmol, 10 mol%) at 100 – 120 °C for 48 h. Quantitative ^{19}F-NMR spectroscopy revealed that 1,2,4,5-tetrafluoro-3-nitrobenzene (**5h**) and 1,2,3,4-tetrafluoro-5-nitrobenzene (**5h’**) were formed as shown in the table below.

Catalyst	Mass (mg)	Temp (°C)	5h yield (%)	5h’ yield (%)
$^{Mes}BDIBF_2$	3.8	100	25	10
$^{Mes}BDIAlF_2$	4.0	100	30	14
$^{Mes}BDIGaF_2$	4.5	120	35	20

1,2,4,5-tetrafluoro-3-nitrobenzene (**5h**)

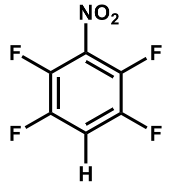

^{19}F NMR (376.5 MHz, in PhF with a sealed glass capillary containing 1,2-difluorobenzene in C_6D_6): δ -135.7 – -135.9 (m, 2F, o-F), -147.6 – -147.8 (m, 2F, m-F).

1,2,3,4-tetrafluoro-5-nitrobenzene (**5h’**)

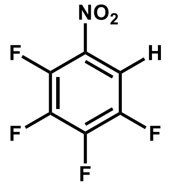

^{19}F NMR (376.5 MHz, in PhF with a sealed glass capillary containing 1,2-difluorobenzene in C_6D_6): δ -136.6 – -136.8 (m, 1F), -142.1 – -142.3 (m, 1F), -145.4 – -145.6 (m, 1F), -151.4 – -151.6 (m, 1F).

The spectral data matched with those reported in the literature.[S13]

- HDF of 3,4,5,6-tetrafluorophthalonitrile

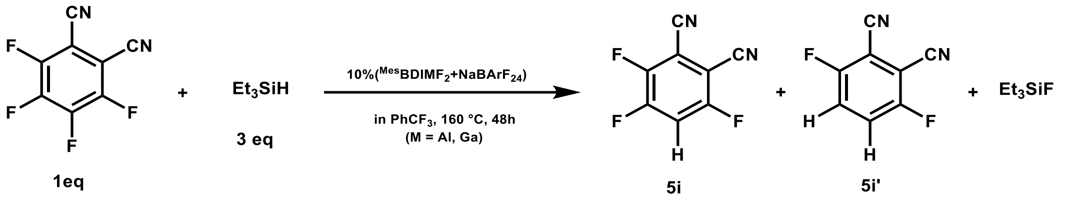

Following the general procedure, the reaction was performed with 3,4,5,6-tetrafluorophthalonitrile (20.0 mg, 0.10 mmol), Et_3SiH (35.1 mg, 0.3 mmol, 3.0 equiv.) $^{Mes}BDIMF_2$ (10.0 µmol, 10 mol%) and $NaBArF_{24}$ (8.9 mg, 10.0 µmol, 10 mol%) at 160 °C for 48 h. Quantitative ^{19}F-NMR spectroscopy revealed that 3,4,6-trifluorophthalonitrile (**5i**) and 3,6-difluorophthalonitrile (**5i'**) were formed as shown in the table below.

Catalyst	Mass (mg)	Temp (°C)	5i NMR yield (%)	5i' NMR yield (%)
$^{Mes}BDIAlF_2$	4.0	160	20	4
$^{Mes}BDIGaF_2$	4.5	160	25	6

3,4,6-trifluorophthalonitrile (**5i**)

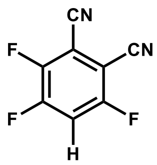

^{19}F NMR (376.5 MHz, in PhF with a sealed glass capillary containing 1,2-difluorobenzene in C_6D_6): δ -103.6 (d, $^3J_{F\text{–}H}$ = 11.0 Hz, 1F), -119.3 (d, $^3J_{F\text{–}H}$ = 19.7 Hz, 1F), -130.8 (ddd, $^3J_{F\text{–}H}$ = 20.1 HZz, $^3J_{F\text{–}F}$ = 12.7 Hz, $^4J_{F\text{–}F}$ = 6.5 Hz, 1F).

3,6-difluorophthalonitrile (**5i'**)

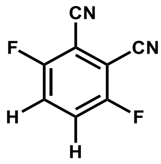

^{19}F NMR (376.5 MHz, in PhF with a sealed glass capillary containing 1,2-difluorobenzene in C_6D_6): δ -108.1 – -108.3 (m, 2F).

IR (cm^{-1}): 2238 (CN str).

The spectral data matched with those reported in the literature.[S11]

- HDF of 2,3,4,5,6-pentafluoro-1,1'-biphenyl

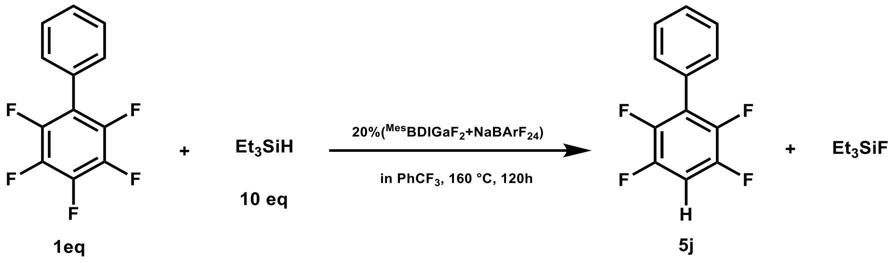

Following the general procedure, the reaction was performed with 2,3,4,5,6-pentafluoro-1,1'-biphenyl (24.4 mg, 0.10 mmol), Et_3SiH (116.8 mg, 1.0 mmol, 10.0 equiv.), $^{Mes}BDIGaF_2$ (9.0 mg, 20.0 µmol, 20 mol%) and $NaBArF_{24}$ (17.8 mg, 20.0 µmol, 20 mol%) at 160 °C for 120 h. Quantitative ^{19}F-NMR spectroscopy revealed that 2,3,5,6-tetrafluoro-1,1'-biphenyl (**5j**) was formed as shown in the table below.

Catalyst	Mass (mg)	Temp (°C)	5j NMR yield (%)
$^{Mes}BDIGaF_2$	9.0	160	20

2,3,5,6-tetrafluoro-1,1'-biphenyl (**5j**)

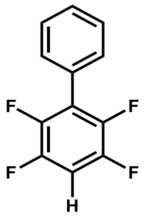

^{19}F NMR (376.5 MHz, in $PhCF_3$ with a sealed glass capillary containing 1,2-difluorobenzene in C_6D_6): δ -139.7– -140.2 (m, 2F), -144.5 – -144.7 (m, 2F).

The spectral data matched with those reported in the literature.[S10,S15]

- HDF of hexafluorobenzezne

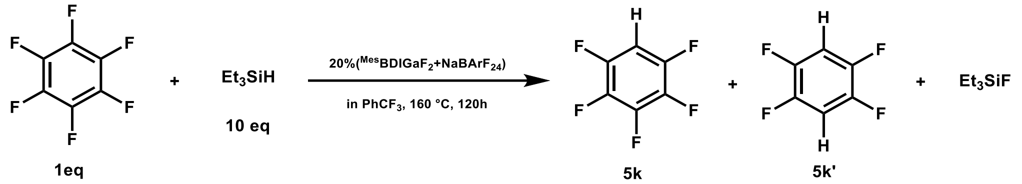

Following the general procedure, the reaction was performed with hexafluorobenzezne (18.6 mg, 0.10 mmol), Et_3SiH (116.8 mg, 1.0 mmol, 10.0 equiv.), $^{Mes}BDIGaF_2$ (9.0 mg, 20.0 µmol, 20 mol%) and $NaBArF_{24}$ (17.8 mg, 20.0 µmol, 20 mol%) at 160 °C for 120 h. Quantitative ^{19}F-NMR spectroscopy revealed that pentafluorobenzene (**5k**), 1,2,4,5-tetrafluorobenzene (**5k'**) were formed as shown in the table below.

Catalyst	Mass (mg)	Temp (°C)	5k NMR yield (%)	5k' NMR yield (%)
$^{Mes}BDIGaF_2$	9.0	160	42	21

pentafluorobenzene (**5k**)

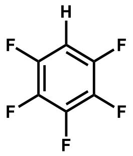

^{19}F NMR (376.5 MHz, in $PhCF_3$ with a sealed glass capillary containing 1,2-difluorobenzene in C_6D_6): δ -139.7 (dt, $^3J_{F\text{–}H}$ = 20.5, $^3J_{F\text{–}F}$ = 9.2 Hz, 2F), -155.1 (t, $^3J_{F\text{–}F}$ = 20.0 Hz, 1F), -163.1 – -163.3 (m, 2F).

1,2,4,5-tetrafluorobenzene (**5k'**)

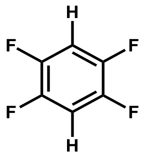

^{19}F NMR (376.5 MHz, in $PhCF_3$ with a sealed glass capillary containing 1,2–difluorobenzene in C_6D_6): δ –140.0 (m, 4F).

The spectral data matched with those reported in the literature.[S11]

- HDF of pentafluorobenzene

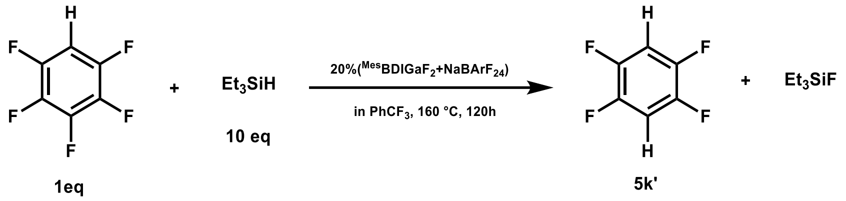

Following the general procedure, the reaction was performed with pentafluorobenzene (24.6 mg, 0.10 mmol), Et_3SiH (116.8 mg, 1.0 mmol, 10.0 equiv.) $^{Mes}BDIGaF_2$ (9.0 mg, 20.0 µmol, 20 mol%) and $NaBArF_{24}$ (17.8 mg, 20.0 µmol, 20 mol%) at 160 °C for 120 h. Quantitative ^{19}F-NMR revealed that 1,2,4,5-tetrafluorobenzene (**5k'**) was formed as shown in the table below.

Catalyst	Mass (mg)	Temp (°C)	5k' NMR yield (%)
$^{Mes}BDIGaF_2$	9.0	160	8

The spectral data matched with those reported in the HDF of hexafluorobenzezne and bromopentafluorobenzene.

- HDF of 2,3,5,6-tetrafluoropyridine

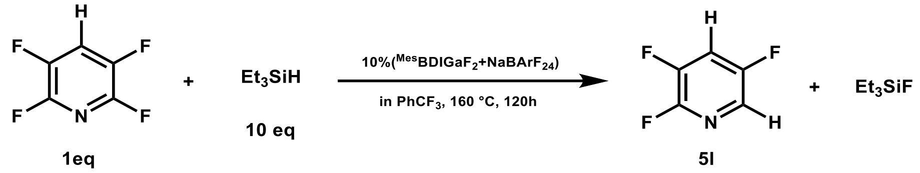

Following the general procedure, the reaction was performed with 2,3,5,6-tetrafluoropyridine (15.1 mg, 0.10 mmol), Et_3SiH (116.8 mg, 1.0 mmol, 10.0 equiv.), $^{Mes}BDIGaF_2$ (4.5 µmol, 10 mol%) and $NaBArF_{24}$ (8.9 mg, 10.0 µmol, 10 mol%) at 160 ℃ for 120 h. Quantitative ^{19}F-NMR revealed that 2,3,5-trifluoropyridine (**5l**) was formed as shown in the table below.

Catalyst	Mass (mg)	Temp (°C)	5l yield (%)
$^{Mes}BDIGaF_2$	4.5	160	24

2,3,5-trifluoropyridine (**5l**)

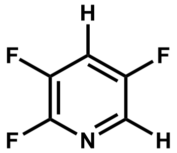

^{19}F NMR (376.5 MHz, in PhF with a sealed glass capillary containing 1,2-difluorobenzene in C_6D_6): δ -90.7– -90.7 (m, 1F), -127.8 – -128.2 (m, 1F), -135.0 – -135.2 (m, 1F).

The spectral data matched with those reported in the literature.[S14]

- HDF of decafluorobiphenyl

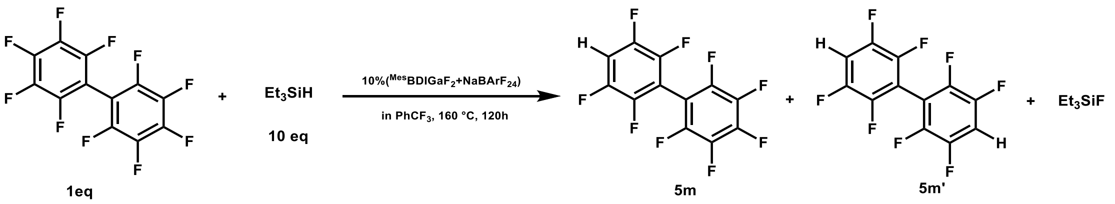

Following the general procedure, the reaction was performed with decafluorobiphenyl (33.4 mg, 0.10 mmol), Et_3SiH (116.8 mg, 1.0 mmol, 10.0 equiv.), $^{Mes}BDIGaF_2$ (4.5 mg, 10.0 μmol, 10 mol%) and $NaBArF_{24}$ (8.9 mg, 10.0 μmol, 10 mol%) at 160 °C for 120 h. Quantitative ^{19}F-NMR spectroscopy revealed that 2,2',3,3',4,5,5',6,6'-nonafluorobiphenyl (**5m**) and 2,2',3,3',5,5',6,6'-octafluoro-1,1'-biphenyl (**5m'**) were formed as shown in the table below.

Catalyst	Mass (mg)	Temp (°C)	5m NMR yield (%)	5m' NMR yield (%)
$^{Mes}BDIGaF_2$	4.5	160	42	21

2,2',3,3',4,5,5',6,6'-nonafluorobiphenyl (**5m**)

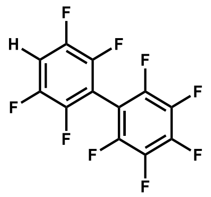

^{19}F NMR (376.5 MHz, in $PhCF_3$ with a sealed glass capillary containing 1,2-difluorobenzene in C_6D_6): δ -138.1 – -138.3 (m, 2F), -139.2 – -139.4 (m, 4F), -151.5 (t, $^3J_{F\text{–}H}$ = 21.9 Hz, 1F), -161.7 – -161.9 (m, 2F).

2,2',3,3',5,5',6,6'-Octafluoro-1,1'-biphenyl (**5m'**)

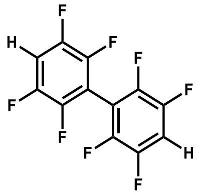

^{19}F NMR (376.5 MHz, in $PhCF_3$ with a sealed glass capillary containing 1,2-difluorobenzene in C_6D_6): δ -138.4 – -138.6 (m, 4F), -138.9 – -139.1 (m, 4F).

The spectral data matched with those reported in the literature.[S2]

- HDF of octafluoronaphthalene

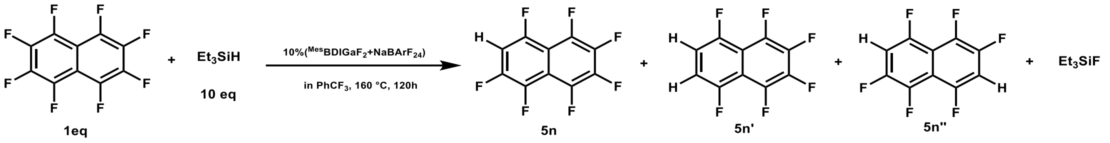

Following the general procedure, the reaction was performed with octafluoronaphthalene (27.2 mg, 0.10 mmol), Et_3SiH (116.8 mg, 1.0 mmol, 10.0 equiv.), $^{Mes}BDIGaF_2$ (4.5 mg, 10.0 µmol, 10 mol%) and $NaBArF_{24}$ (8.9 mg, 10.0 µmol, 10 mol%) at 160 °C for 12 h. Quantitative ^{19}F-NMR spectroscopy revealed that 2H-heptafluoro-naphthalene (**5n**), 1,2,3,4,5,8-hexafluoronaphthalene (**5n'**) and 1,2,4,5,6,8-hexafluoronaphthalene (**5n''**) were formed as shown in the table below.

Catalyst	Mass (mg)	Temp (°C)	5n NMR yield (%)	5n' NMR yield (%)	5n'' NMR yield (%)
$^{Mes}BDIGaF_2$	4.5	160	14	10	46

2H-heptafluoro-naphthalene (**5n**)

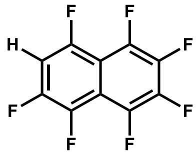

^{19}F NMR (376.5 MHz, in $PhCF_3$ with a sealed glass capillary containing 1,2-difluorobenzene in C_6D_6): δ -116.1 (d, $^3J_{F-H}$ = 64.0 Hz, 1F), -134.4 – -134.6 (s, 1F), -145.6 (dt, $^3J_{F-H}$ = 64.0, $^3J_{F-F}$ = 16.0 Hz, 1F), -147.4 (dt, $^3J_{F-F}$ = 58.0, 16.0 Hz, 1F), -151.2 – -151.4 (m, 1F), -155.2 (t, $^3J_{F-F}$ = 18.0 Hz, 1F), -157.8 – -158.2 (m, 1F).

1,2,3,4,5,8-hexafluoronaphthalene (**5n'**)

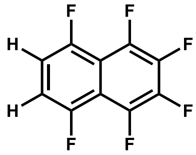

^{19}F NMR (376.5 MHz, in $PhCF_3$ with a sealed glass capillary containing 1,2-difluorobenzene in C_6D_6): δ -117.1 (d, 2F, $^3J_{F-H}$ = 64.0 Hz), -134.9 – -135.1 (m, 2F), -150.5 (d, 2F, $^3J_{F-H}$ = 64.0 Hz).

1,2,4,5,6,8-hexafluoronaphthalene (**5n''**)

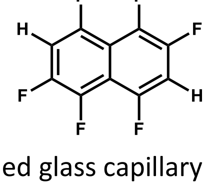

^{19}F NMR (376.5 MHz, in $PhCF_3$ with a sealed glass capillary containing 1,2-difluorobenzene in C_6D_6): δ -117.6 – -118.0 (m, 2F), -137.0 – -137.2 (m, 2F), -149.2 – -149.8 (m, 2F).

The spectral data matched with those reported in the literature.[S2]

- HDF of perfluoroprop-1-ene

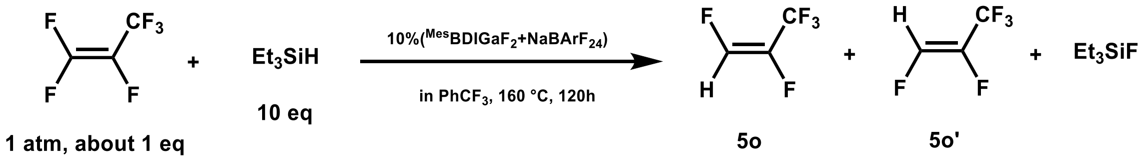

Following the general procedure, the reaction was performed with perfluoroprop-1-ene (1 atm. in J. Young NMR tube, approx.. 0.1 mmol, 14.9 mg), Et_3SiH (116.8 mg, 1.0 mmol, 10.0 equiv.), $^{Mes}BDIGaF_2$ (9.0 mg, 20.0 µmol, 20 mol%) and $NaBArF_{24}$ (17.8 mg, 20.0 µmol, 20 mol%) at 160 °C for 120 h. Quantitative ^{19}F-NMR spectroscopy revealed that (E)-1,2,3,3,3-pentafluoroprop-1-ene (**5o**) and (Z)-1,2,3,3,3-pentafluoroprop-1-ene (**5o'**) were formed as shown in the table below.

Catalyst	Mass (mg)	Temp (°C)	5o NMR yield (%)	5o' NMR yield (%)
$^{Mes}BDIGaF_2$	4.5	160	23	31

(E)-1,2,3,3,3-pentafluoroprop-1-ene (**5o**)

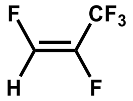

^{19}F NMR (376.5 MHz, in $PhCF_3$ with a sealed glass capillary containing 1,2-difluorobenzene in C_6D_6): δ -69.9 (dd, $^3J_{F\text{–}F}$ = 20.4 Hz, $^4J_{F\text{–}H}$ =11.3 Hz, 3F), -165.8 – -166.8 (m, 1F), -179.7– -180.4 (m, 1F).

(Z)-1,2,3,3,3-pentafluoroprop-1-ene (**5o'**)

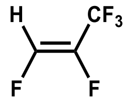

^{19}F NMR (376.5 MHz, in $PhCF_3$ with a sealed glass capillary containing 1,2-difluorobenzene in C_6D_6): δ -72.9 (dd, $^3J_{F\text{–}F}$ = 13.9 Hz, $^4J_{F\text{–}H}$ = 5.7 Hz, 3F), -155.6 – -156.0 (m, 1F), -159.1 – -159.4 (m, 1F).

The spectral data matched with those reported in the literature.[S14]

- HDF of perfluorocyclopent-1-ene

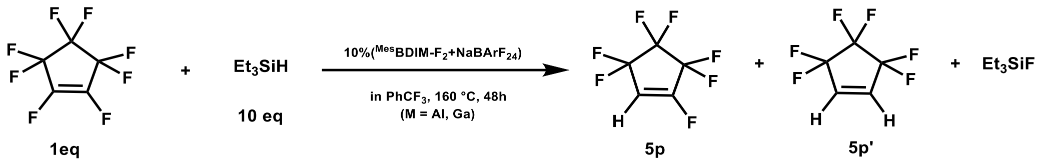

Following the general procedure, the reaction was performed with perfluorocyclopent-1-ene (21.3 mg, 0.10 mmol), Et_3SiH (116.8 mg, 1.0 mmol, 10.0 equiv.), $^{Mes}BDIMF_2$ (10.0 µmol, 10 mol%) and $NaBArF_{24}$ (8.9 mg, 10.0 µmol, 10 mol%) at 160 °C for 48 h. Quantitative ^{19}F-NMR revealed that 1,3,3,4,4,5,5-heptafluorocyclopent-1-ene (**5p**) and 3,3,4,4,5,5-hexafluorocyclopent-1-ene (**5p'**) were formed as shown in the table below.

Catalyst	Mass (mg)	Temp (°C)	5p NMR yield (%)	5p' NMR yield (%)
$^{Mes}BDIAlF_2$	4.0	160	41	57
$^{Mes}BDIGaF_2$	4.5	160	33	65

1,3,3,4,4,5,5-heptafluorocyclopent-1-ene (**5p**)

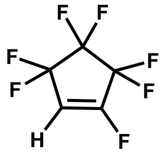

^{19}F NMR (376.5 MHz, in PhF with a sealed glass capillary containing 1,2–difluorobenzene in C_6D_6): -107.1 (d, $^3J_{F–H}$ = 11.9 Hz, 2F), -120.3 (d, $^3J_{F–H}$ = 14.2 Hz, 2F), -124.9 (d, $^3J_{F–H}$ = 14.8 Hz, 1F), -130.4 (s, 2F).

3,3,4,4,5,5-hexafluorocyclopent-1-ene (**5p'**)

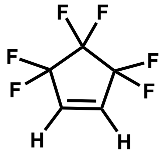

^{19}F NMR (376.5 MHz, in PhF with a sealed glass capillary containing 1,2-difluorobenzene in C_6D_6): δ -110.0 (s, 4F), -132.4 – -132.6 (m, 2F).

The spectral data matched with those reported in the literature.[S11]

4) Single Crystal X-ray Diffraction Data

- The X-ray crystal structure of **2j**

Crystal data for **2j**: $C_{36}H_{15}F_{12}PS_3$, *M* = 802.63, orthorhombic, *Pca*2$_1$ (no. 29), *a* = 24.8245(12), *b* = 8.5079(4), *c* = 30.2964(16) Å, *V* = 6398.8(5) Å^3, *Z* = 8 [two independent molecules], D_c = 1.666 g cm^{-3}, μ(Cu-Kα) = 3.502 mm^{-1}, *T* = 173 K, colourless needles, Agilent Xcalibur PX Ultra A diffractometer; 9898 independent measured reflections (R_{int} = 0.1108), F^2 refinement, [16-17] R_1(obs) = 0.0859, wR_2(all) = 0.2208, 5424 independent observed absorption-corrected reflections [$|F_o| > 4\sigma(|F_o|)$, completeness to θ_{full}(67.7°) = 98.3%], 939 parameters. The absolute structure of **2j** was determined by use of the Flack parameter [*x* = –0.002(87)]. CCDC 2235593.

The crystal of **2j** that was studied was found to be a three-component twin in a *ca*. 40:32:28 ratio, with the two major lattices related by the approximate twin law [–1.00 0.00 0.00 0.00 1.00 –0.03 0.00 –0.04 –1.00]. The structure was found to contain two independent molecules, **2j-A** and **2j-B**, in the asymmetric unit. Thermal parameter restraints were applied to C8A, C19A, C1B, C6B, C16B, C17B, C21B, C26B, and C27B to keep these atoms having sensibly shaped thermal ellipsoids.

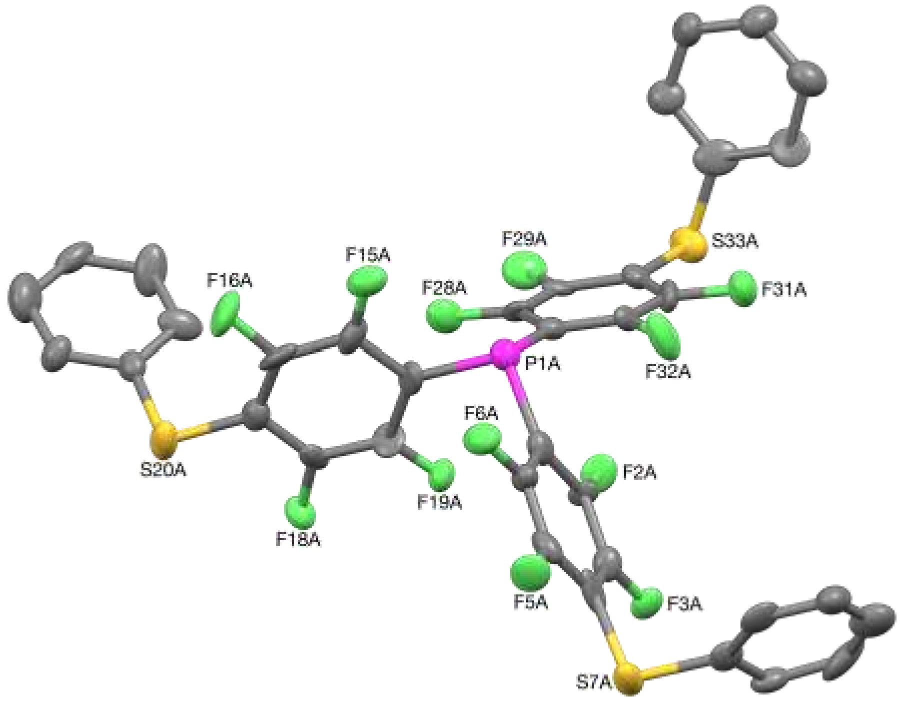

Figure S3. The structure of **2j-A**, one of the two independent molecules present in the crystal of **2j** (50% probability ellipsoids). [S16-S17]

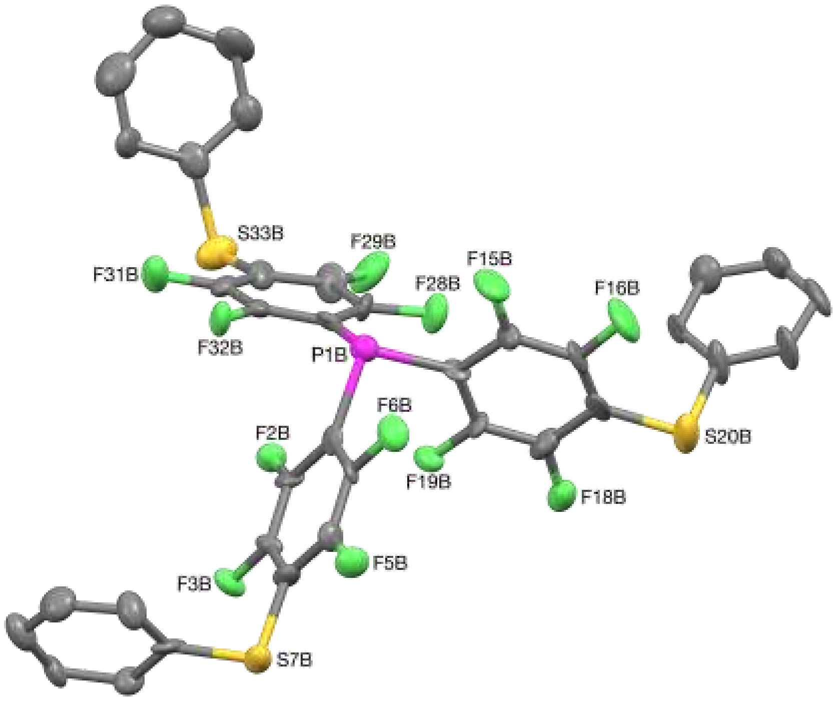

Figure S4. The structure of **2j-B**, one of the two independent molecules present in the crystal of **2j** (50% probability ellipsoids). [S16-S17]

- The X-ray crystal structure of **2h**

Crystal data for **2h**: $C_{18}H_9F_5S$, M = 352.31, monoclinic, $C2$ (no. 5), a = 18.1081(11), b = 5.9794(3), c = 27.4902(18) Å, β = 102.389(7)°, V = 2907.2(3) Å^3, Z = 8 [two independent molecules], D_c = 1.610 g cm^{-3}, μ(Cu-Kα) = 2.500 mm^{-1}, T = 173 K, colourless plates, Agilent Xcalibur PX Ultra A diffractometer; 5213 independent measured reflections (R_{int} = 0.0741), F^2 refinement,[S16-S17] R_1(obs) = 0.0712, wR_2(all) = 0.2029, 3735 independent observed absorption-corrected reflections [$|F_o| > 4\sigma(|F_o|)$, completeness to θ_{full}(67.7°) = 99.9%], 433 parameters. The absolute structure of **2h** could not be unambiguously determined [Flack parameter x = –0.11(5)]. CCDC 2235594.

The structure of **2h** was found to contain two independent molecules, **2h-A** and **2h-B**, in the asymmetric unit.

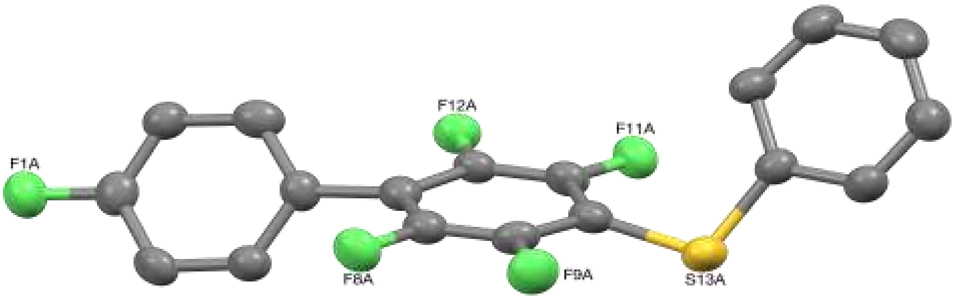

Figure S5. The structure of **2h-A**, one of the two independent molecules present in the crystal of **2h** (50% probability ellipsoids). [S16-S17]

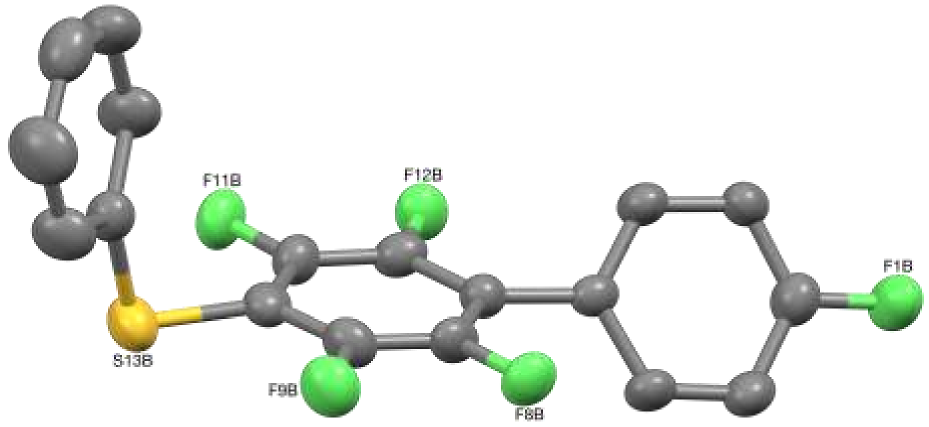

Figure S6. The structure of **2h-B**, one of the two independent molecules present in the crystal of **2h** (50% probability ellipsoids). [S16-S17]

- The X-ray crystal structure of $^{Mes}BDIAlS_2Ph_2$

Crystal data for **4**-**Al**: $C_{35}H_{39}AlN_2S_2$, *M* = 578.78, triclinic, *P*-1 (no. 2), *a* = 10.4157(4), *b* = 10.8795(6), *c* = 15.8587(7) Å, α = 95.577(4), β = 96.355(4), γ = 115.988(5)°, *V* = 1583.94(14) Å^3, *Z* = 2, D_c = 1.214 g cm^{-3}, μ(Cu-Kα) = 1.980 mm^{-1}, *T* = 173 K, colourless tabular needles, Agilent Xcalibur PX Ultra A diffractometer; 12670 independent measured reflections (R_{int} = 0.0788), F^2 refinement,[16-17] R_1(obs) = 0.0499, wR_2(all) = 0.1457, 9323 independent observed absorption-corrected reflections [$|F_o| > 4\sigma(|F_o|)$, completeness to θ_{full}(67.7°) = 99.8%], 370 parameters. CCDC 2235595.

The crystal of **4**-**Al** that was studied was found to be a two component twin in a *ca*. 52:48 ratio, with the two lattices related by the approximate twin law [0.97 –0.02 –0.08 0.04 1.02 0.11 0.09 –0.19 0.98].

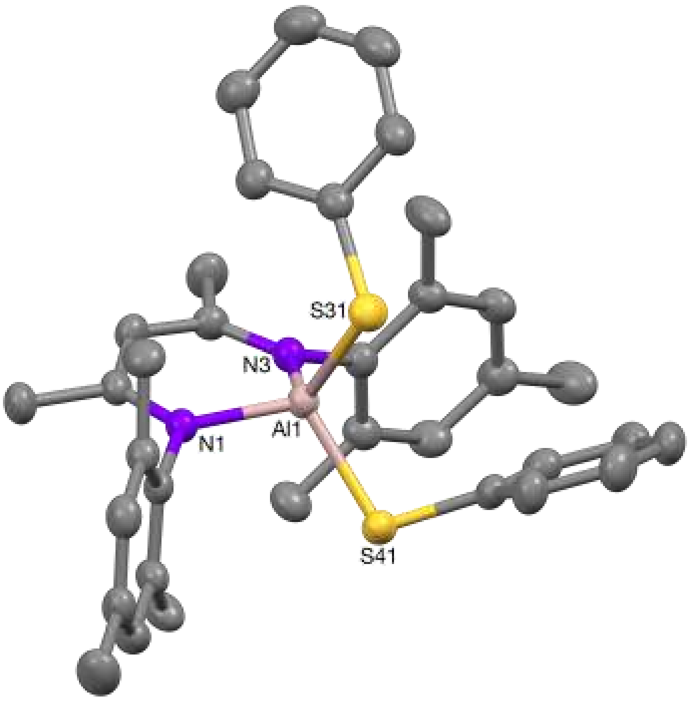

Figure S7. The crystal structure of **4**-**Al** (50% probability ellipsoids).[S16-S17]

5) Mechanistic Studies of Group 13-catalyzed Defluorofunctionalisation reactions

5.1. Mechanistic Probe Experiments for Thiodefluorination

5.1.1. Stoichiometric reaction of ${}^{Mes}BDIMF_2$ with Me_3Si-SPh

Procedure One: In the glovebox, ${}^{Mes}BDIAlF_2$ (10.0 mg, 0.025 mmol) and Me_3Si-SPh (14.3 mg, 0.075 mmol) were weighed in a vial, and dissolved in 0.5 ml C_6D_6. The reaction mixture was transferred to a J. Young NMR tube. The reaction mixture was heated to 100 °C for 48h and monitored by ${}^{1}H$-NMR and ${}^{19}F$-NMR spectroscopy. The NMR spectra are shown in Figure S8 and Figure S9 and suggest the formation of **3-Al** and **4-Al**. Repeated attempts to crystallise the products from this reaction led to the isolation of ${}^{Mes}BDIAl(S\text{-}C_6H_5)_2$. **3-Al** was characterised by a single fluorine resonance along with a 1:1 ratio of thiolate : β-diketiminate ligands and its assignment is further supported by preparation of an analogue **3-Al'**.

${}^{Mes}BDIAl(S\text{-}C_6H_5)F$ (**3-Al**) NMR Yield (%)	${}^{Mes}BDIAl(S\text{-}C_6H_5)_2$ (**4-Al**) NMR Yield (%)
71	22

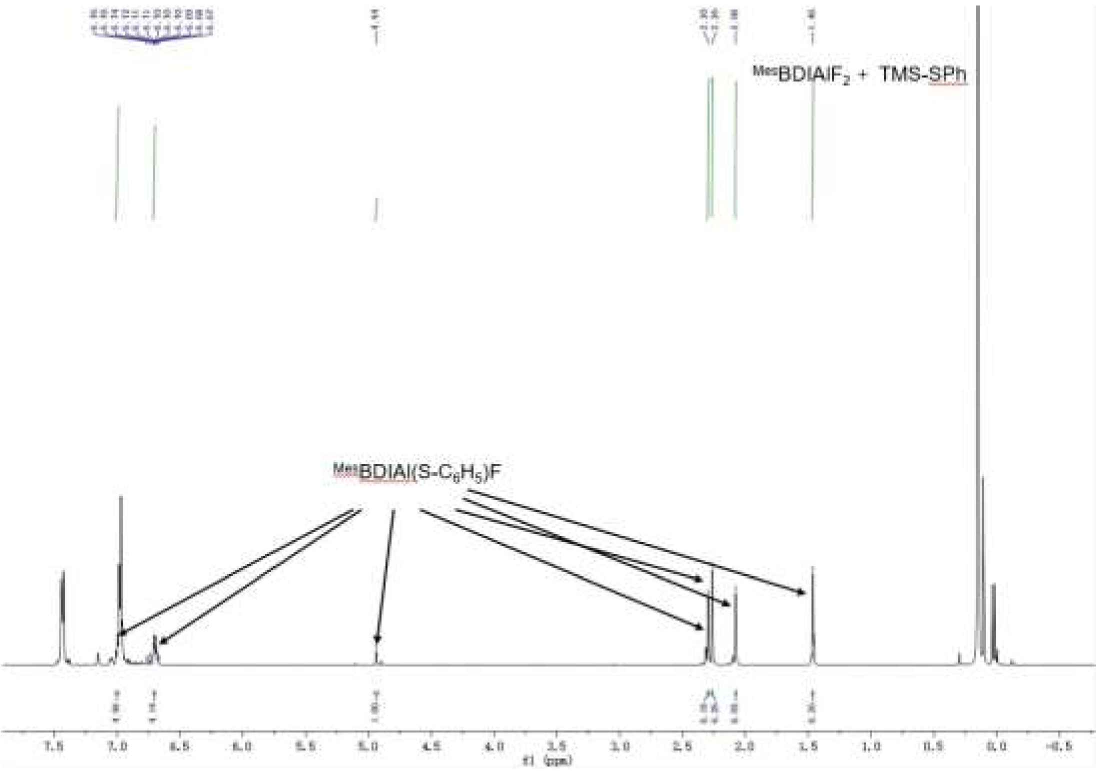

Figure S8. ${}^{1}H$ NMR (C_6D_6, 400 MHz)NMR spectrum of the mixture of ${}^{Mes}BDIAlF_2$ + Me_3Si-SPh in C_6D_6

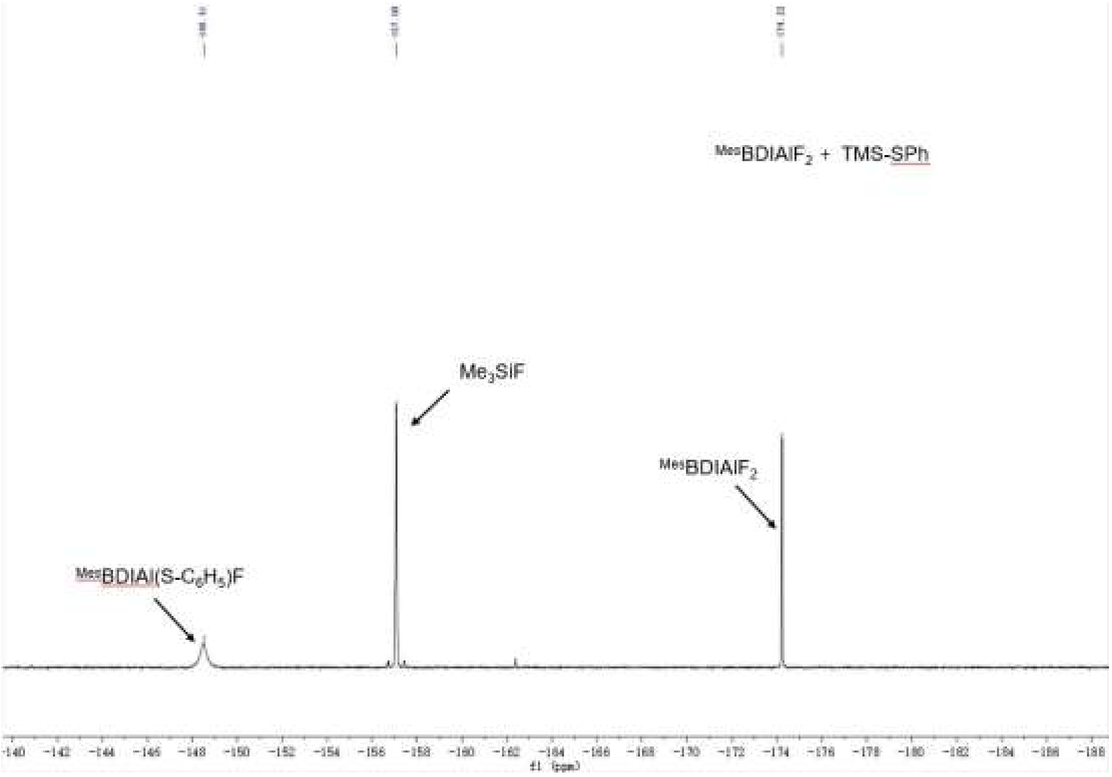

Figure S9. ^{19}F NMR (C_6D_6, 376.5 MHz) NMR spectrum of the mixture of ^{Mes}BDIAlF$_2$ + Me_3Si-SPh in C_6D_6

^{Mes}BDIAlFS-C_6H_5 (3-Al)

^{1}H-NMR (C_6D_6, 298 K, 400 MHz) δ = 7.04 – 6.96 (m, 5H), 6.74 – 6.68 (m, 4H), 4.94 (s, 1H), 2.30 (s, 6H), 2.26 (s, 6H), 2.08 (s, 6H), 1.46 (s, 6H).

^{19}F-NMR (376.5 MHz, C_6D_6, 298 K, 400 MHz): δ -148.5 (bs, 1F).

Procedure Two: In the glovebox, ^{Mes}BDIBF$_2$ (9.6 mg, 0.025 mmol) and Me_3Si-SPh (14.3 mg, 0.075 mmol) were weighed in a vial, and dissolved in 0.5 ml C_6D_6. The reaction mixture was transferred to a J. Young NMR tube. The reaction mixture was heated to 100 °C for 48h and monitored by ^{1}H-NMR and ^{19}F-NMR spectroscopy. NMR spectrum showed no new product formation.

5.1.2. Stoichiometric reaction of $^{Mes}BDIAlF_2$ and $^{Mes}BDIAl(S\text{-}C_6H_4Me)_2$

Procedure: In the glovebox, $^{Mes}BDIAlF_2$ (10.0 mg, 0.025 mmol), $^{Mes}BDIAl(S\text{-}C_6H_4Me)_2$ (15.2 mg, 0.025 mmol) were weighed into a vial, and dissolved in 0.5 ml C_6D_6. The reaction mixture was transferred to J. Young NMR tube. The reaction mixture was heated to 100 °C for 48h and then monitored by ^1H-NMR and ^{19}F-NMR spectroscopy. The NMR spectra were showed in Figure S10 and Figure S11.

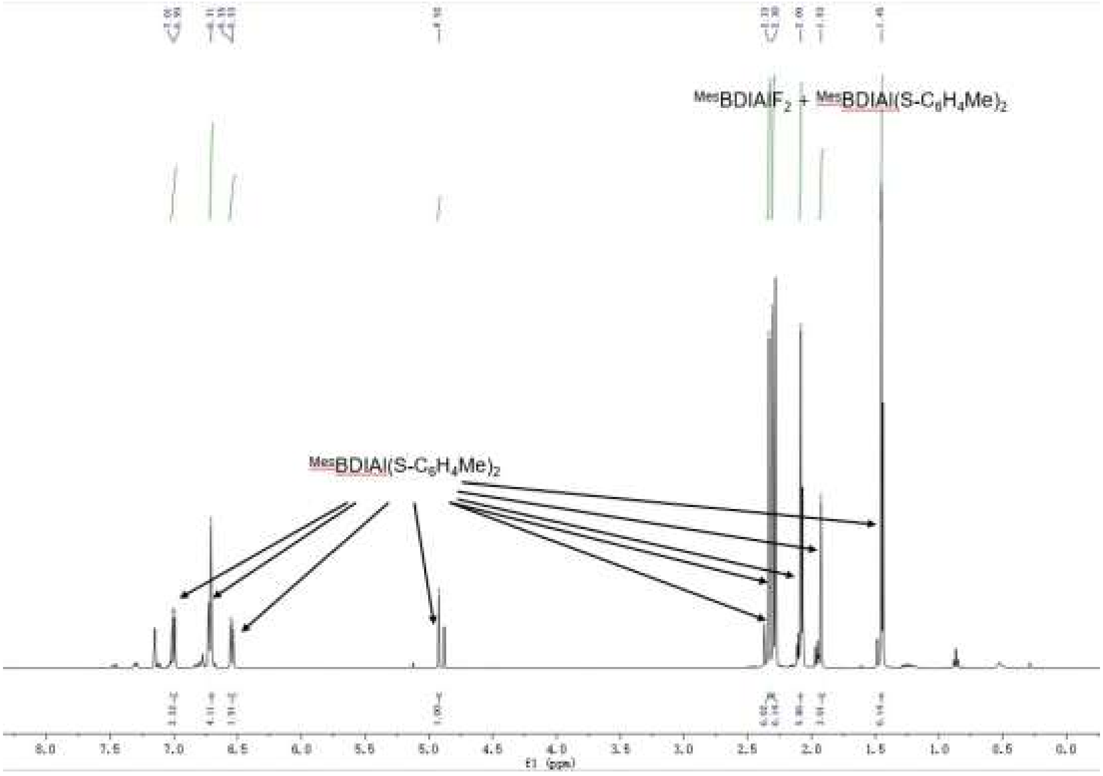

Figure S10. ^1H NMR (C_6D_6, 400 MHz)-NMR spectrum of the mixture of $^{Mes}BDIAlF_2$ + $^{Mes}BDIAl(S\text{-}C_6H_4Me)_2$ (1:1) in C_6D_6

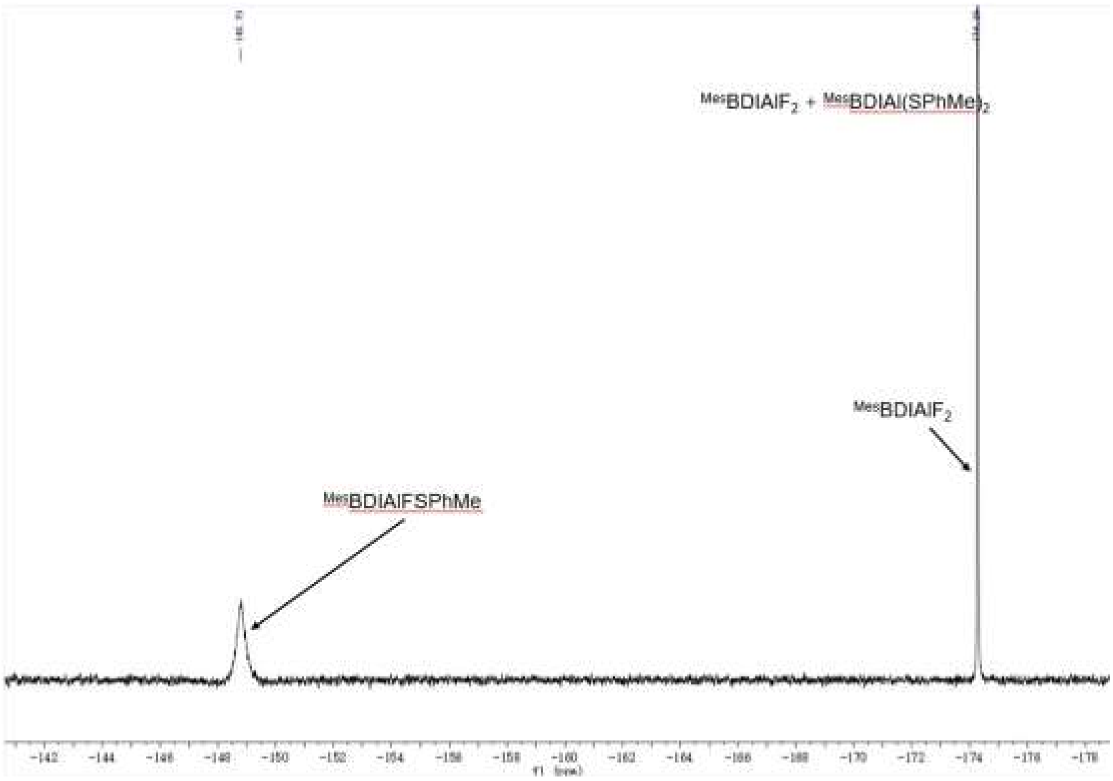

Figure S11. ^{119}F NMR (C_6D_6, 376.5 MHz)-NMR spectrum of the mixture of ^{Mes}BDIAlF$_2$ + ^{Mes}BDIAl(S-$C_6H_4Me)_2$ (1:1) in C_6D_6

^{Mes}BDIAl(S-C_6H_4Me)$_2$ (4-Al'):

^{1}H-NMR (C_6D_6, 298 K, 400 MHz) δ = 7.00 (d, *J* = 8.1 Hz, 2H), 6.71 (s, 1H), 6.54 (d, *J* = 7.9 Hz, 2H), 4.92 (s, 1H), 2.33 (s, 6H), 2.30 (s, 6H), 2.09 (s, 3H), 1.93 (s, 6H), 1.45 (s, 6H).

^{19}F-NMR (376.5 MHz, C_6D_6, 298 K, 400 MHz): δ -148.5 (bs, 1F).

5.1.3. Stoichiometric reaction of $^{Mes}BDIAl(S\text{-}C_6H_4Me)_2$ and pentafluoropyridine

Procedure: In the glovebox, $^{Mes}BDIAl(S\text{-}C_6H_4Me)_2$ (15.2 mg, 0.025 mmol) and pentafluoropyridine (4.3 mg, 0.025 mmol), were weighed into a vial, dissolved in 0.5 ml $C_6H_5CF_3$ and transferred to a J. Young NMR tube. The reaction mixture was heated to 100 °C for 60h and monitored by ^{19}F-NMR spectroscopy. $^{Mes}BDIAlF_2$ and 2,3,5,6-tetrafluoro-4-(p-tolylthio)pyridine (**2n**) were observed and the NMR spectra are shown in Figure S12.

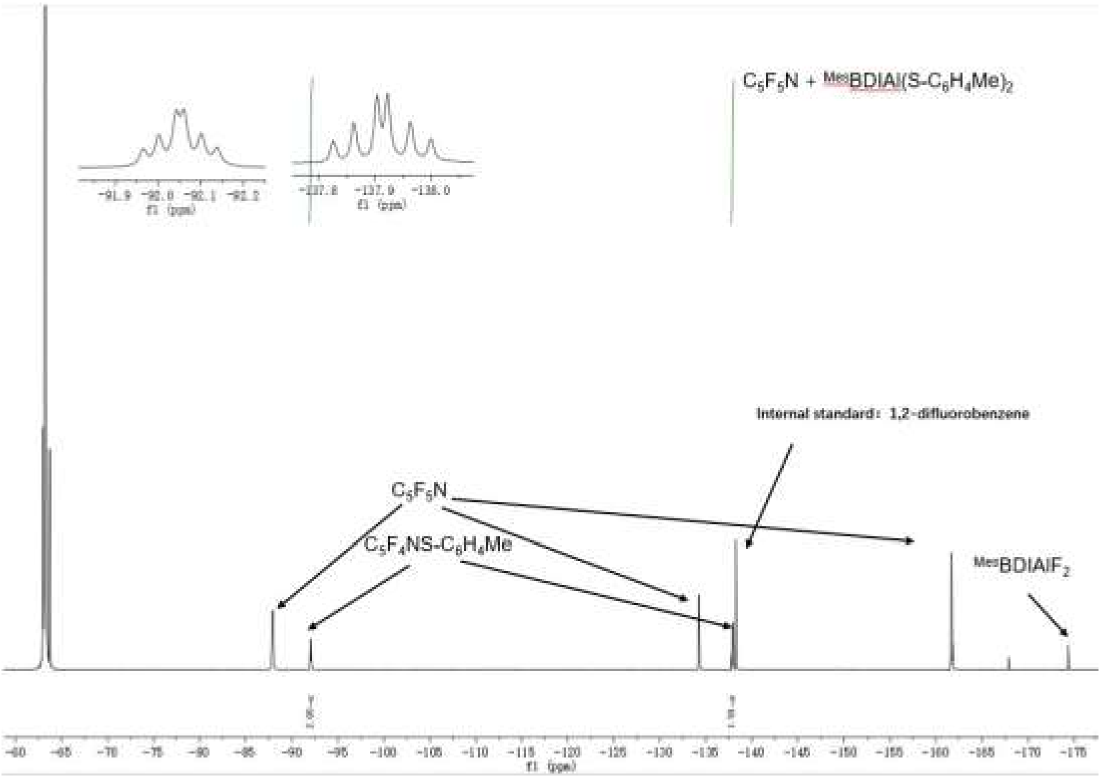

Figure S12. ^{1}H-NMR spectrum of the mixture of MesBDIAl($S\text{-}C_6H_4Me)_2$ and pentafluoropyridine (1:1) in $C_6H_5CF_3$

2,3,5,6-tetrafluoro-4-(p-tolylthio)pyridine (**2n**)

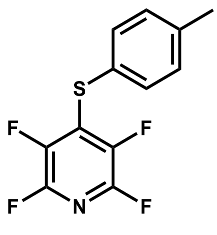

^{19}F-NMR (376.5 MHz, in $C_6H_5CF_3$ with a sealed glass capillary containing 1,2-difluorobenzene in C_6D_6): δ -91.8 – 92.3 (m, 2F, o-F), -137.8 – -138.1 (m, 2F).

5.1.4. Results and Characterisation Data of Thiodefluorination Products (cat: ^{Mes}BDIAl(S-$C_6H_4Me)_2$)

- Thiodefluorination of pentafluoropyridine

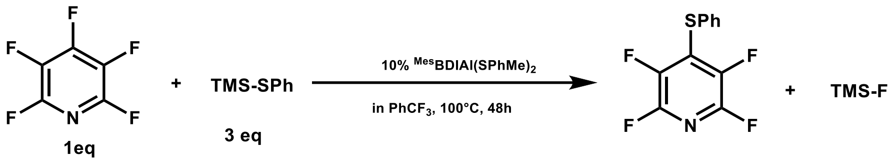

Following the general procedure, the reaction was performed with pentafluoropyridine (42.4 mg, 0.25 mmol), Me_3Si-SPh (142.02 mg, 0.75 mmol, 3 equiv.) and ^{Mes}BDIAl(SPhMe)$_2$ (**4-Al'**, 25 µmol, 10 mol%) at 100 °C for 48 h. Quantitative ^{19}F-NMR spectroscopy revealed that 2,3,5,6-Tetrafluoropyridine (**2a**) and 2,3,5,6-tetrafluoro-4-(phenylthio)pyridine (**2n**) were formed as shown in the table below. The NMR spectra were showed in Figure S13.

Catalyst	Mass (mg)	Temp (°C)	2a NMR yield (%)	2n NMR yield (%)
^{Mes}BDIAl(SPhMe)$_2$	10.0	100	91	4

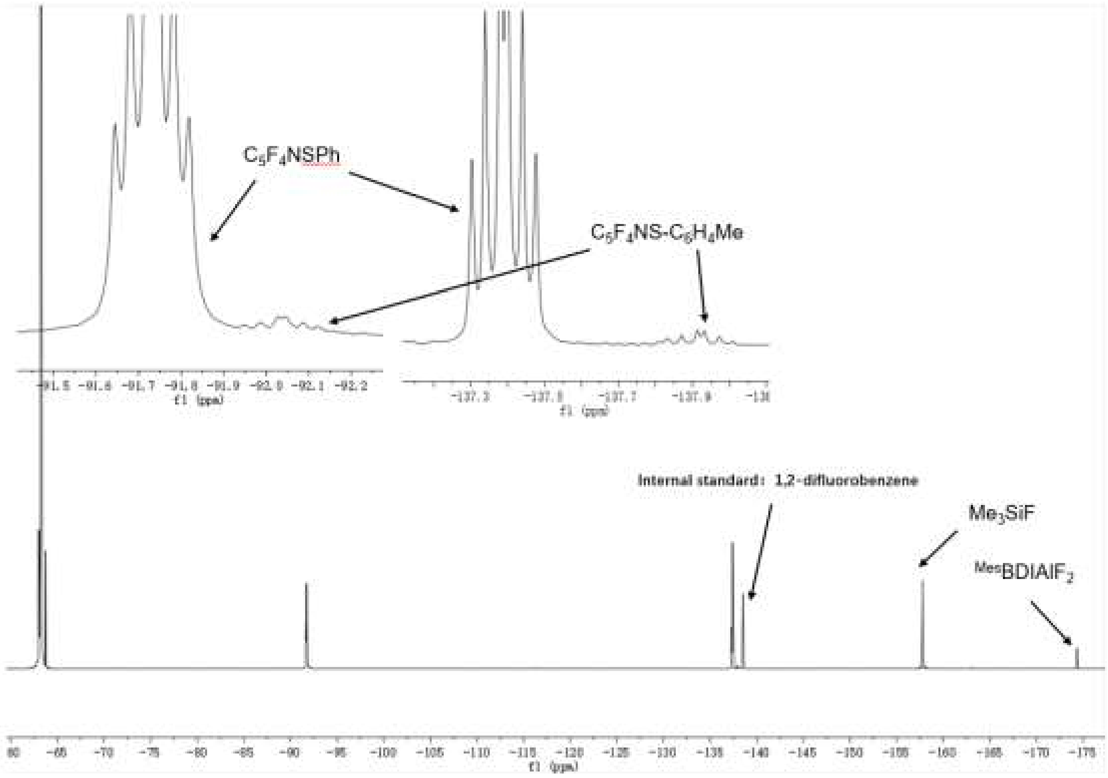

Figure S13. ^{19}F-NMR spectrum showed the chemical shift of the product distribution (cat: ^{Mes}BDIAl(S-$C_6H_4Me)_2$ (**4-Al'**).

5.2. Mechanistic Probe Experiments for Hydrodefluorination

5.2.1. Stoichiometric reaction of $^{Mes}BDIMF_2$ with $NaBArF_{24}$

Procedure: In the glovebox, $^{Mes}BDIAlF_2$ (**1-Al**, 10.0 mg, 0.025 mmol) and $NaBArF_{24}$ (22.3 mg, 0.025 mmol) were weighed in a vial and dissolved in 1ml PhF. The mixture was transferred to a J. Young NMR tube. The reaction mixture was monitored by ^{19}F-NMR spectroscopy at 25 °C. The NMR spectra were showed in Figure S14 and Figure S15.

	Chemical shift (δ)
$^{Mes}BDIAlF_2$	-174.5 (Al***F***)
$^{Mes}BDIAlHF$	-157.7 (AlH***F***) (See Figure S19)
$^{Mes}BDIAlF_2 \cdot NaBArF_{24}$:	-180.4 (Al***F***)
$^{Mes}BDIAlHF \cdot NaBArF_{24}$:	-172.2 (AlH***F***) (See Figure S22)

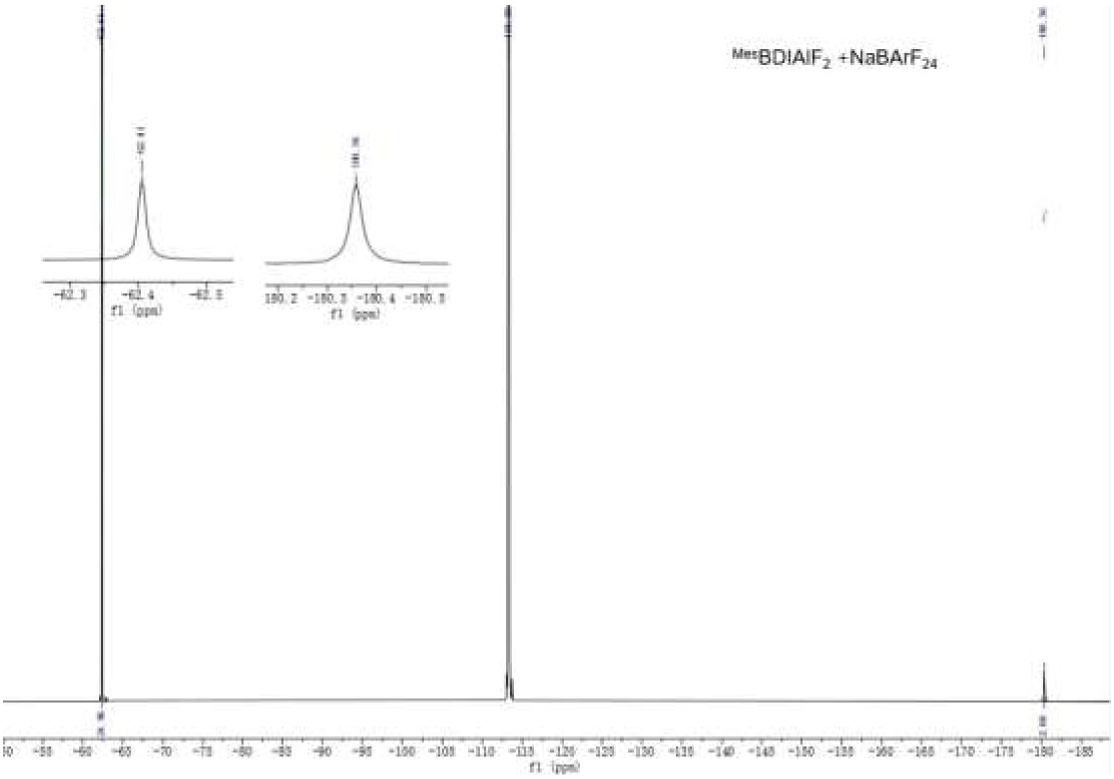

Figure S14. ^{19}F-NMR spectrum of the mixture of $^{Mes}BDIAlF_2$ +$NaBArF_{24}$ (1:1) in dry PhF

$^{Mes}BDIAlF_2 \cdot NaBArF_{24}$ (**1-Al·NaBArF$_{24}$**):
^{19}F-NMR (376.5 MHz, in PhF (standard capillary: PhF (113.3 ppm in C_6D_6))): δ -62.4 (s, 24F), -180.4 (s, 2F).

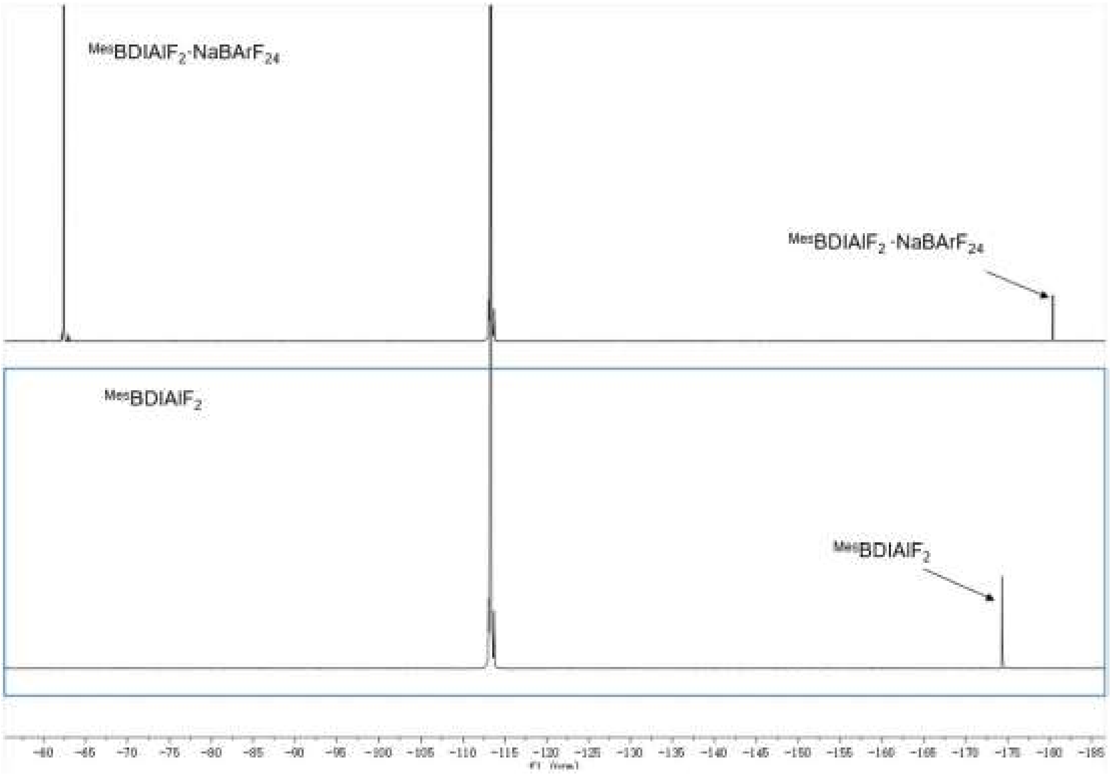

Figure S15. ^{19}F-NMR spectrum showing the chemical shift difference between the mixture of ^{Mes}BDIAlF$_2$ +NaBArF$_{24}$ (1:1) and pure ^{Mes}BDIAlF$_2$ in dry PhF

Procedure: In the glovebox, $^{Mes}BDIGaF_2$ (**1-Ga**, 10.0 mg, 0.023 mmol) and $NaBArF_{24}$ (20.1 mg, 0.023 mmol) were weighed in a vial and dissolved in 1ml PhF. The mixture was transferred to a J. Young NMR tube. The reaction mixture was monitored by ^{19}F-NMR spectroscopy at 25 °C. The NMR spectra are shown in Figure S16.

	Chemical shift (δ)
$^{Mes}BDIGaF_2$	-200.7 (Ga***F***)
$^{Mes}BDIGaHF$	-182.0 (GaH***F***) (See Figure 23)
$^{Mes}BDIGaF_2 \cdot NaBArF_{24}$:	-203.1 (Ga***F***)
$^{Mes}BDIGaHF \cdot NaBArF_{24}$:	-197.3 (GaH***F***) (See Figure 23)

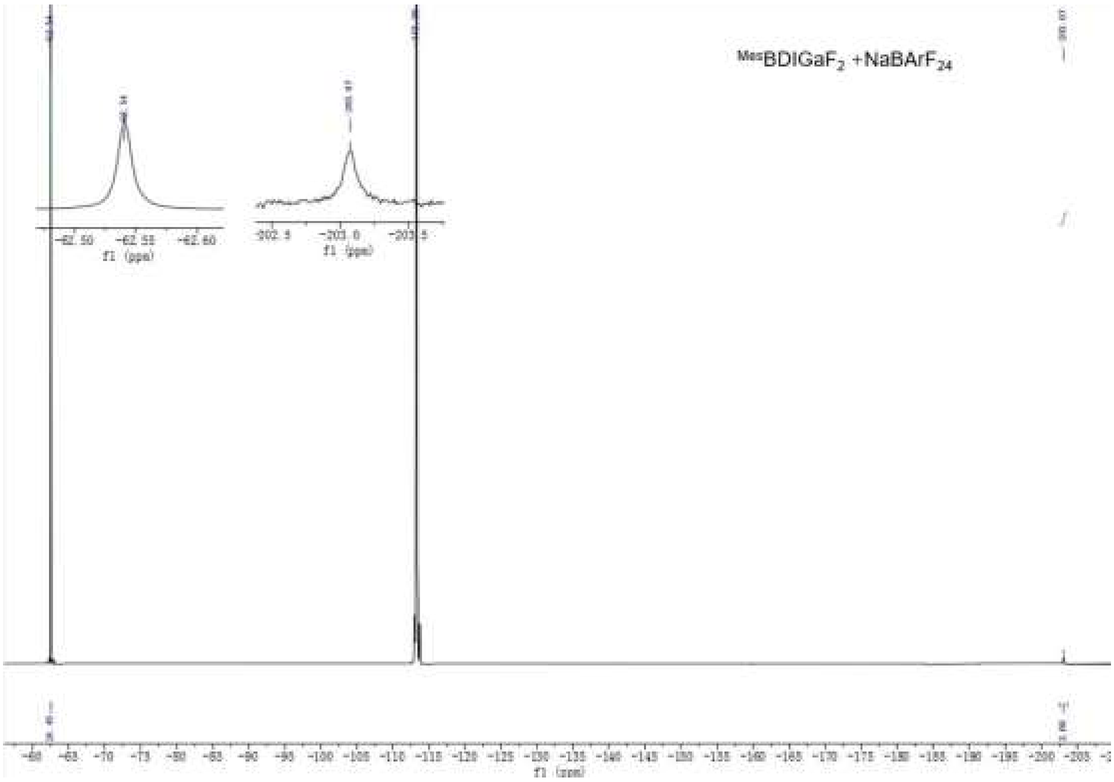

Figure S16. ^{19}F-NMR spectrum of the mixture of $^{Mes}BDIGaF_2$ +$NaBArF_{24}$ (1:1) in dry PhF

$^{Mes}BDIGaF_2 \cdot NaBArF_{24}$:

^{19}F-NMR (376.5 MHz, in PhF (standard capillary: PhF (113.3 ppm in C_6D_6))): δ -62.5 (s, 24F), -203.1 (s, 2F).

5.2.2. VT-NMR study on $^{Mes}BDIAlF_2$ with $NaBArF_{24}$

Procedure: In a glovebox, $^{Mes}BDIAlF_2$ (**1-Al**, 5 – 40 mg, 0.0125 – 0.05 mmol) and $NaBArF_{24}$ (0 or 22.3 mg, 0.025 mmol) were weighed in a vial and dissolved in 1ml PhF. The mixture was transferred to a J. Young NMR tube. Each reaction mixture was monitored by ^{19}F-NMR spectroscopy across 233 to 313 K. The chemical shift of the ^{19}F resonance of the fluoride vs Na : Al ratio for each temperature is plotted in Figure S17.

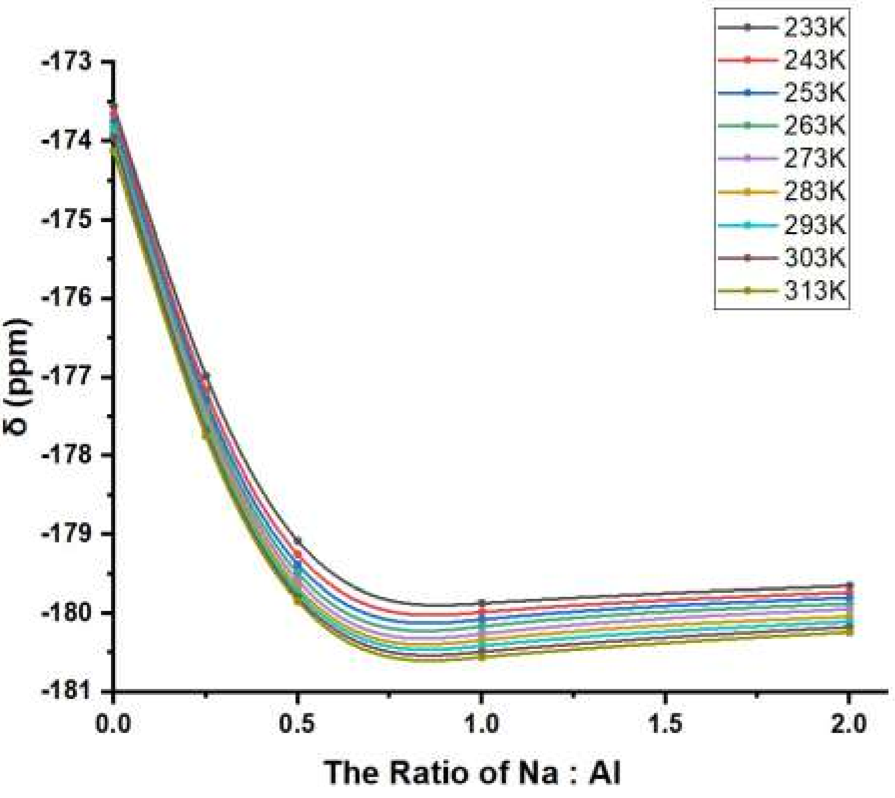

Figure S17. VT-NMR experiments tested the reactions of $^{Mes}BDIAlF_2$ and $NaBArF_{24}$. The x-axis represents the chemical shift of [Al]-F in $^{Mes}BDIAlF_2$, and the y-axis means the raw material ratio of $^{Mes}BDIAlF_2$ and $NaBArF_{24}$ in PhF.

5.2.3. Stoichiometric reaction of ^{Mes}BDIGaF$_2$, NaBArF$_{24}$ with Et$_3$SiH

Procedure: In the glovebox, ^{Mes}BDIGaF$_2$ (**1-Ga**, 5.0 mg, 0.011 mmol), NaBArF$_{24}$ (10.0 mg, 0.011 mmol) and Et$_3$SiH (4.0 mg, 0.03 mmol) were weighed in a vial, dissolved in 0.6 ml PhF and transferred into a J. Young NMR tube. The reaction mixture was heated to 80 °C for 6h and monitored by ^{19}F-NMR spectroscopy. The ^{19}F-NMR spectrum showed a singlet peak at -176.1 ppm which is assigned to Et$_3$SiF based on comparison to the literature. The NMR spectra are shown in Figure S18.

Control experiments 1: In the glovebox, ^{Mes}BDIGaF$_2$ (**1**-Ga, 5.0 mg, 0.011 mmol) and Et$_3$SiH (4.0 mg, 0.03 mmol) were weighed in a vial, dissolved in 0.6 ml PhF and placed in a J. Young NMR tube. The reaction mixture was heated to 80 °C for 6h and monitored by ^{19}F-NMR and ^{1}H-NMR spectroscopy. No reaction was observed.

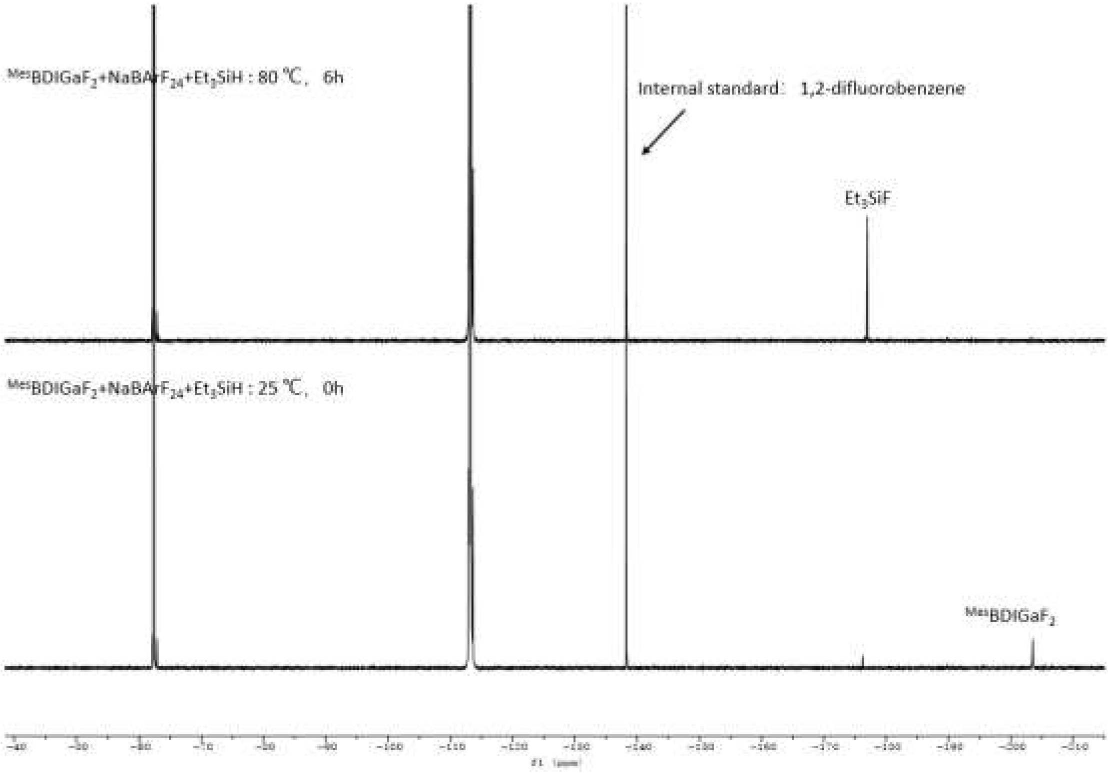

Figure S18. ^{19}F-NMR spectrum of the mixture of ^{Mes}BDIGaF$_2$ + NaBArF$_{24}$ + Et$_3$SiH (1:1:3) in dry PhF

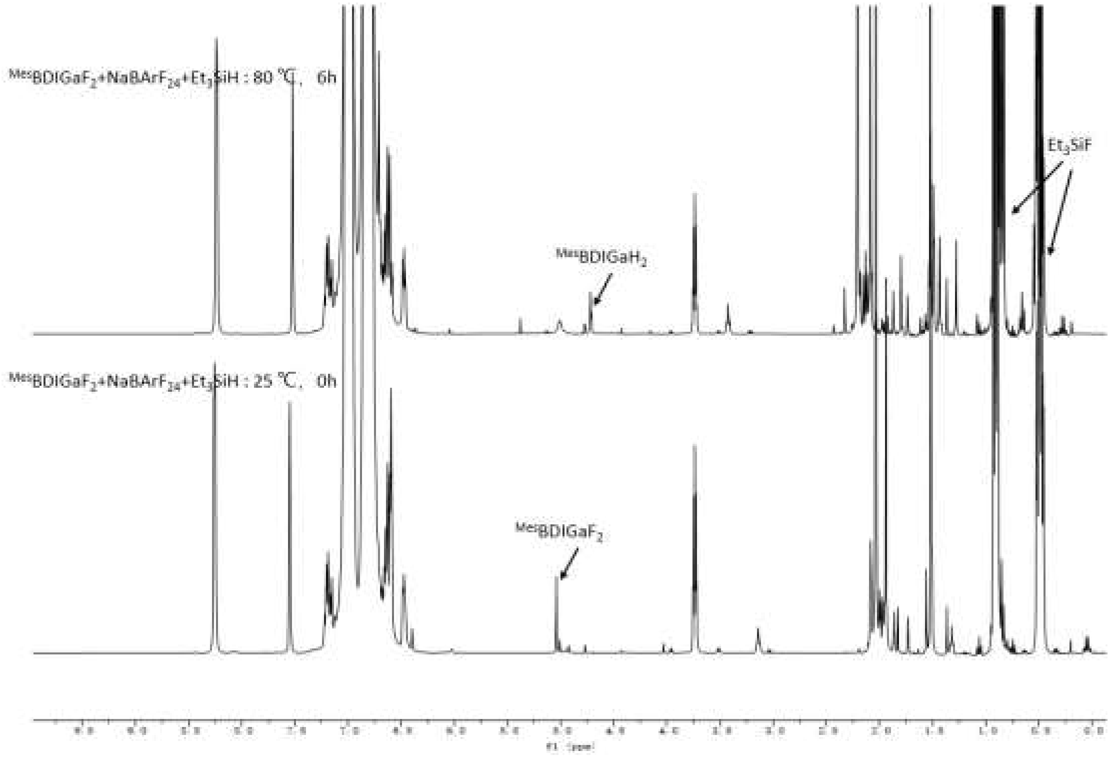

Figure S19. ^{1}H-NMR spectrum of the mixture of ^{Mes}BDIGaF$_2$ + NaBArF$_{24}$ + Et$_3$SiH (1:1:3) in dry PhF

Et$_3$SiF: (in very close agreement with literature reports)[6]
^{19}F-NMR (376.5 MHz, in PhF (standard capillary: 1,2-difluorobenzene): δ -176.9 (m, 1F).

^{Mes}BDIGaF$_2$·NaBArF$_{24}$ (1-Ga·NaBArF$_{24}$)
^{19}F-NMR (376.5 MHz, in PhF (standard capillary: 1,2-difluorobenzene): δ -203.5 (s, 2F).

5.2.4. Stoichiometric reaction of $^{Mes}BDIGaF_2$ (1-Ga) with $^{Mes}BDIGaH_2$ (7-Ga)

Procedure: In the glovebox, $^{Mes}BDIGaF_2$ (**1-Ga**, 5.0 mg, 0.011 mmol), $^{Mes}BDIGaH_2$ (**7-Al,** 22.9 mg, 0.056 mmol) were weighed in a vial, dissolved in 0.6ml PhF and the resulting reaction mixture transferred to a J. Young NMR tube. $^{Mes}BDIGaHF$ (**6-Ga**) was observed by ^{19}F-NMR spectroscopy by a broad doublet at -182.0 ppm, which is similar to the reported literature value $^{Mes}BDIAlHF$.[2] The NMR spectra are shown in Figure S20.

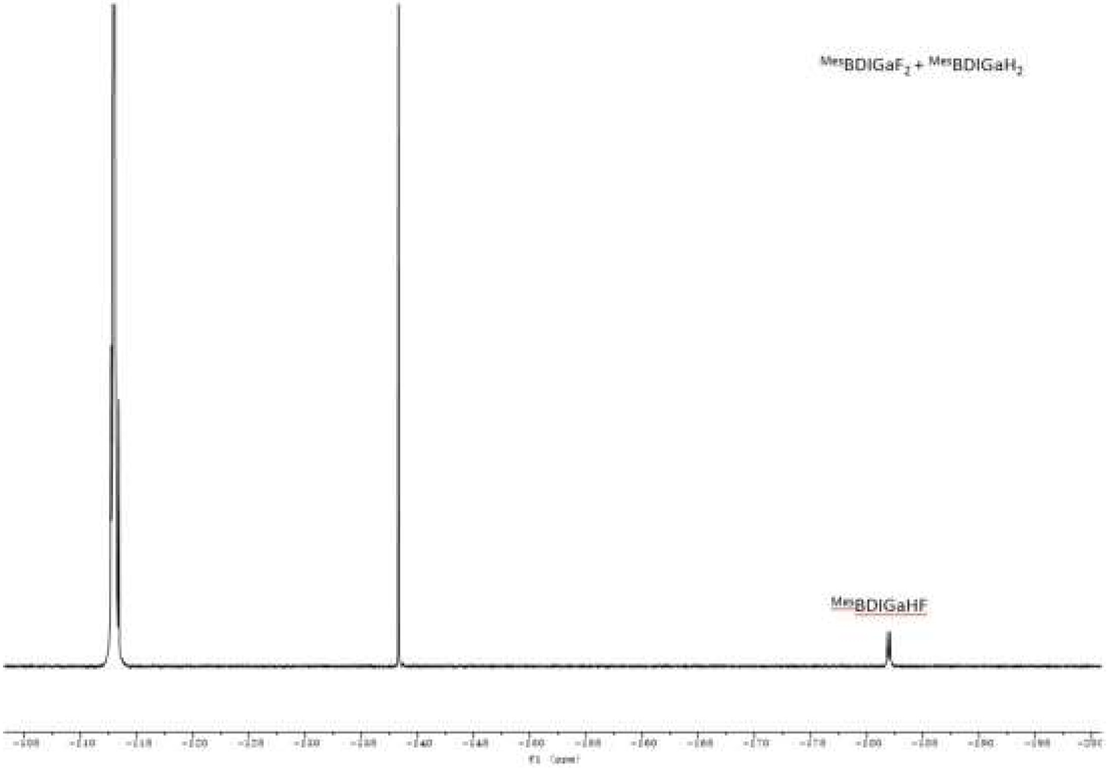

Figure S20. ^{19}F-NMR spectrum of the mixture of $^{Mes}BDIGaF_2$ + $^{Mes}BDIGaH_2$ (1:5) in dry PhF

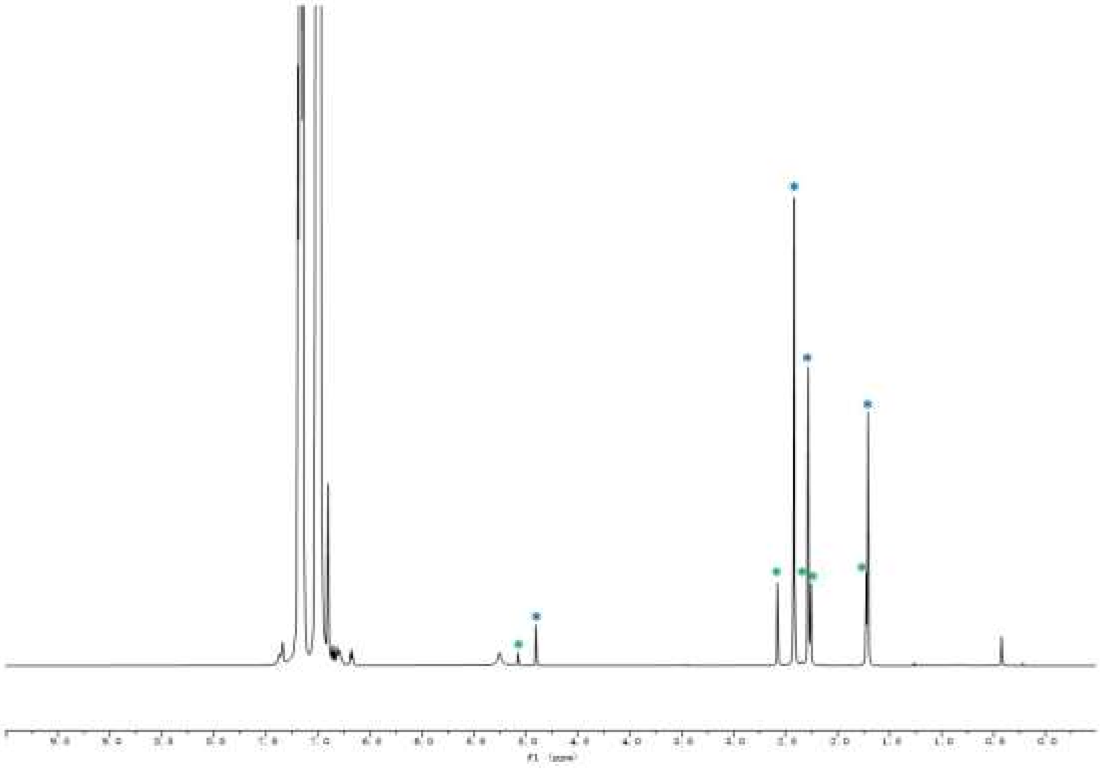

Figure S21. ^{1}H-NMR spectrum of the mixture of ^{Mes}BDIGaF$_2$ + ^{Mes}BDIGaH$_2$ (1:5) in dry PhF(“*”is the peak of (**^{Mes}BDIGaH$_2$**), isolated yield is 40%; “*”is the peak of (**^{Mes}BDIGaHF**)

^{Mes}BDIGaHF (6-Ga)

^{19}F-NMR (376.5 MHz, in PhF (standard capillary: 1,2-difluorobenzene): δ -182.0 (d, $J_{H\text{-}F}$ = 83.1 Hz, 1F)

5.2.5. Stoichiometric reaction of $^{Mes}BDIGaH_2$ with pentafluoropyridine

Procedure: In the glovebox, $^{Mes}BDIGaH_2$ (**7-Ga**, 5.0 mg, 0.012 mmol) and pentafluoropyridine (4.2 mg, 0.025 mmol) were weighed in a vial, dissolved in 1ml PhF and placed in a J. Young NMR tube. The reaction mixture was heated at 100 °C for 18 hours and monitored by ^{19}F-NMR spectroscopy. The NMR spectra are shown in Figure S22.

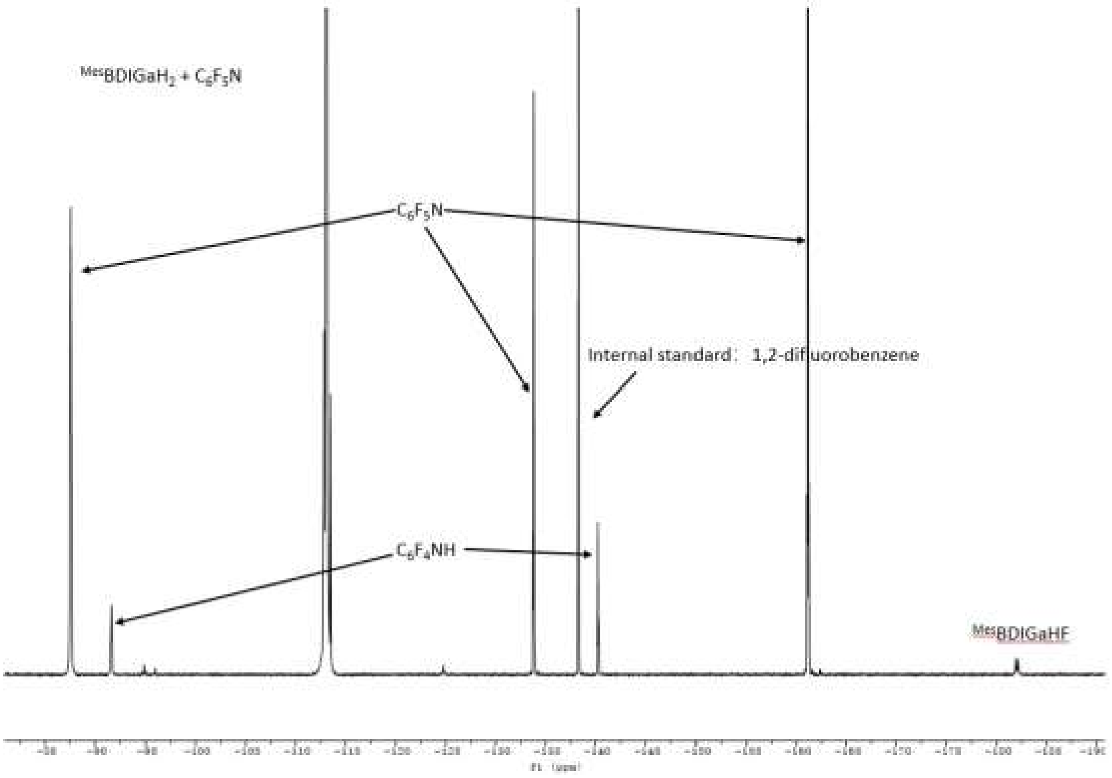

Figure S22. ^{19}F-NMR spectrum of the mixture of $^{Mes}BDIGaH_2 + C_5F_5N$ (1:2) in dry PhF

5.2.6. $^{Mes}BDIMH_2$ as the catalyst in HDF

- HDF of pentafluoropyridine

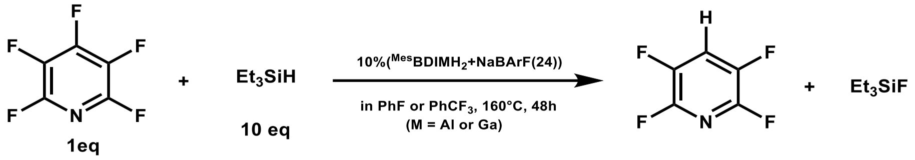

Procedure: In a glovebox, pentafluoropyridine (18.7 mg, 0.11 mmol), Et_3SiH (129.0 mg, 1.1 mmol, 10.0 equiv.), $^{Mes}BDIMH_2$ (**1-M**, 10.0 µmol, 10 mol%) and $NaBArF_{24}$ (9.8 mg, 10.0 µmol, 10 mol%) and PhF were added to a J-Young's NMR tube and sealed. The total volume of PhF and C_6D_6 (5:1) solution was 0.6 ml. After heating at 160 °C for 48 h, 1,2-difluorobenzene in a sealed glass capillary containing C_6D_6 (δ = 138.3 ppm) was added as internal standard and the reaction mixture was analyzed by quantitative ^{19}F-NMR spectroscopy. 2,3,5,6-tetrafluoropyridine (**2a**) was formed as shown in the table below.

Catalyst	Mass (mg)	Temp (°C)	2a NMR yield (%)
$^{Mes}BDIAlH_2$	4.0	160	99
$^{Mes}BDIGaH_2$	4.5	160	99

The spectral data matched with those reported in the literature.[5]

5.2.7. Stepwise Stoichiometric reactions

Procedure one: In the glovebox, the catalytic mechanism was supported using a step-by-step reaction sequence.

Step one: ^{Mes}BDIAlH$_2$ (**1-Al**, 45.5 mg, 0.125 mmol, 5 eq), ^{Mes}BDIAlF$_2$ (10.0 mg, 0.025 mmol, 1 eq) were weighed in a vial and dissolved in 1ml PhF. The mixture was transferred to a J. Young NMR tube. The reaction mixture was monitored by ^{19}F-NMR spectroscopy at 25 °C. The NMR spectra are shown in Figure S22.
Step two: NaBArF$_{24}$ (22.3 mg, 0.025 mmol, 1eq) was weighed in a vial and dissolved in the mixture from step one. The reaction mixture was monitored by ^{19}F-NMR spectroscopy at 25 °C. The NMR spectra are shown in Figure S22.
Step three: Et$_3$SiH (29.1 mg, 0.25 mmol, 10.0 equiv.) was weighed and transferred to the J. Young NMR tube of step two. The reaction mixture was monitored by ^{19}F-NMR spectroscopy at 25 °C. After that, the reaction was heated to 60 °C for 2h and then up to 80 °C for another 2 h. The NMR spectra are shown in Figure S22.
Step four: pentafluoropyridine (4.2 mg, 0.025 mmol, 1eq) was transferred to the J. Young NMR tube of step three. The reaction mixture was monitored by ^{19}F-NMR spectroscopy at 100 °C for 18h and then up to 160 °C for another 14 h. The NMR spectra are shown in Figure S23.

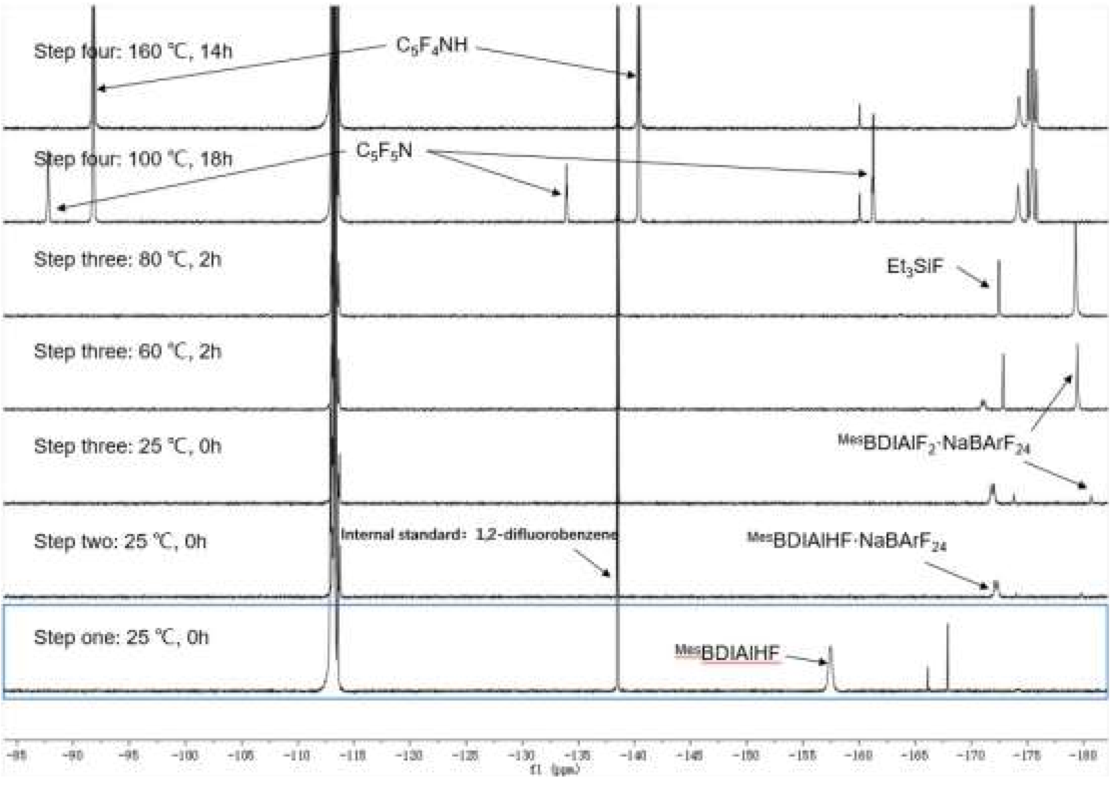

Figure S23. ^{19}F-NMR spectrum of Stoichiometric reaction of the pass way of the catalytic mechanism (Al).

^{Mes}BDIAlHF·NaBArF$_{24}$:
^{19}F-NMR (376.5 MHz, in PhF (standard capillary: PhF (113.3 ppm in C$_6$D$_6$))): δ -62.4 (s, 24F), -172.2 (s, 1F, J = 91.1 Hz).

Procedure two: In the glovebox, the catalytic mechanism was supported using a step-by-step reaction sequence.

Step one: ^{Mes}BDIGaH$_2$ (50.1 mg, 0.125 mmol, 5 eq), ^{Mes}BDIGaF$_2$ (11.0 mg, 0.025 mmol, 1 eq) were weighed in a vial and dissolved in 1ml PhF. The mixture was transferred to a J. Young NMR tube. The reaction mixture was monitored by ^{19}F-NMR spectroscopy at 25 °C. The NMR spectra are shown in Figure S23.
Step two: NaBArF$_{24}$ (22.3 mg, 0.025 mmol, 1eq) was weighed in a vial and dissolved in the mixture from step one. The reaction mixture was monitored by ^{19}F-NMR spectroscopy at 25 °C. The NMR spectra are shown in Figure S23.
Step three: Et$_3$SiH (29.1 mg, 0.25 mmol, 10.0 equiv.) was weighed and transferred to the J. Young NMR tube from step two. The reaction mixture was monitored by ^{19}F-NMR spectroscopy at 25 °C. After that, the reaction was heated to 60 °C for 4h and then up to 80 °C for another 12 h. The NMR spectra are shown in Figure S23.
Step four: pentafluoropyridine (4.2 mg, 0.025 mmol, 1eq) was transferred to the J. Young NMR tube of step three. The reaction mixture was monitored by ^{19}F-NMR spectroscopy at 100 °C for 3h and then up to 150 °C for another 3 h. The NMR spectra are shown in Figure S24.

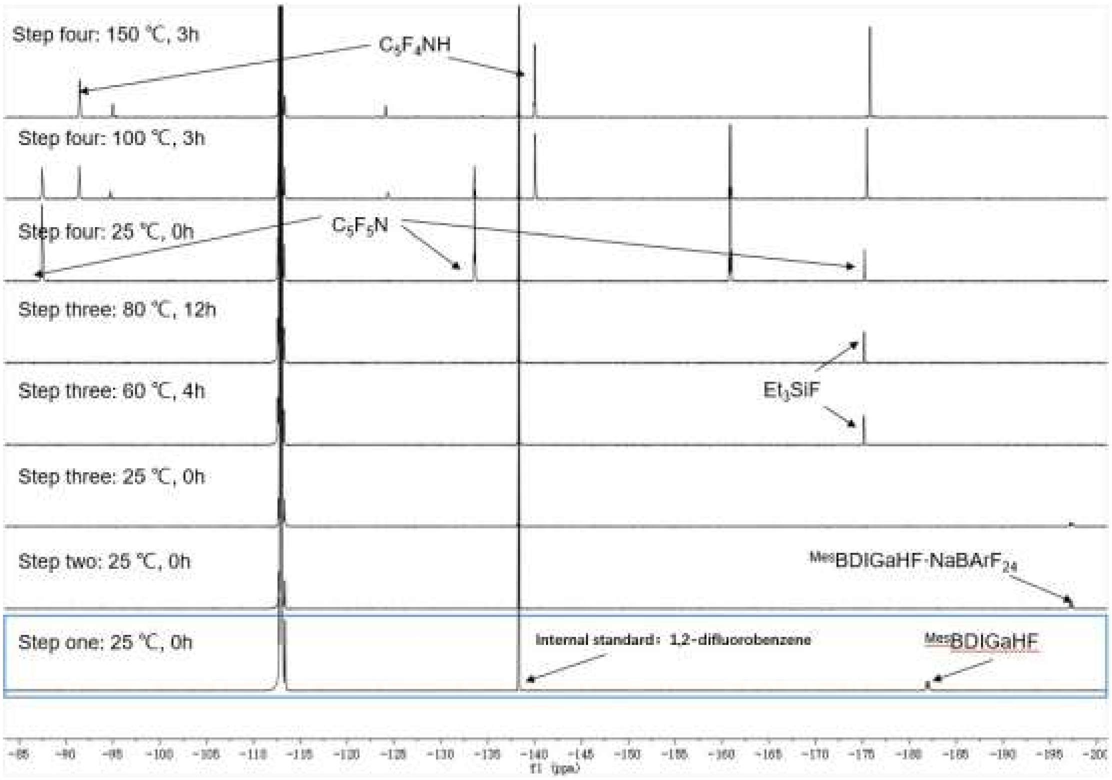

Figure S24. ^{19}F-NMR spectrum of Stoichiometric reaction of the pass way of the catalytic mechanism (Ga).

^{Mes}BDIGaHF
^{19}F-NMR (376.5 MHz, in PhF (standard capillary: PhF (113.3 ppm in C$_6$D$_6$))): δ -182.0 (s, 1F, J = 91.1 Hz).

^{Mes}BDIGaHF·NaBArF$_{24}$:
^{19}F-NMR (376.5 MHz, in PhF (standard capillary: PhF (113.3 ppm in C$_6$D$_6$))): δ -62.2 (s, 24F), -197.3 (s, 1F, J = 102.6 Hz).

6) DFT Studies

6.1. Computational methods

DFT calculations were performed using Gaussian 09 (Revision D.01) using an ultrafine integration grid (int=ultrafine).[S10] Geometry optimisations and frequency calculations were performed using the ωB97xD density functional including solvent corrections (PCM, fluorobenzene, ε = 5.42) with SDDAll (Na, Ga), 6-31G** (C, H) and 6-311+G* (N, F, Si) basis set. Frequency analyses for all stationary points were performed using the enhanced criteria to confirm the nature of the structures as either minima (no imaginary frequency) or transition states (only one imaginary frequency). The electronic energies of the optimised geometries were calculated using the ωB97xD functional with def2TZVPP basis sets for all atoms with solvent corrections (PCM, fluorobenzene, ε = 2.2706). The Gibbs free energy correction from the frequency calculation was added to this electronic energy to generate Gibbs free energy values for the calculated stationary points.

Intrinsic reaction coordinate (IRC) calculations were used to connect transition states and minima located on the potential energy surface allowing a full energy profile (calculated at 298.15 K, 1 atm.) of the reaction to be constructed.

NBO analysis was performed at the ωB97xD/def2TZVPP level using NBO 6.0 and stated as such.[S19] QTAIM analysis was conducted using the AIMAll package.[S20]

6.2. Calculated Reaction Pathway: Hydrodefluorination

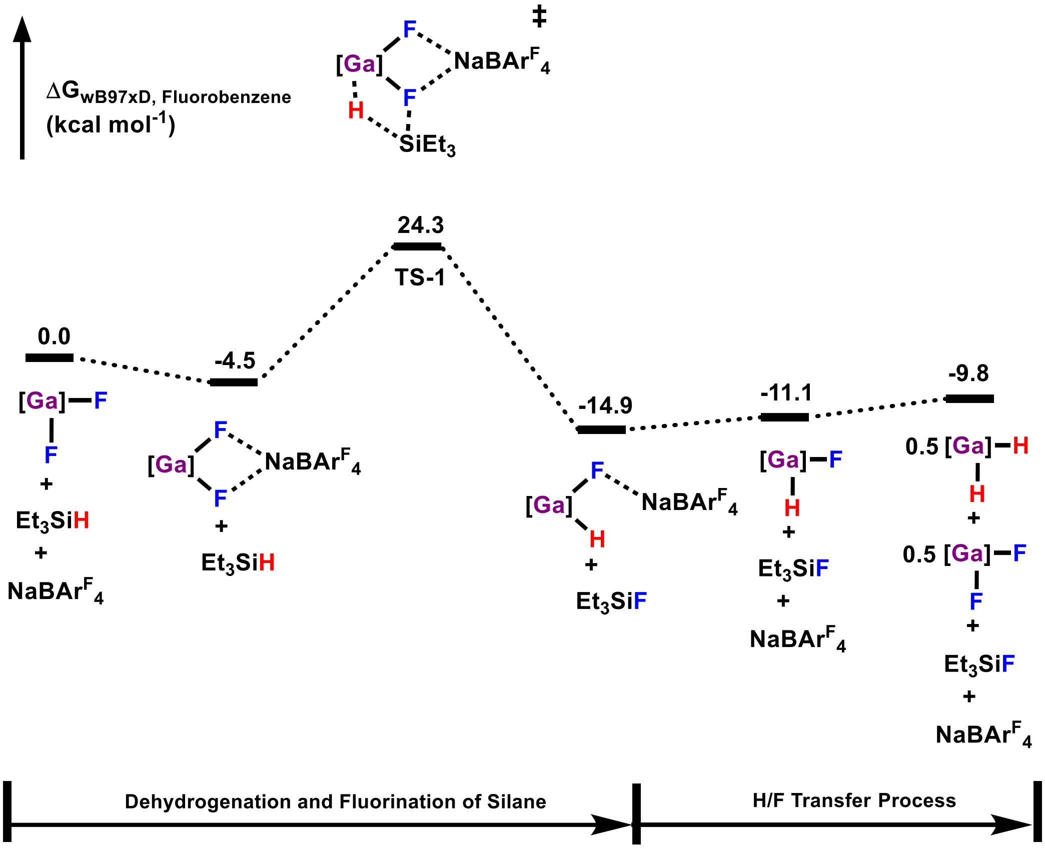

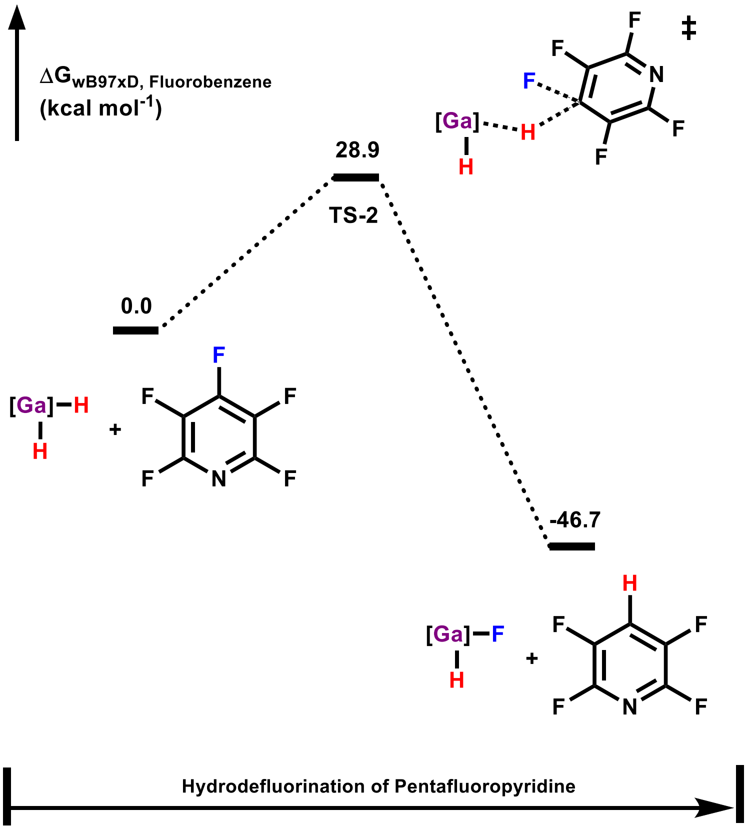

Figure S25. Proposed pathway for hydrodefluorination based on DFT calculations G09: wB97xD /6-311+G**/ PCM (fluorobenzene) // wB97xD / 6-31G** / 6-311+G* (N, F, Si)/ SDDAll (Na, Ga)

6.2.1. Calculated Reaction Pathway: Hydrodefluorination process of pentafluoropyridine (Without $NaBAr^F{}_4$)

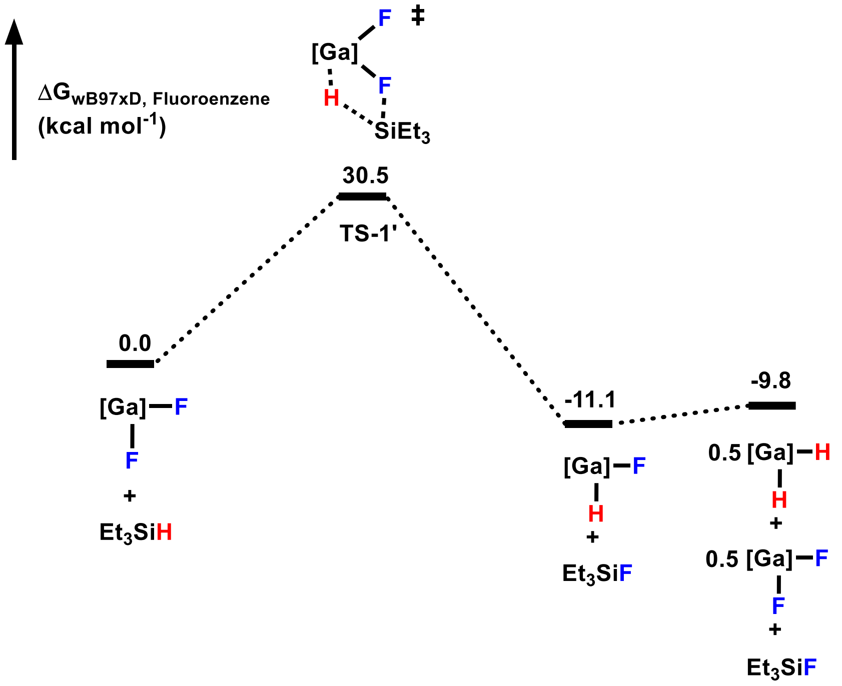

Figure S26. Proposed pathway for hydrodefluorination based on DFT calculations G09: wB97xD /6-311+G**/ PCM (fluorobenzene) // wB97xD / 6-31G** / 6-311+G* (N, F, Si)/ SDDAll (Ga)

6.3. Optimised structures

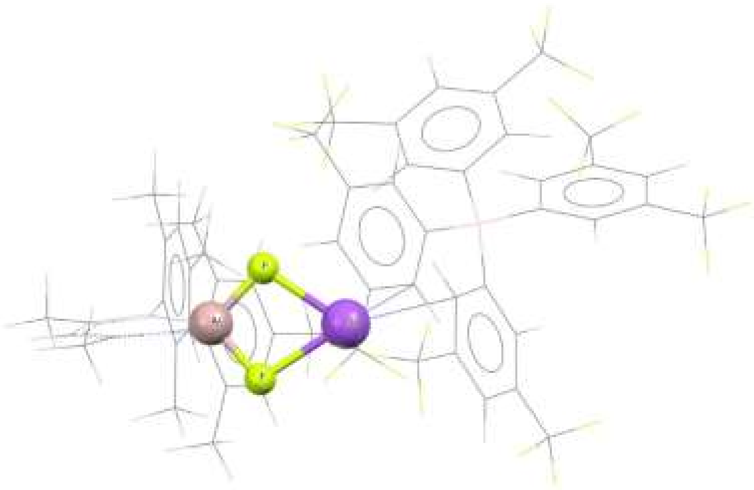

1-Al-NaBArF$_{24}$

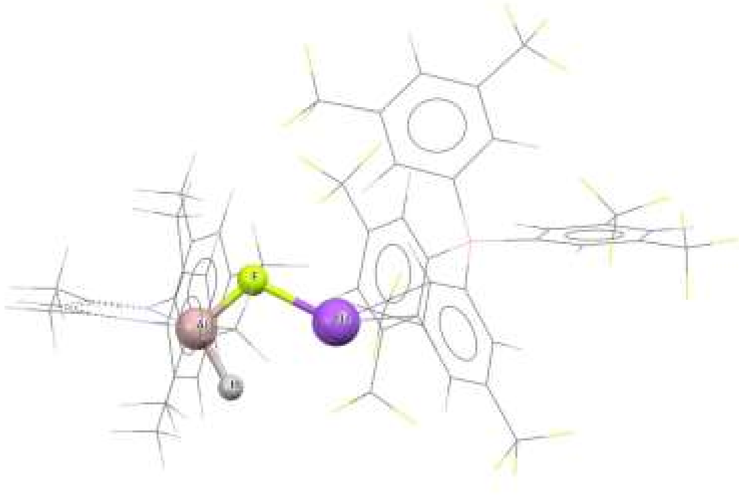

2-Al-NaBArF$_{24}$

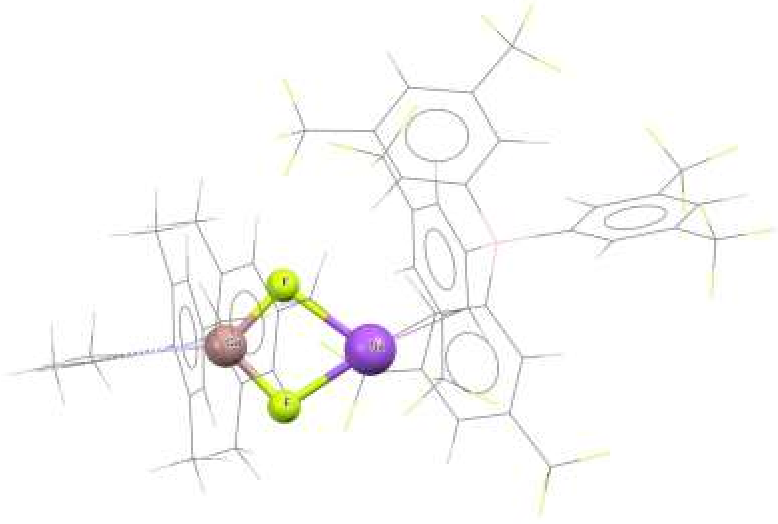

1-Ga-NaBArF$_{24}$

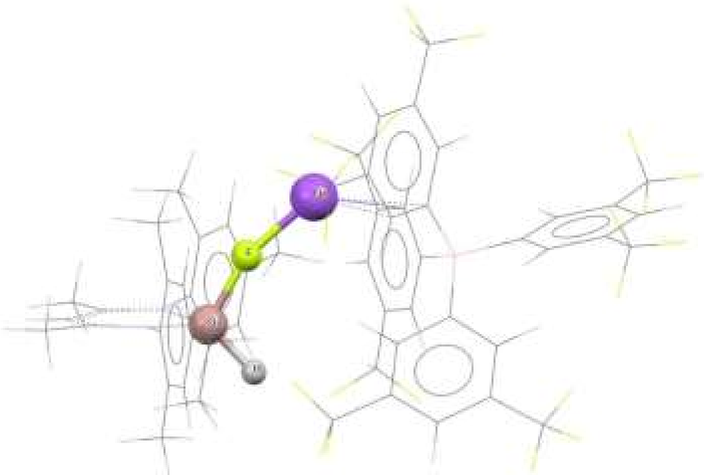

2-Ga-NaBArF$_{24}$

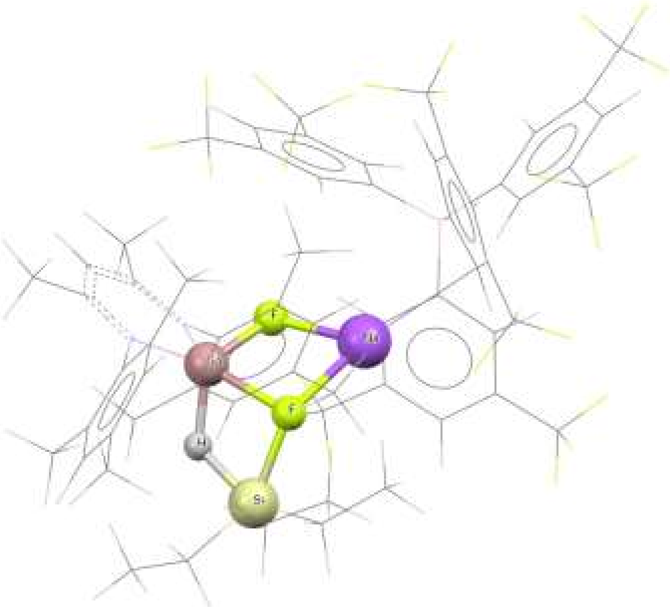

TS1

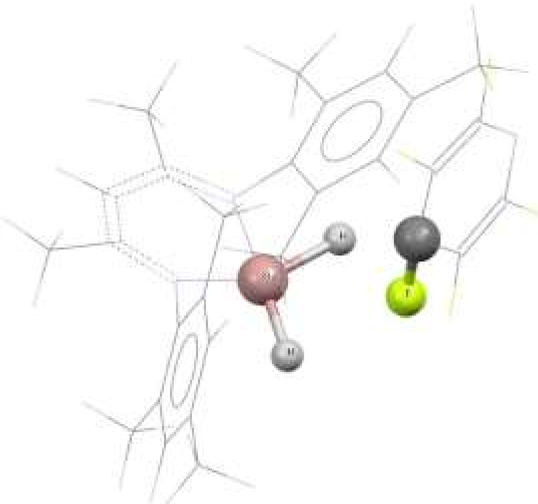

TS2

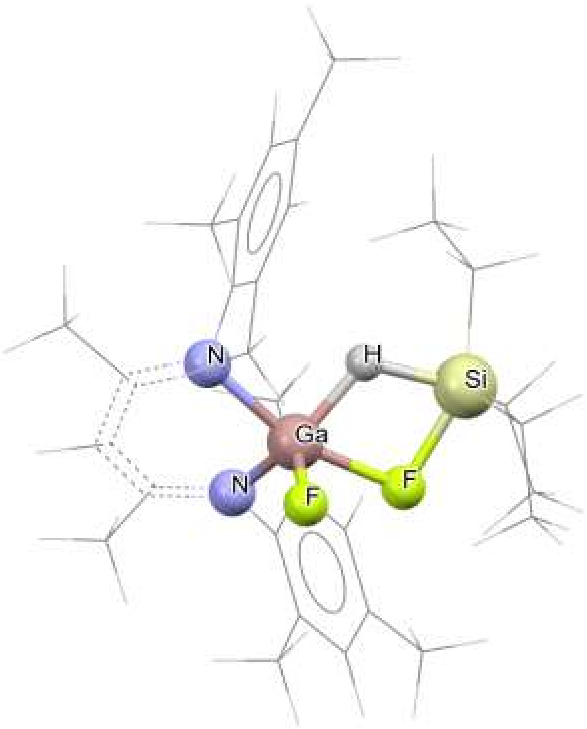

TS1’

6.4 NBO Analysis

NBO and QTAIM calculations were used to better understand the changes of electronic structure comparing **1-Ga-NaBAr$^F_4$** to **1-Ga**. Calculated Ga–F bond lengths in **1-Ga** are ca. 0.03 Å shorter than in **1-Ga-NaBAr$^F_4$**. The NPA charges on the fluoride ligands become more negative when comparing **1-Ga** (–0.67,–0.67) to **1-Ga-NaBAr$^F_4$** (–0.69,–0.70), this is perhaps expected and would be consistent with the binding of Na^+ effecting a polarisation of the Ga–F bonds. Second-order perturbation analysis within the NBO framework reveals donor-acceptor interactions between 2p LP on F and the empty 3s orbital of Na^+.

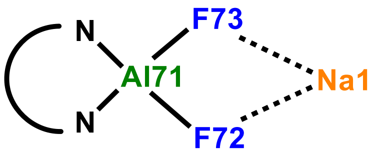

1-Al-NaBArF$_{24}$

Atom	No	Charge
Na1	1	0.95
F72	72	-0.79
F73	73	-0.78
Al71	71	2.13

Table S5. NPA change data for **1-Al-NaBArF$_{24}$**

Wiberg bond index matrix	
Na1 - F72	0.006
Na1 - F73	0.005
Al71 - F72	0.367
Al71 - F73	0.373
Na1 - Al71	0.007

Table S6. WBI data for **1-Al-NaBArF$_{24}$**

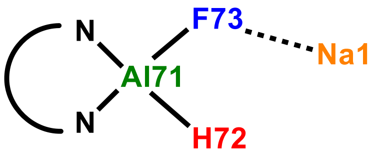

2-Al-NaBArF$_{24}$

Atom	No	Charge
Na1	1	0.94
H72	72	-0.46
F73	73	-0.79
Al71	71	1.82

Table S7. NPA change data for **2-Al-NaBArF$_{24}$**

Wiberg bond index matrix	
Na1 - H72	0.008
Na1 - F73	0.006
Al71 - H72	0.747
Al71 - F73	0.345
Na1 - Al71	0.008

Table S8. WBI data for **2-Al-NaBArF$_{24}$**

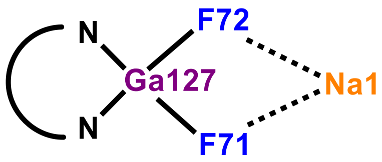

1-Ga-NaBArF$_{24}$

Atom	No	Charge
Na1	1	0.94
F71	71	-0.78
F72	72	-0.77
Ga127	127	2.04

Table S9. NPA change data for **1-Ga-NaBArF$_{24}$**

Wiberg bond index matrix	
Na1 - F71	0.006
Na1 - F72	0.005
Ga127 - F71	0.357
Ga127 - F72	0.359
Na1 - Ga127	0.009

Table S10. WBI data for **1-Ga-NaBArF$_{24}$**

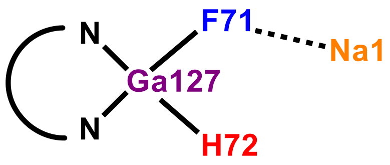

2-Ga-NaBArF$_{24}$

Atom	No	Charge
Na1	1	0.95
F71	71	-0.81
H72	72	-0.33
Ga127	127	1.63

Table S11. NPA change data for **2-Ga-NaBArF$_{24}$**

Wiberg bond index matrix	
Na1 - F71	0.004
Na1 - H72	0.003
Ga127 - F71	0.309
Ga127 - H72	0.841
Na1 - Ga127	0.005

Table S12. WBI data for **2-Ga-NaBArF$_{24}$**

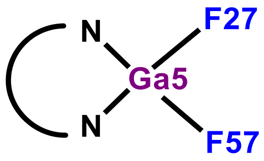

1-Ga

Atom	No	Charge
Ga5	5	2.03
F27	27	-0.75
F57	57	-0.75

Table S13. NPA change data for **1-Ga**

Wiberg bond index matrix	
Ga5–F27	0.415
Ga5–F57	0.415

Table S14. WBI data for **1-Ga**

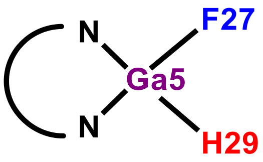

2-Ga

Atom	No	Charge
Ga5	5	1.62
F27	27	-0.76
H29	29	-0.34

Table S15. NPA change data for **2-Ga**

Wiberg bond index matrix	
Ga5–F27	0.393
Ga5–H29	0.836

Table S16. WBI data for **2-Ga**

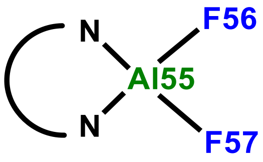

1-Al

Atom	No	Charge
Al55	55	2.12
F56	56	-0.76
F57	57	-0.76

Table S17. NPA change data for **1-Al**

Wiberg bond index matrix	
Al55–F56	0.407
Al55–F57	0.408

Table S18. WBI data for **1-Ga**

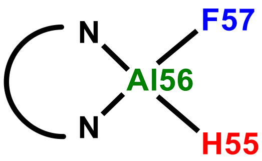

2-Al

Atom	No	Charge
Al56	56	1.80
H55	55	-0.45
F57	57	-0.77

Table S19. NPA change data for **2-Ga**

Wiberg bond index matrix	
Al56–H55	0.766
Al55–F57	0.390

Table S20. WBI data for **2-Al**

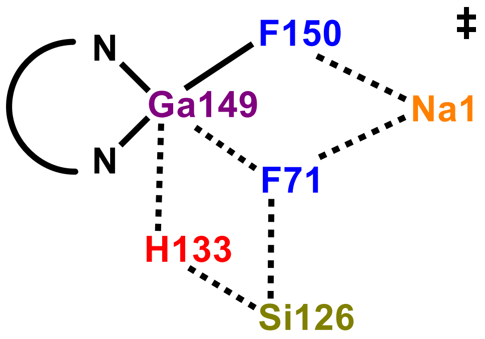

TS1

Atom	No	Charge
Na1	1	0.92
F71	71	-0.75
F150	150	-0.79
Ga149	149	1.92
H133	133	-0.39
Si126	126	1.83

Table S21. NPA change data for **TS1**

Wiberg bond index matrix	
Na1 - F71	0.005
Na1 - F150	0.005
Ga149 - F71	0.164
Ga149 - F150	0.808
Ga149 - H133	0.333
Si126 - F71	0.212
Si126 - H133	0.419

Table S22. WBI data for **TS1**

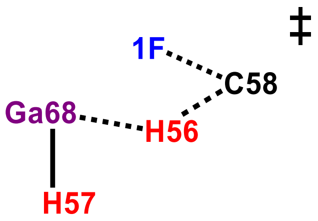

TS2

Atom	No	Charge
F1	1	-0.33
H56	71	-0.20
H57	72	-0.30
C58	58	0.28
Ga68	68	1.36

Table S23. NPA change data for **TS2**

Wiberg bond index matrix	
Ga68 - H56	0.430
Ga68 - H57	0.840
Ga68 - F1	0.418
H56 - C58	0.391
F1 - C58	0.857

Table S24. WBI data for **TS2**

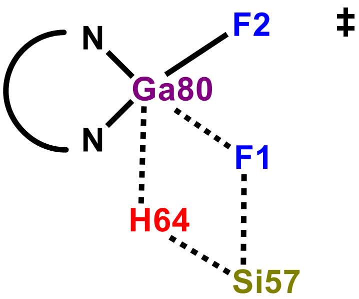

TS1’

Atom	No	Charge
F1	1	-0.33
H56	71	-0.20
H57	72	-0.30
C58	58	0.28
Ga68	68	1.36

Table S25. NPA change data for **TS2**

Wiberg bond index matrix	
Ga68 - H56	0.430
Ga68 - H57	0.840
Ga68 - F1	0.418
H56 - C58	0.391
F1 - C58	0.857

Table S26. WBI data for **TS2**

6.5 QTAIM Analysis

QTAIM calculations return bond paths between Ga and F atoms along with F and Na atoms in **1-Ga-NaBAr^F$_4$**. Comparison of the r(bcp) values for bond critical points connecting Ga and F between **1-Ga** (0.11 and 0.11 e bohr^{-3}) and **1-Ga-NaBAr^F$_4$** (0.10 and 0.11 e bohr^{-3}) is consistent with similar electron-density between these atoms on coordination. r(r) and ∇^2r(r) values for bond critical points between Na and F atoms in **1-Ga-NaBAr^F$_4$** support the idea that the binding interaction is primarily electrostatic (Figure 6c). Based on the analysis, it is possible that the increased $^2J_{H\text{–}F}$ coupling constant for **6-Ga-NaBAr^F$_4$** compared to **6-Ga** might be due to a lower p-character (and hence increased s-character) for the main group fluoride bond in the Ga–F---Na interaction compared with Ga–F.

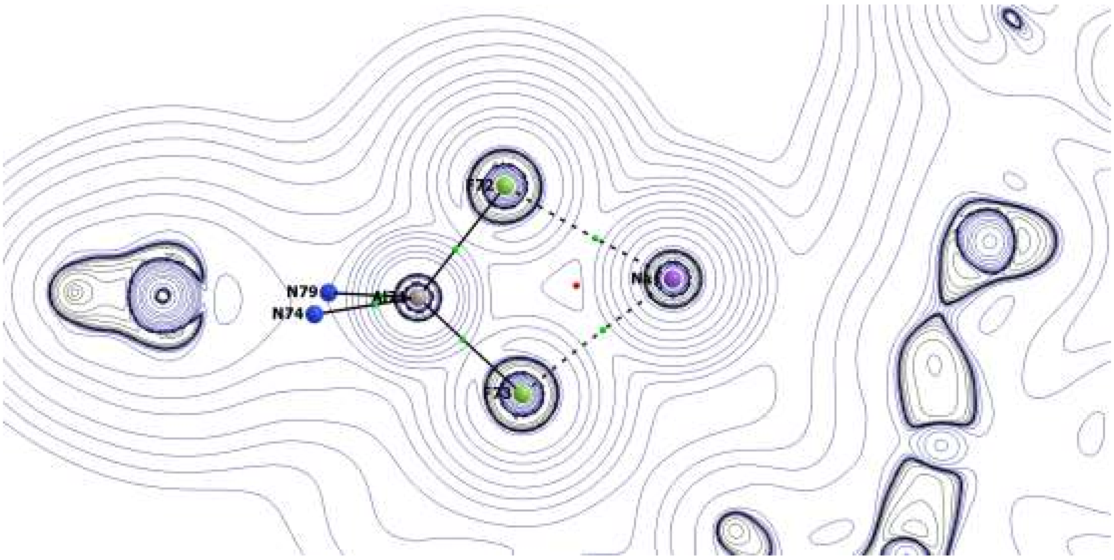

Figure S27. QTAIM molecular graphs for **1-Al-NaBArF$_{24}$**

Atoms	r (r) (e bohr^{-3})	∇^2 r (r) (e bohr^{-5})	Ellipticity
Na1 - F73	0.016	0.123	0.070
Na1 - F72	0.016	0.120	0.074
Al71 - N79	0.090	0.460	0.118
Al71 - N74	0.090	0.459	0.117
Al71 - F73	0.089	0.679	0.005
Al71 - F72	0.088	0.669	0.004

Table S27. QTAIM data for **1-Al-NaBArF$_{24}$**

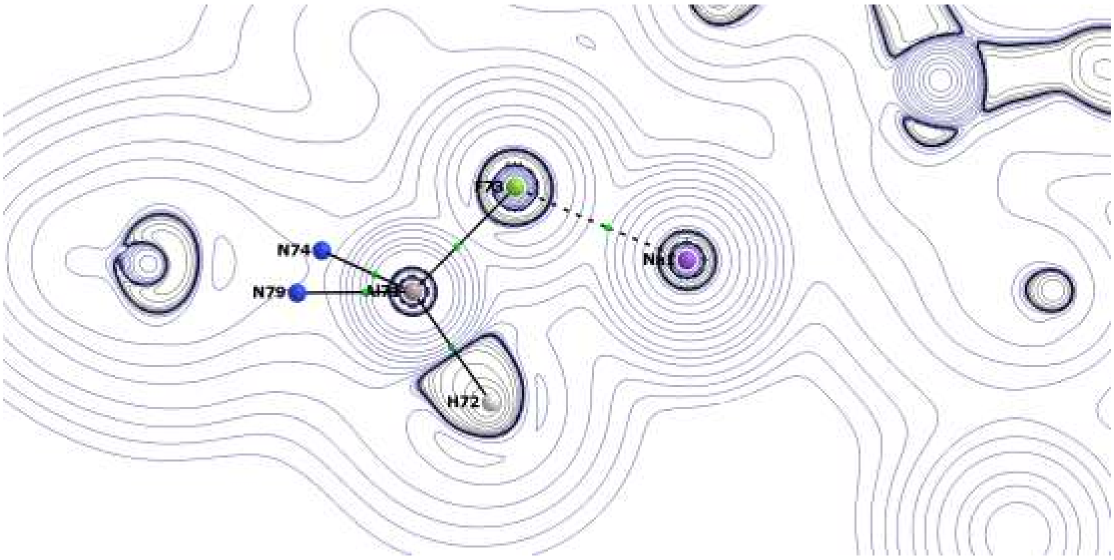

Figure S28. QTAIM molecular graphs for **2-Al-NaBArF$_{24}$**

Atoms	r (r) (e bohr^{-3})	∇^2 r (r) (e bohr^{-5})	Ellipticity
Na1 - F73	0.023	0.178	0.004
Al71 - N79	0.083	0.417	0.109
Al71 - N74	0.084	0.420	0.109
Al71 - H72	0.081	0.213	0.004
Al71 - F73	0.083	0.626	0.002

Table S28. QTAIM data for **2-Al-NaBArF$_{24}$**

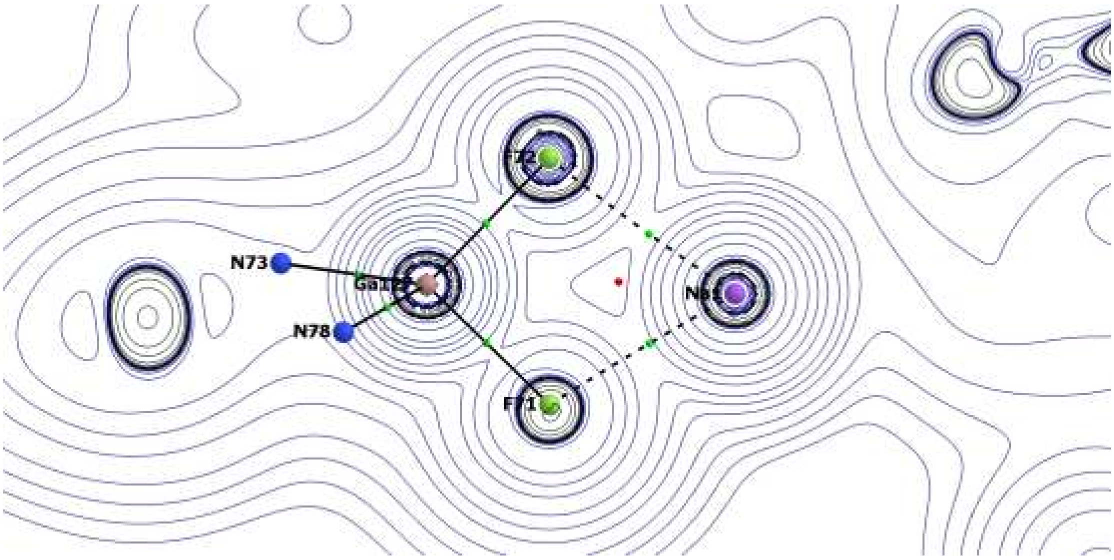

Figure S29. QTAIM molecular graphs for **1-Ga-NaBArF$_{24}$**

Atoms	r (r) (e bohr^{-3})	∇^2 r (r) (e bohr^{-5})	Ellipticity
F71 - Ga127	0.105	0.627	0.013
F72 - Ga127	0.110	0.664	0.013
Na1 - F71	0.020	0.150	0.016
Na1 - F72	0.015	0.106	0.072
N78 - Ga127	0.116	0.446	0.096
N73 - Ga127	0.119	0.450	0.101

Table S29. QTAIM data for **1-Ga-NaBArF$_{24}$**

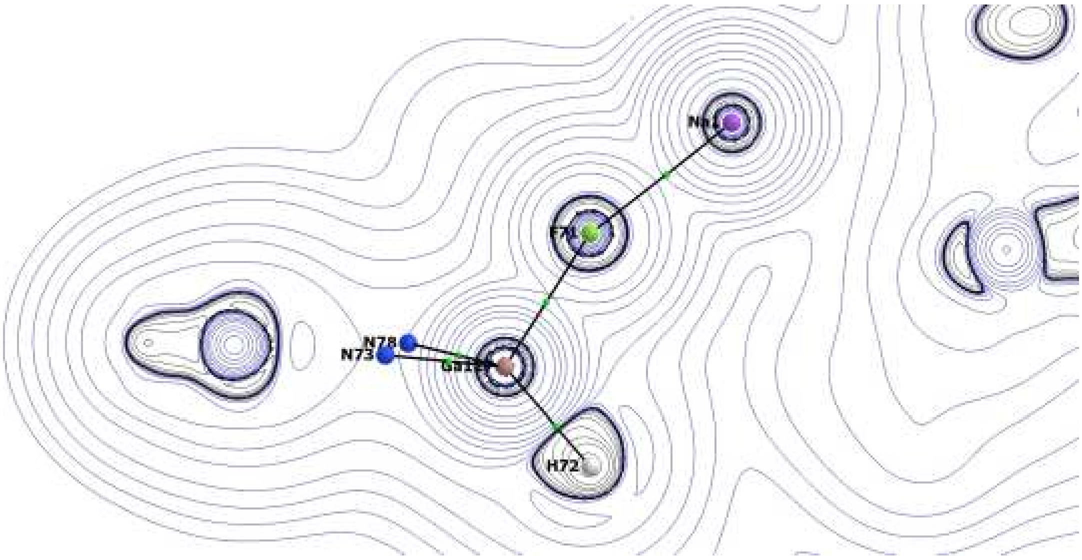

Figure S30. QTAIM molecular graphs for **2-Ga-NaBArF$_{24}$**

Atoms	r (r) (e bohr^{-3})	∇^2 r (r) (e bohr^{-5})	Ellipticity
Na1 - F71	0.028	0.224	0.001
N78 - Ga127	0.103	0.383	0.091
N73 - Ga127	0.103	0.380	0.091
H72 - Ga127	0.121	0.149	0.011
F71 - Ga127	0.091	0.539	0.001

Table S30. QTAIM data for **2-Ga-NaBArF$_{24}$**

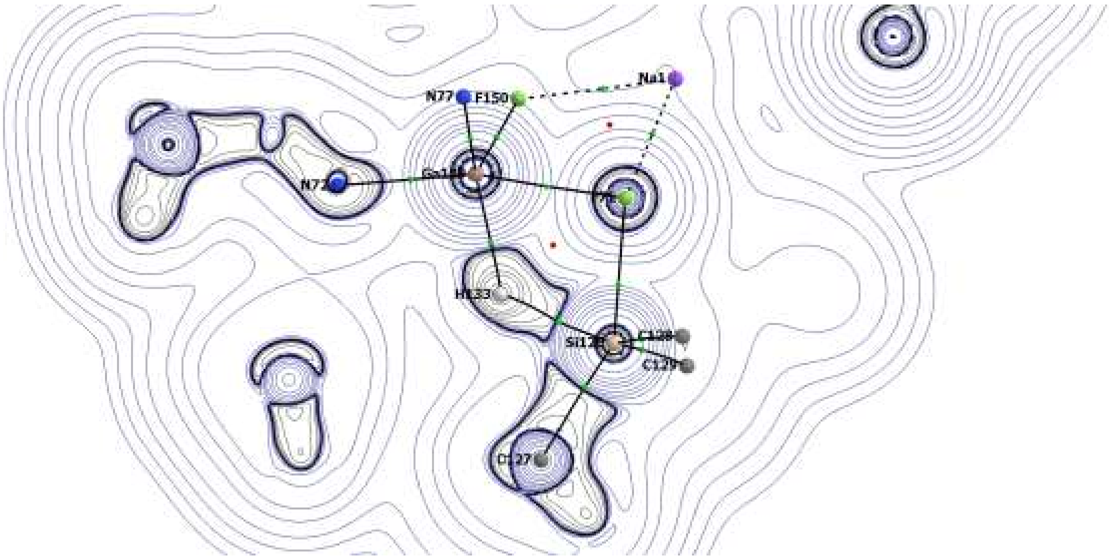

Figure S31. QTAIM molecular graphs for **TS1**

Atoms	r (r) (e bohr^{-3})	∇^2 r (r) (e bohr^{-5})	Ellipticity
F71 - Ga149	0.054	0.246	0.118
F71 - Si126	0.050	0.155	0.158
Ga149 - F150	0.102	0.599	0.016
H133 - Ga149	0.079	0.148	0.016
Na1 - F150	0.023	0.180	0.003
Na1 - F71	0.020	0.154	0.033
Si126 - C127	0.120	0.134	0.021
Si126 - C128	0.126	0.150	0.034
Si126 - C129	0.126	0.153	0.037
Si126 - H133	0.066	0.044	0.501
N77 - Ga149	0.109	0.401	0.108
N72 - Ga149	0.102	0.367	0.082

Table S31. QTAIM data for **TS1**

c

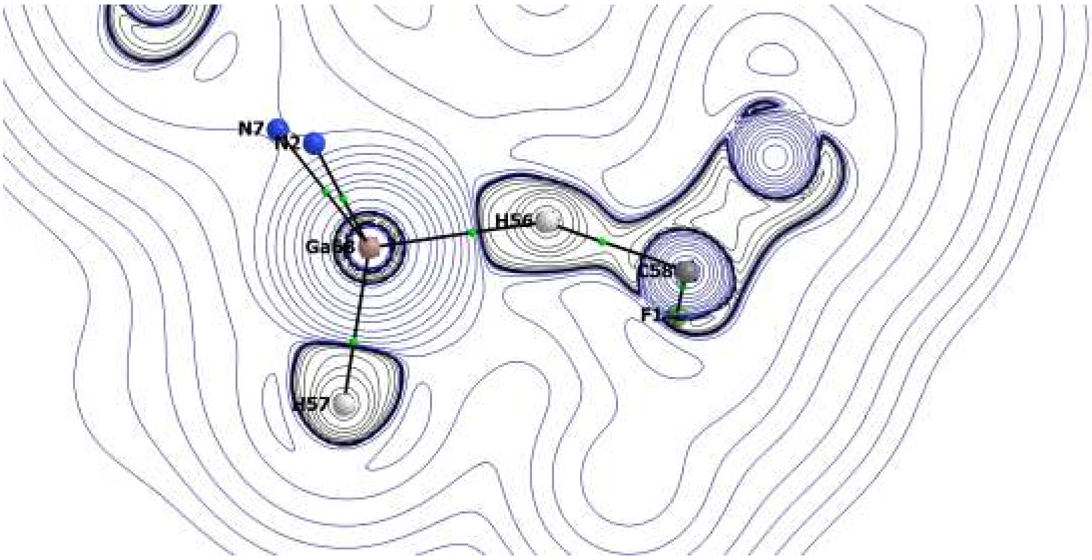

Figure S32. QTAIM molecular graphs for **TS2**

**Atoms**	**r (r) (e bohr^{-3})**	**∇^2 r (r) (e bohr^{-5})**	**Ellipticity**
F1 - C58	0.254	-0.339	0.070
H56 - C58	0.114	-0.113	0.038
H56 - Ga68	0.075	0.095	0.030
H57 - Ga68	0.117	0.148	0.001
N7 - Ga68	0.103	0.387	0.100
N2 - Ga68	0.103	0.391	0.100

Table S32. QTAIM data for **TS2**

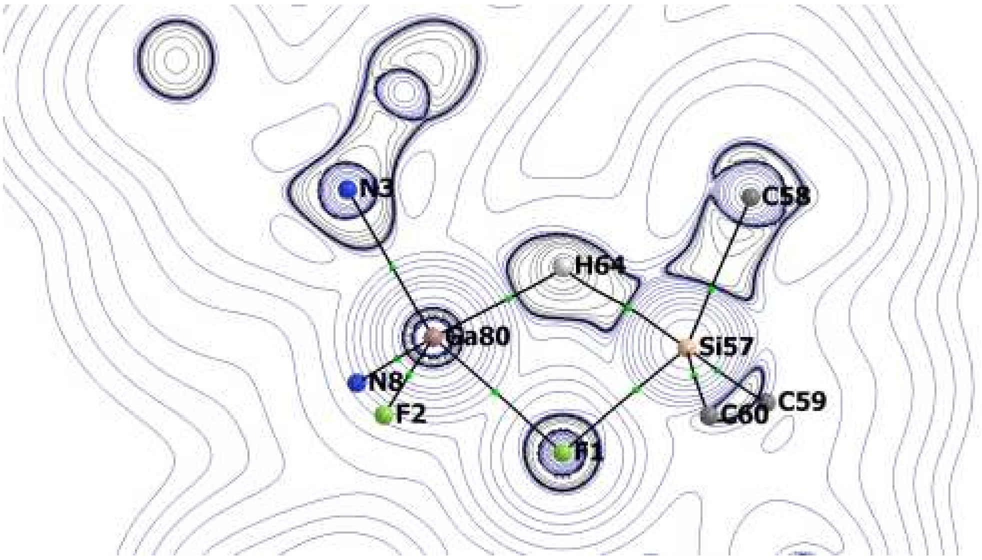

Figure S33. QTAIM molecular graphs for **TS1'**

**Atoms**	**r (r) (e bohr^{-3})**	**∇^2 r (r) (e bohr^{-5})**	**Ellipticity**
Si57 - H64	0.067	0.055	0.543
Si57 - C60	0.125	0.153	0.038
Si57 - C59	0.124	0.150	0.040
Si57 - C58	0.118	0.138	0.022
N8 - Ga80	0.105	0.392	0.103
N3 - Ga80	0.095	0.339	0.075
H64 - Ga80	0.078	0.151	0.031
F2 - Ga80	0.109	0.638	0.014
F1 - Si57	0.061	0.239	0.124
F1 - Ga80	0.066	0.333	0.064

Table S33. QTAIM data for **TS1'**

7) NMR Spectra

7.1. NMR Spectra of Group 13 complexes and the Precursors

$^{Mes}BDIBF_2$：

^1H NMR ($CDCl_3$, 400 MHz)

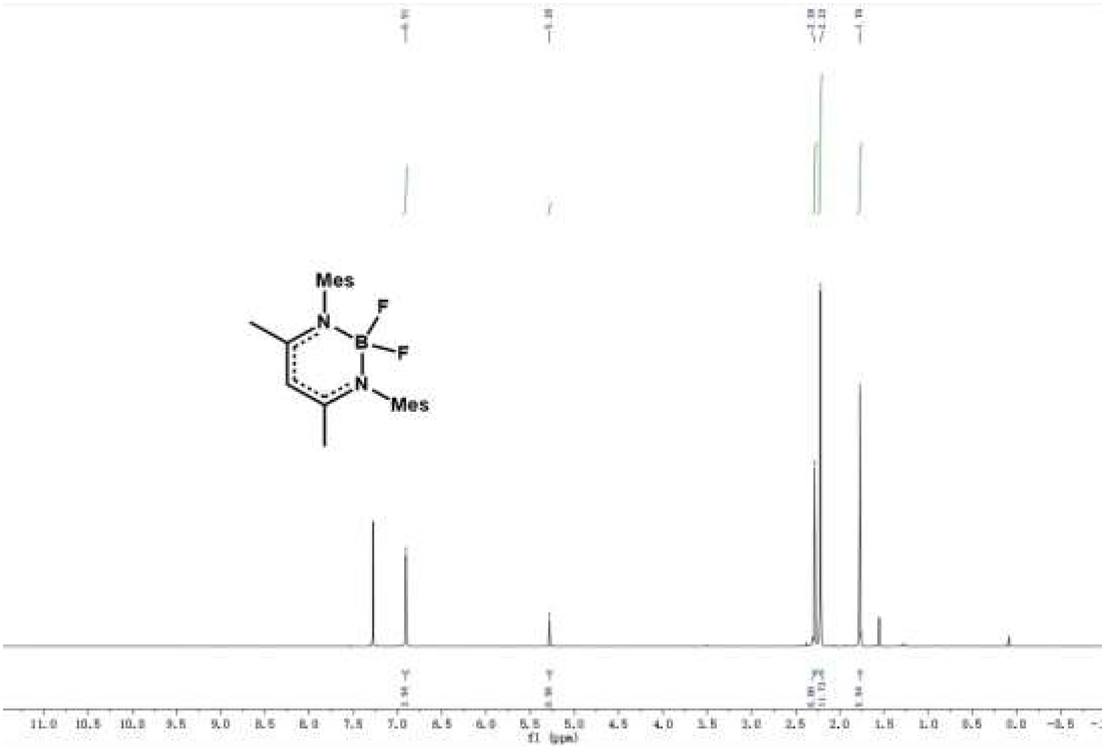

^{19}F NMR ($CDCl_3$, 376.5 MHz

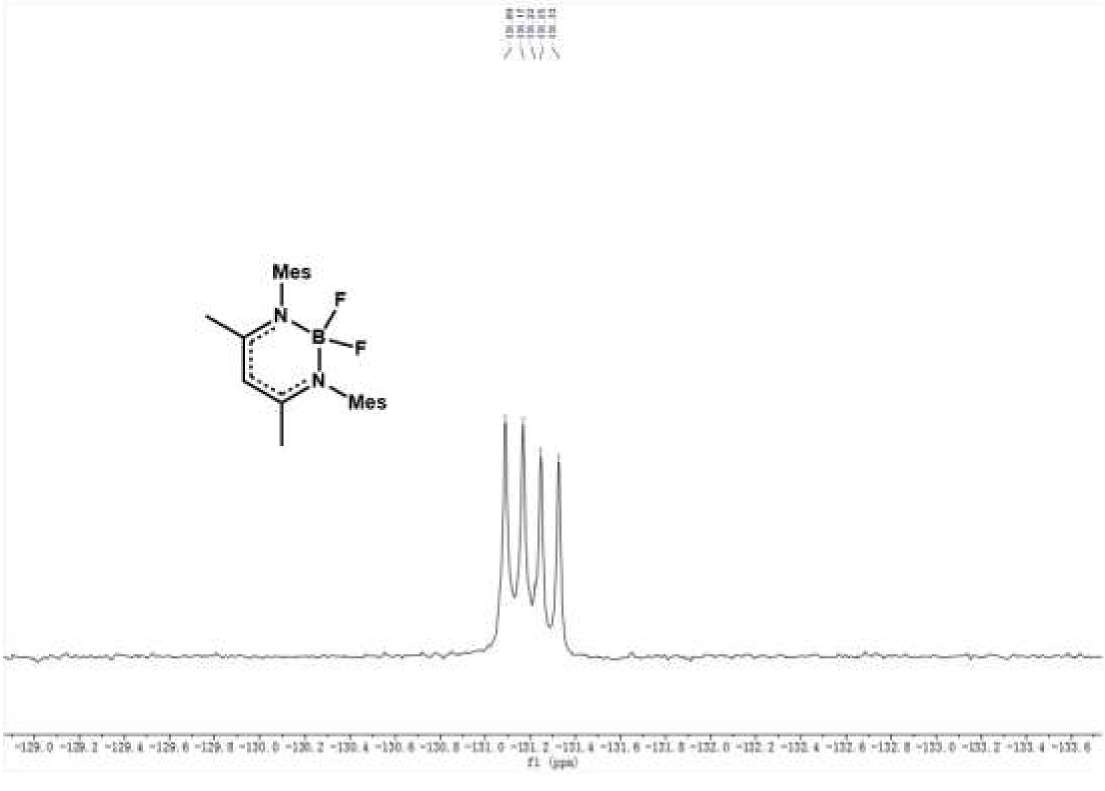

^{11}B NMR ($CDCl_3$, 128.0MHz)

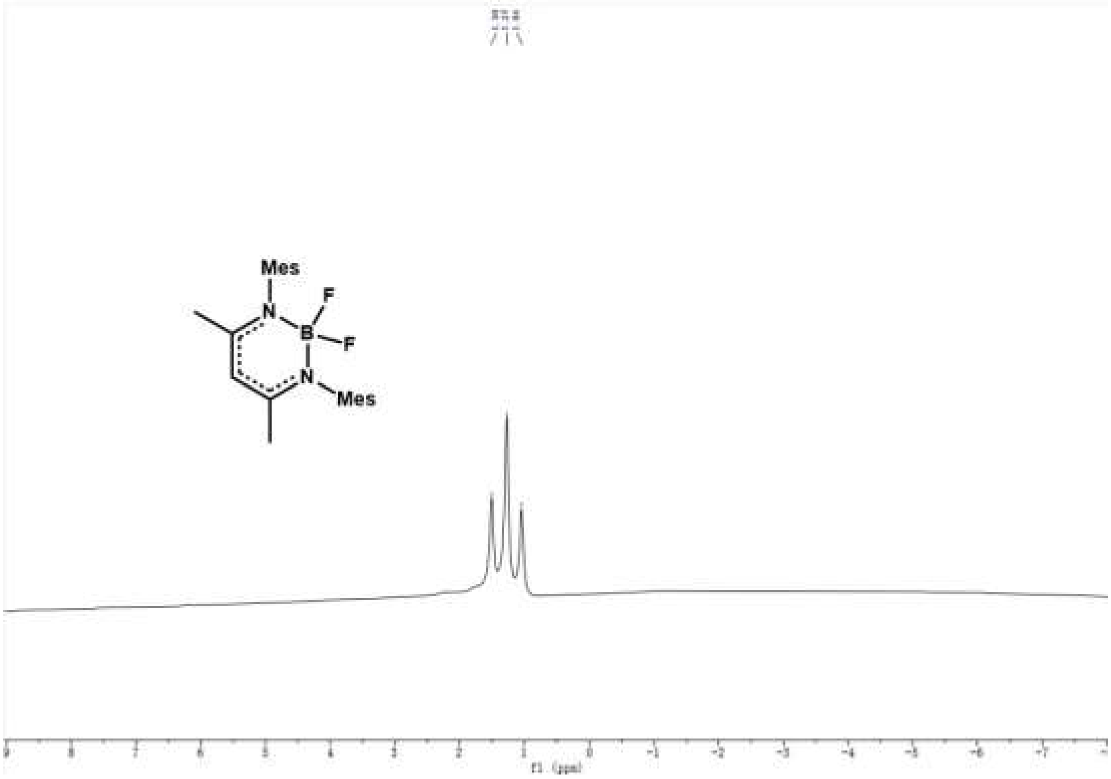

$^{Mes}BDIAlH_2$：

^1H NMR (C_6D_6, 400 MHz)

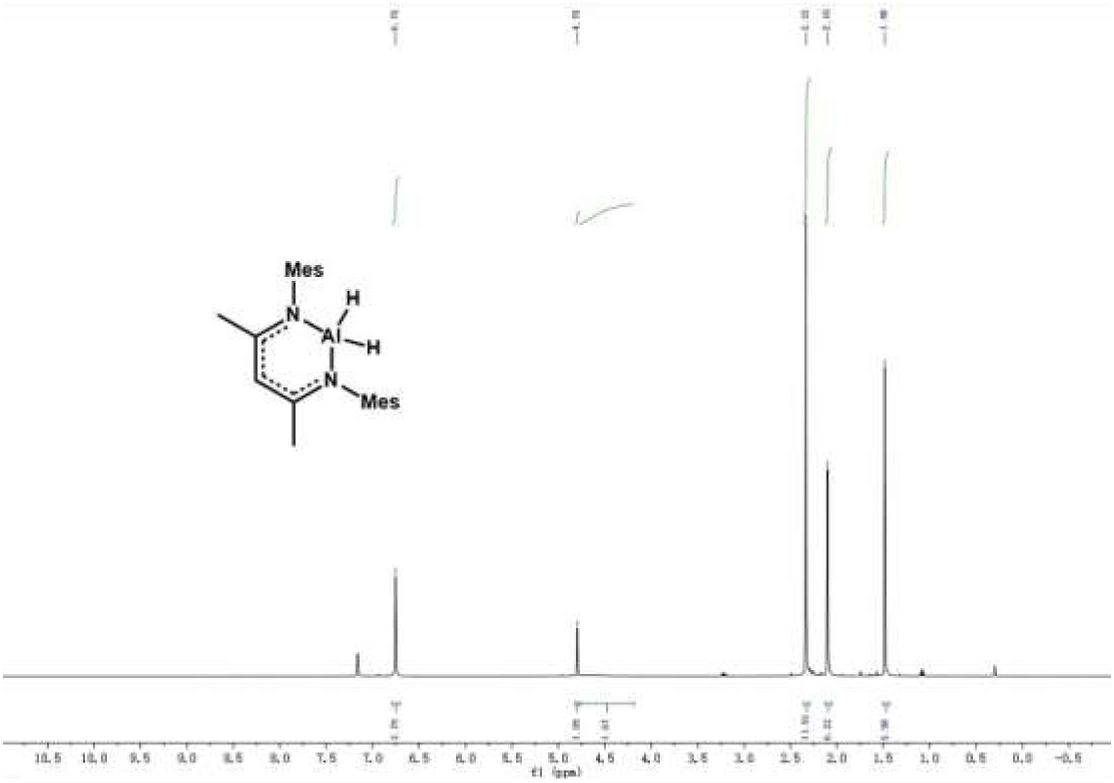

$^{Mes}BDIAlF_2$:

^{1}H NMR (C_6D_6, 400 MHz)

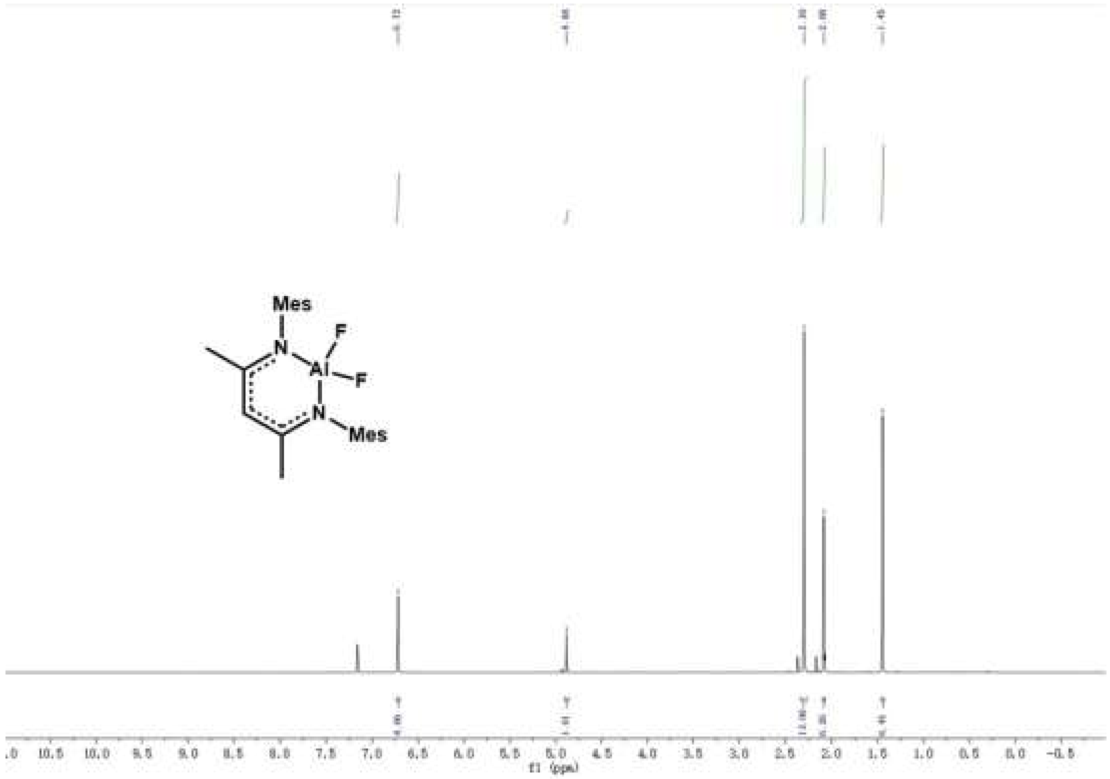

$^{Mes}BDIAlF_2$:

^{19}F NMR (C_6D_6, 376.5 MHz)

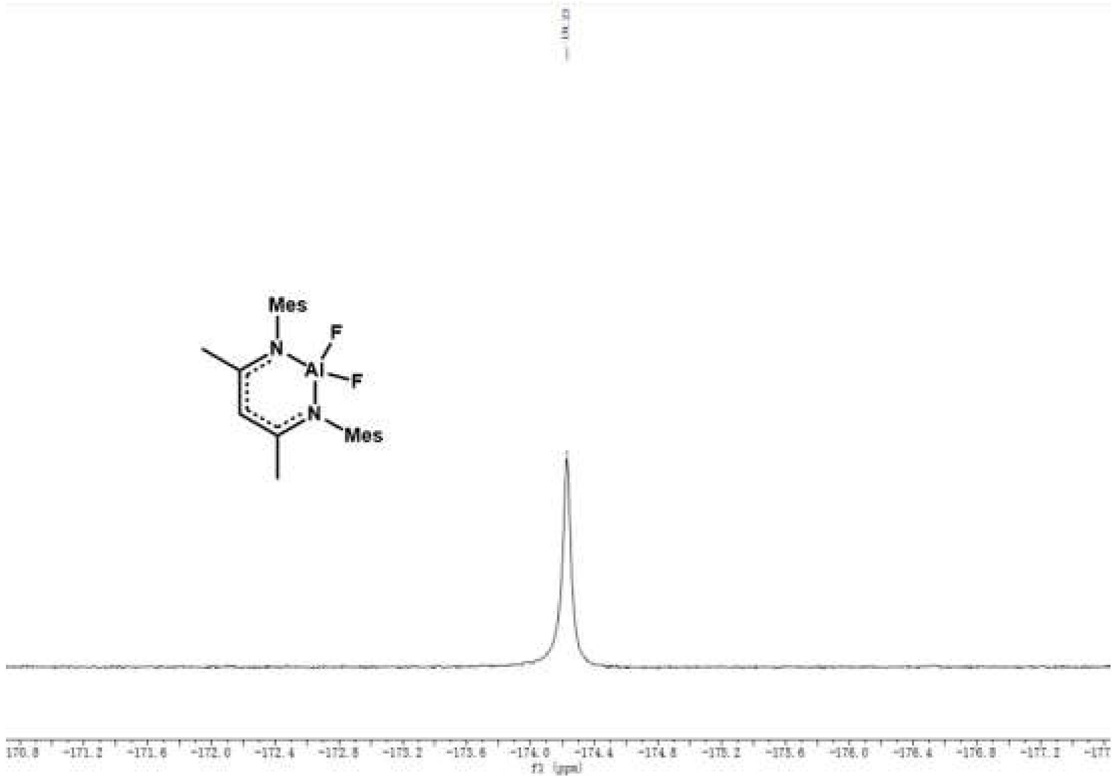

$^{Mes}BDIGaH_2$:

^1H NMR (C_6D_6, 400 MHz)

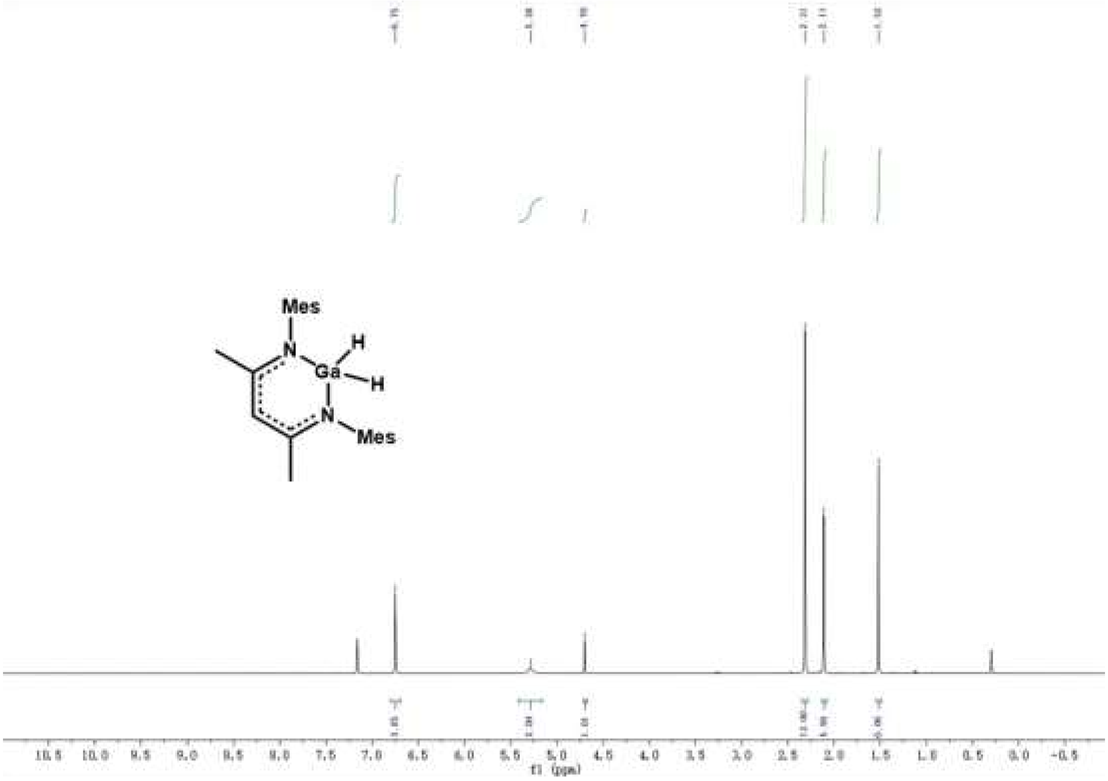

$^{Mes}BDIGaI_2$:

^1H NMR (C_6D_6, 400 MHz)

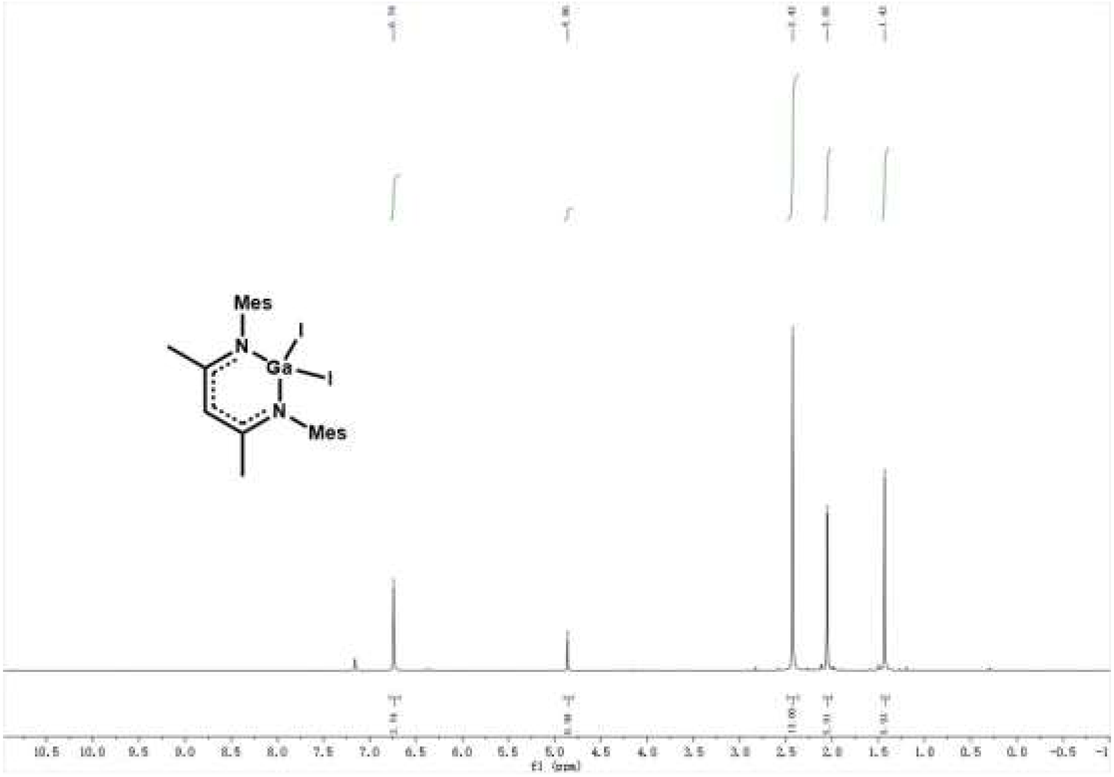

^{13}C NMR (C_6D_6, 101 MHz)

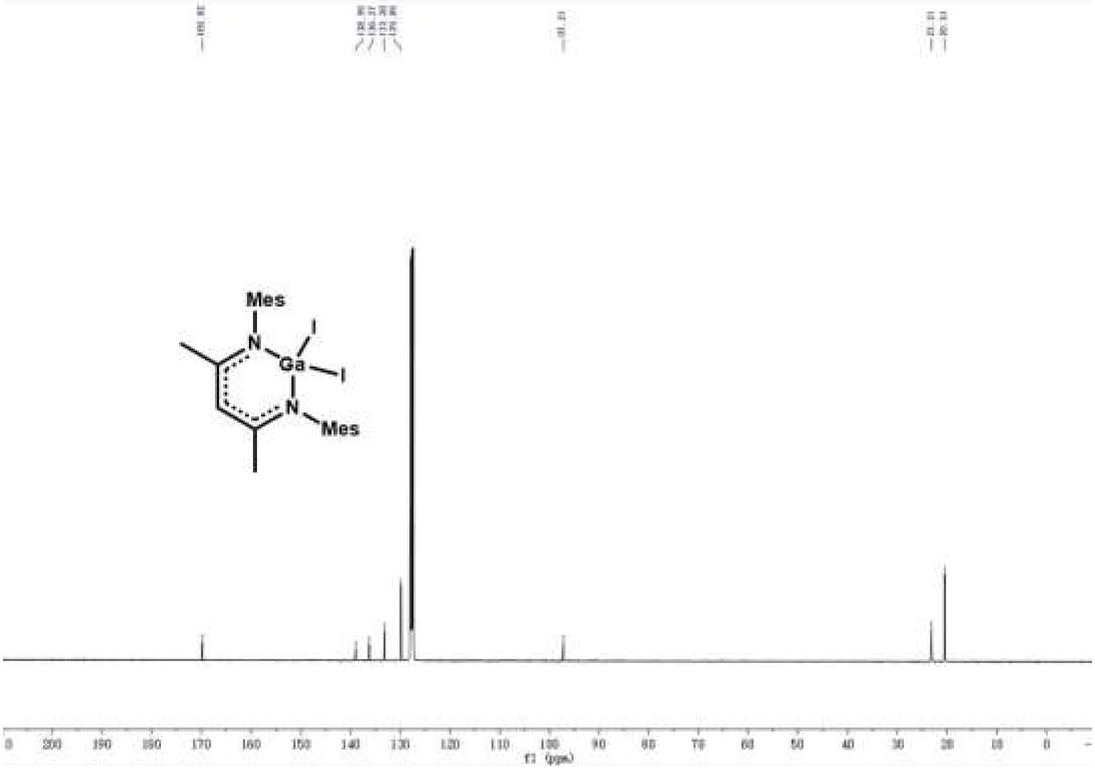

$^{Mes}BDIGaF_2$:

^{1}H NMR (C_6D_6, 400 MHz)

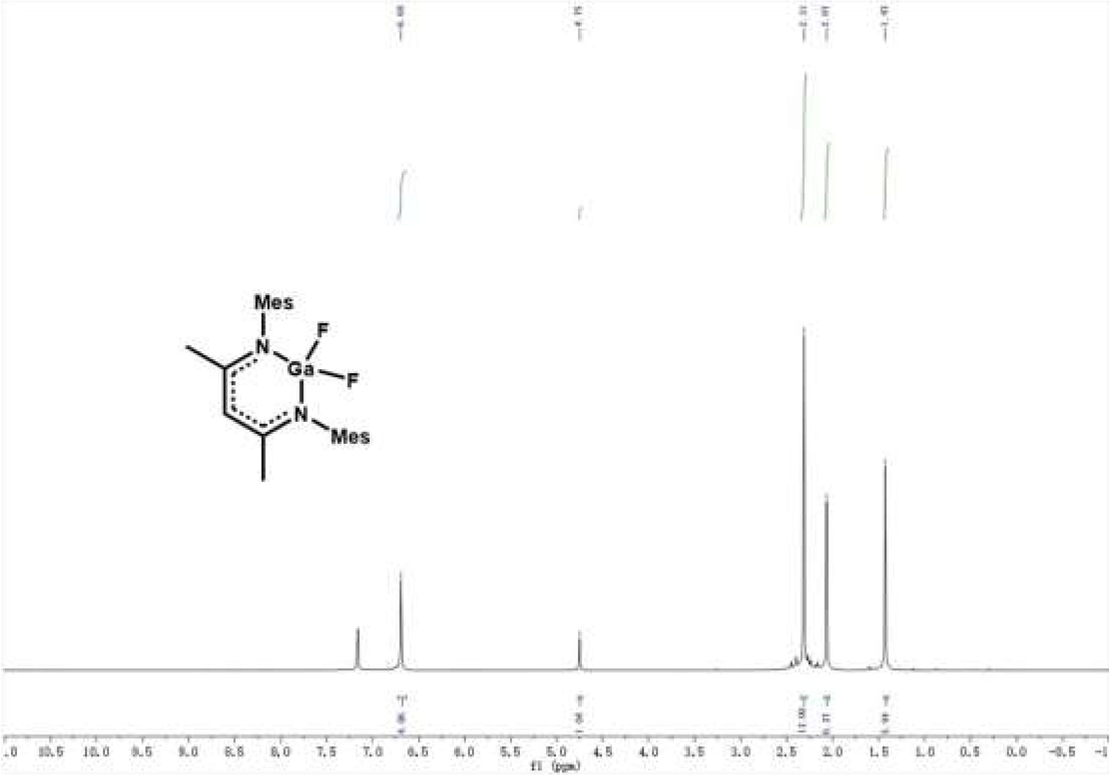

^{19}F NMR (C_6D_6, 376.5 MHz)

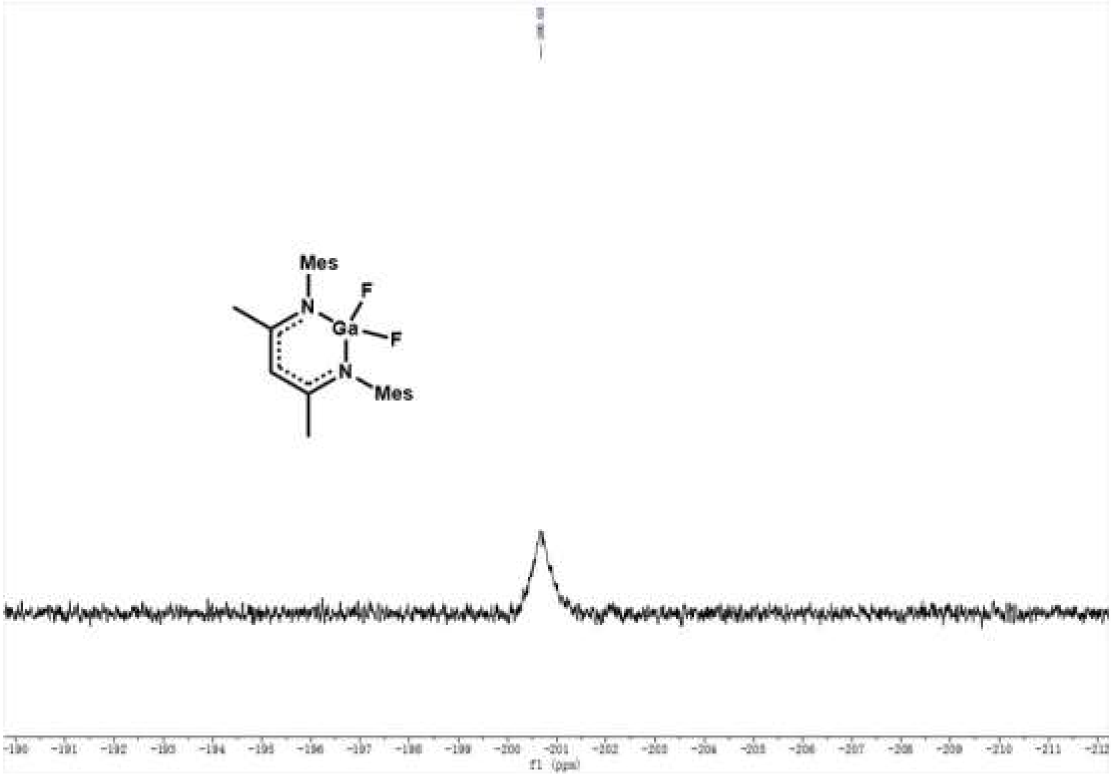

^{13}C NMR (C_6D_6, 101 MHz)

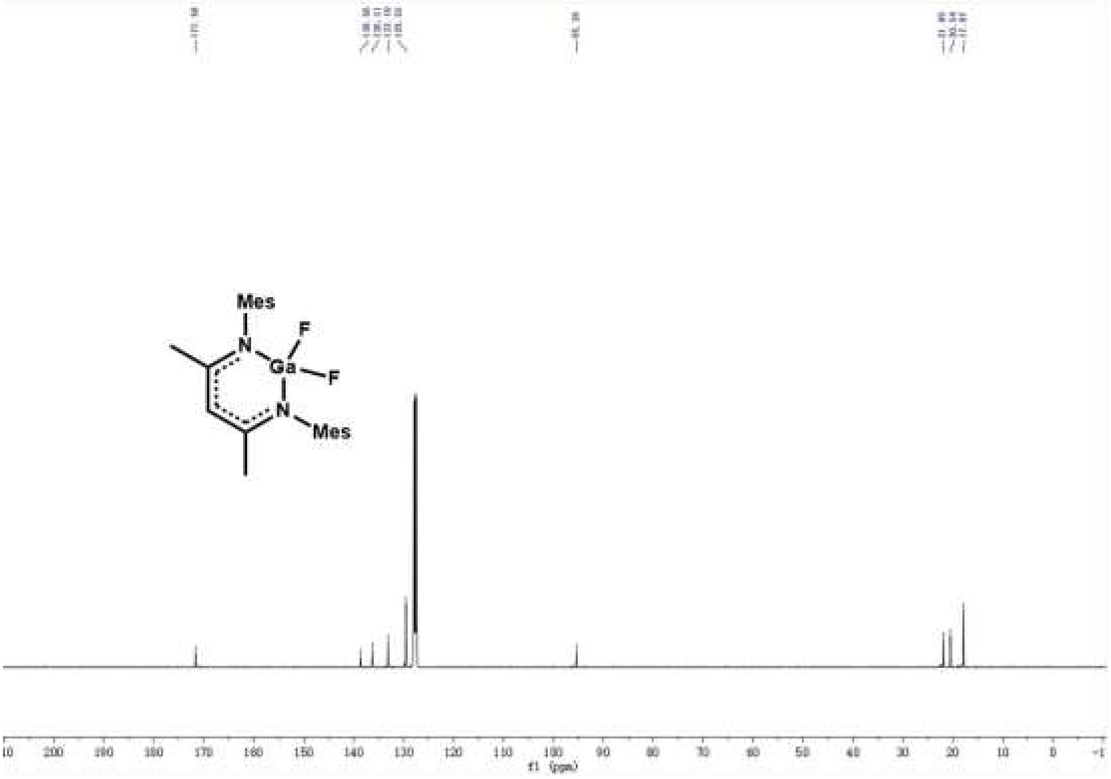

^{Mes}BDIAl(S-C_6H_4Me)$_2$: ("*"is the impurity peak of ^{Mes}BDIAl(S-C_6H_4Me)F, about 6%)

^{1}H NMR (C_6D_6, 400 MHz)

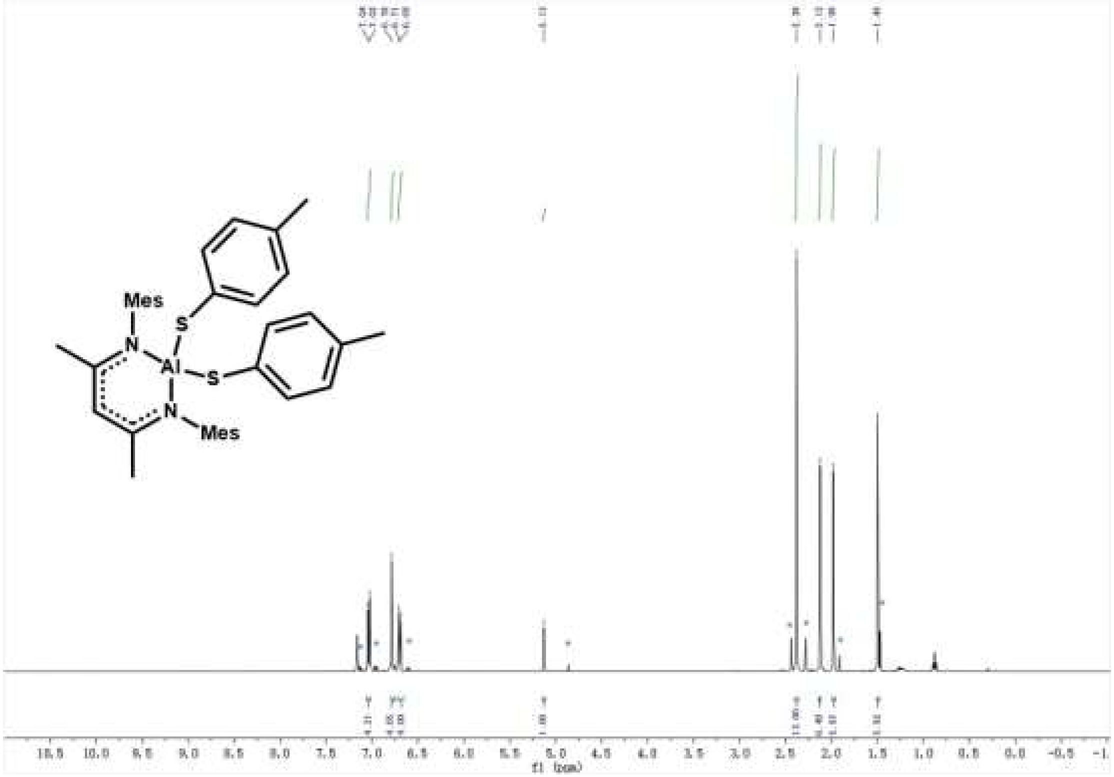

^{13}C NMR (C_6D_6, 101 MHz)

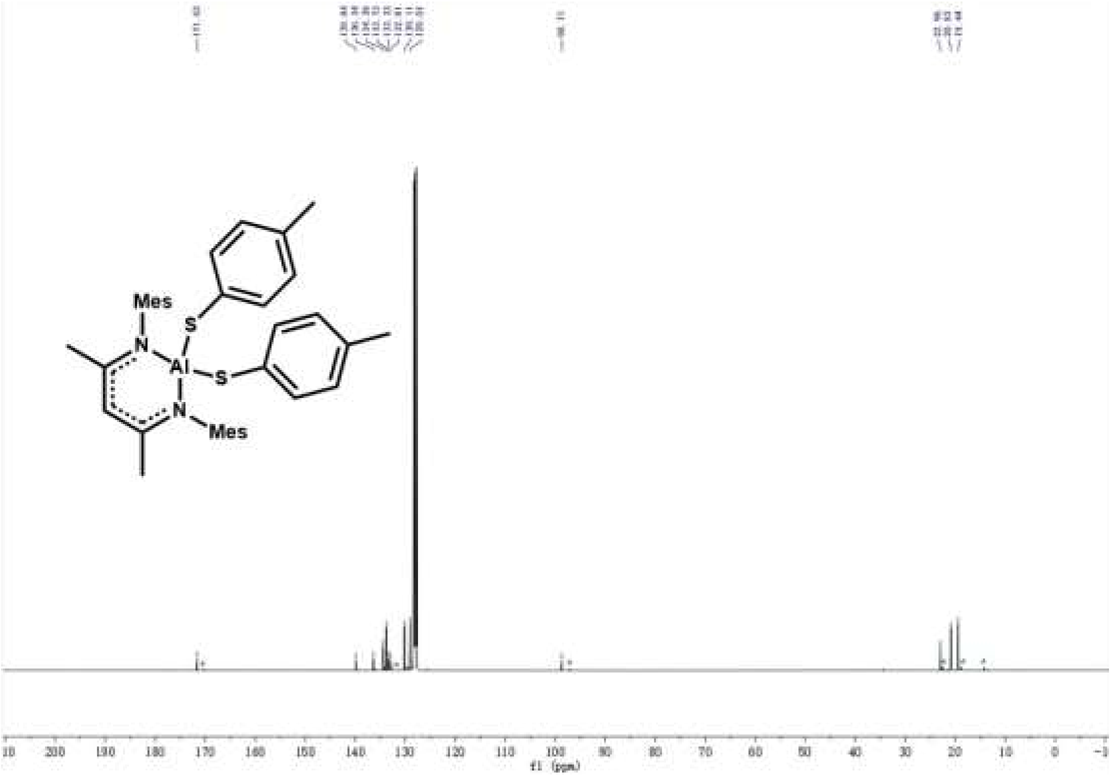

7.2. NMR Spectra of the Isolated Products

2,3,5,6-Tetrafluoropyridine (**2a**)

^{1}H NMR ($CDCl_3$, 400 MHz)

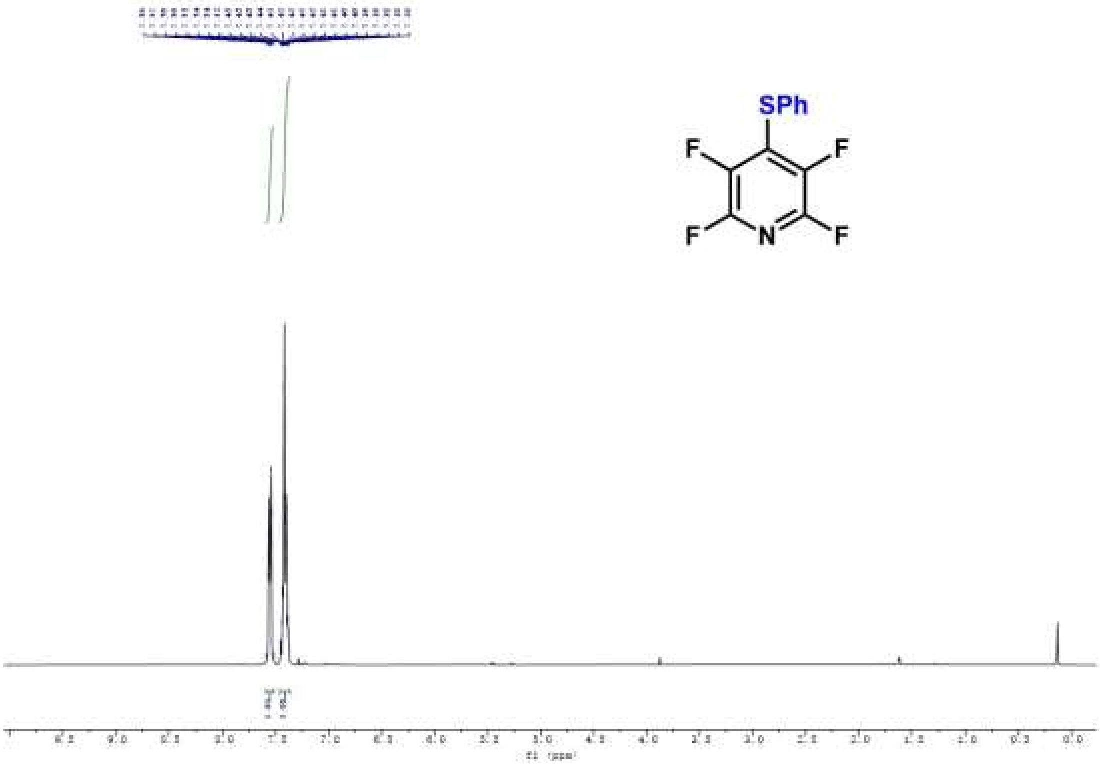

^{19}F NMR ($CDCl_3$, 376.5 MHz)

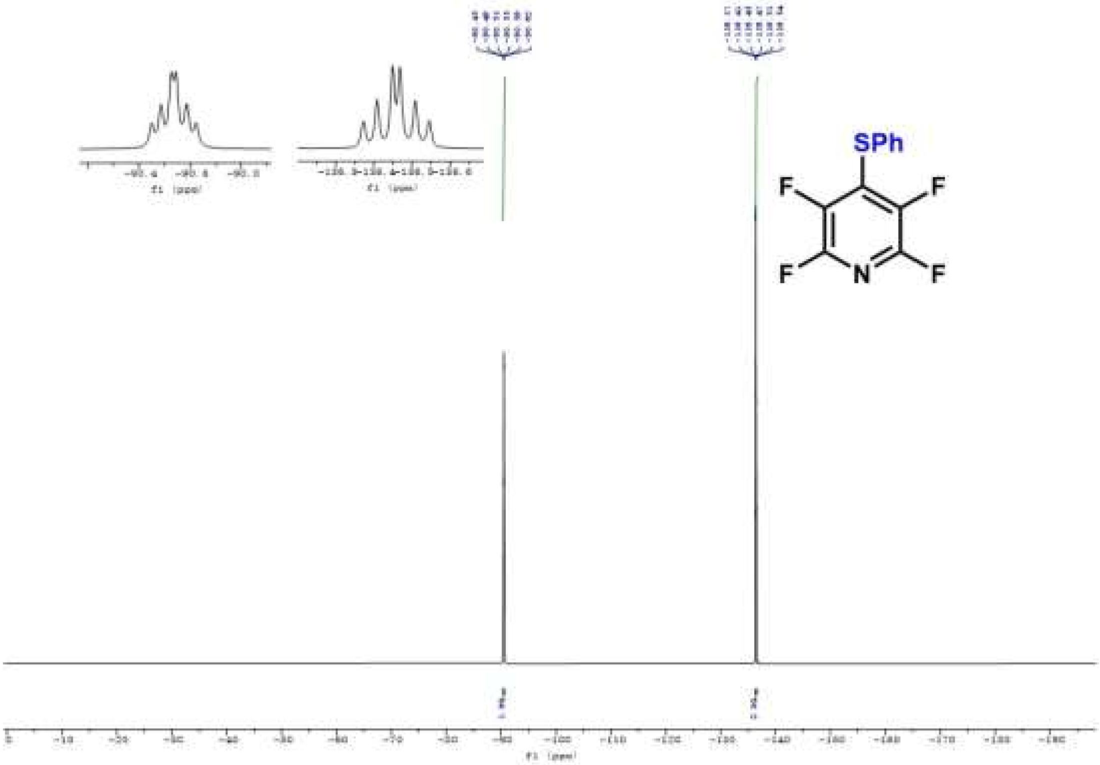

^{13}C NMR ($CDCl_3$, 101 MHz)

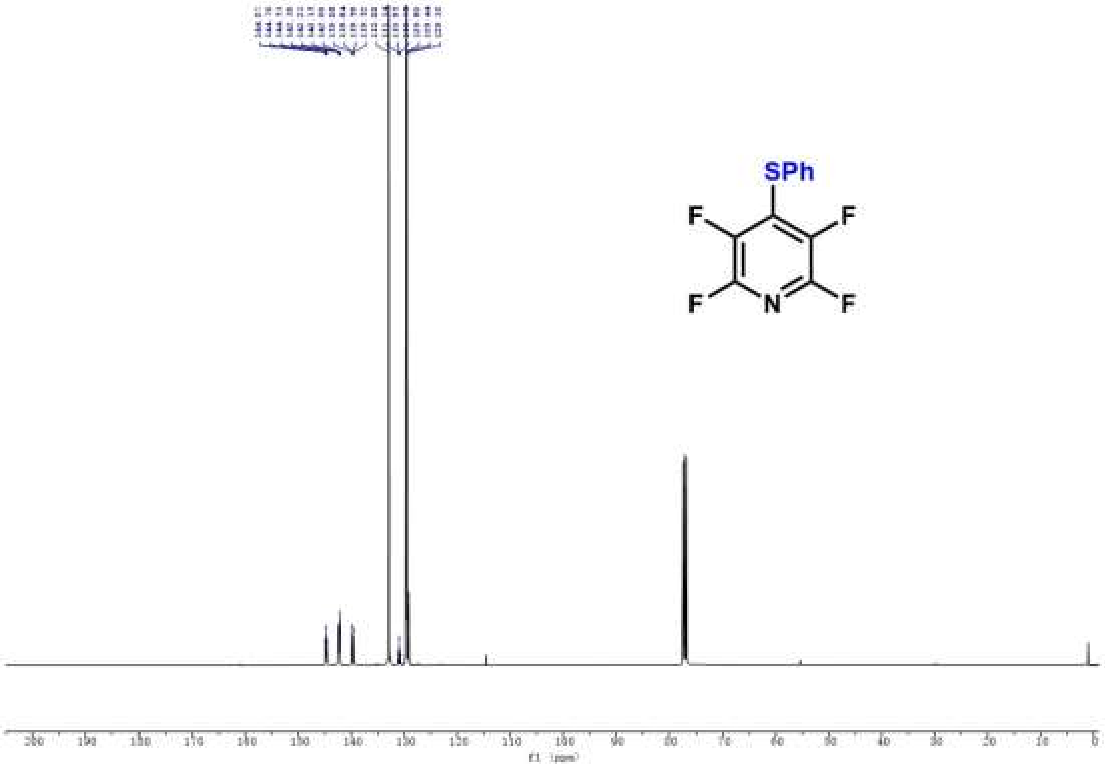

2,5-difluoro-3,4,6-tris(phenylthio)pyridine (**2a’**)

^{1}H NMR (CDCl$_3$, 400 MHz)

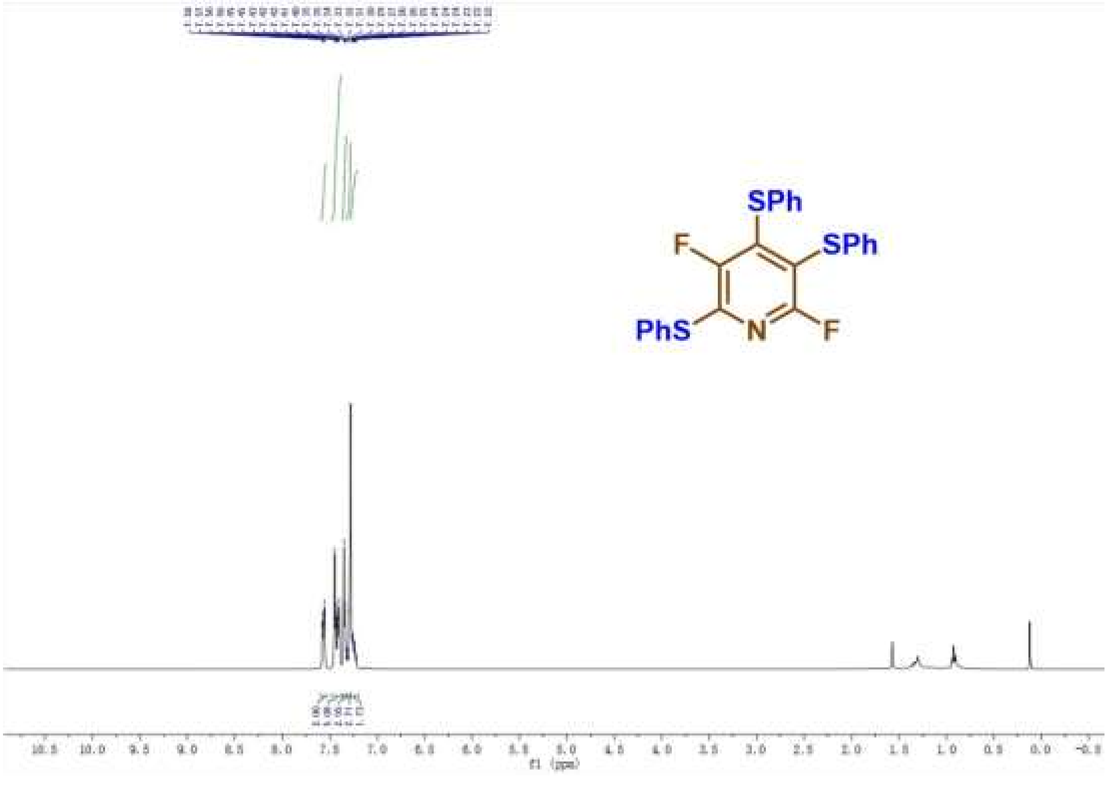

^{19}F NMR ($CDCl_3$, 376.5 MHz)

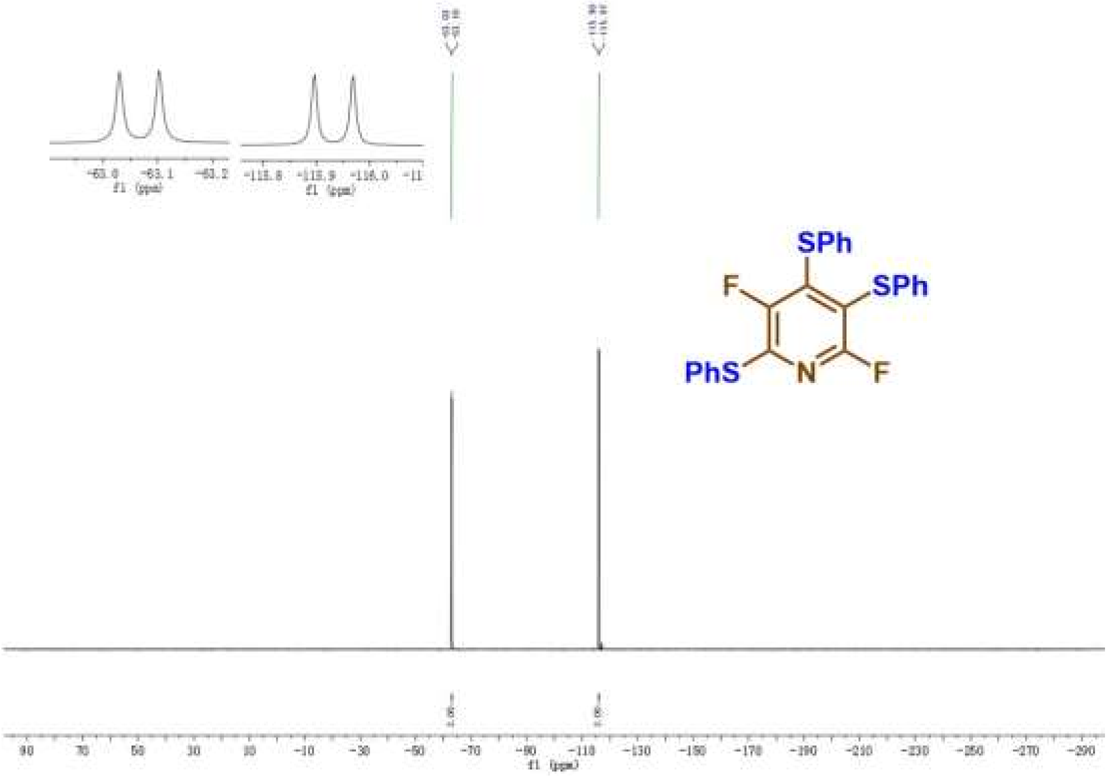

^{13}C NMR ($CDCl_3$, 101 MHz)

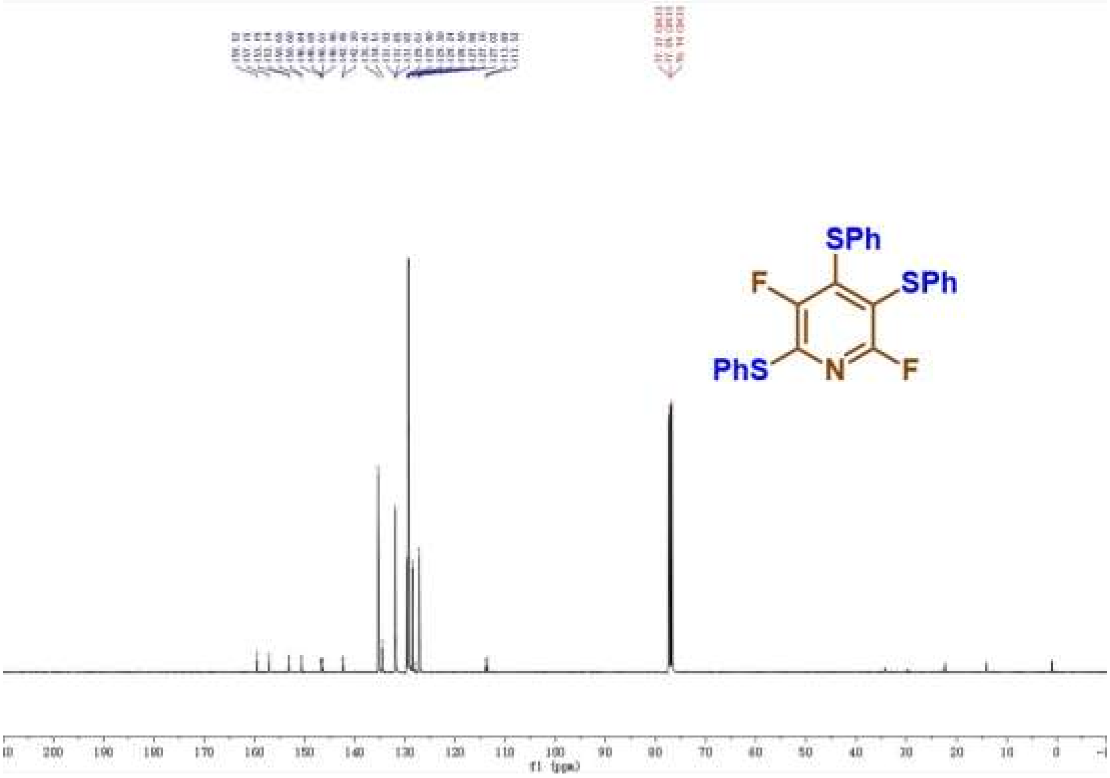

3,5-dichloro-2,6-difluoro-4-(phenylthio)pyridine (**2b**)

^{1}H NMR ($CDCl_3$, 400 MHz)

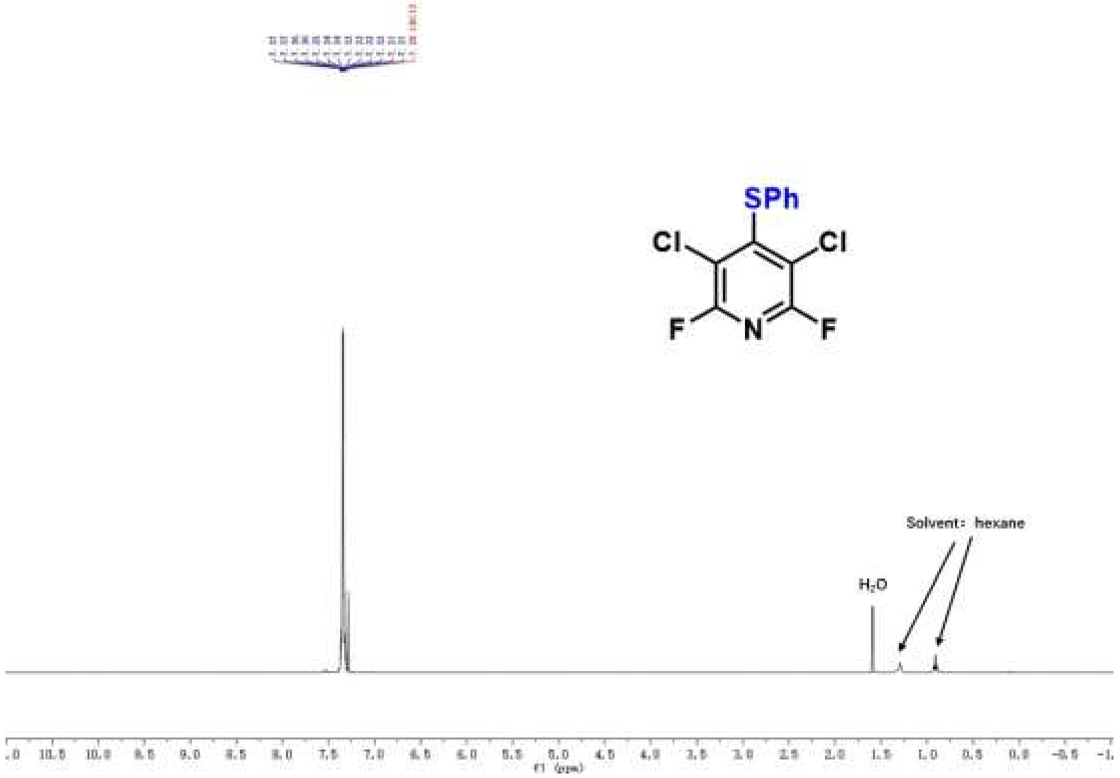

^{19}F NMR ($CDCl_3$, 376.5 MHz)

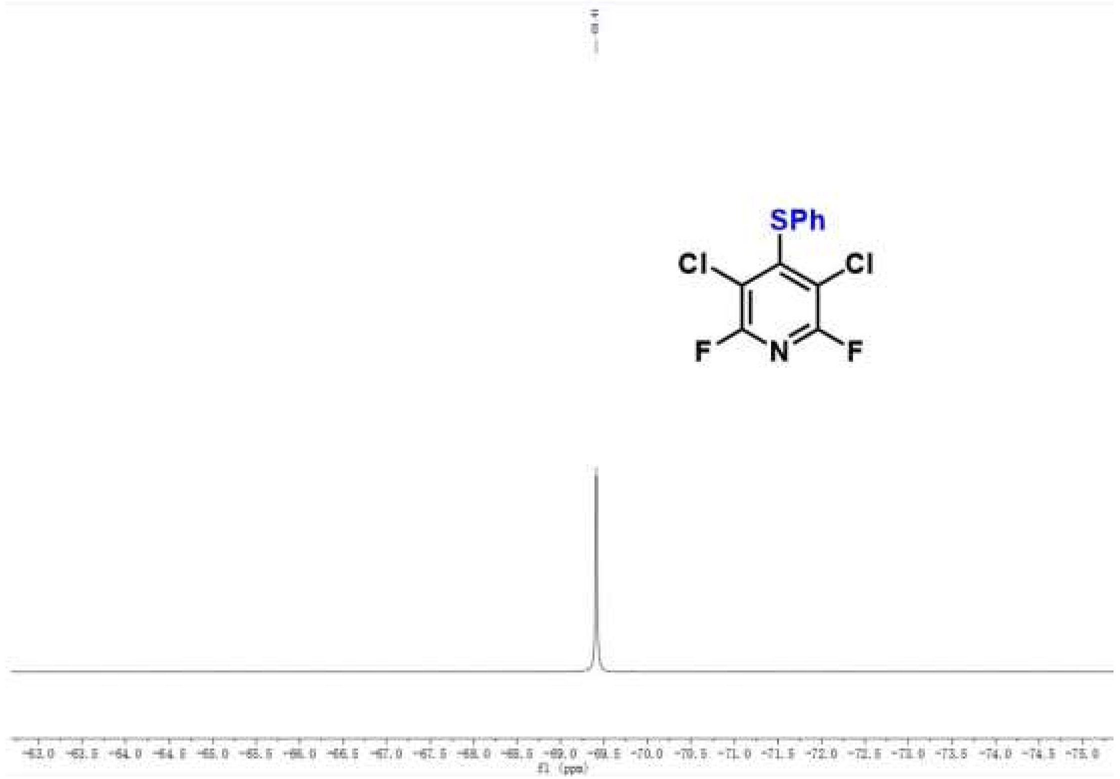

^{13}C NMR ($CDCl_3$, 101 MHz)

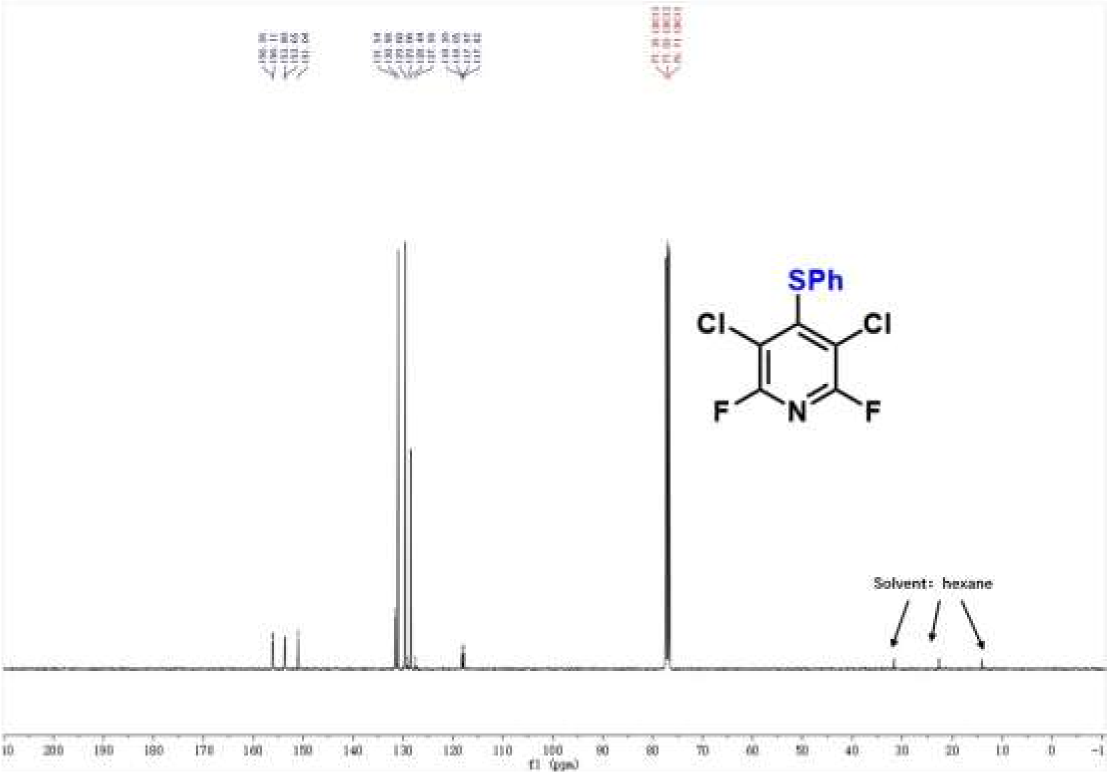

phenyl(2,3,5,6-tetrafluoro-4-(trifluoromethyl)phenyl)sulfane (**2c**)

^{1}H NMR ($CDCl_3$, 400 MHz)

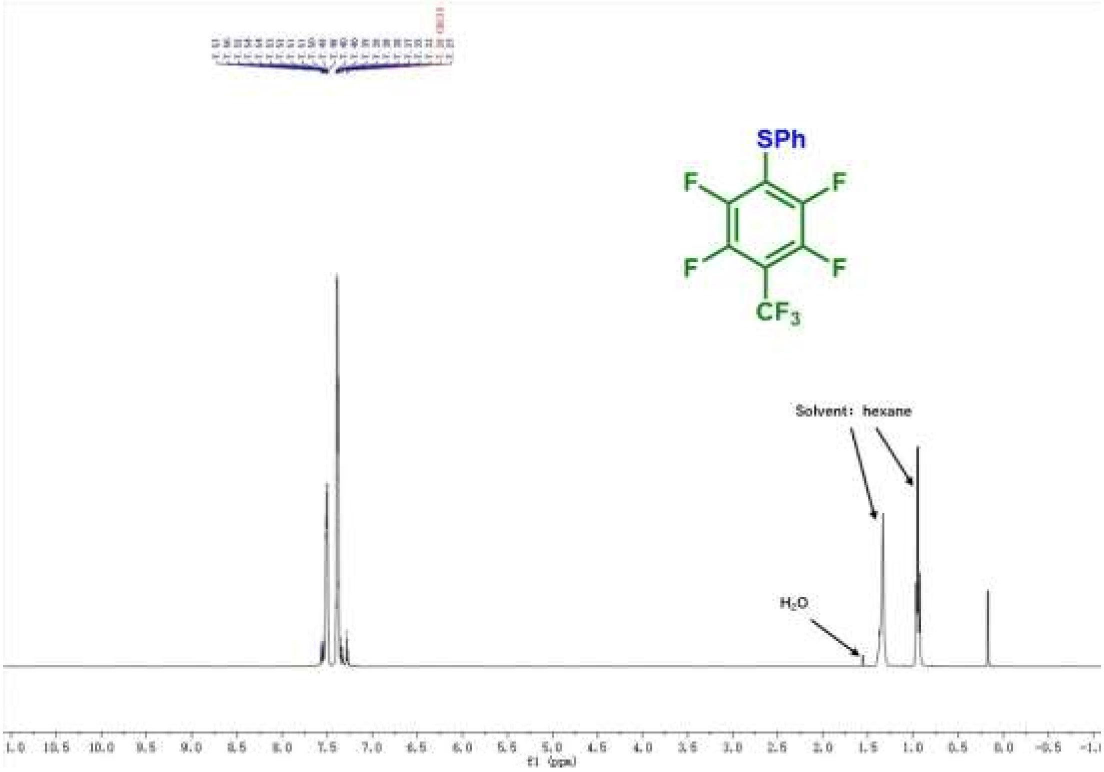

^{19}F NMR (CDCl$_3$, 376.5 MHz)

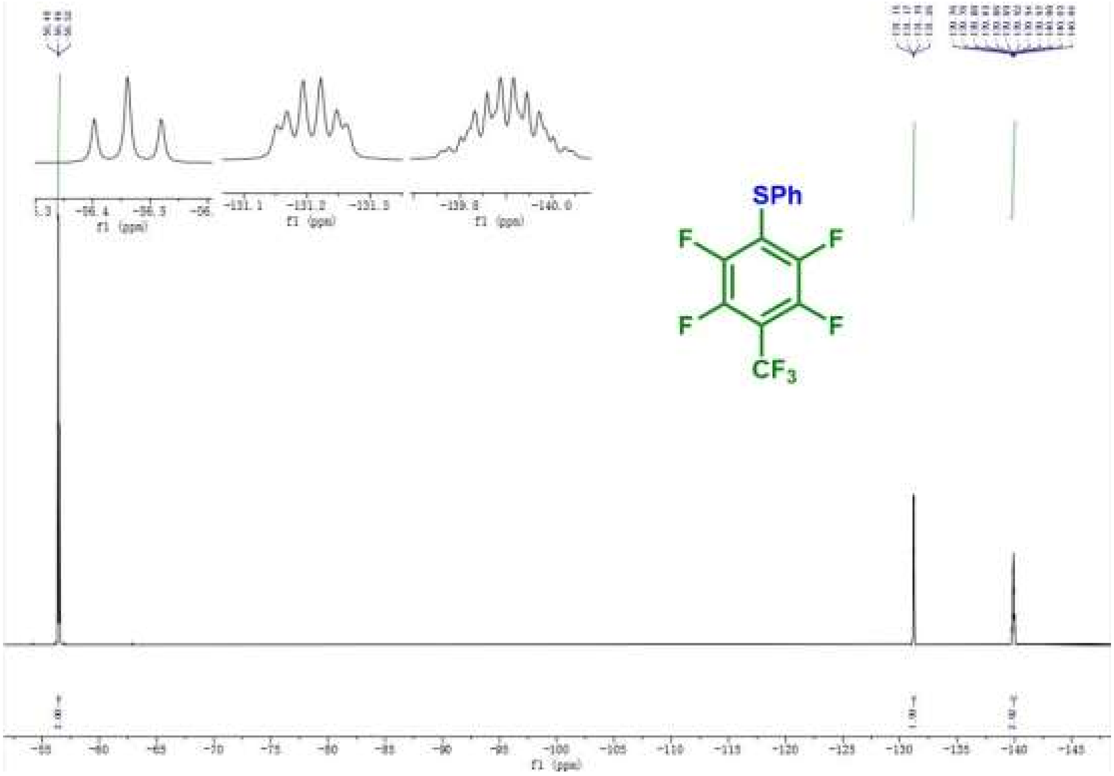

^{13}C NMR (CDCl$_3$, 101 MHz)

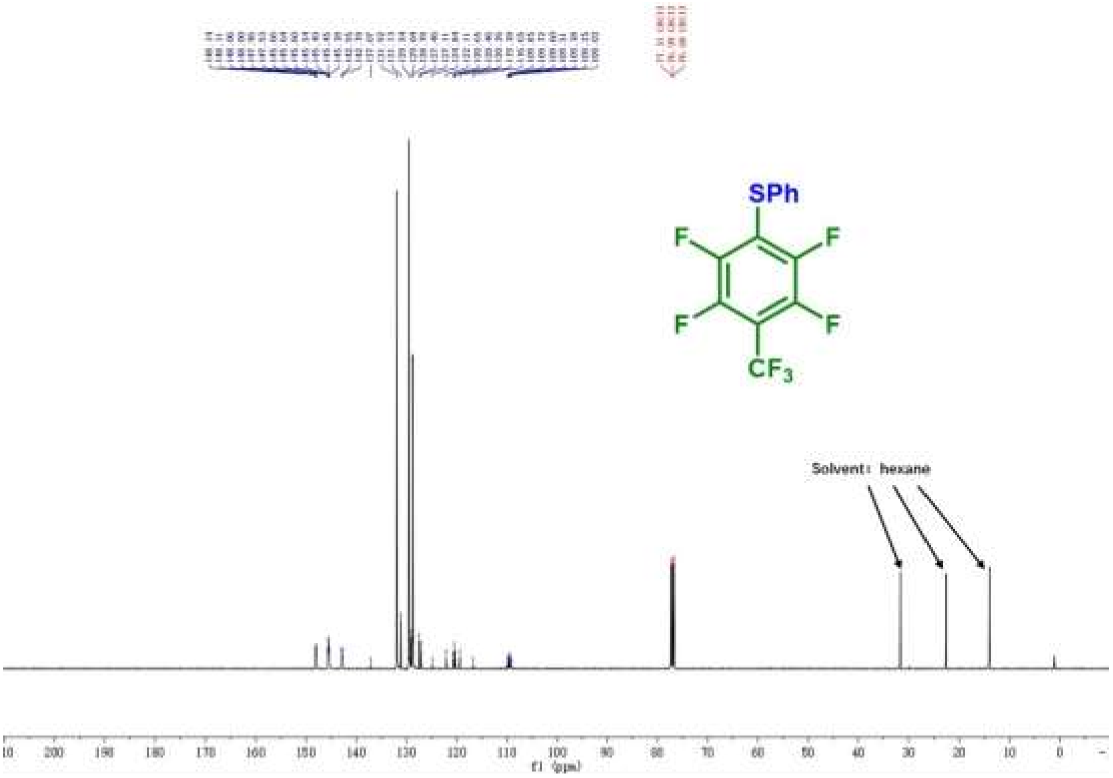

(3,6-difluoro-5-(trifluoromethyl)benzene-1,2,4-triyl)tris(phenylsulfane) (**2c'**)

^{1}H NMR ($CDCl_3$, 400 MHz)

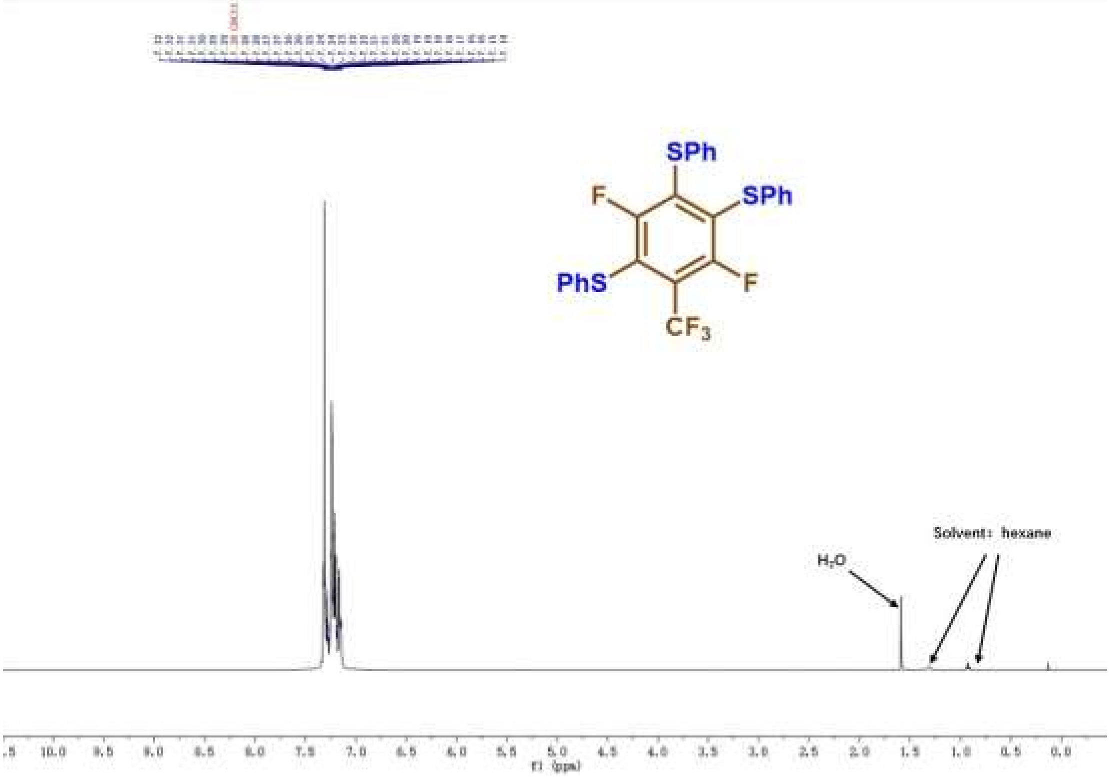

^{19}F NMR ($CDCl_3$, 376.5 MHz)

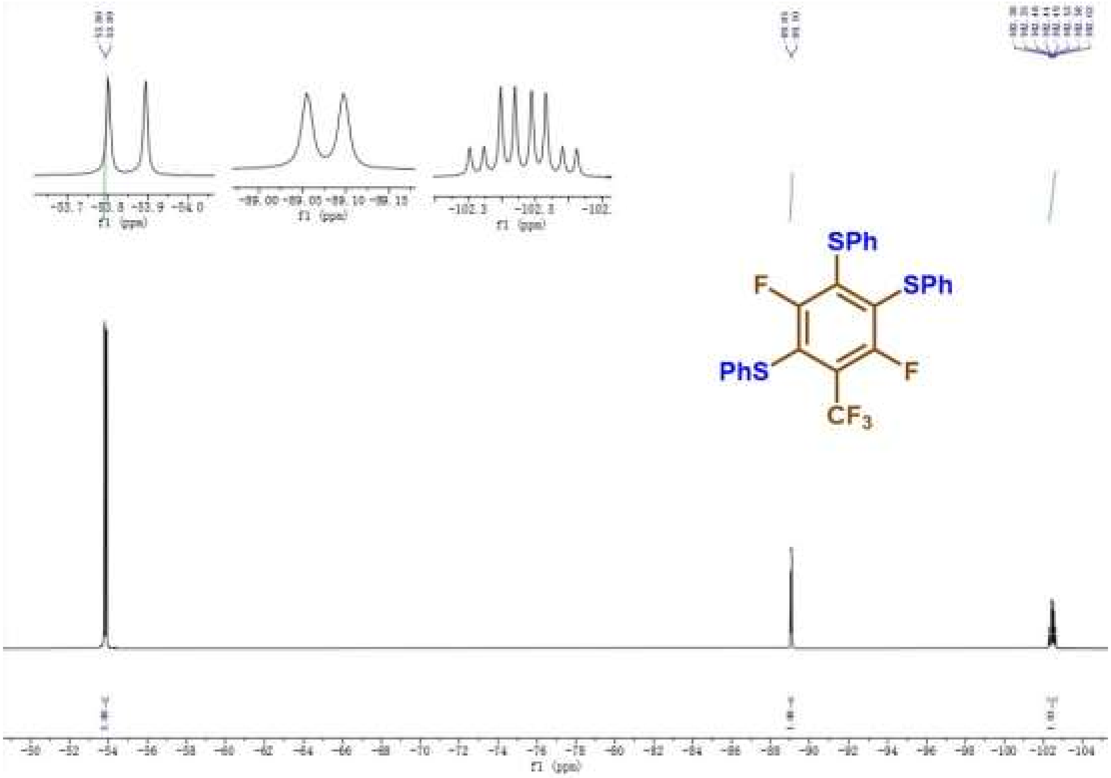

^{13}C NMR ($CDCl_3$, 101 MHz)

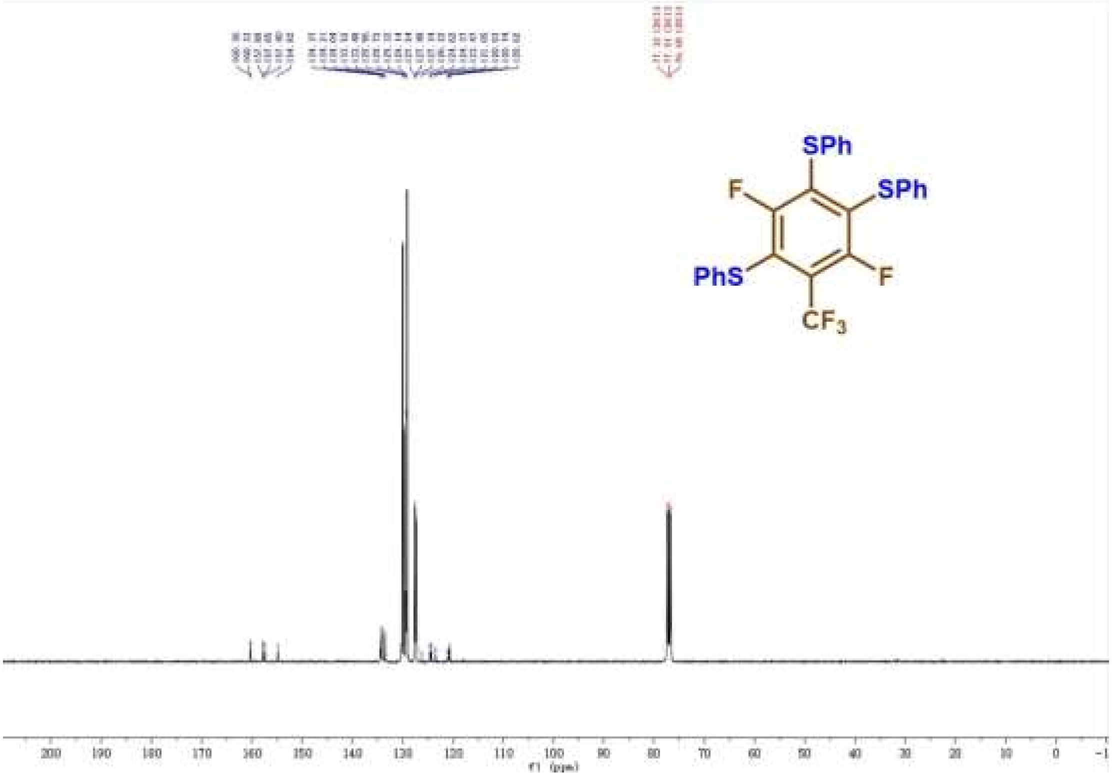

diphenyl(2,3,5,6-tetrafluoro-4-(phenylthio)phenyl)phosphane (**2d**)

^{1}H NMR ($CDCl_3$, 400 MHz)

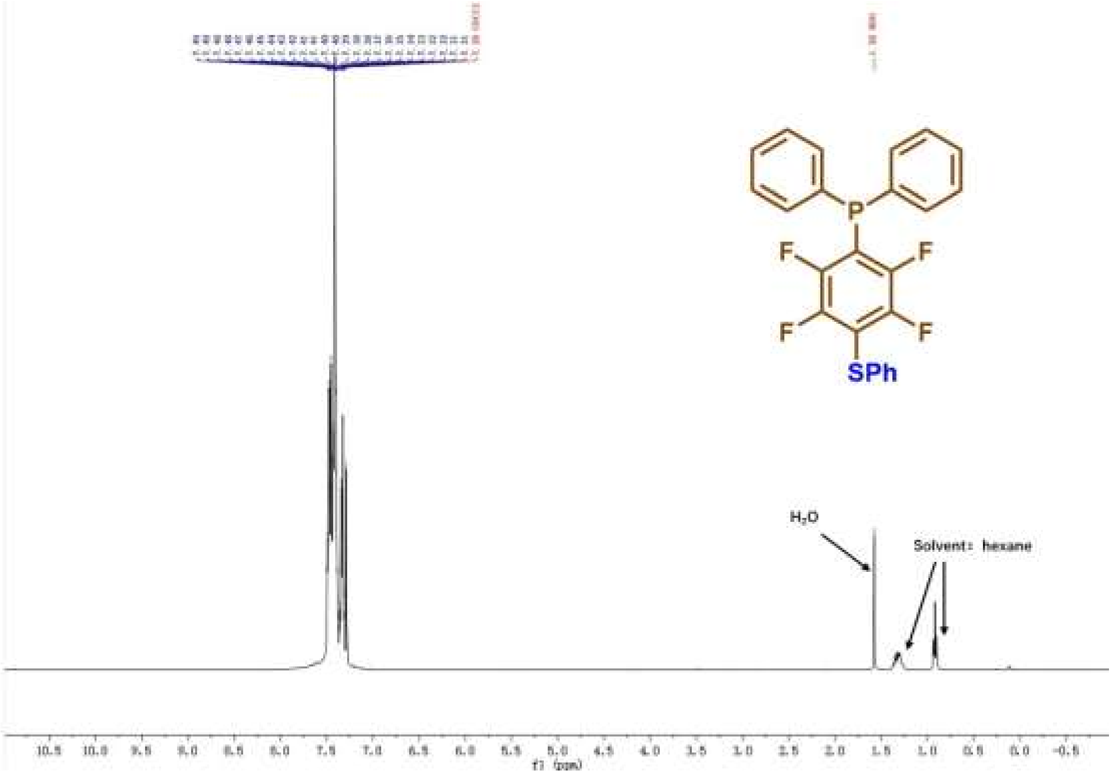

^{19}F NMR (CDCl$_3$, 376.5 MHz)

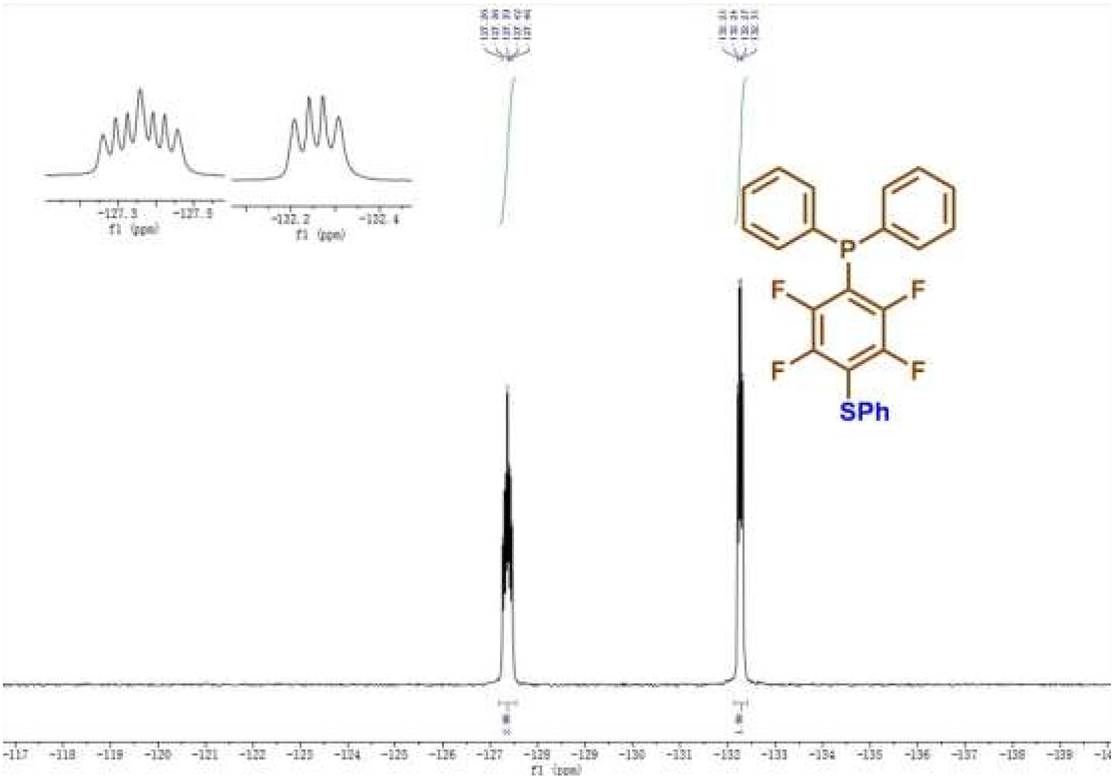

^{31}P NMR (162 MHz, CDCl$_3$)

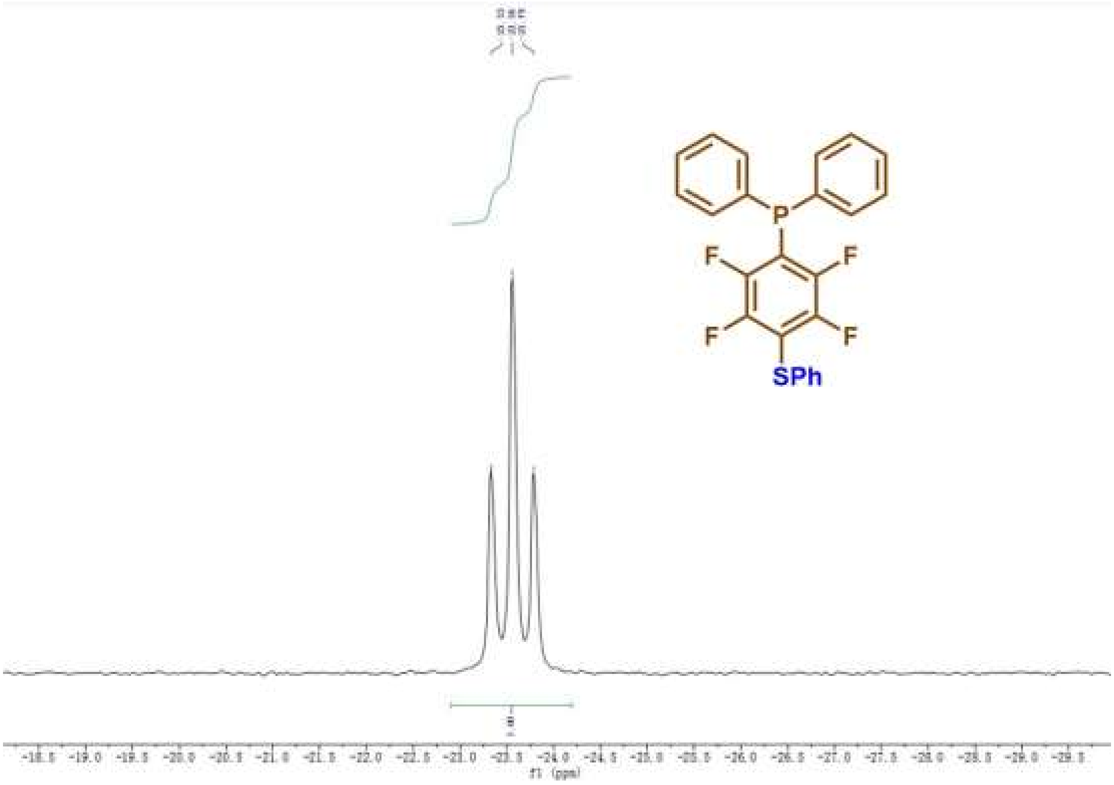

^{13}C NMR ($CDCl_3$, 101 MHz)

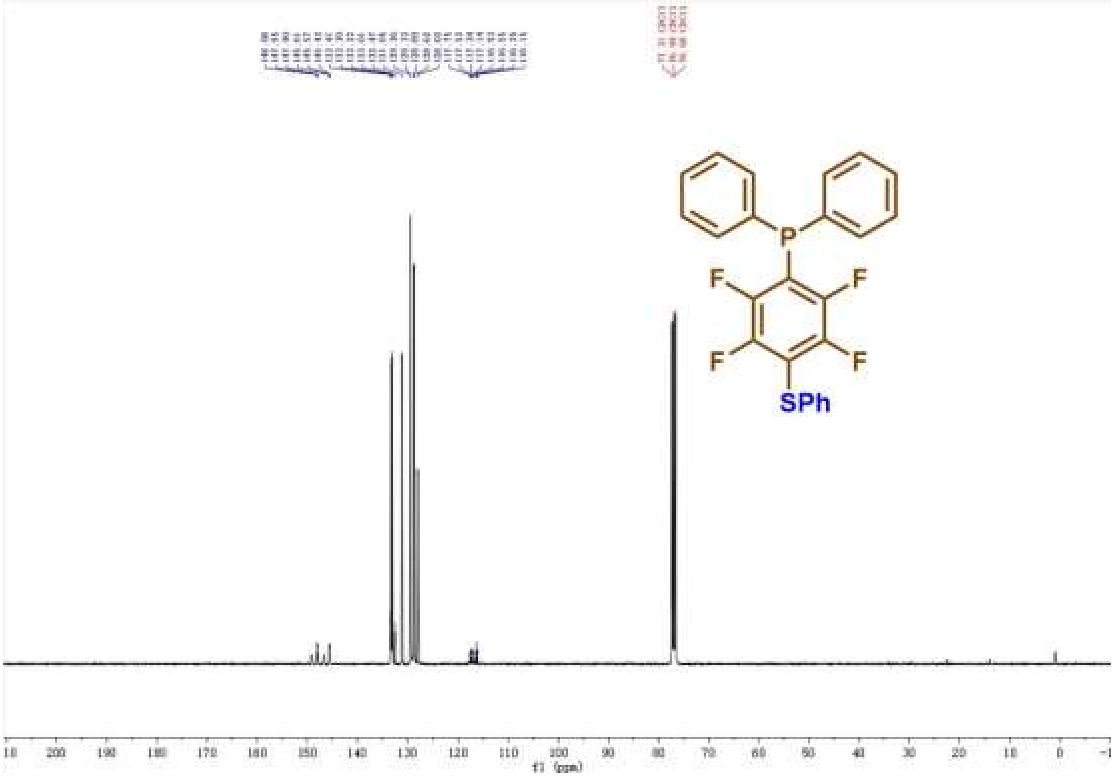

phenyl(2,3,5,6-tetrafluoro-4-(1,2,2-triphenylvinyl)phenyl)sulfane (**2e**)
^{1}H NMR ($CDCl_3$, 400 MHz)

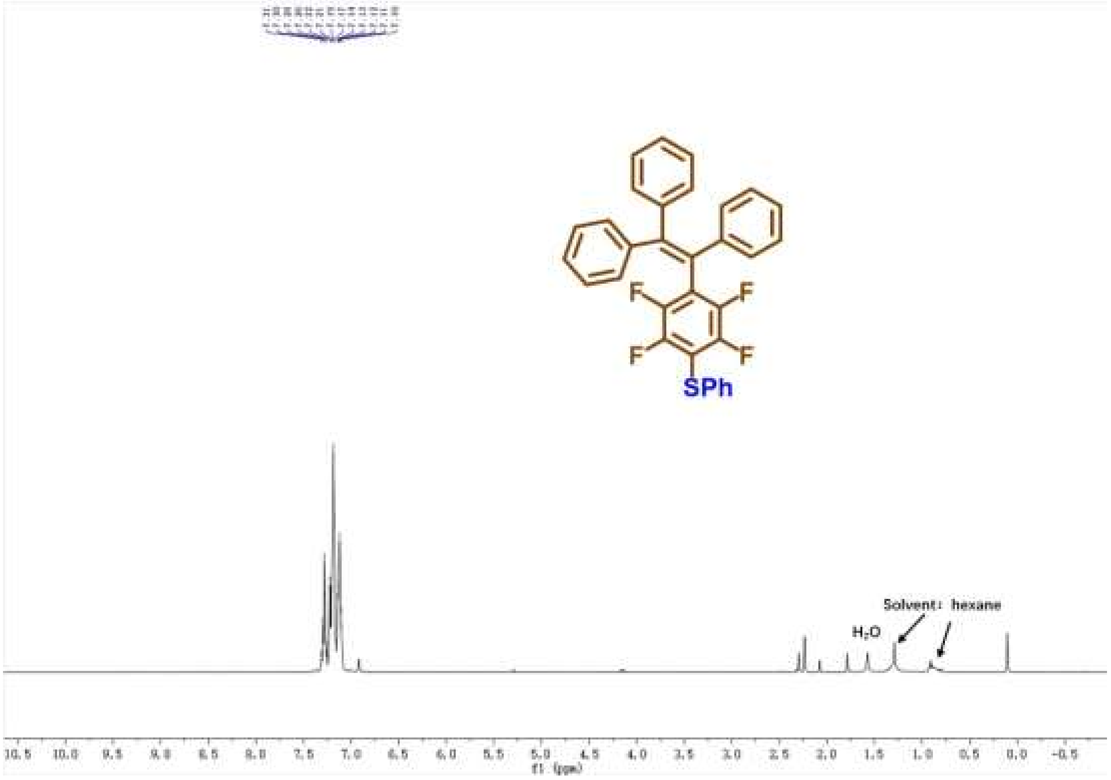

^{19}F NMR ($CDCl_3$, 376.5 MHz)

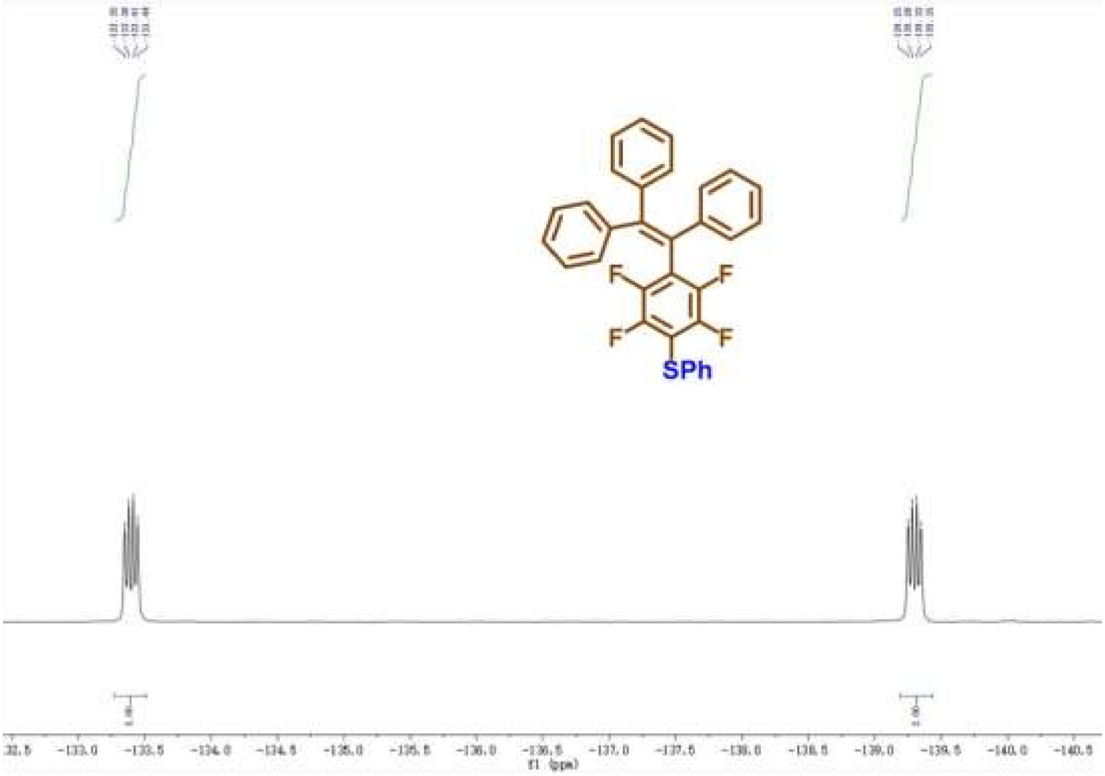

^{13}C NMR ($CDCl_3$, 101 MHz)

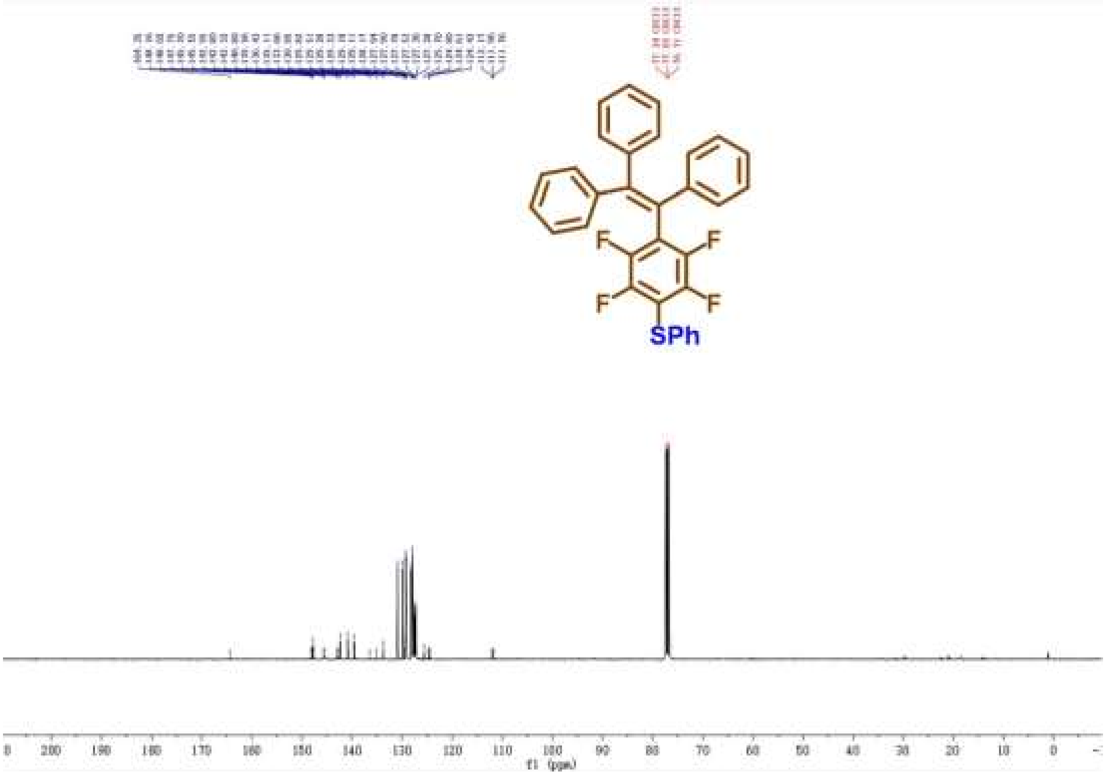

phenyl(2,3,5,6-tetrafluoro-4-(naphthalen-2-yl)phenyl)sulfane (**2f**)

^{1}H NMR ($CDCl_3$, 400 MHz)

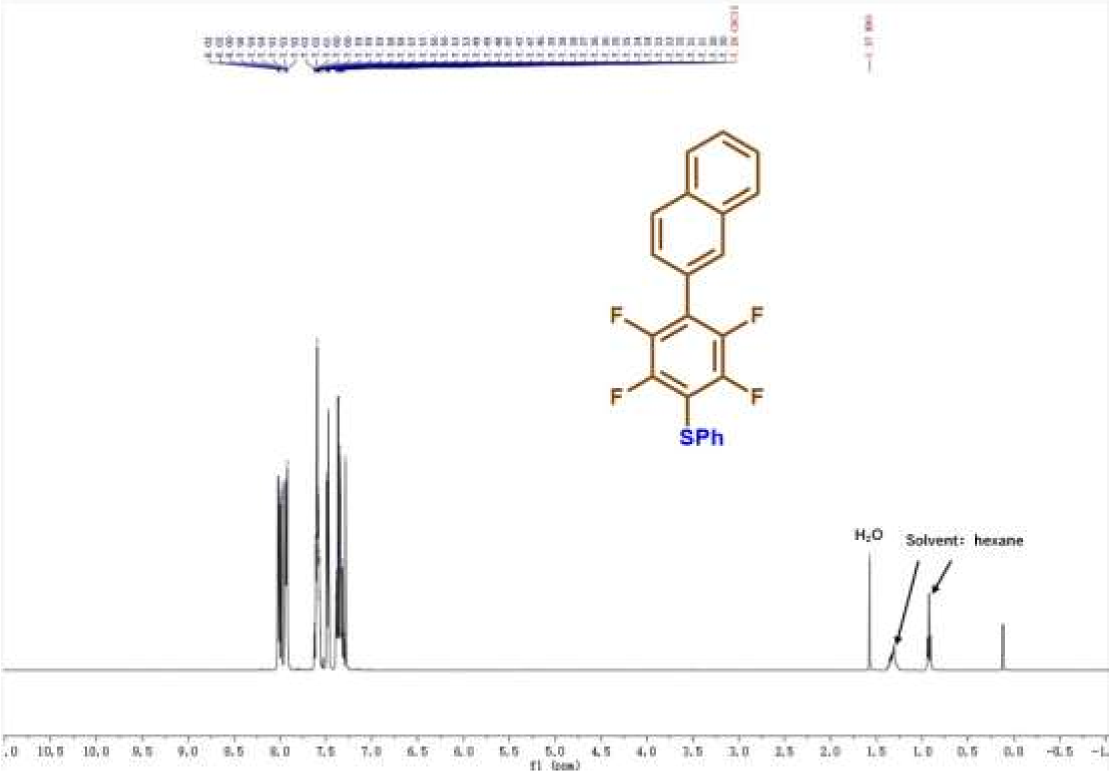

^{19}F NMR ($CDCl_3$, 376.5 MHz)

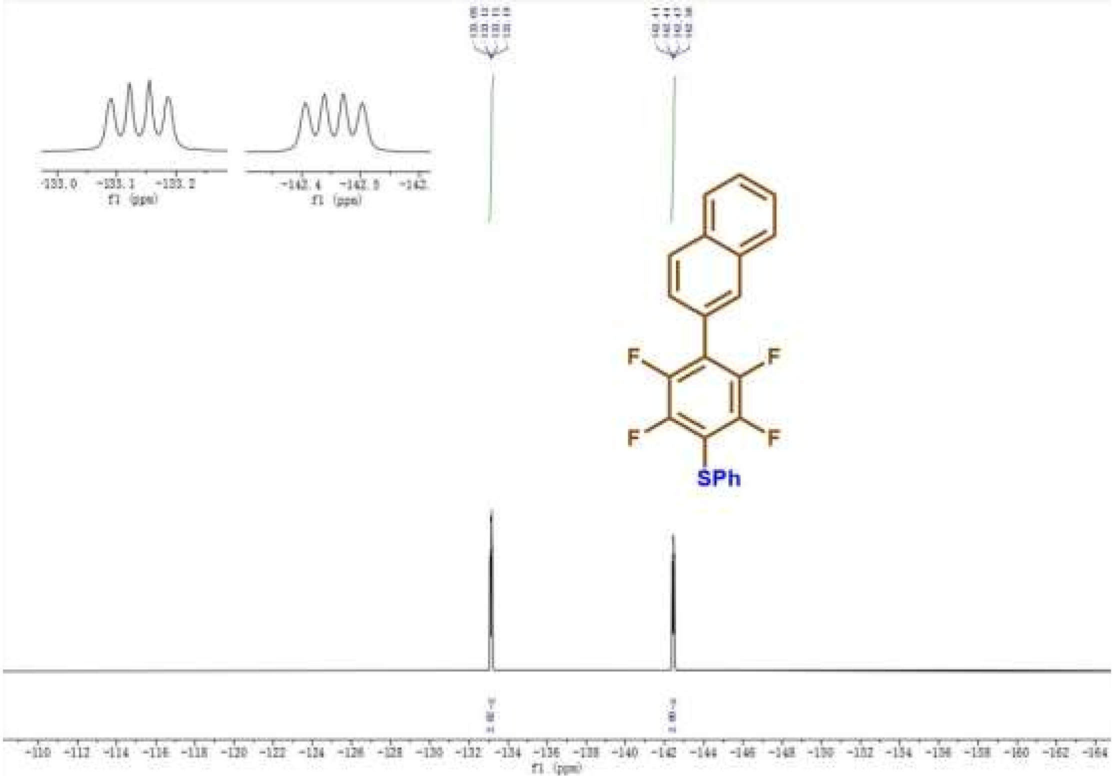

^{13}C NMR ($CDCl_3$, 101 MHz)

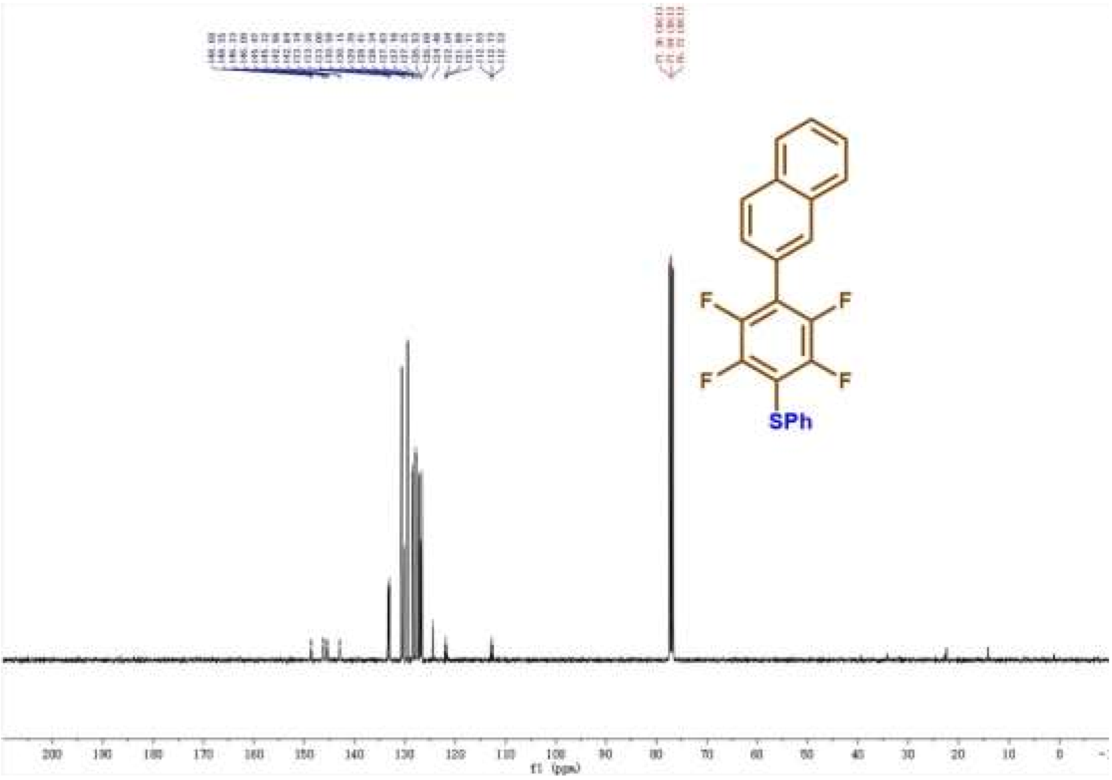

Phenyl(2,3,5,6-tetrafluoro-4'-(trifluoromethyl)-[1,1'-biphenyl]-4-yl) sulfane (**2g**)

^1H NMR ($CDCl_3$, 400 MHz, "*"is the peak of (**2g**), "*"is the peak of trimethyl(phenylthio)silane, about 5%)

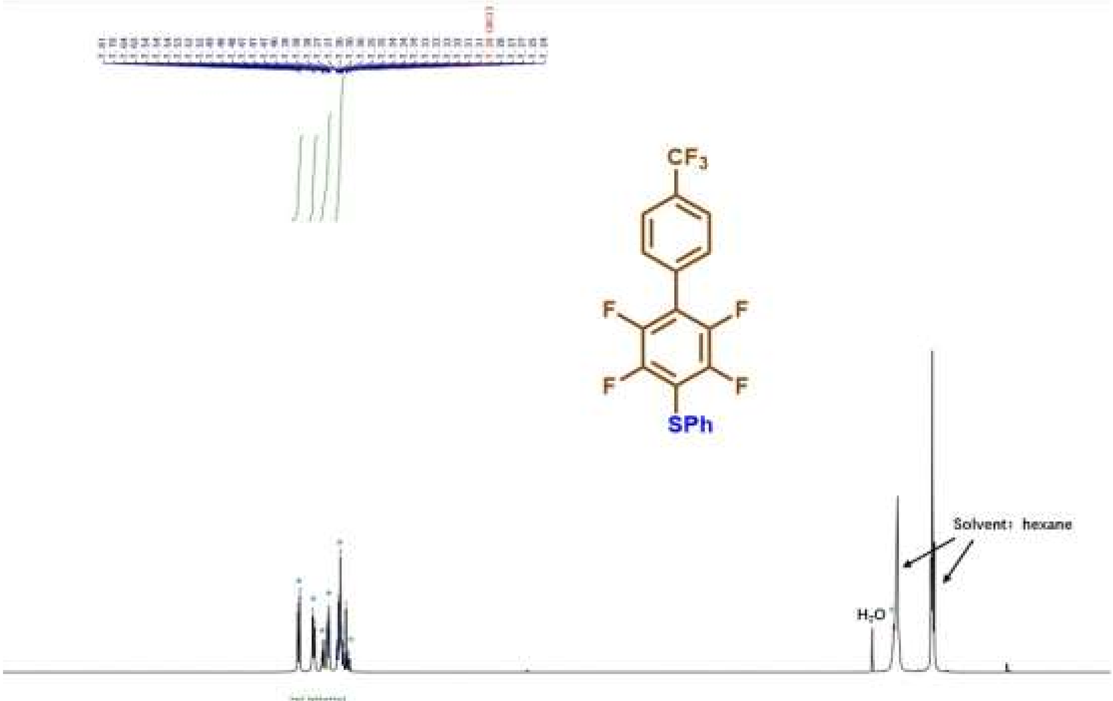

^{19}F NMR ($CDCl_3$, 376.5 MHz)

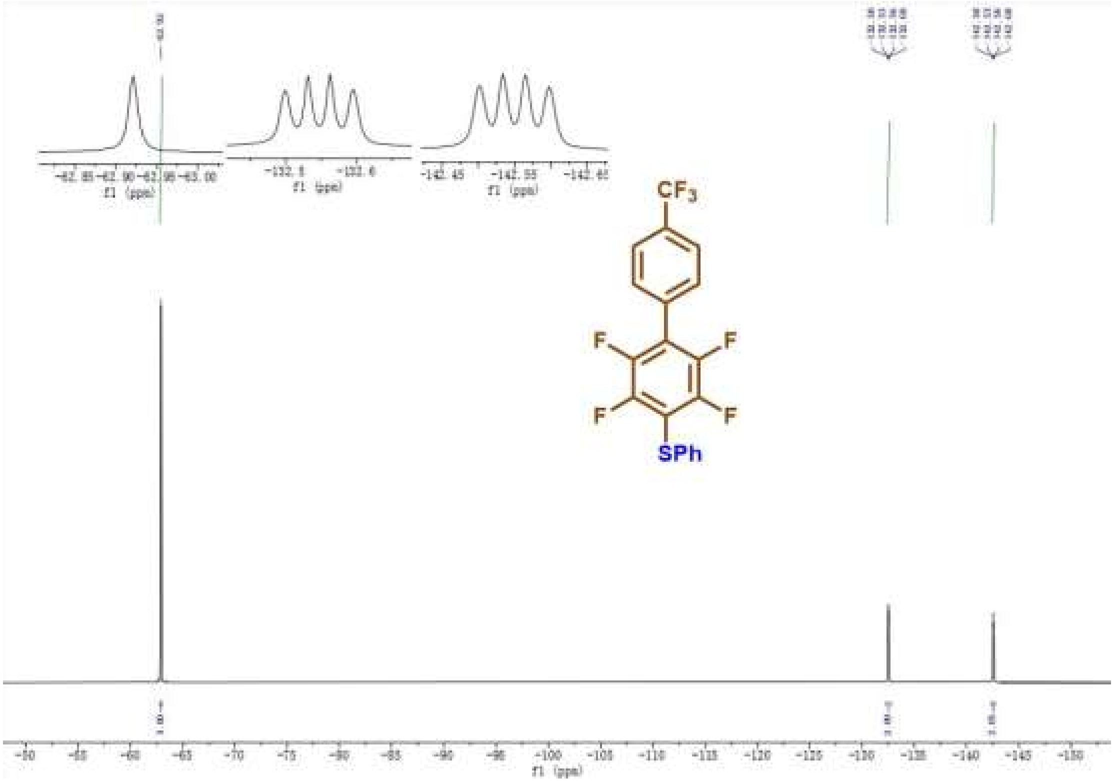

^{13}C NMR ($CDCl_3$, 101 MHz)

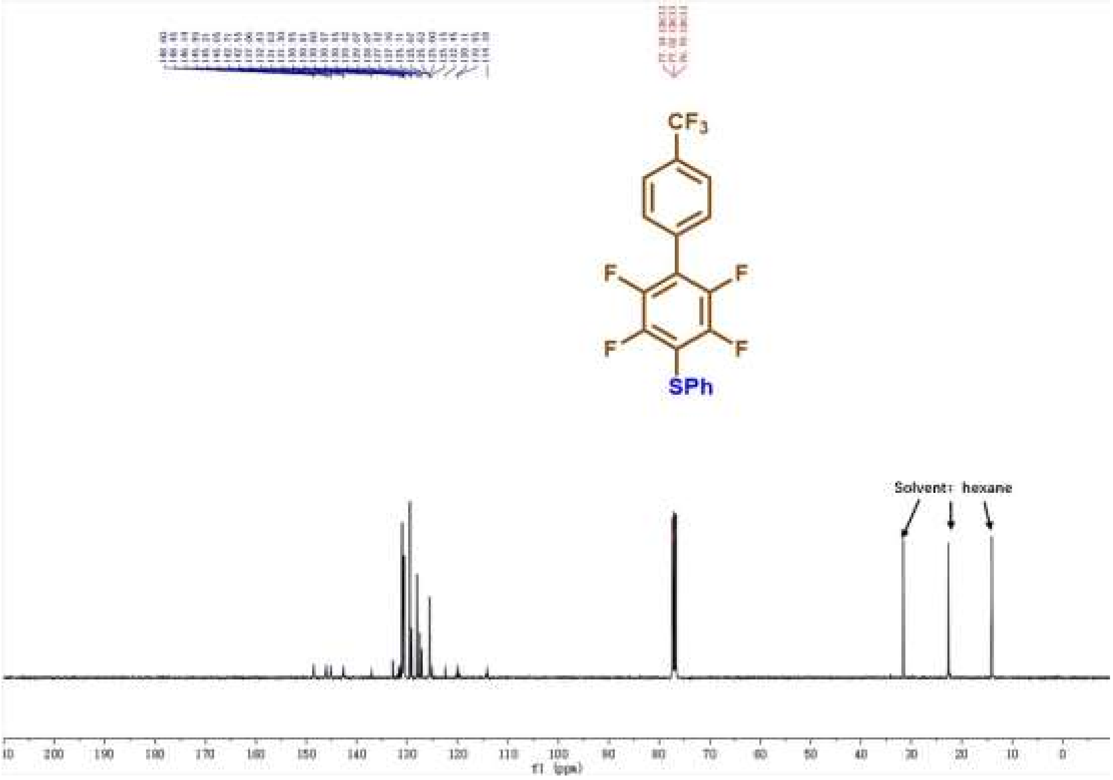

(2,3,4',5,6-pentafluoro-[1,1'-biphenyl]-4-yl)(phenyl)sulfane (**2h**)

^{1}H NMR ($CDCl_3$, 400 MHz)

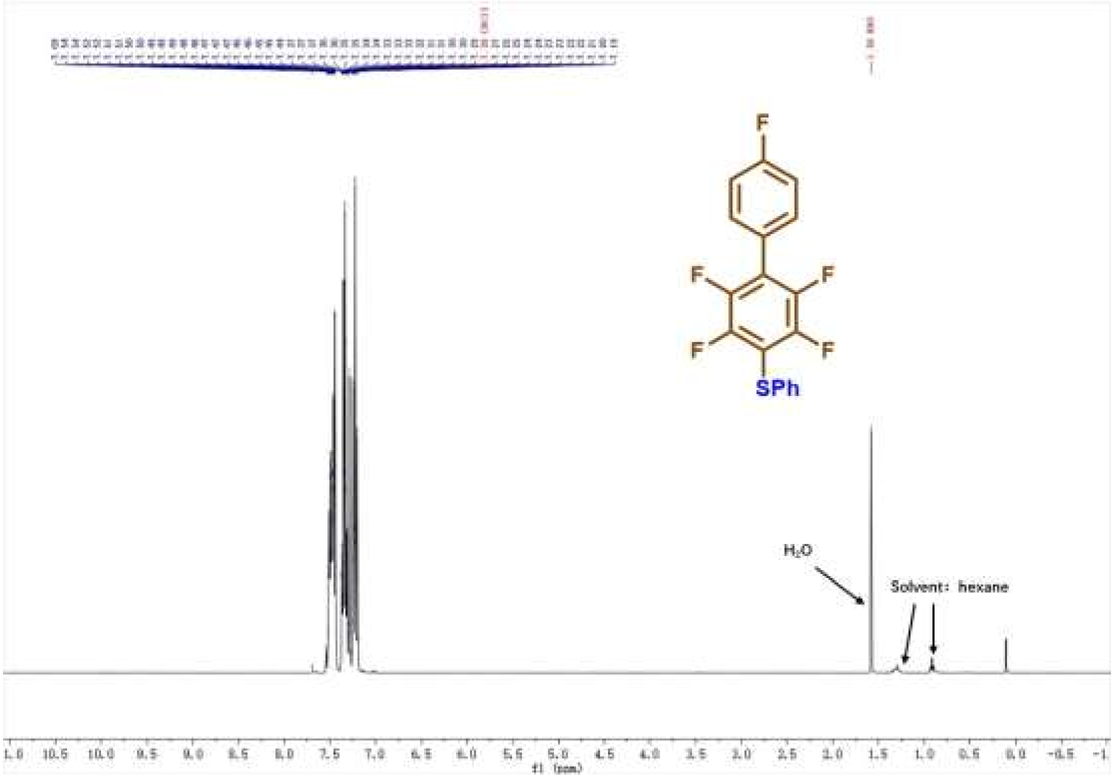

^{19}F NMR ($CDCl_3$, 376.5 MHz)

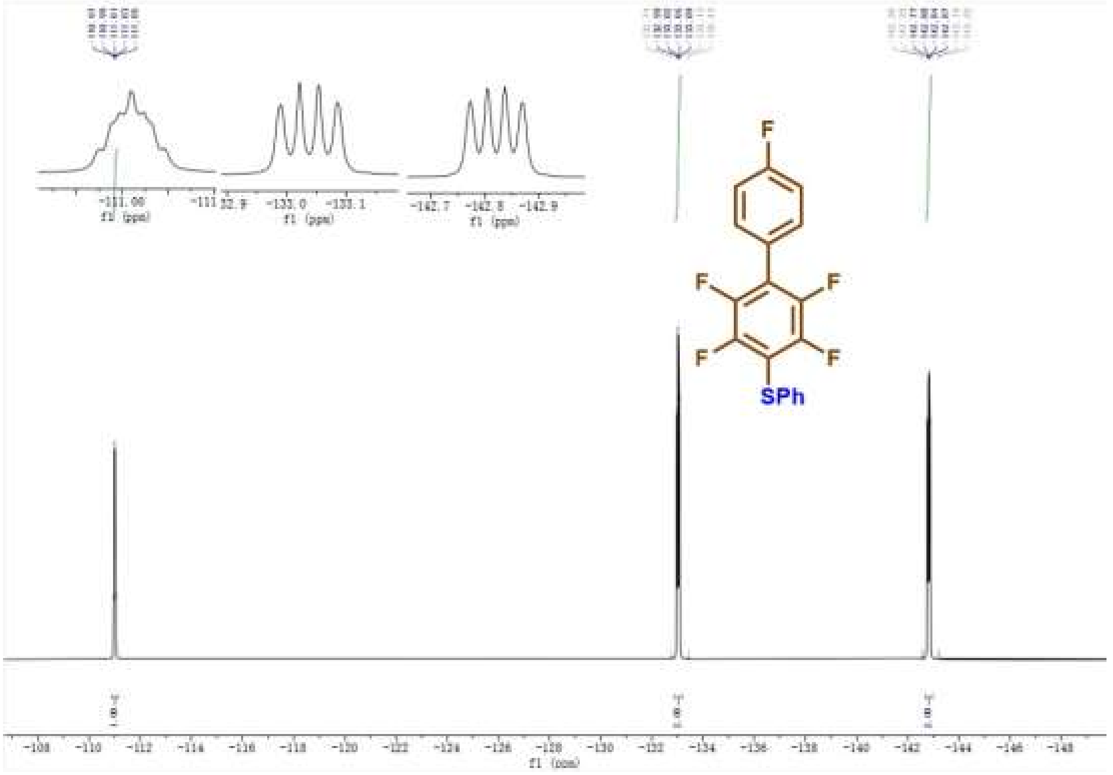

^{13}C NMR ($CDCl_3$, 101 MHz)

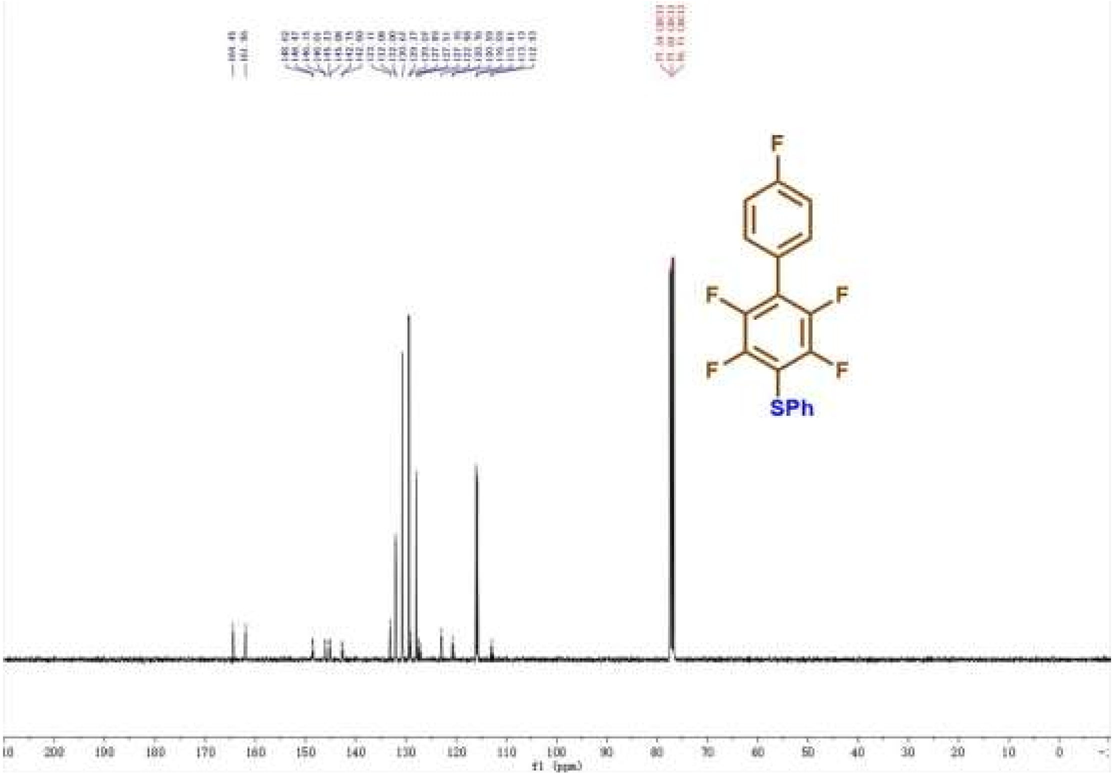

(perfluoro-1,4-phenylene)bis(phenylsulfane) (**2i**)

^1H NMR (C_6D_6, 400 MHz)

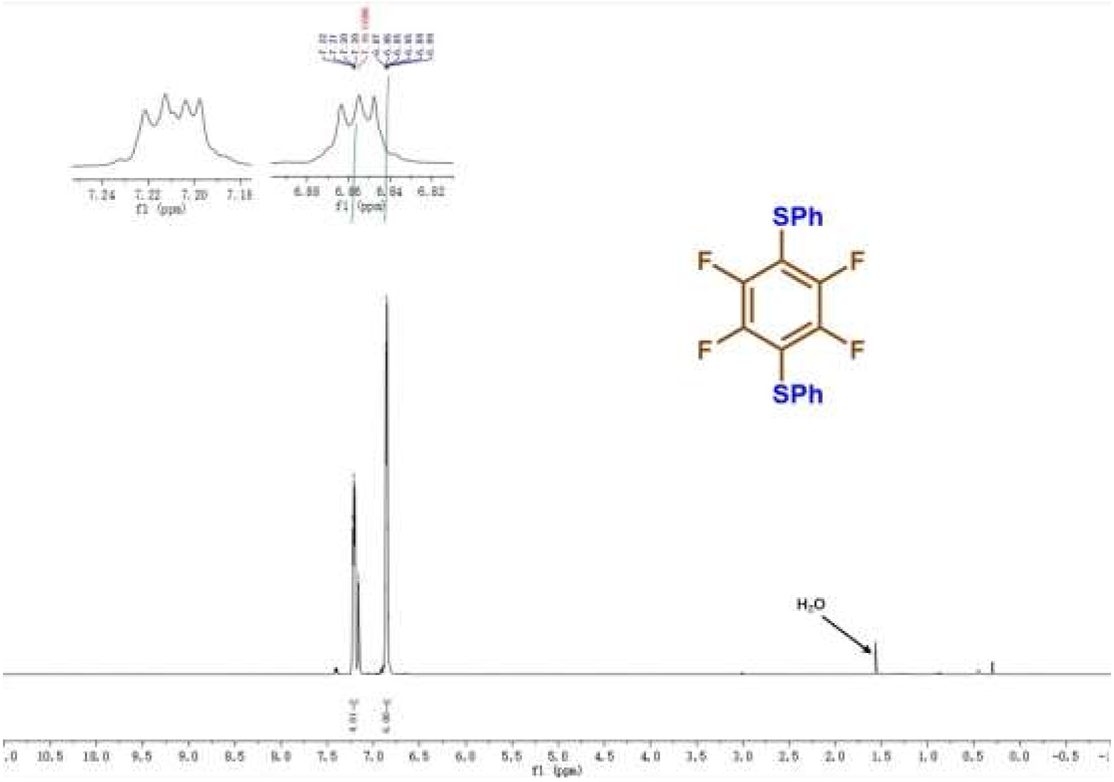

^{19}F NMR (C_6D_6, 376.5 MHz)

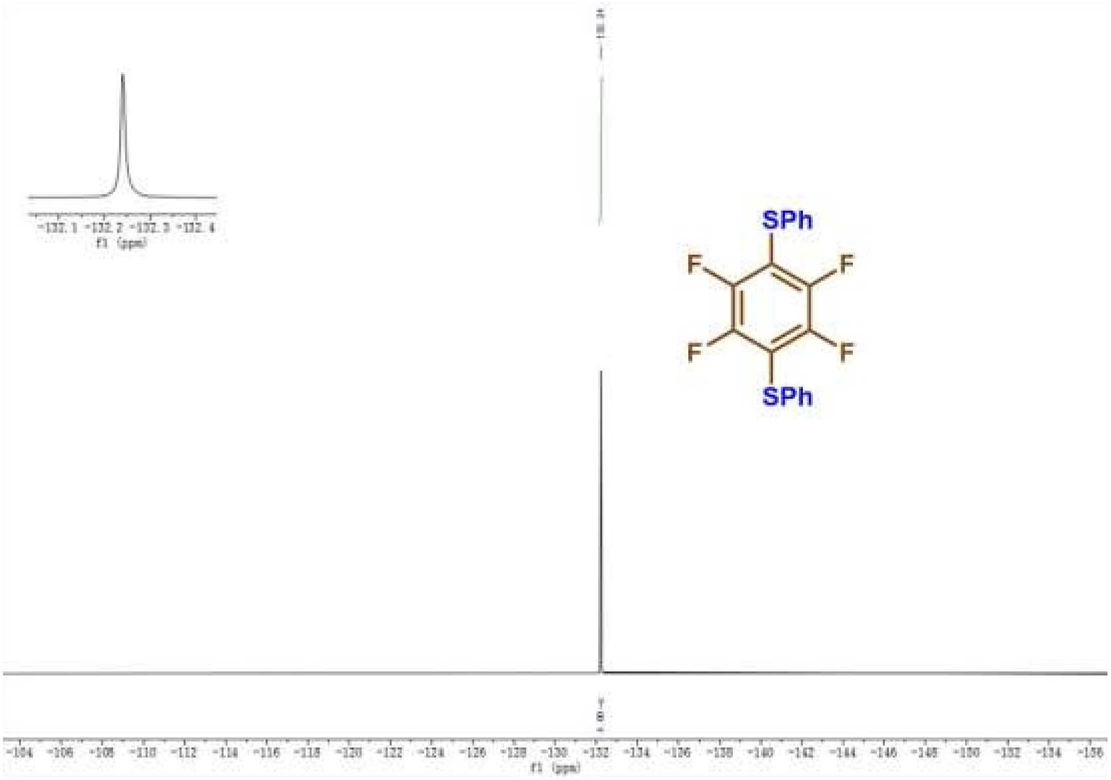

^{13}C NMR (C_6D_6, 101 MHz)

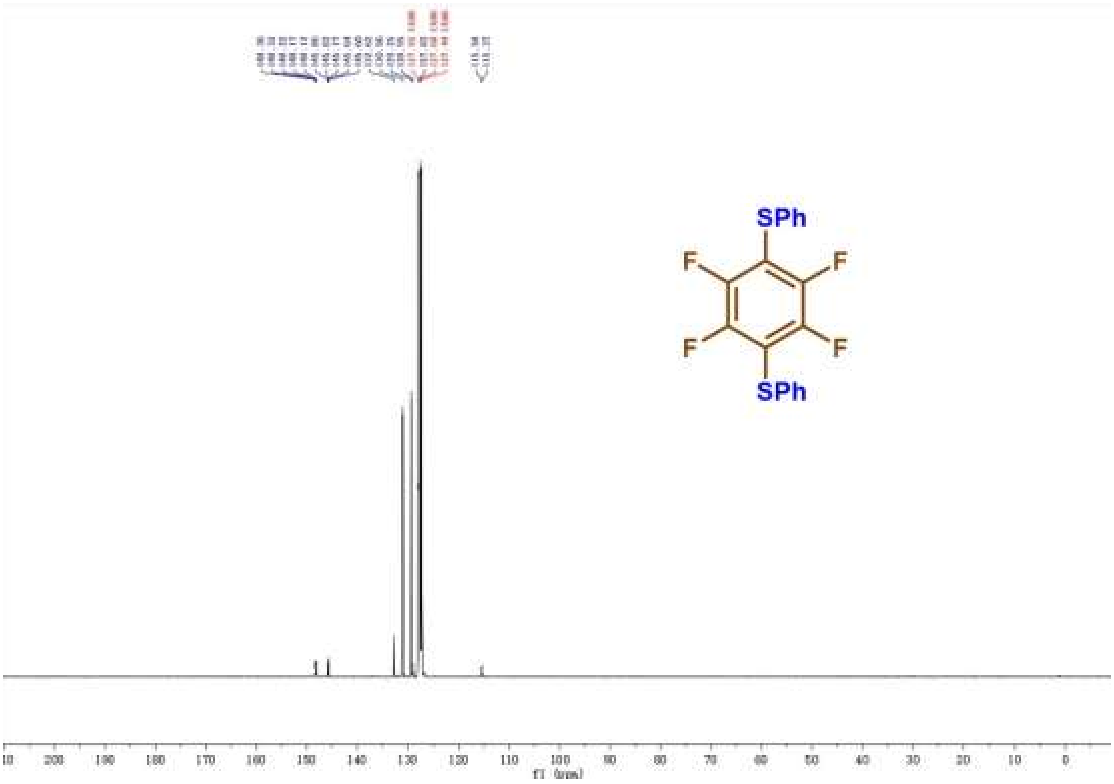

tris(2,3,5,6-tetrafluoro-4-(phenylthio)phenyl)phosphane (**2j**)

^1H NMR ($CDCl_3$, 400 MHz)

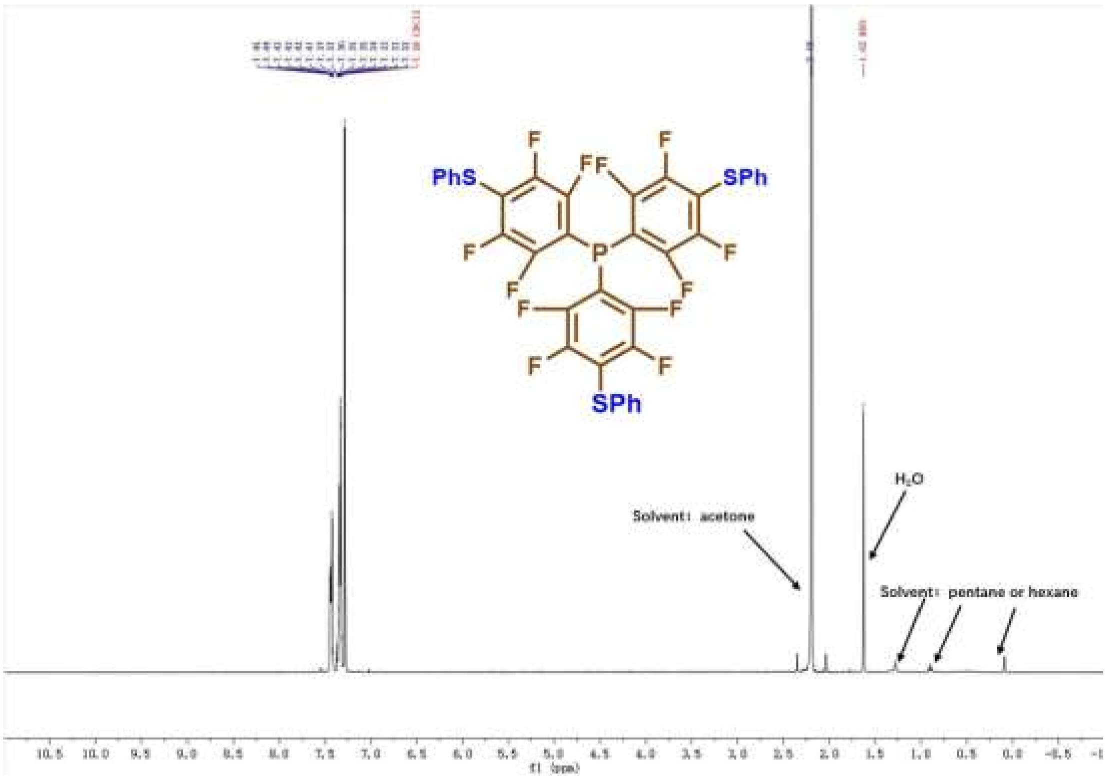

^{19}F NMR ($CDCl_3$, 376.5 MHz)

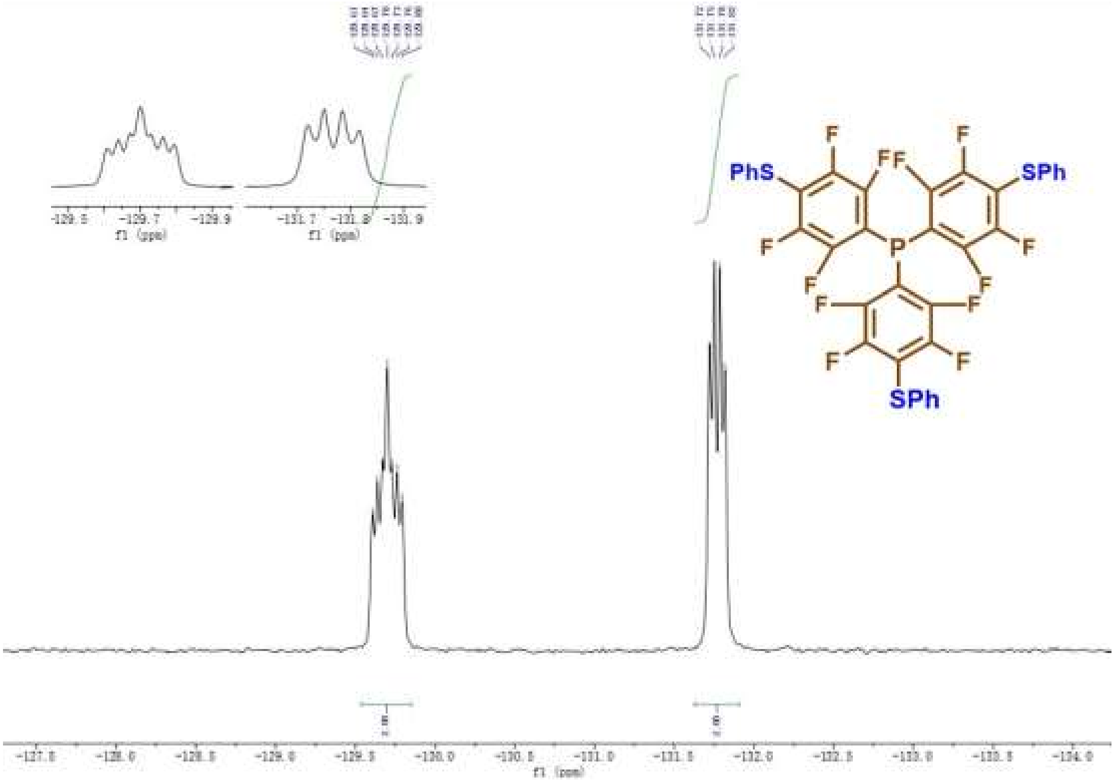

^{31}P NMR (162 MHz, $CDCl_3$):

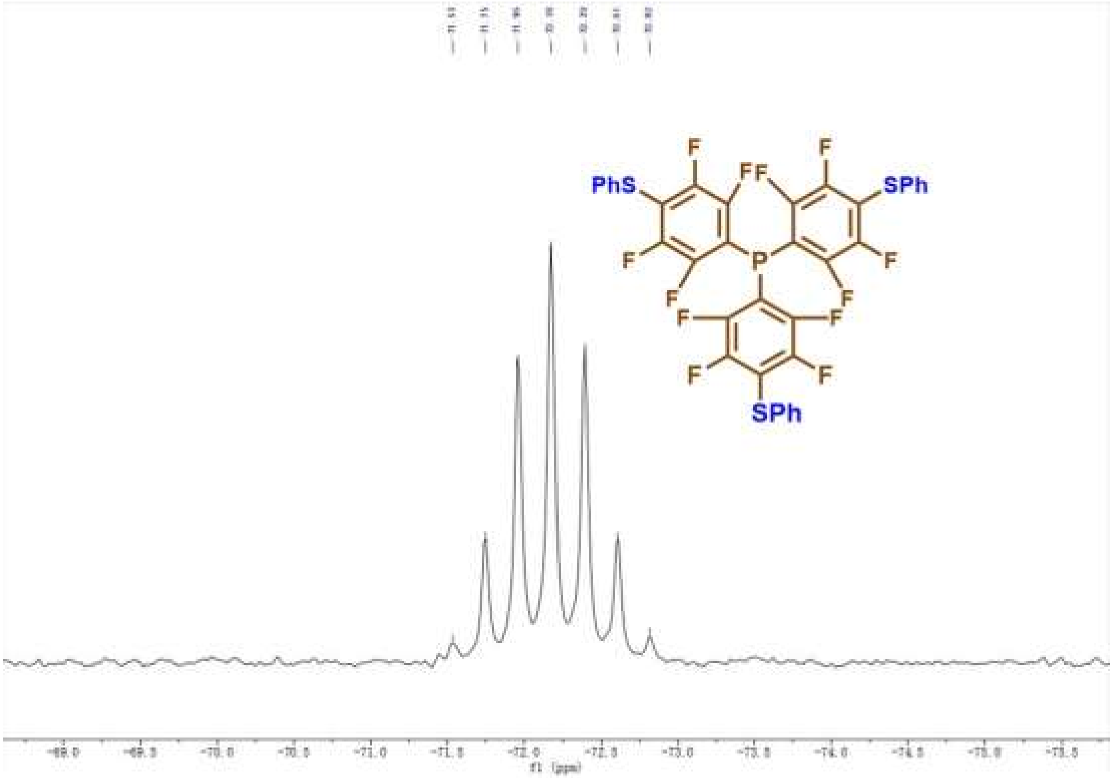

^{13}C NMR ($CDCl_3$, 101 MHz)

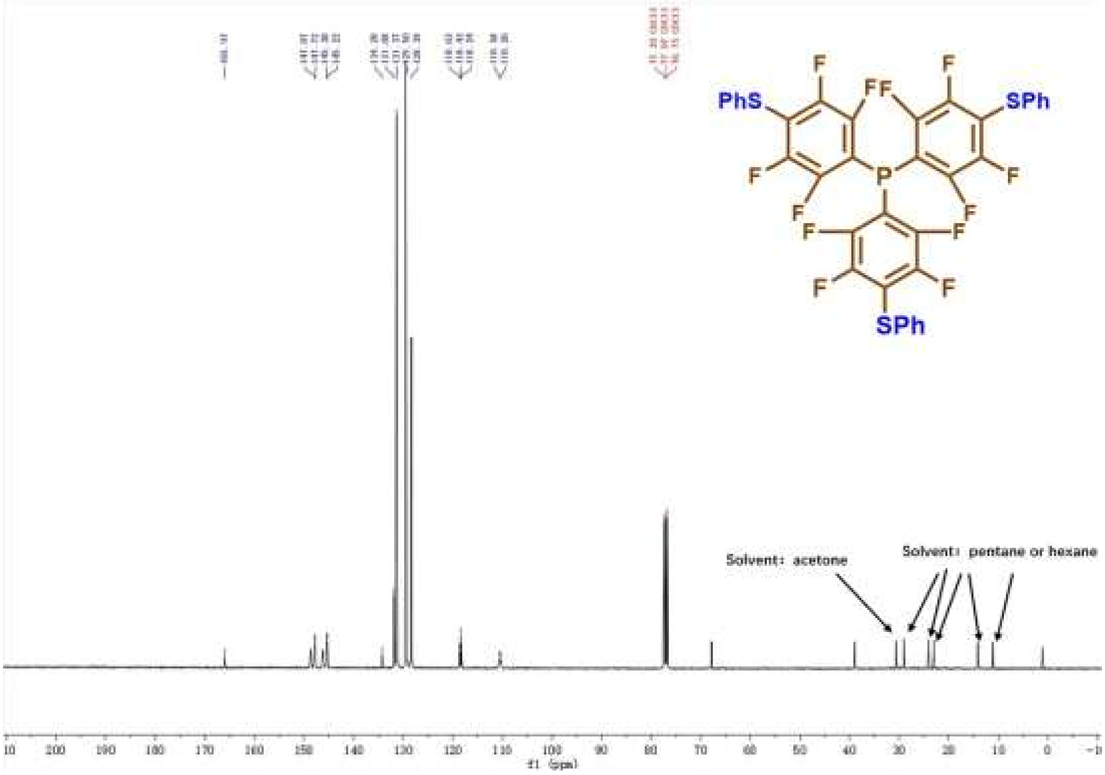

2-2-(2,3,5,6-tetrafluoro-4-(phenylthio)phenyl)pyridine (**2k**) and 2-(2,3,4,5-tetrafluoro-6-(phenylthio)phenyl)pyridine (**2k'**)

^{1}H NMR ($CDCl_3$, 400 MHz, "*"is the peak of (**2k**), isolated yield is 25%; "*"is the peak of (**2k'**), isolated yield is 5%)

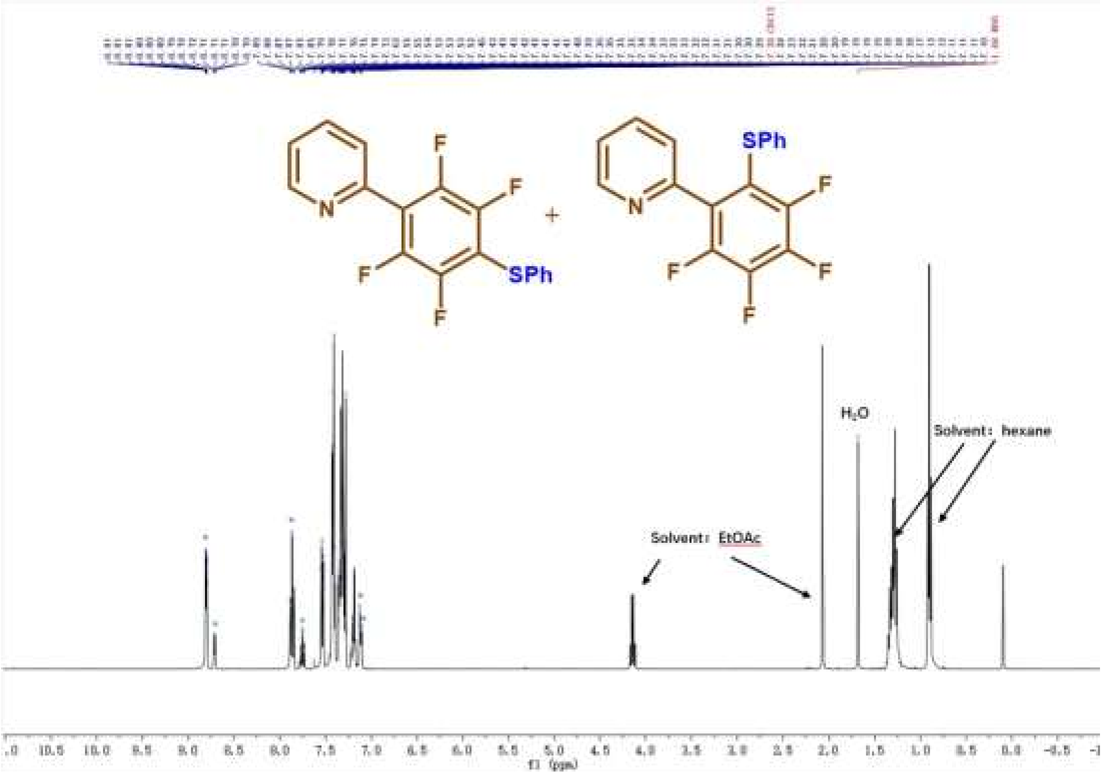

^{19}F NMR ($CDCl_3$, 376.5 MHz)

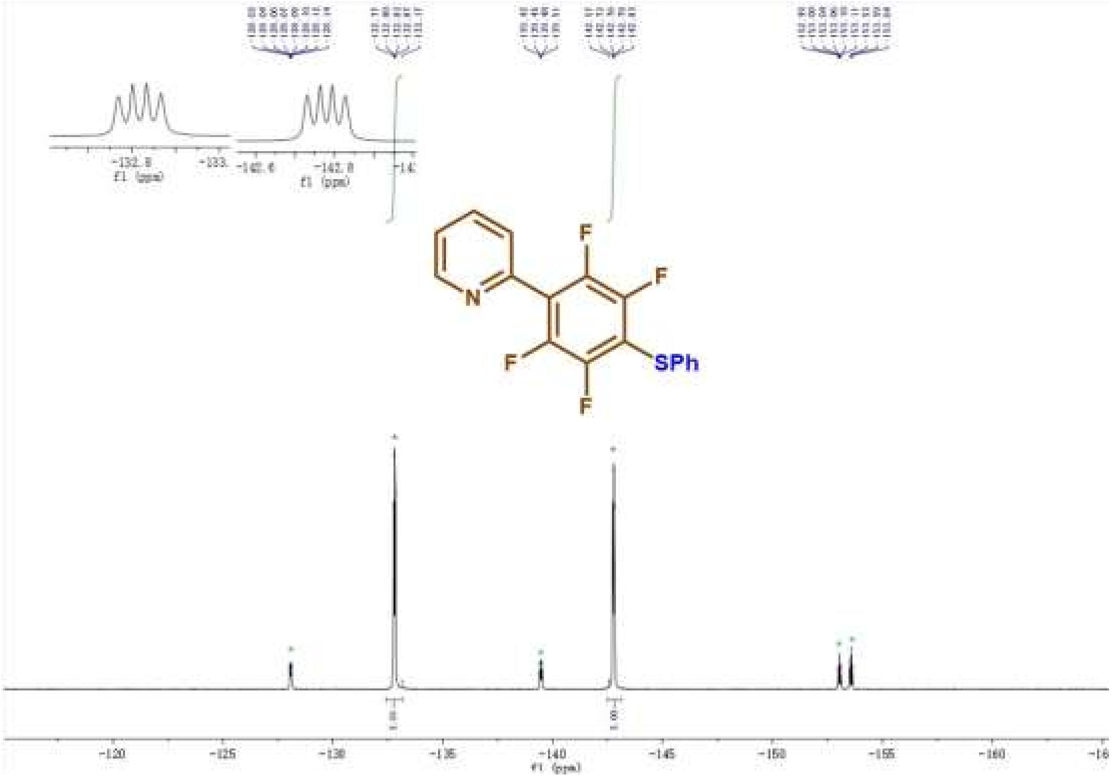

^{19}F NMR ($CDCl_3$, 376.5 MHz)

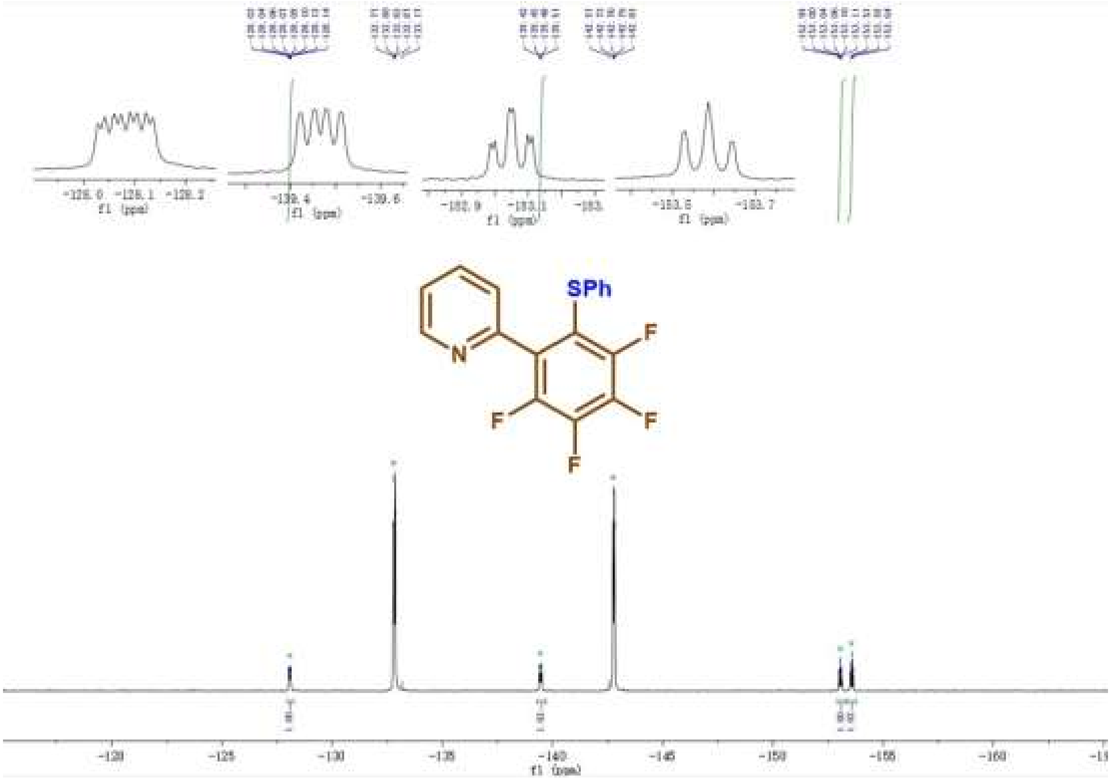

2-(2,3,5-trifluoro-4,6-bis(phenylthio)phenyl)pyridine (**2k''**) and 2-(2,4,5-trifluoro-3,6-bis(phenylthio)phenyl)pyridine (**2k'''**)

^{1}H NMR ($CDCl_3$, 400 MHz , "*"is the peak of (**2k''**), isolated yield is 40%; "*"is the peak of (**2k'''**), isolated yield is 10%)

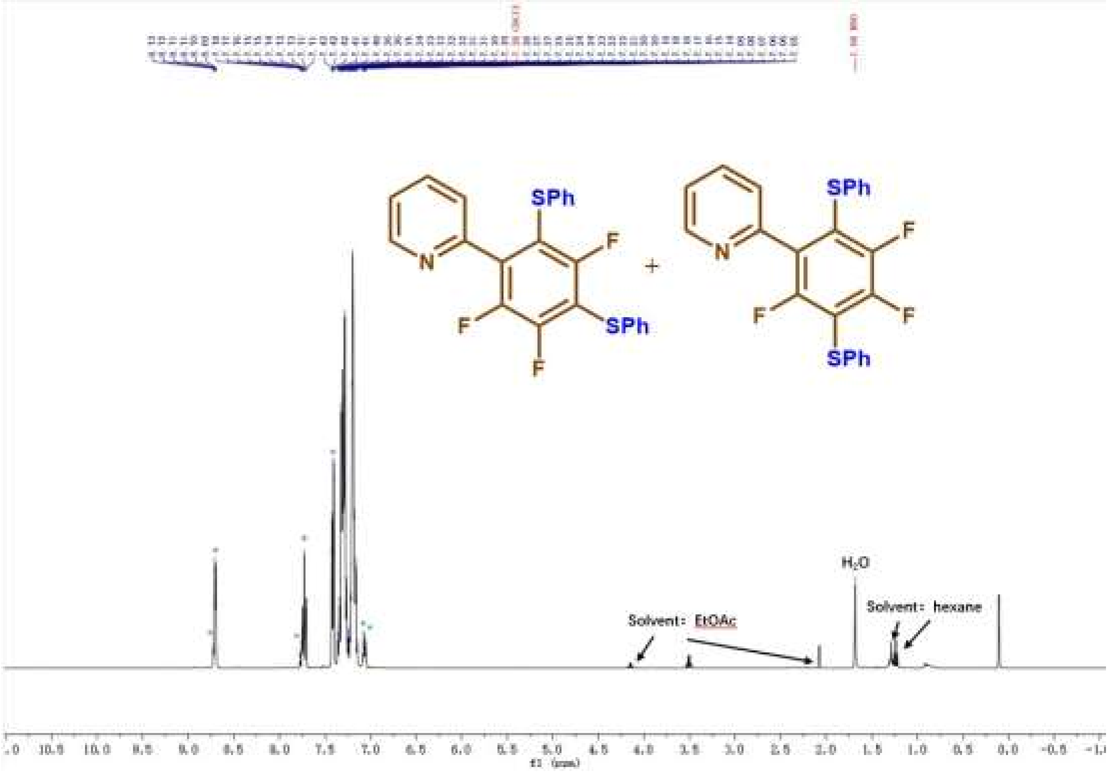

^{19}F NMR ($CDCl_3$, 376.5 MHz)

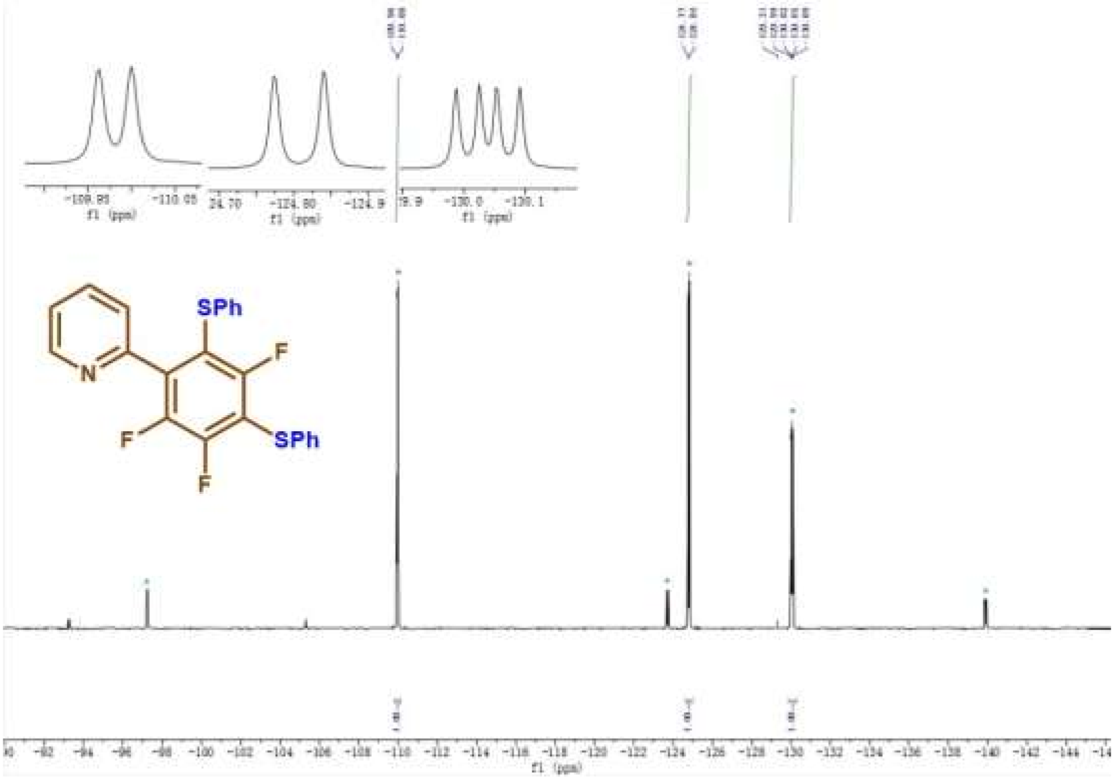

^{19}F NMR ($CDCl_3$, 376.5 MHz)

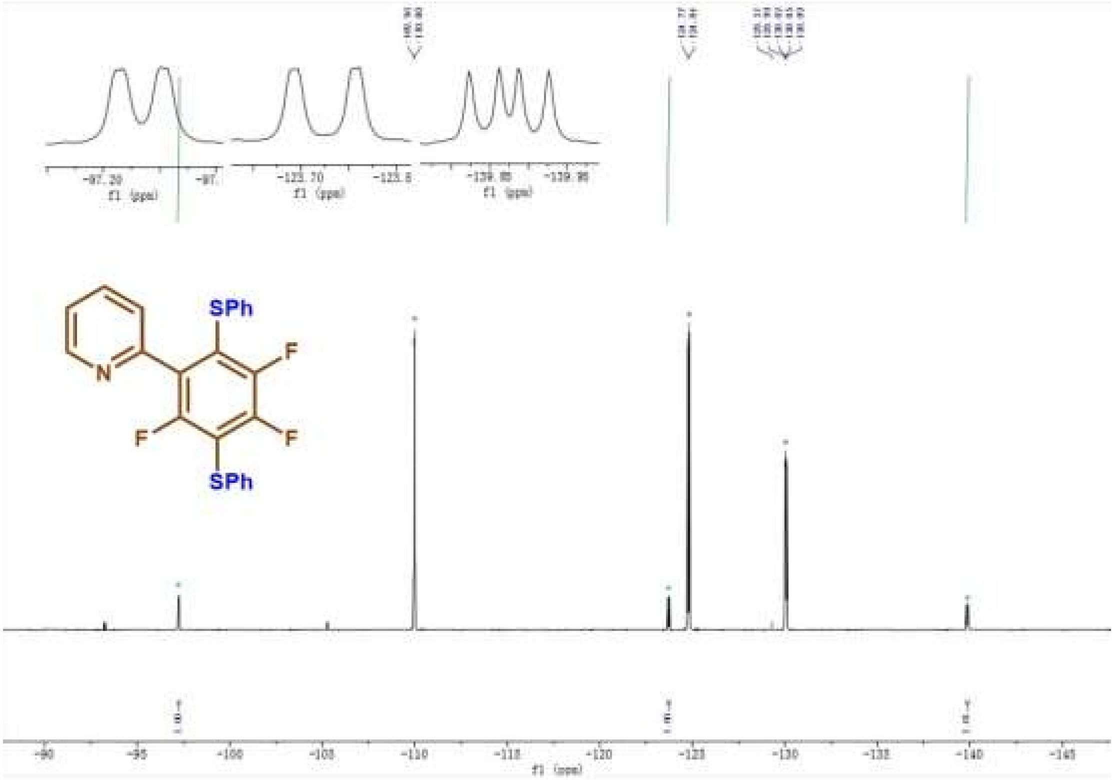

phenyl(3',5',6'-trifluoro-4,4''-bis(trifluoromethyl)-[1,1':2',1''-terphenyl]-4'-yl)sulfane (**2l**)

^{1}H NMR ($CDCl_3$, 400 MHz)

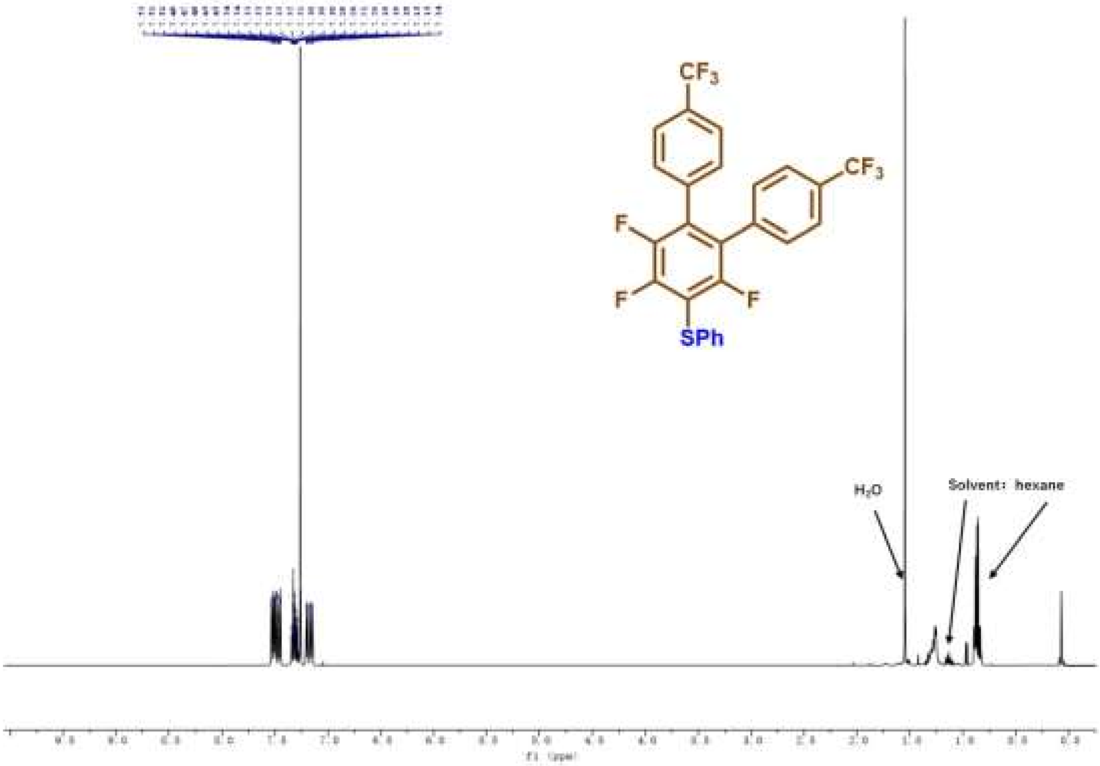

^{19}F NMR ($CDCl_3$, 376.5 MHz)

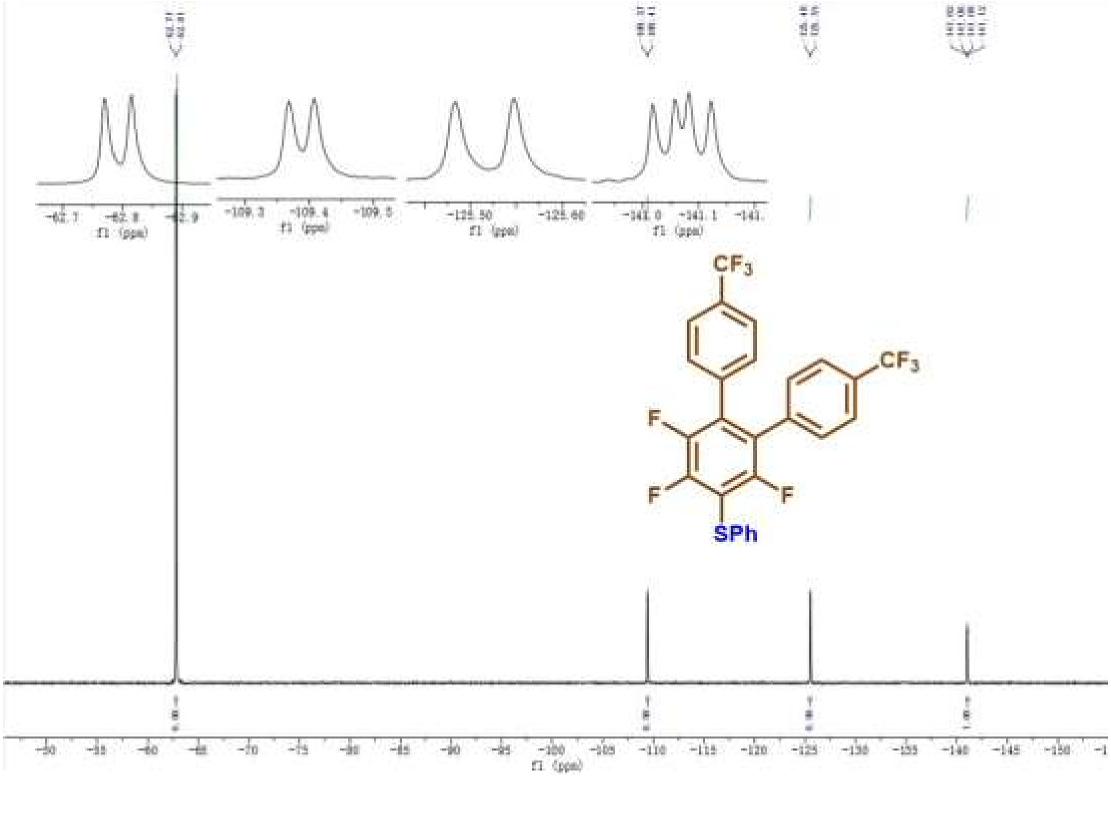

^{13}C NMR ($CDCl_3$, 101 MHz)

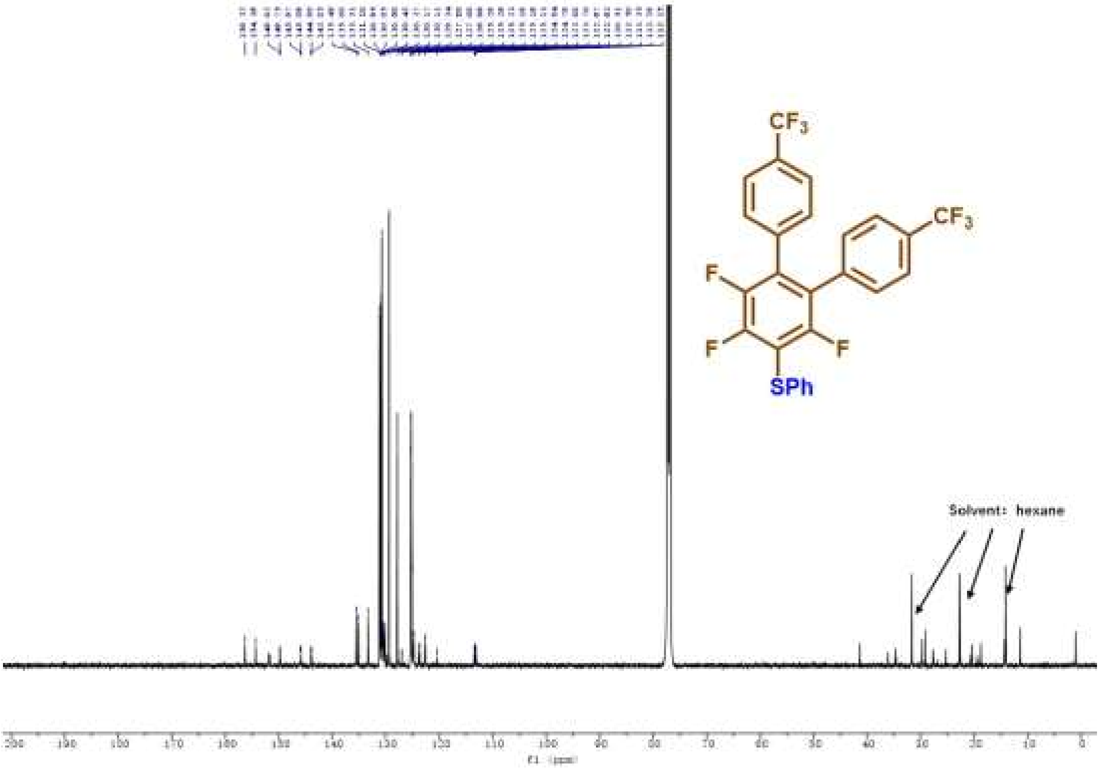

(2,3,4',5,6-pentafluoro-[1,1'-biphenyl]-4-yl)(phenyl)sulfane (**2l’**)

^{1}H NMR ($CDCl_3$, 400 MHz)

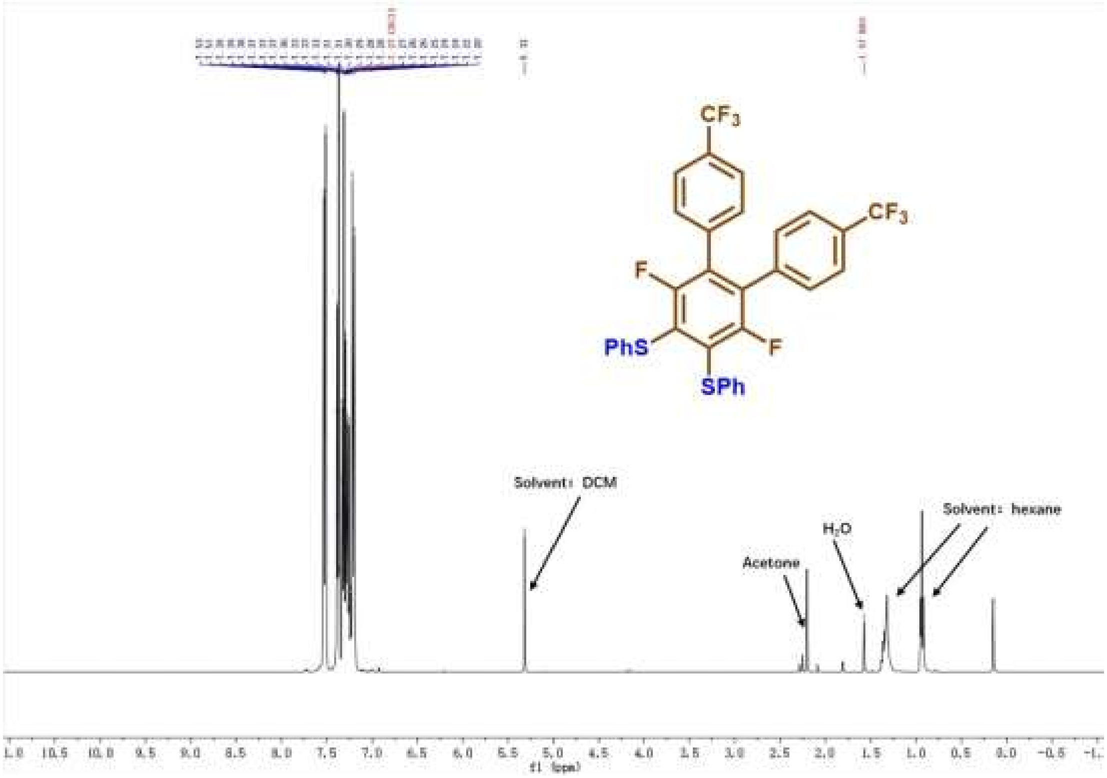

^{19}F NMR ($CDCl_3$, 376.5 MHz)

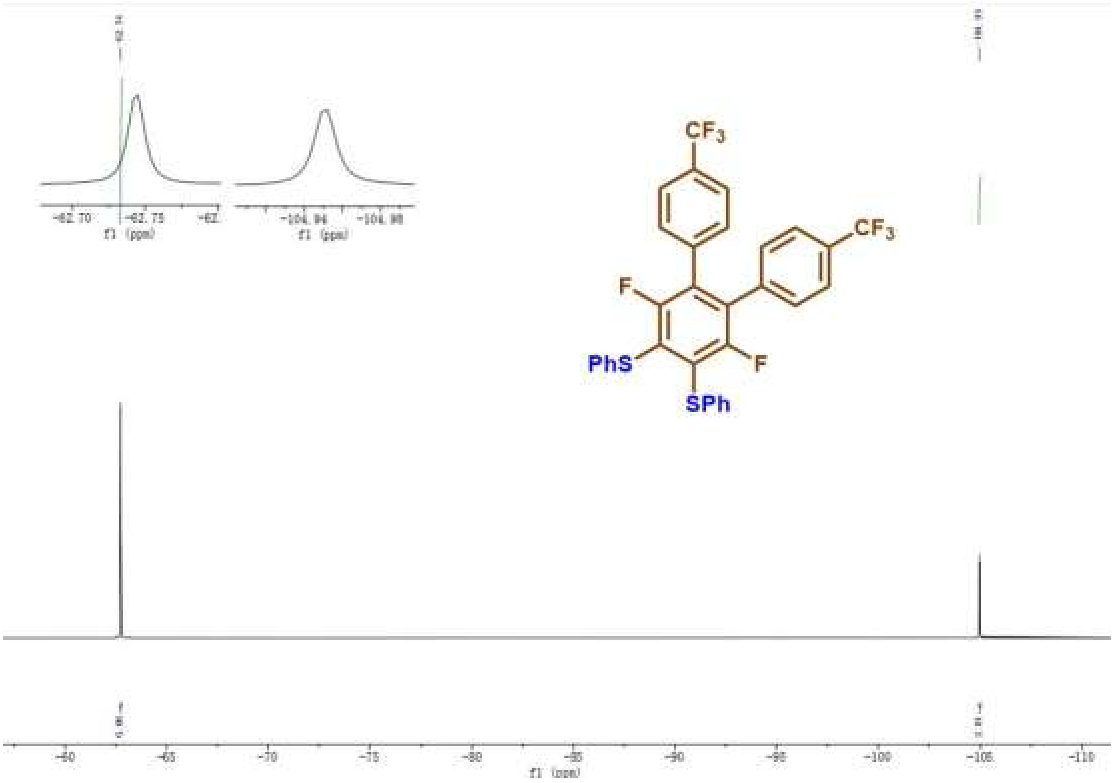

^{13}C NMR ($CDCl_3$, 101 MHz)

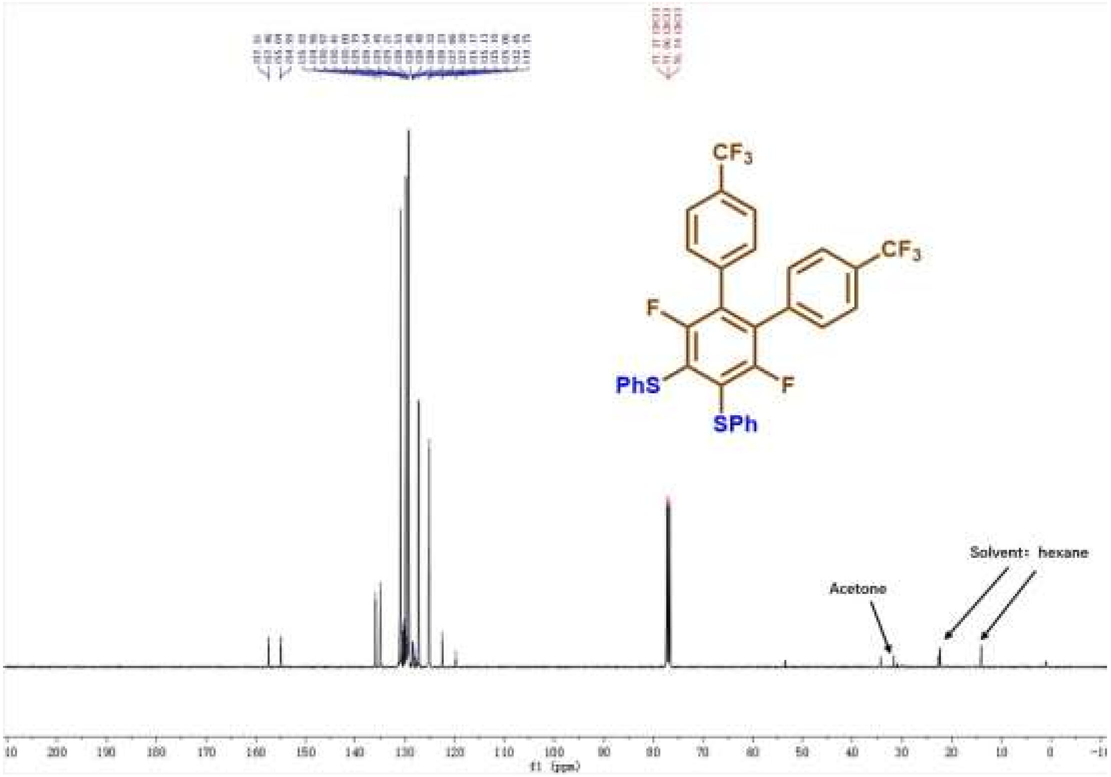

3,5-dichloro-2,6-difluoropyridine (**5b**)

^{1}H NMR (CDCl$_3$, 400 MHz)

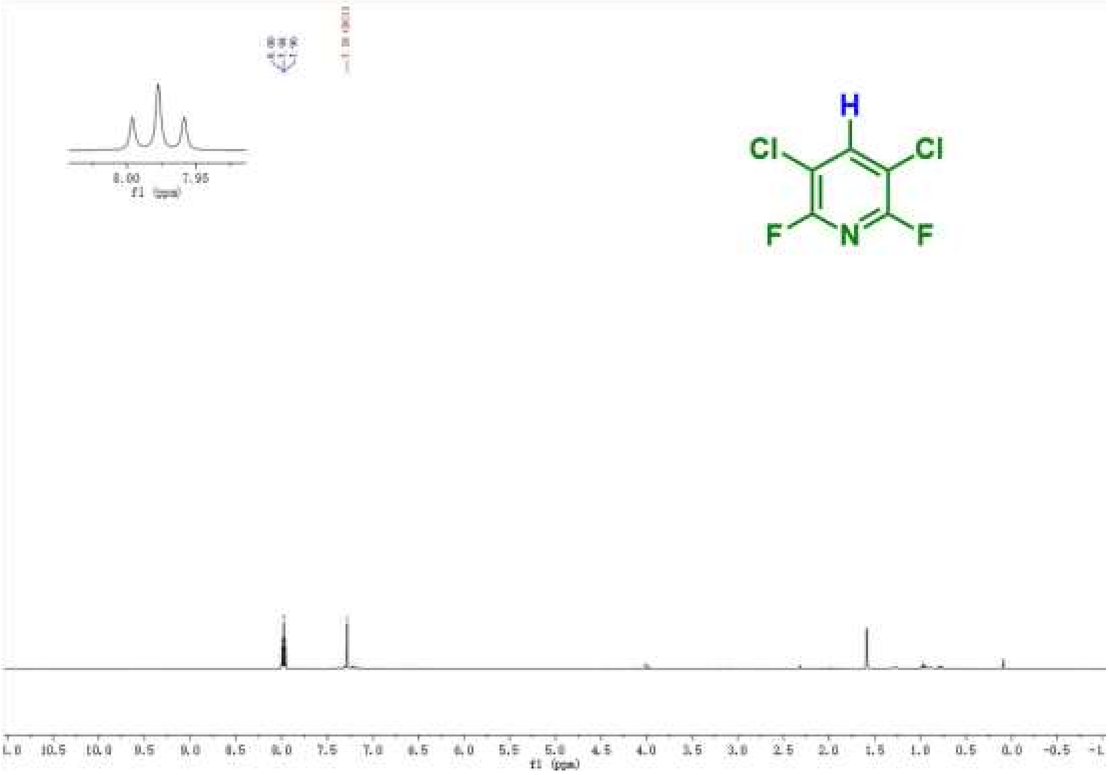

^{19}F NMR (CDCl$_3$, 376.5 MHz)

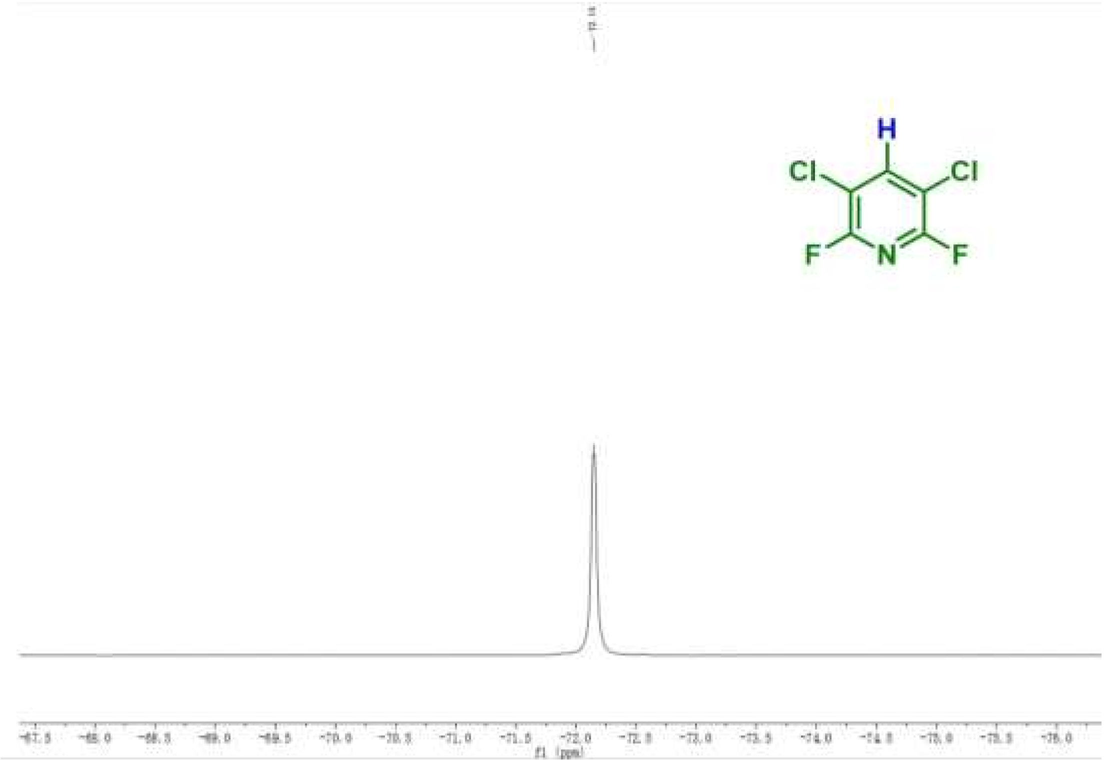

^{13}C NMR ($CDCl_3$, 101 MHz)

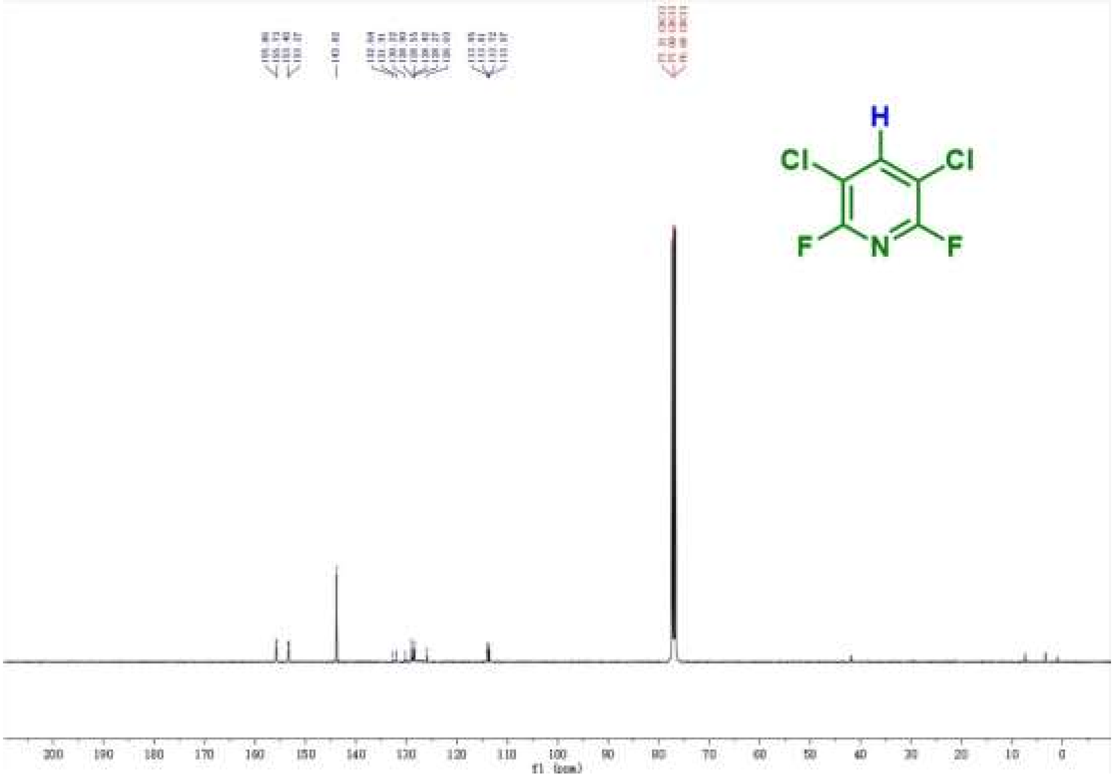

2,5-difluoroisophthalonitrile (**5g**)

^{1}H NMR ($CDCl_3$, 400 MHz)

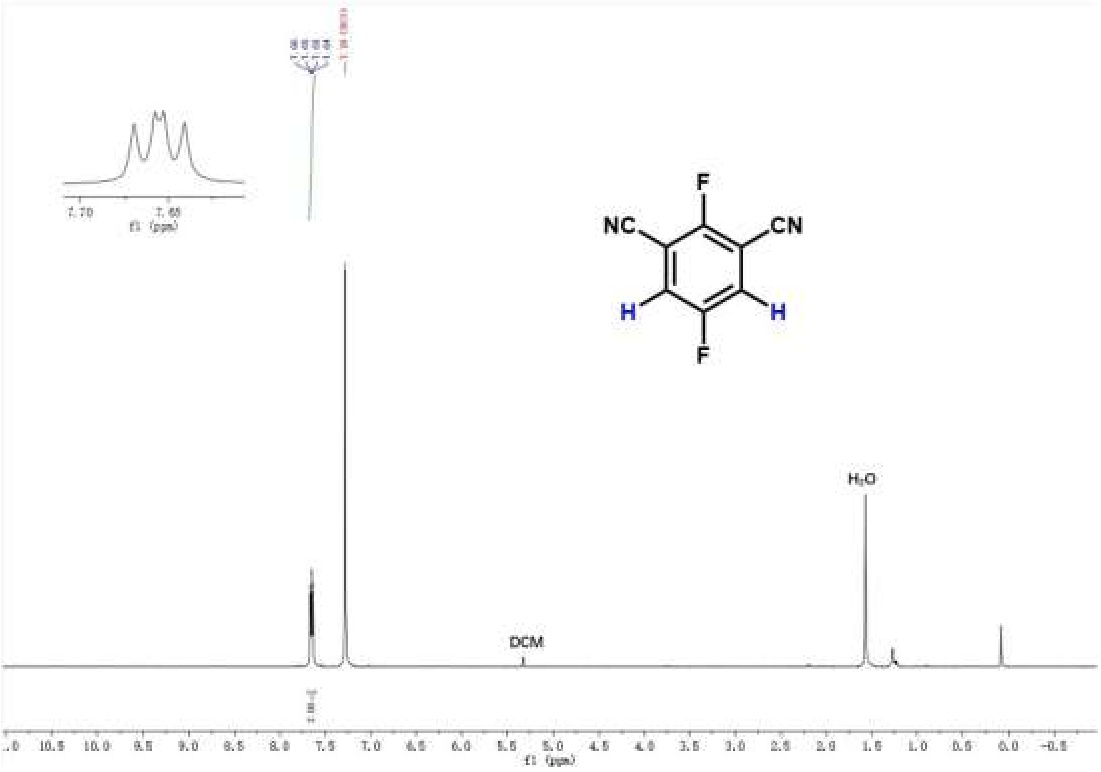

^{19}F NMR ($CDCl_3$, 376.5 MHz)

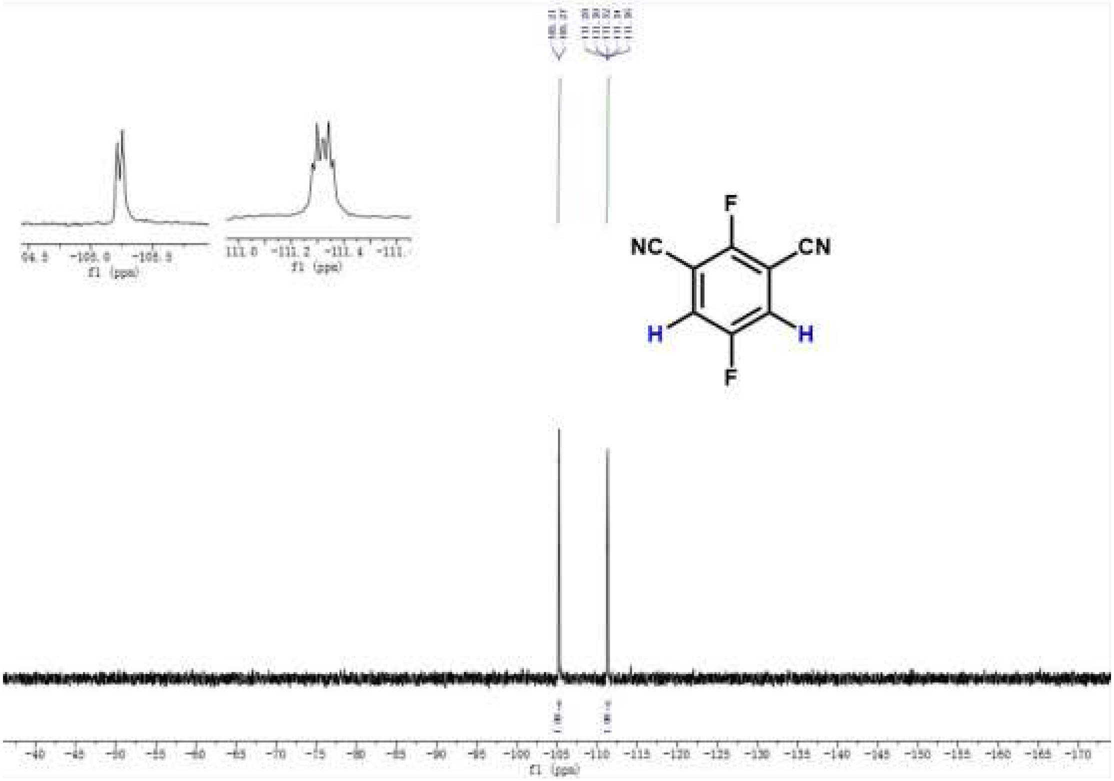

^{13}C NMR ($CDCl_3$, 101 MHz)

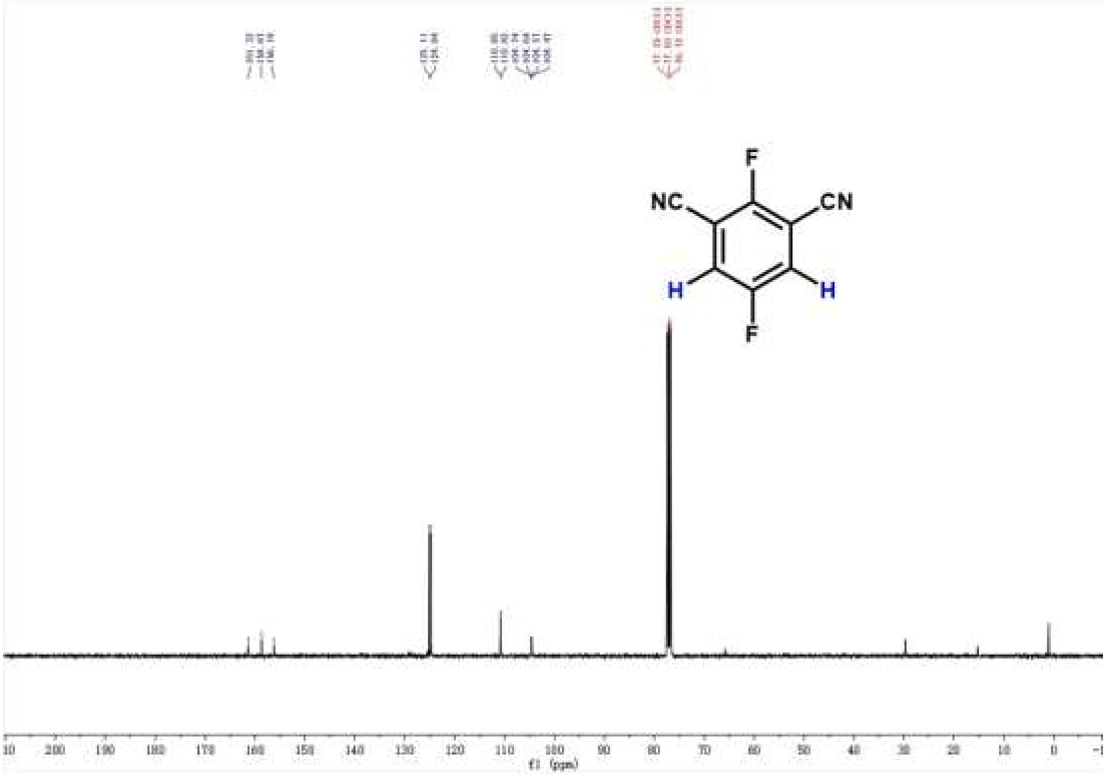

7.3. ^{19}F-NMR Spectra for Determining the NMR Yields of Defluorofunctionalisation Reactions

- Thiodefluorination of HDF of perfluoroprop-1-ene

^{19}F NMR ($PhCF_3$ with a sealed glass capillary containing C_6D_6, 376.5 MHz, "*" is the peak of (Z)-(perfluoroprop-1-en-1-yl)(phenyl)sulfane (**2m**), "*" is the peak of (E)-(perfluoroprop-1-en-1-yl)(phenyl)sulfane (**2m'**), "*" is the peak of (1,1,2,3,3,3-hexafluoropropyl)(phenyl)sulfane (**2m''**), "*" is the peak of ^{Mes}BDIBF$_2$)

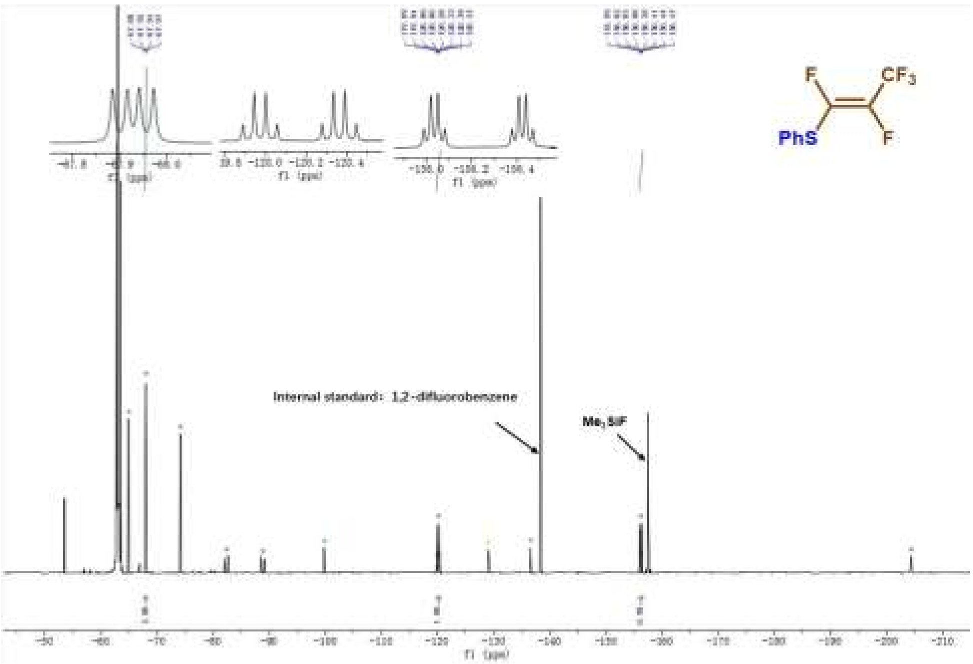

- Thiodefluorination of perfluoroprop-1-ene

^{19}F NMR ($PhCF_3$ with a sealed glass capillary containing C_6D_6, 376.5 MHz"*" is the peak of (Z)-(perfluoroprop-1-en-1-yl)(phenyl)sulfane (**2m**), "*" is the peak of (E)-(perfluoroprop-1-en-1-yl)(phenyl)sulfane (**2m'**), "*" is the peak of (1,1,2,3,3,3-hexafluoropropyl)(phenyl)sulfane (**2m''**), "*" is the peak of ^{Mes}BDIBF$_2$)

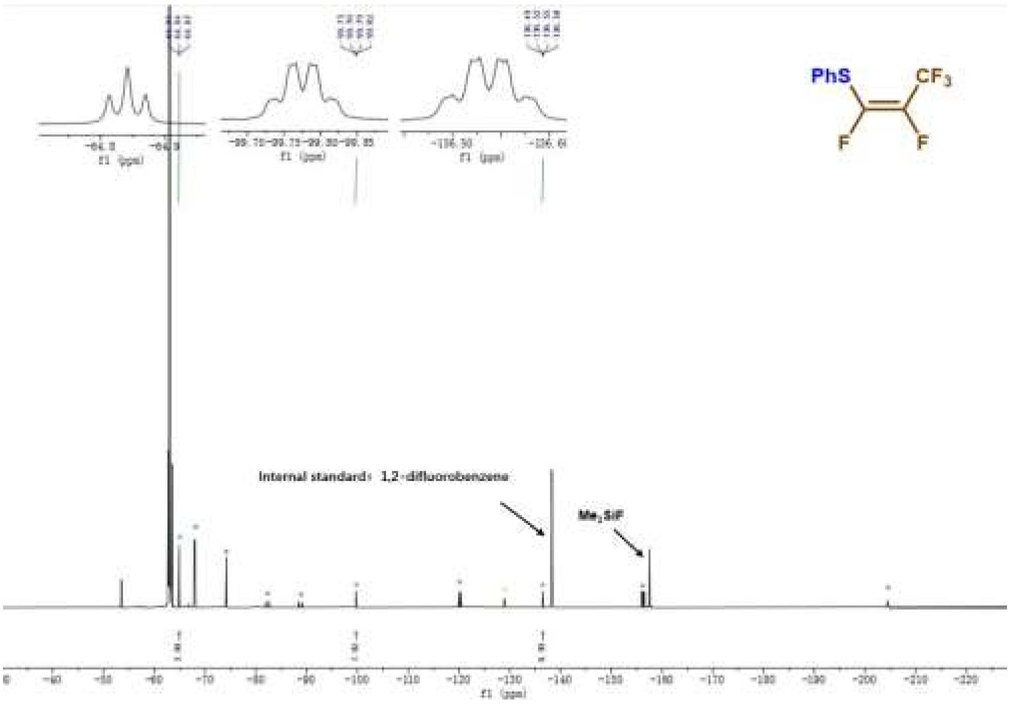

- Thiodefluorination of perfluoroprop-1-ene

^{19}F NMR ($PhCF_3$ with a sealed glass capillary containing C_6D_6, 376.5 MHz, “*” is the peak of (Z)-(perfluoroprop-1-en-1-yl)(phenyl)sulfane (**2m**), “*” is the peak of (E)-(perfluoroprop-1-en-1-yl)(phenyl)sulfane (**2m’**), “*” is the peak of (1,1,2,3,3,3-hexafluoropropyl)(phenyl)sulfane (**2m’’**), “*” is the peak of $^{Mes}BDIBF_2$)

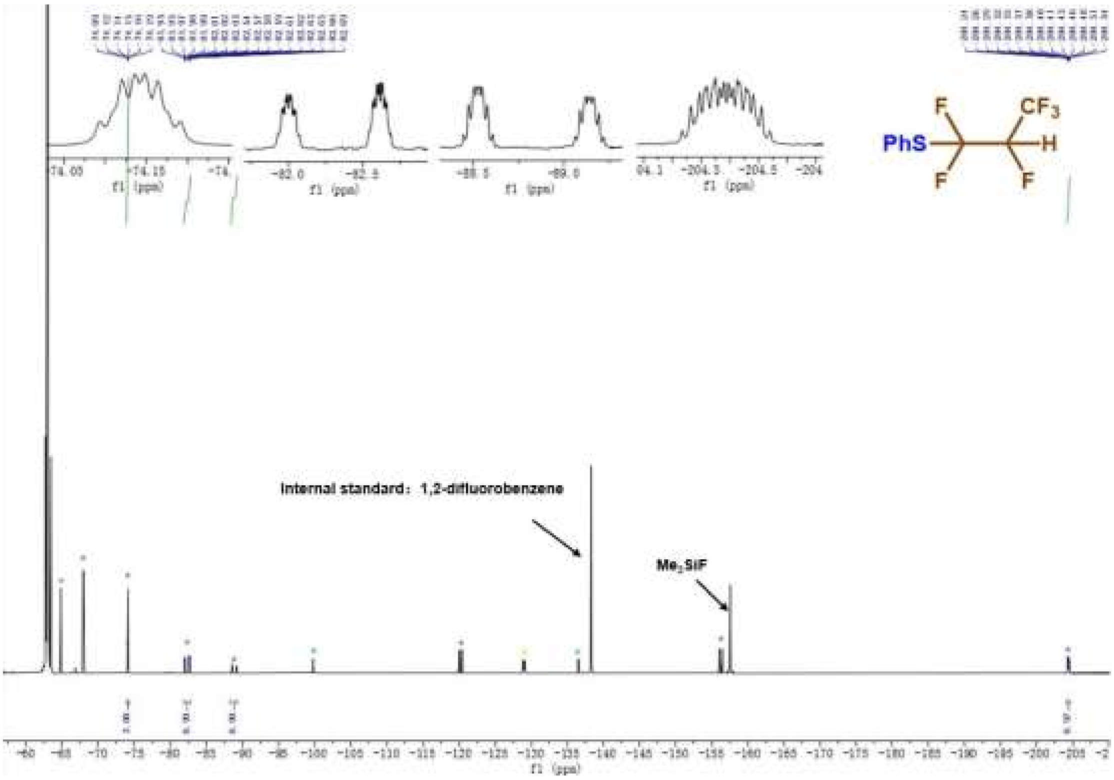

- HDF of pentafluoropyridine

^{19}F NMR (PhF with a sealed glass capillary containing C_6D_6, 376.5 MHz, “*”is the peak of 2,3,5,6-Tetrafluoropyridine (**5a**))

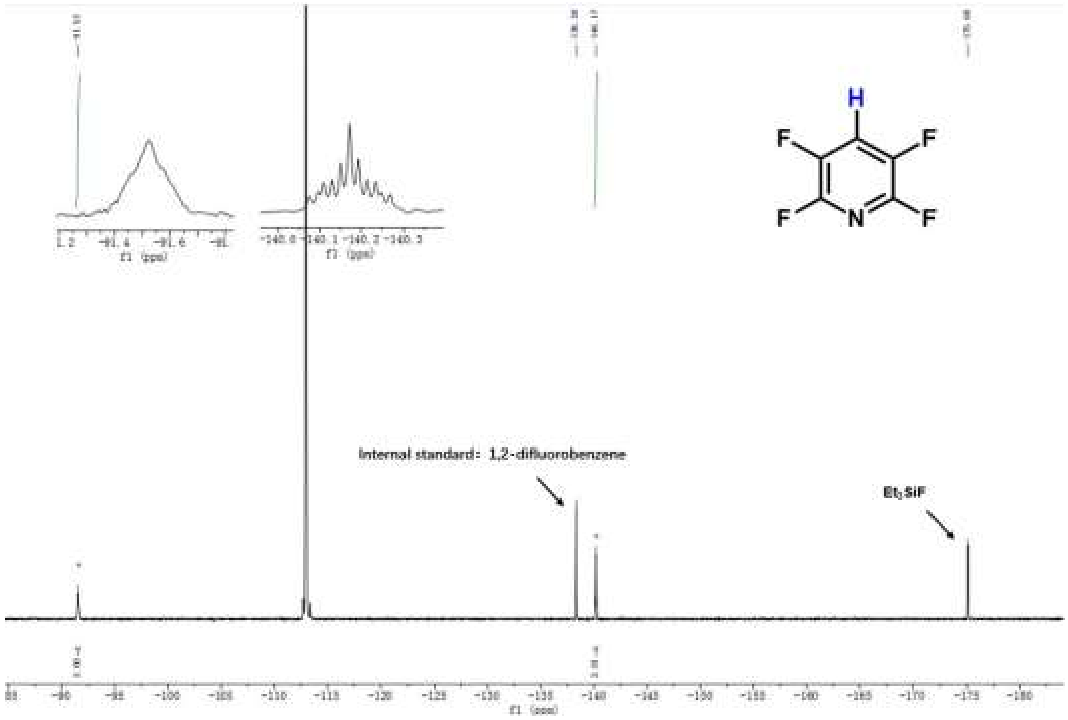

● HDF of octafluorotoluene

^{19}F NMR ($PhCF_3$ with a sealed glass capillary containing C_6D_6, 376.5 MHz, "*"is the peak of 1,2,4,5-Tetrafluoro-3-(trifluoromethyl)benzene (**5c**))

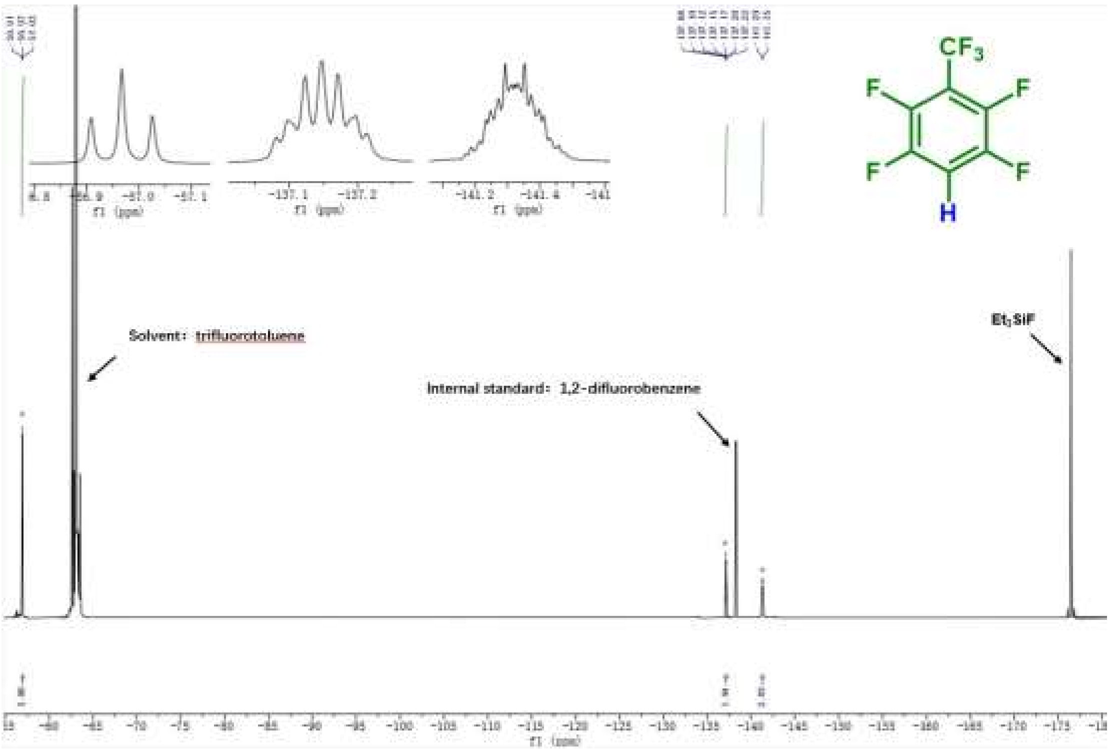

● HDF of 2,3,4,5,6-pentafluorobenzoic acid

^{19}F NMR ($PhCF_3$ with a sealed glass capillary containing C_6D_6, 376.5 MHz, "*"is the peak of 2,3,5,6-tetrafluorobenzoic acid (**5d**), "*"is the peak of 2,3,4,5,6-pentafluorobenzoic acid)

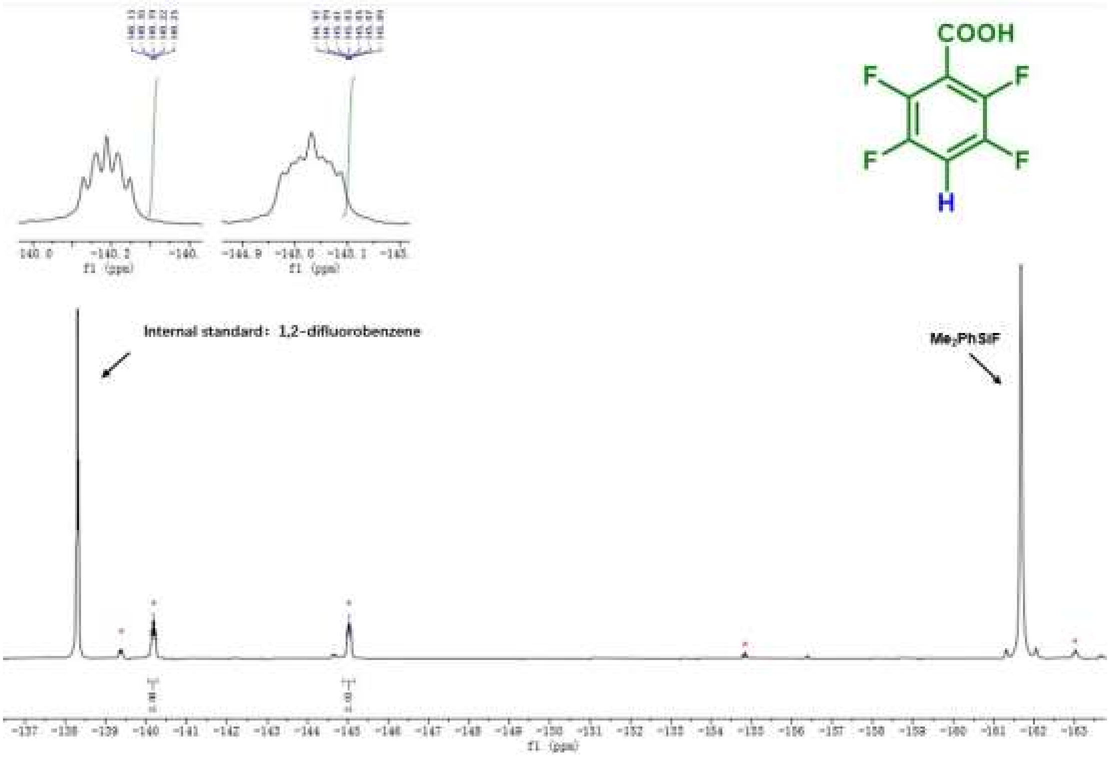

- HDF of 2,3,4,5,6-pentafluorocyanobenzene

^{19}F NMR (PhF with a sealed glass capillary containing C_6D_6, 376.5 MHz, "*"is the peak of 2,3,5,6-tetrafluorobenzonitrile (**5e**), "*"is the peak of 2,3,4,5,6-pentafluorocyanobenzene)

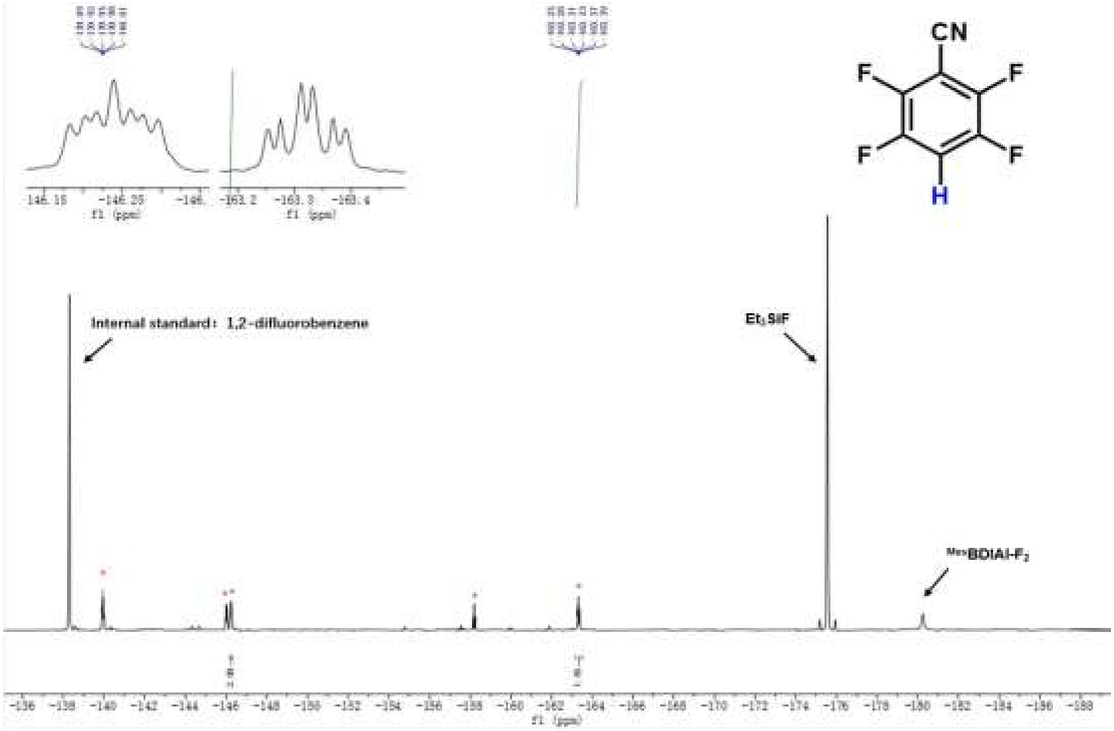

- HDF of 2,3,4,5,6-pentafluorobenzoate

^{19}F NMR ($PhCF_3$ with a sealed glass capillary containing C_6D_6, 376.5 MHz, "*"is the peak of 2,3,5,6-tetrafluorobenzoate (**5f**), "*"is the peak of 2,3,4,5,6-pentafluorobenzoate)

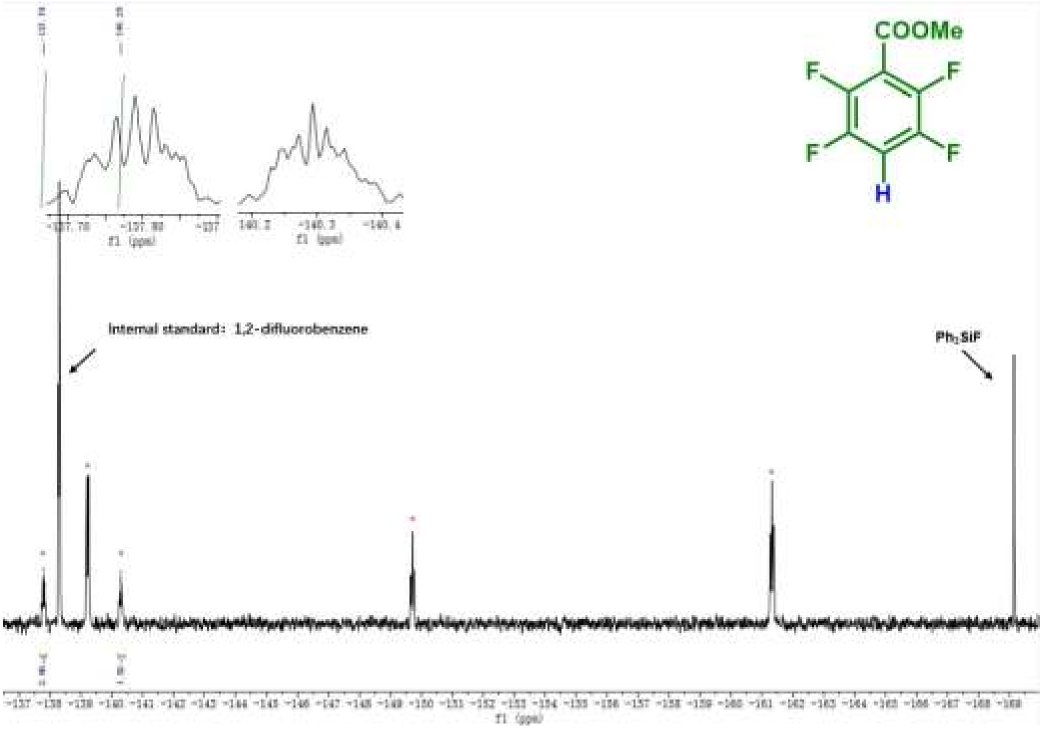

● HDF of 2,3,4,5,6-pentafluoronitrobenzene

^{19}F NMR (PhF with a sealed glass capillary containing C_6D_6, 376.5 MHz, "*"is the peak of 1,2,4,5-tetrafluoro-3-nitrobenzene (**5h**), "*"is the peak of 1,2,3,4-tetrafluoro-3-nitrobenzene (**5h'**), "*"is the peak of 2,3,4,5,6-pentafluoronitrobenzene)

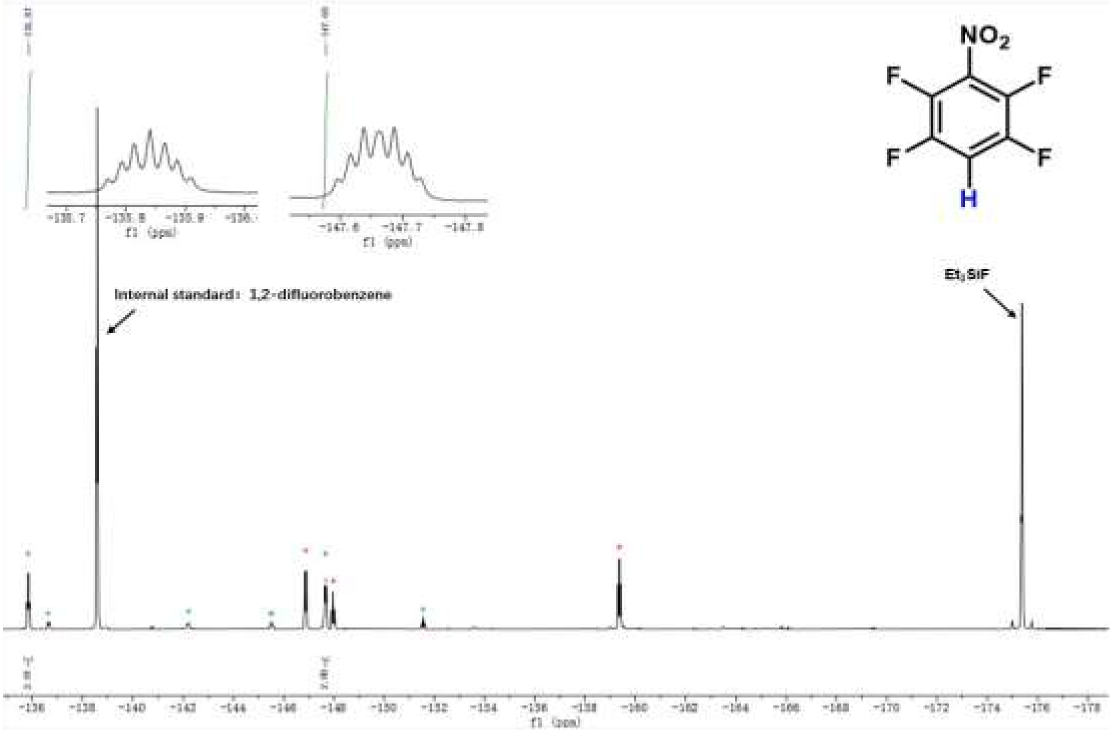

● HDF of 2,3,4,5,6-pentafluoronitrobenzene

^{19}F NMR (PhF with a sealed glass capillary containing C_6D_6, 376.5 MHz, "*"is the peak of 1,2,4,5-tetrafluoro-3-nitrobenzene (**5h**), "*"is the peak of 1,2,3,4-tetrafluoro-3-nitrobenzene

(**5h'**), "*"is the peak of 2,3,4,5,6-pentafluoronitrobenzene)

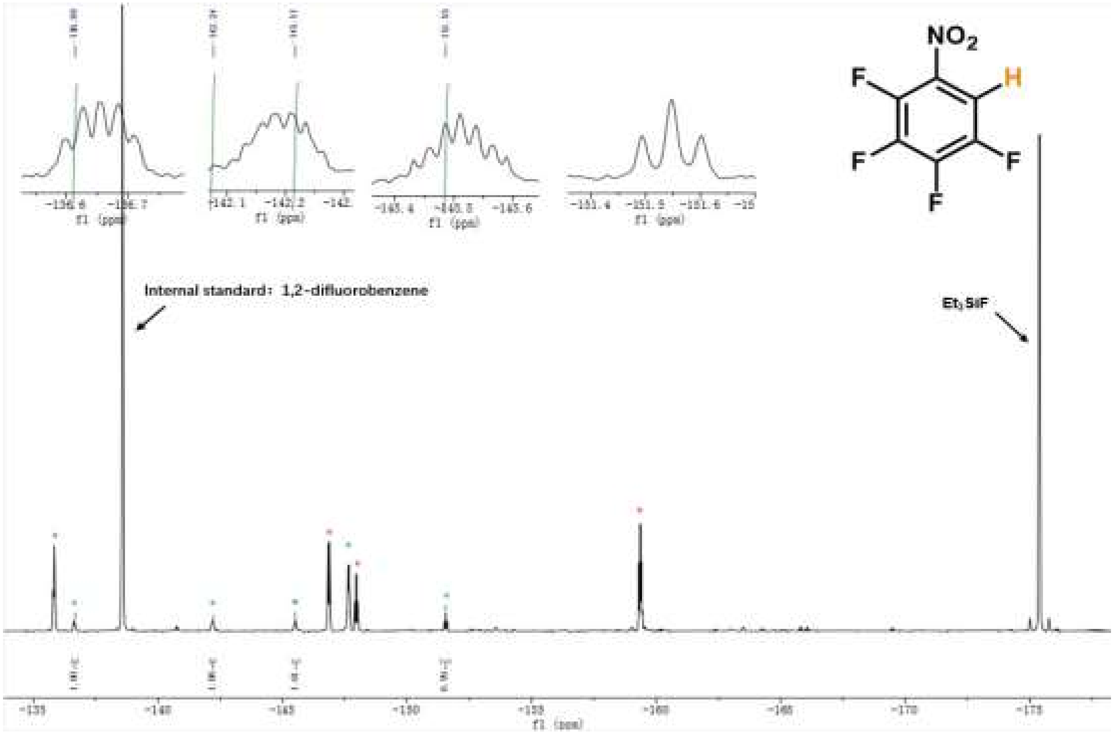

- HDF of 3,4,5,6-tetrafluorophthalonitrile

^{19}F NMR (PhF with a sealed glass capillary containing C_6D_6, 376.5 MHz, "*"is the peak of 3,4,6-trifluorophthalonitrile (**5i**), "*"is the peak of 3,6-difluorophthalonitrile (**5i'**))

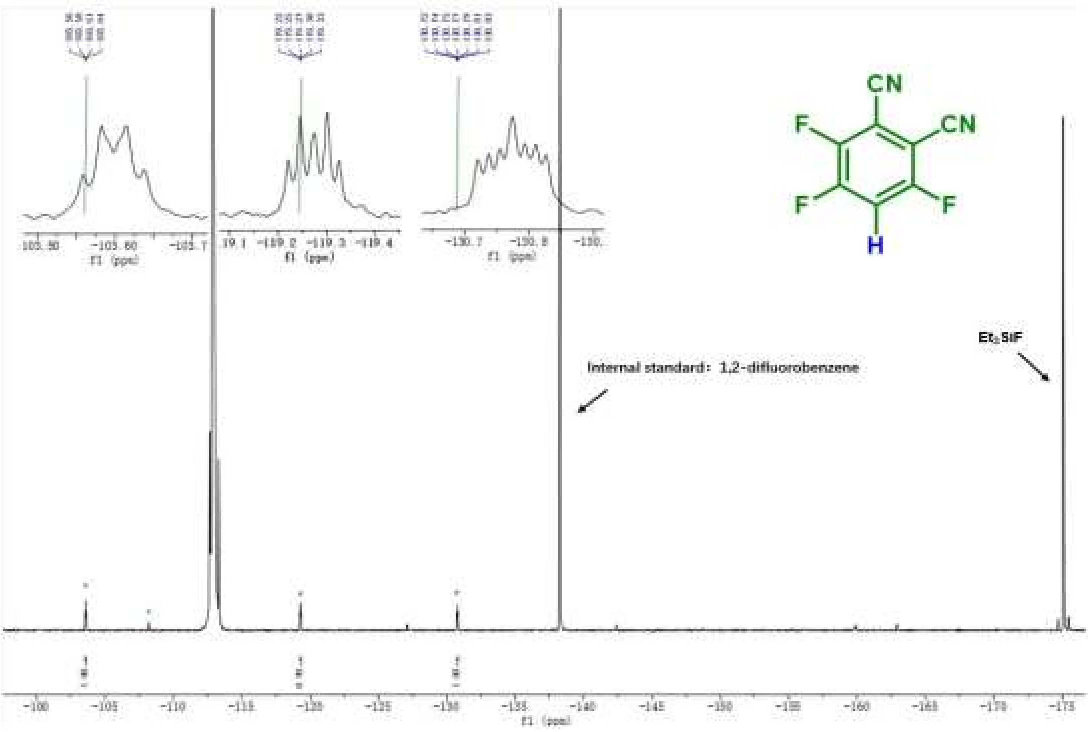

- HDF of 3,4,5,6-tetrafluorophthalonitrile

^{19}F NMR (PhF with a sealed glass capillary containing C_6D_6, 376.5 MHz, "*"is the peak of 3,4,6-trifluorophthalonitrile (**5i**), "*"is the peak of 3,6-difluorophthalonitrile (**5i'**))

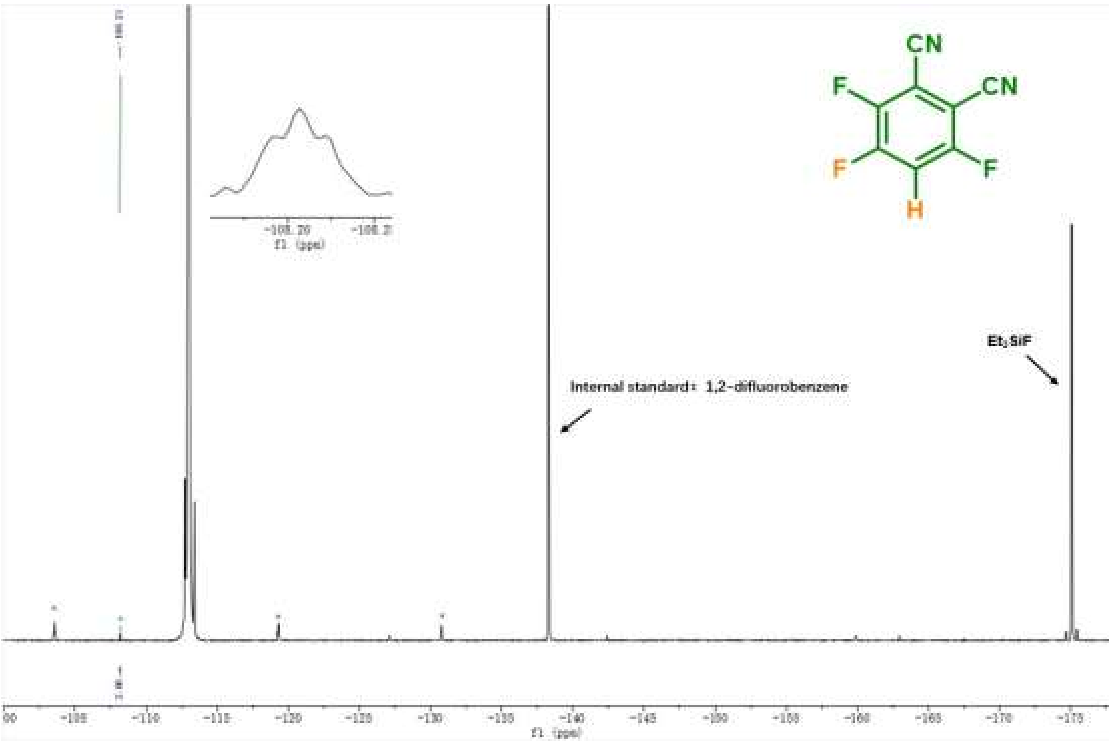

- HDF of HDF of 2,3,4,5,6-pentafluoro-1,1'-biphenyl

^{19}F NMR ($PhCF_3$ with a sealed glass capillary containing C_6D_6, 376.5 MHz, "*"is the peak of 2,3,5,6-tetrafluoro-1,1'-biphenyl (**5j**), "*"is the peak of 2,3,4,5,6-pentafluoro-1,1'-biphenyl)

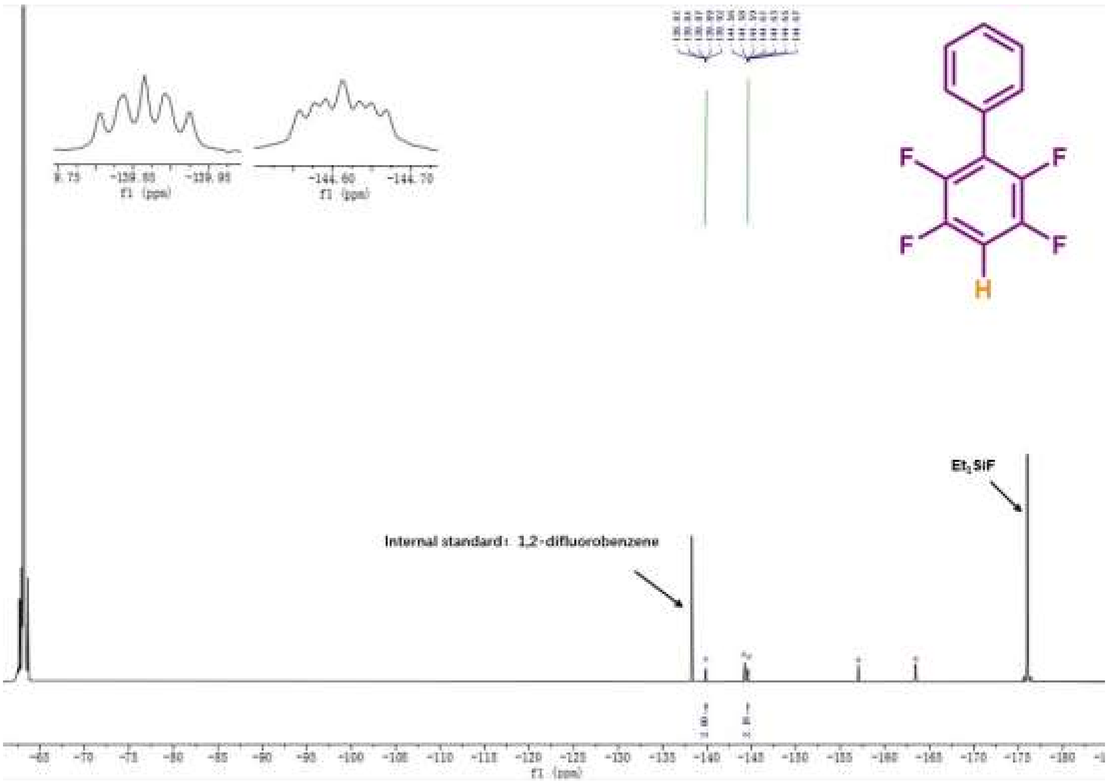

- HDF of hexafluorobenzezne

^{19}F NMR ($PhCF_3$ with a sealed glass capillary containing C_6D_6, 376.5 MHz, "*"is the peak of pentafluorobenzene (**5k**), "*"is the peak of 1,2,4,5-tetrafluorobenzene (**5k'**), "*"is the peak of

hexafluorobenzezne)

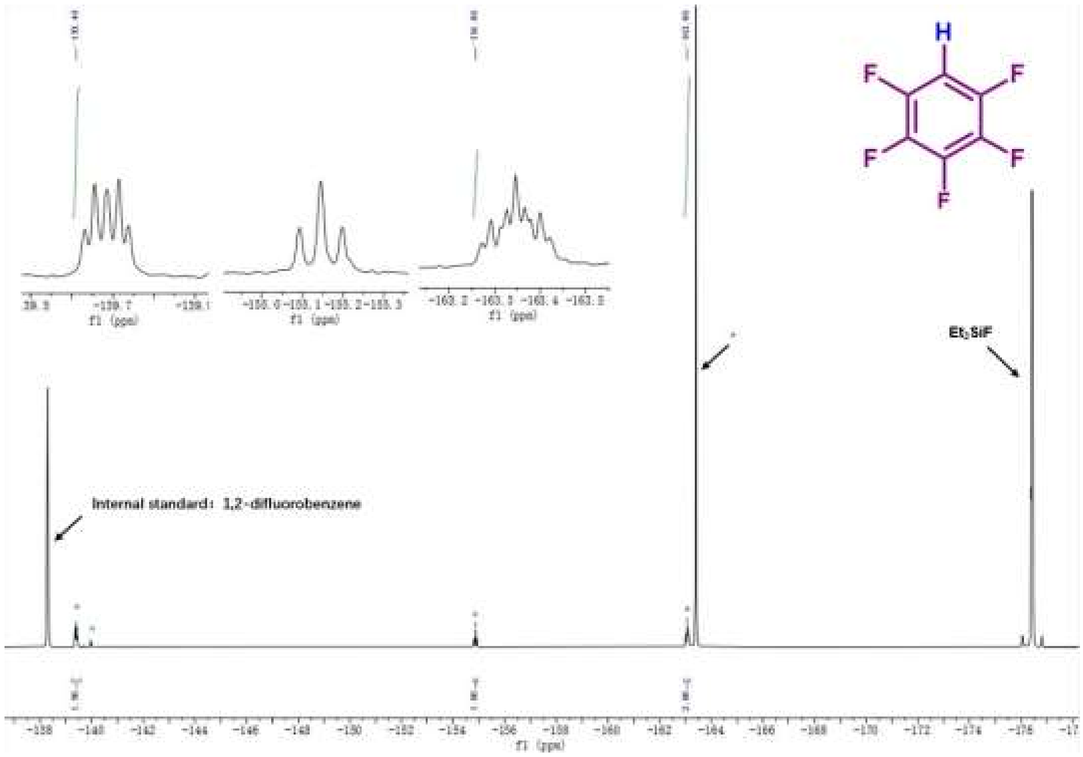

- HDF of hexafluorobenzezne ＆ HDF of pentafluorobenzene

^{19}F NMR ($PhCF_3$ with a sealed glass capillary containing C_6D_6, 376.5 MHz, “*”is the peak of pentafluorobenzene (**5k**), “*”is the peak of 1,2,4,5-tetrafluorobenzene (**5k’**))

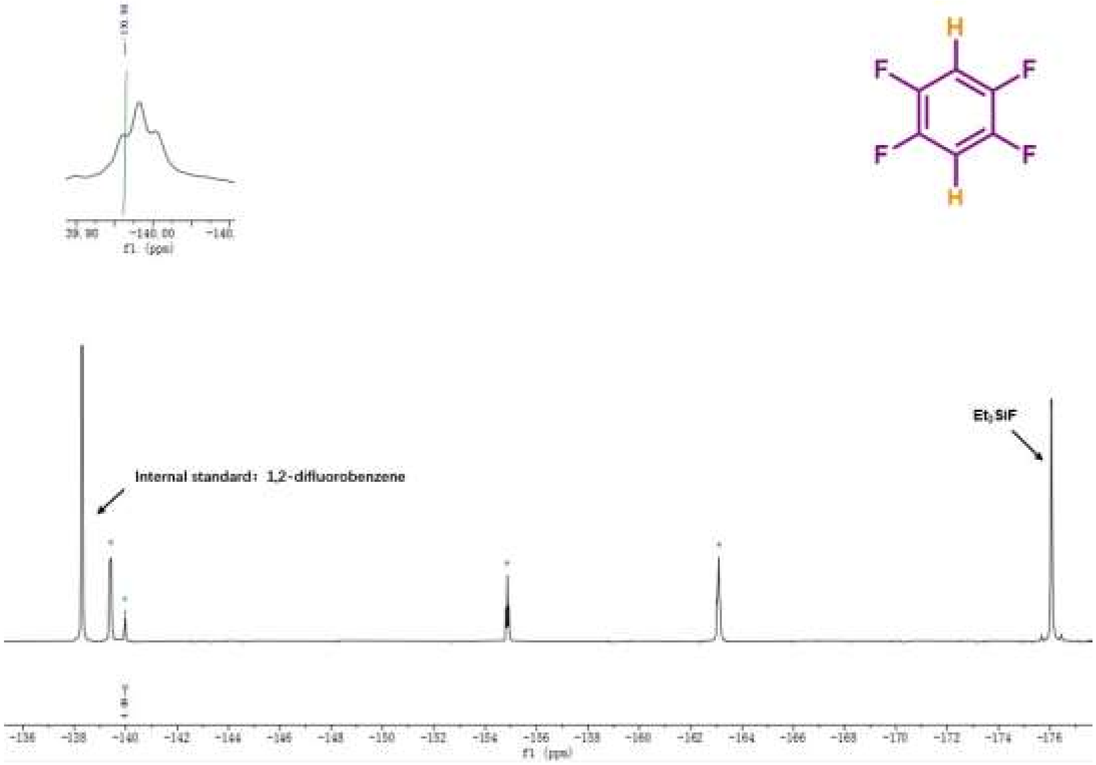

- HDF of 2,3,5,6-tetrafluoropyridine

^{19}F NMR ($PhCF_3$ with a sealed glass capillary containing C_6D_6, 376.5 MHz, “*”is the peak of 2,3,5-trifluoropyridine (**5l**))

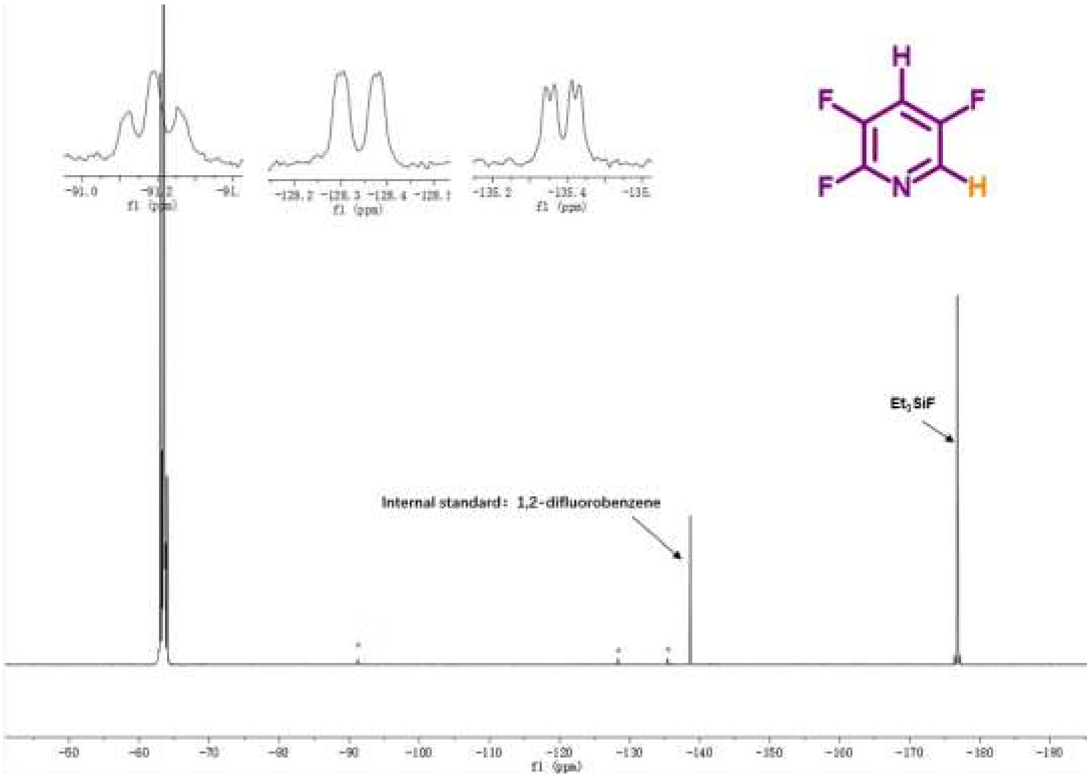

● HDF of decafluorobiphenyl

^{19}F NMR ($PhCF_3$ with a sealed glass capillary containing C_6D_6, 376.5 MHz, "*"is the peak of 2,2',3,3',4,5,5',6,6'-nonafluorobiphenyl (**5m**), "*"is the peak of 2,2',3,3',5,5',6,6'-Octafluoro-1,1'-biphenyl (**5m'**), "*"is the peak of decafluorobiphenyl)

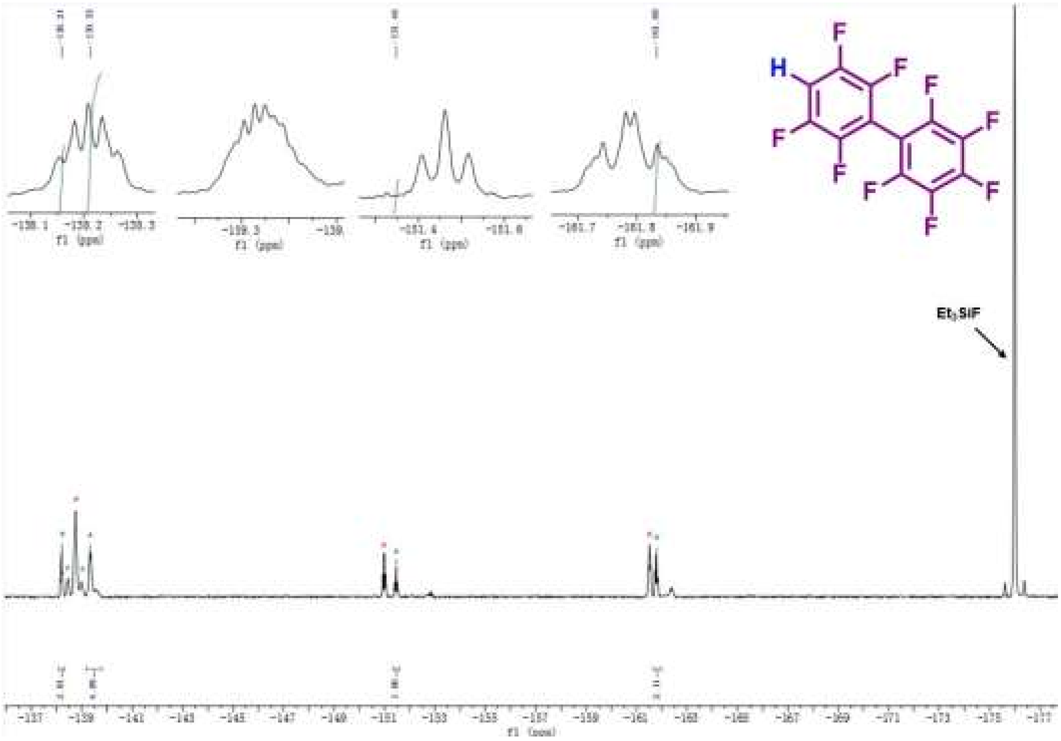

● HDF of decafluorobiphenyl

^{19}F NMR ($PhCF_3$ with a sealed glass capillary containing C_6D_6, 376.5 MHz, "*"is the peak of 2,2',3,3',4,5,5',6,6'-nonafluorobiphenyl (**5m**), "*"is the peak of 2,2',3,3',5,5',6,6'-Octafluoro-

1,1'-biphenyl (**5m'**), "*"is the peak of decafluorobiphenyl)

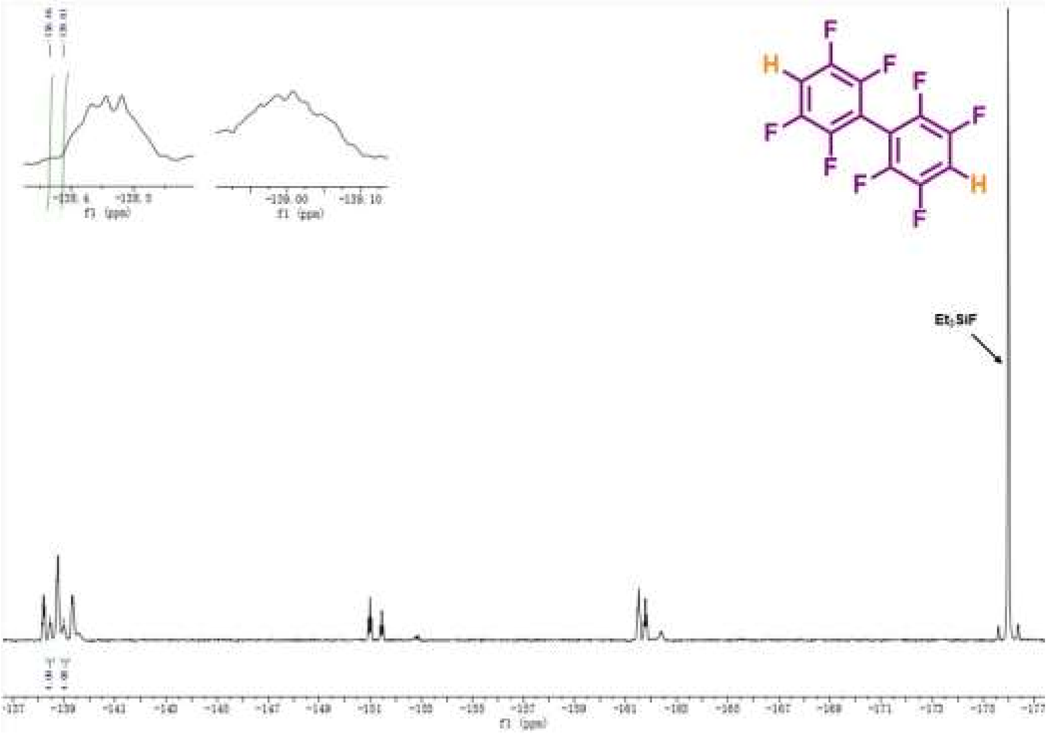

● HDF of octafluoronaphthalene

^{19}F NMR ($PhCF_3$ with a sealed glass capillary containing C_6D_6, 376.5 MHz, "*"is the peak of 2H-heptafluoro-naphthalene (**5n**), "*"is the peak of 1,2,3,4,5,8-hexafluoronaphthalene (**5n'**), "*"is the peak of 1,2,4,5,6,8-hexafluoronaphthalene (**5n''**), "*"is the peak of octafluoronaphthalene)

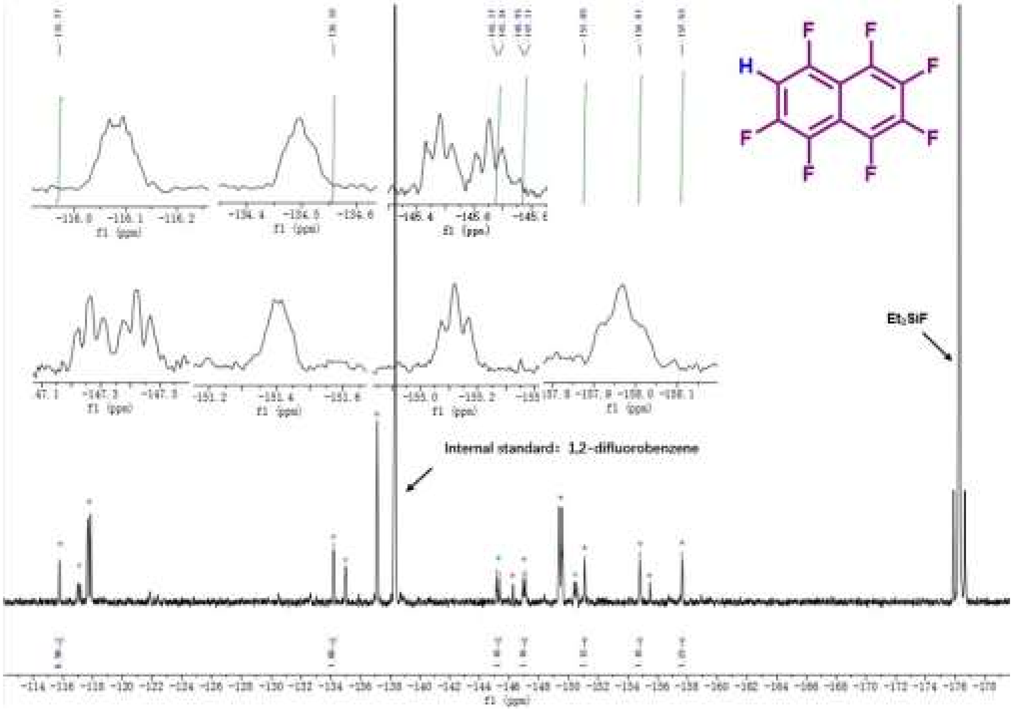

● HDF of octafluoronaphthalene

^{19}F NMR ($PhCF_3$ with a sealed glass capillary containing C_6D_6, 376.5 MHz, "*"is the peak of 2H-

heptafluoro-naphthalene (**5n**), “*”is the peak of 1,2,3,4,5,8-hexafluoronaphthalene (**5n’**), “*”is the peak of 1,2,4,5,6,8-hexafluoronaphthalene (**5n’’**), “*”is the peak of octafluoronaphthalene)

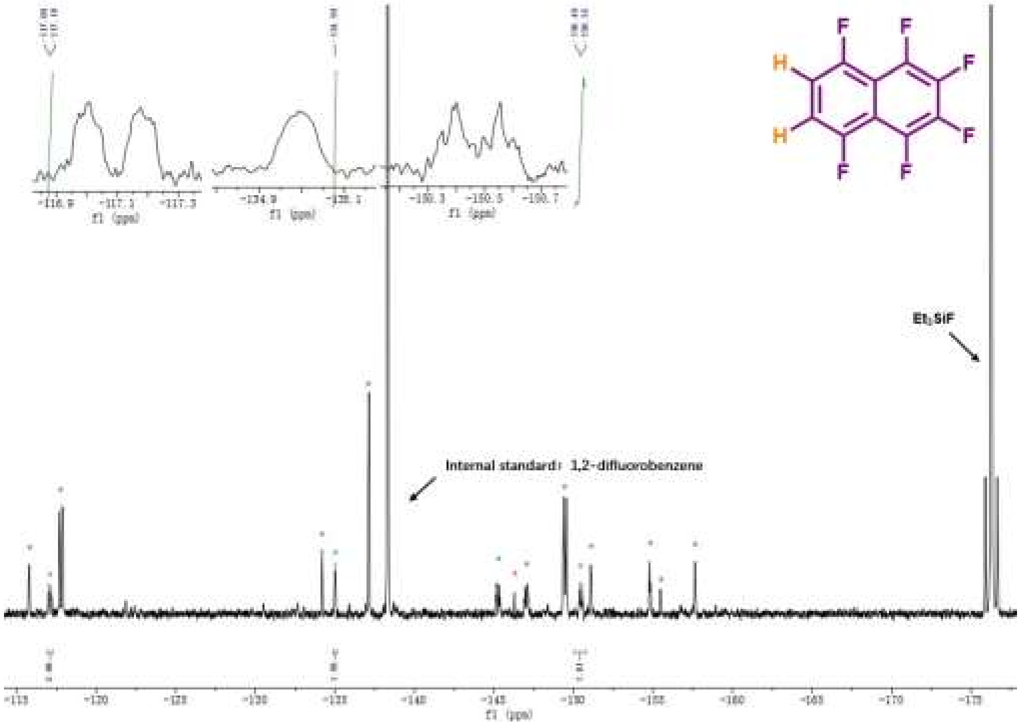

- HDF of octafluoronaphthalene

^{19}F NMR ($PhCF_3$ with a sealed glass capillary containing C_6D_6, 376.5 MHz, “*”is the peak of 2H-heptafluoro-naphthalene (**5n**), “*”is the peak of 1,2,3,4,5,8-hexafluoronaphthalene (**5n’**), “*”is the peak of 1,2,4,5,6,8-hexafluoronaphthalene (**5n’’**), “*”is the peak of octafluoronaphthalene)

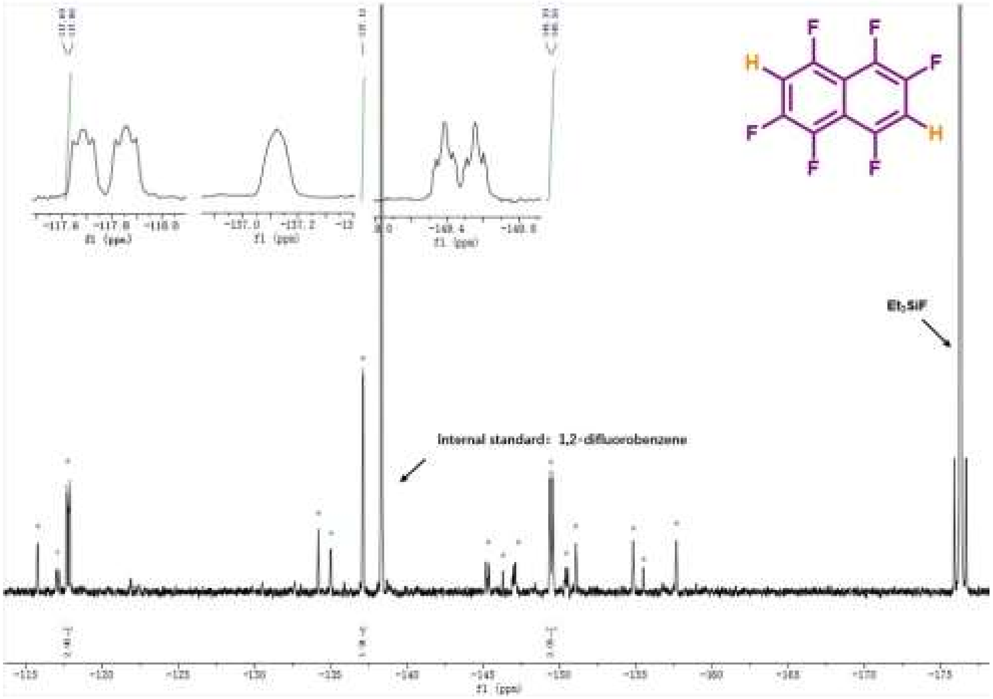

- HDF of HDF of perfluoroprop-1-ene

^{19}F NMR ($PhCF_3$ with a sealed glass capillary containing C_6D_6, 376.5 MHz, "*"is the peak of (Z)-1,2,3,3,3-pentafluoroprop-1-ene (**5o**), "*"is the peak of (E)-1,2,3,3,3-pentafluoroprop-1-ene (**5o'**), "*"is the peak of perfluoroprop-1-ene)

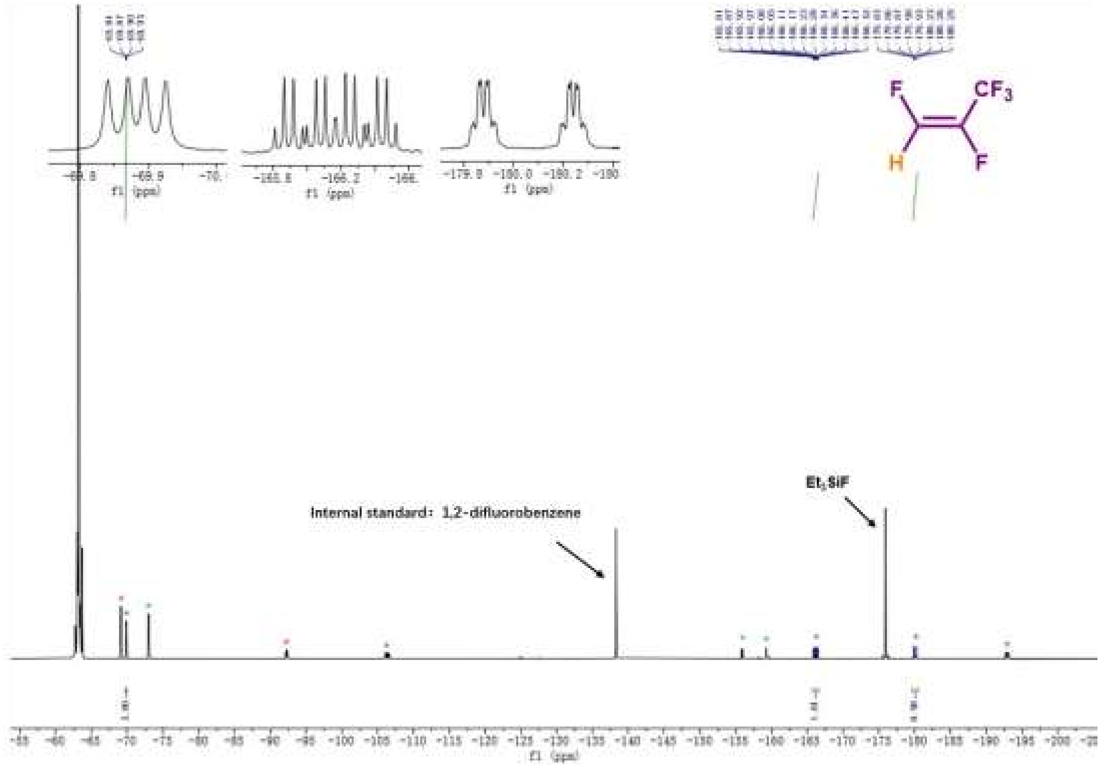

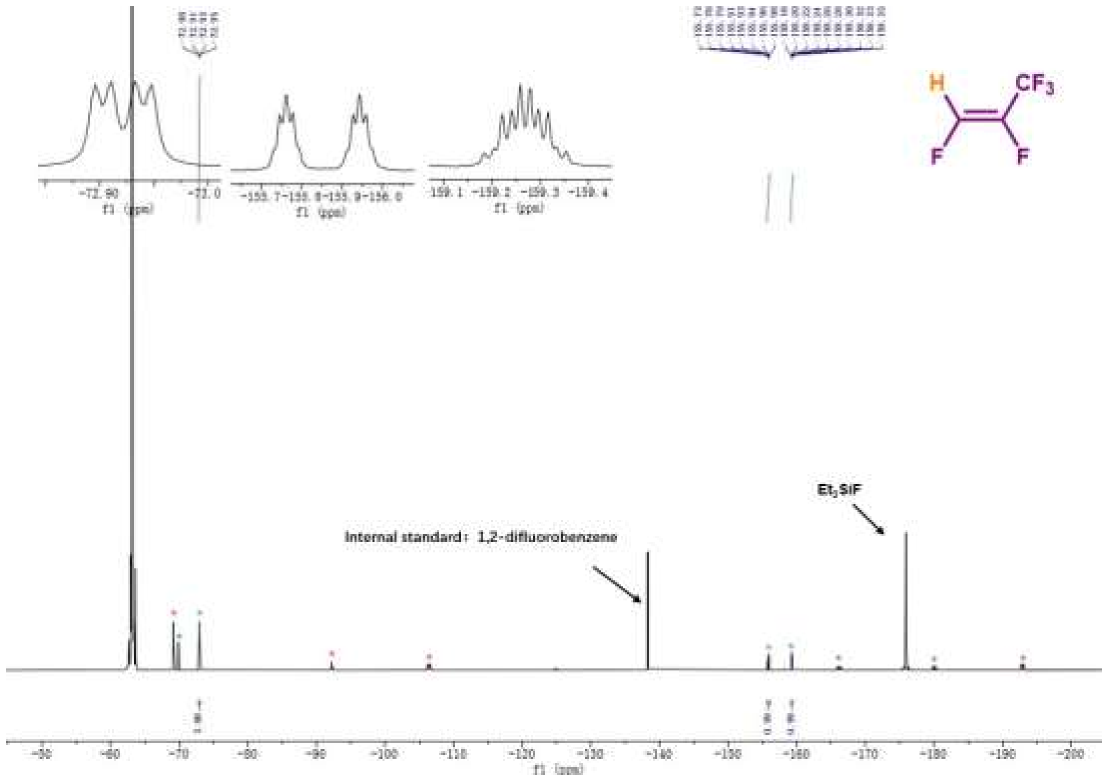

- HDF of perfluorocyclopent-1-ene

^{19}F NMR ($PhCF_3$ with a sealed glass capillary containing C_6D_6, 376.5 MHz, "*"is the peak of 1,3,3,4,4,5,5-heptafluorocyclopent-1-ene (**5p**), "*"is the peak of 3,3,4,4,5,5-hexafluorocyclopent-1-ene (**5p'**))

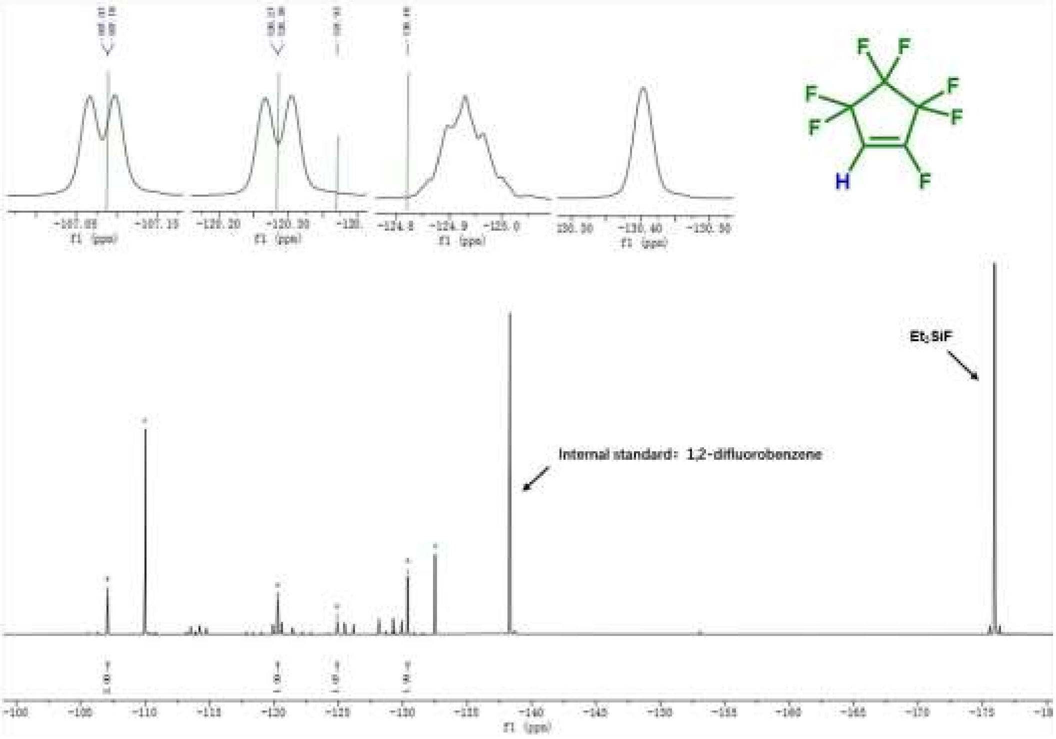

- HDF of perfluorocyclopent-1-ene

^{19}F NMR ($PhCF_3$ with a sealed glass capillary containing C_6D_6, 376.5 MHz, "*"is the peak of 1,3,3,4,4,5,5-heptafluorocyclopent-1-ene (**5p**), "*"is the peak of 3,3,4,4,5,5-hexafluorocyclopent-1-ene (**5p'**))

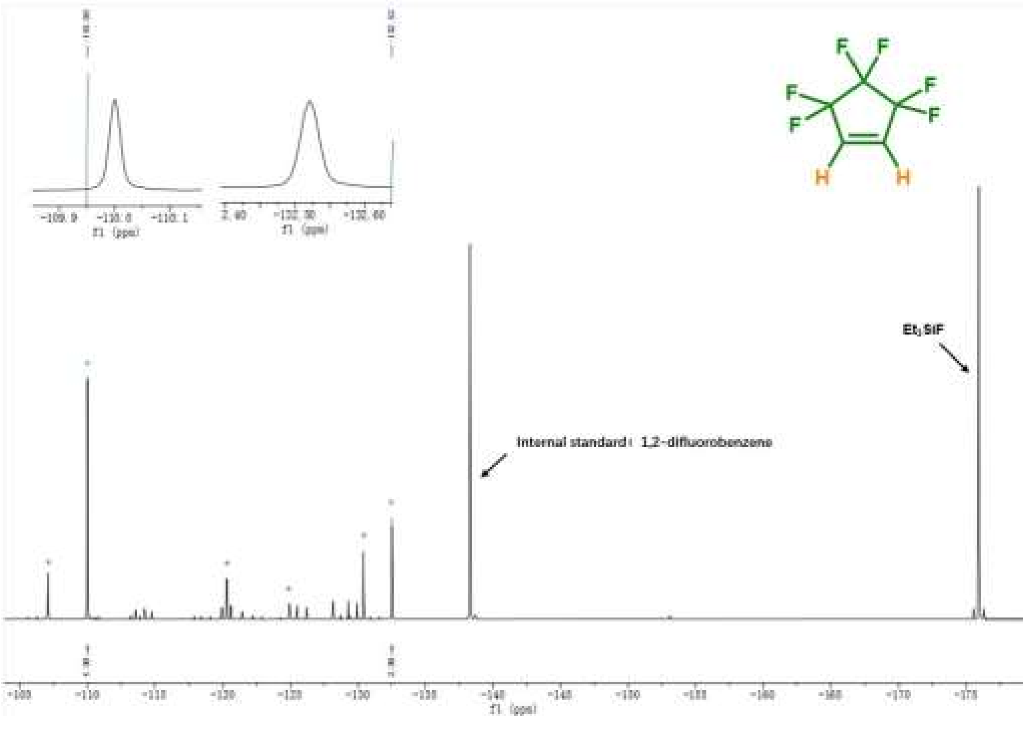

8) Selected IR spectra

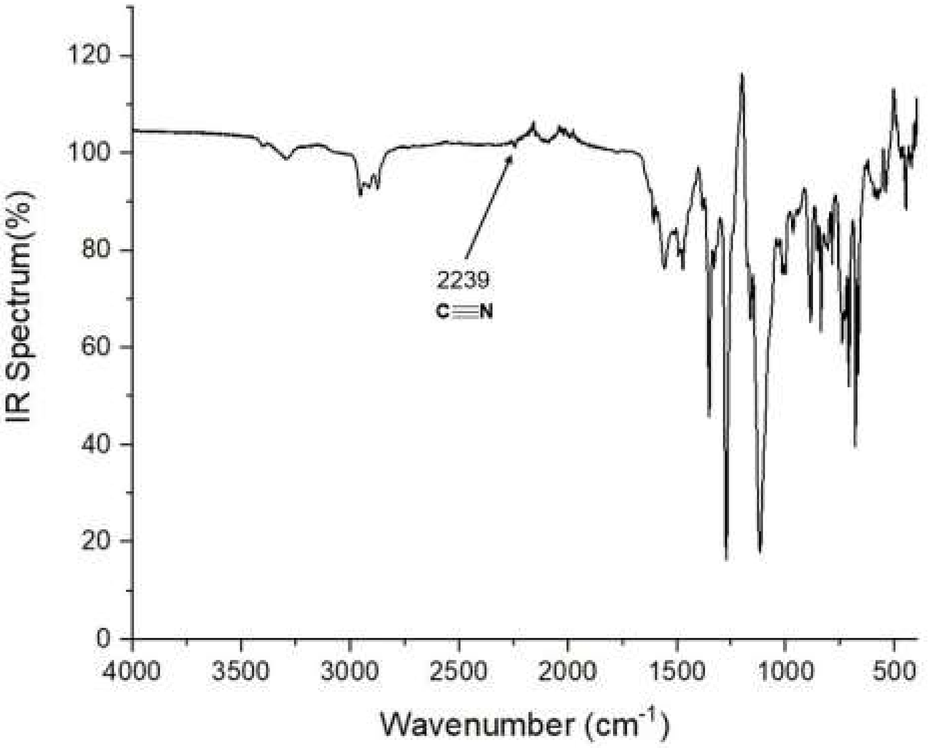

Figure S53. ATR IR spectra of **2f** (298 K)

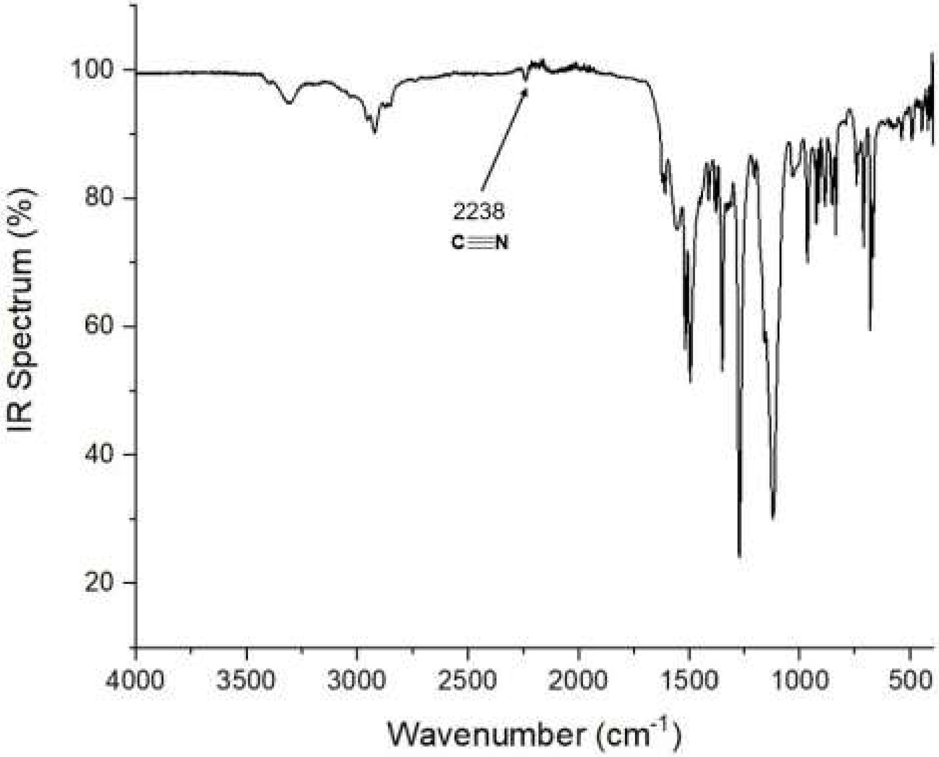

Figure S54. ATR IR spectra of **2g** and **2g'** (298 K)

9) XYZ Coordinate

AlF2-NaBArF24.log

SCF (wB97x) = -4853.12337748

E(SCF)+ZPE(0 K)= -4852.235267

H(298 K)= -4852.151384

G(298 K)= -4852.371291

Lowest Frequency = 1.9351cm-1

Na	-1.164620	-0.185709	1.196952
F	-1.402070	2.824926	2.792420
F	0.586353	3.399081	3.386849
F	-0.669112	4.801780	2.332377
F	-0.950434	3.108570	-3.237524
F	-0.329410	5.026669	-2.468912
F	1.097156	3.739875	-3.449563
C	1.495305	1.268636	-0.102825
C	1.013085	1.841765	1.082055
H	1.263464	1.387036	2.037782
C	0.224394	2.995016	1.084578
C	-0.106071	3.635372	-0.102841
H	-0.722903	4.524795	-0.104981
C	0.387125	3.100521	-1.288209
C	1.161881	1.943921	-1.287273
H	1.525749	1.560383	-2.236052
C	-0.309254	3.514441	2.393681
C	0.052973	3.748181	-2.606203
B	2.470633	-0.059702	-0.116937
F	4.948866	-2.103270	-4.785026
F	5.268153	-3.370353	-3.072274
F	4.014590	-4.044136	-4.694907
F	-1.314183	-1.011781	-3.724280
F	-0.658893	-2.711721	-4.880785
F	-1.277168	-2.972556	-2.830814
C	2.272797	-0.909980	-1.508617
C	3.335412	-1.549881	-2.159195
H	4.345732	-1.426244	-1.782265
C	3.140890	-2.354210	-3.281069
C	1.870716	-2.556270	-3.809474
H	1.718386	-3.186445	-4.677281
C	0.803214	-1.921952	-3.186985
C	1.004153	-1.112875	-2.070114
H	0.136943	-0.627518	-1.631718
C	4.337224	-2.971979	-3.953977
C	-0.604422	-2.151089	-3.663575
F	5.881962	4.362948	0.185698
F	5.562085	3.833763	-1.877217
F	7.511257	3.575546	-0.987262
F	7.047860	-1.514122	2.304768
F	8.606993	-0.605639	1.121217
F	7.399276	-2.188912	0.290128
C	4.029355	0.422786	0.022512
C	4.467099	1.687365	-0.388353
H	3.746299	2.406998	-0.765791
C	5.810323	2.057064	-0.334705
C	6.777857	1.178832	0.140711
H	7.820892	1.466742	0.185785
C	6.363342	-0.081307	0.554009
C	5.019751	-0.448279	0.495813
H	4.737789	-1.444947	0.827348
C	6.196105	3.448985	-0.756232
C	7.357629	-1.087734	1.065305
F	-0.169142	-4.646950	1.028530
F	-1.588553	-3.095483	1.503763
F	-0.829622	-4.381920	3.064684
F	3.884889	-0.880377	4.945829
F	2.249750	-1.932958	5.881805
F	2.027789	0.141590	5.332820
C	2.044363	-1.007745	1.160149
C	1.112213	-2.047203	1.053477
H	0.734329	-2.331152	0.074390
C	0.624754	-2.729000	2.171379
C	1.079361	-2.430562	3.449140
H	0.702485	-2.959275	4.316109
C	2.040667	-1.431614	3.573588
C	2.503470	-0.735352	2.459771
H	3.244688	0.046030	2.606835
C	-0.478749	-3.727822	1.952815
C	2.552743	-1.035545	4.934159
Al	-4.157222	0.043778	0.324541
F	-3.391101	0.022254	1.883469
F	-2.678871	-0.068163	-0.572759
N	-5.387567	-1.305858	0.044053
C	-6.680408	-1.059323	-0.199979
C	-7.207673	0.235533	-0.302573
H	-8.271352	0.303720	-0.485509
C	-6.511596	1.449977	-0.250306

N -5.192894 1.532971 -0.023513
C -7.612018 -2.224233 -0.389593
H -7.299510 -2.821730 -1.251065
H -8.634817 -1.885518 -0.548192
H -7.585684 -2.885717 0.480505
C -7.283987 2.720396 -0.476720
H -7.149250 3.407532 0.363155
H -8.346065 2.514438 -0.601273
H -6.917311 3.235196 -1.369553
C -4.899451 -2.661633 0.077761
C -4.413588 -3.232505 -1.106032
C -3.901809 -4.528317 -1.047467
H -3.520234 -4.982016 -1.958730
C -3.866675 -5.250942 0.145549
C -4.366406 -4.651442 1.301480
H -4.346071 -5.202469 2.238708
C -4.877586 -3.354546 1.294842
C -4.450218 -2.466866 -2.403667
H -3.813541 -1.577888 -2.355548
H -5.463461 -2.130056 -2.646769
H -4.092015 -3.086754 -3.227170
C -3.270211 -6.634187 0.192651
H -3.370472 -7.144761 -0.768580
H -3.748296 -7.247952 0.960646
H -2.202200 -6.581796 0.429165
C -5.383605 -2.713093 2.561361
H -4.796703 -1.823807 2.812807
H -5.319432 -3.410670 3.398930
H -6.426867 -2.393351 2.468177
C -4.535912 2.814013 -0.077499
C -4.373686 3.556246 1.100833
C -3.729692 4.789154 1.015110
H -3.595806 5.375166 1.920758
C -3.256464 5.289613 -0.199106
C -3.419474 4.514611 -1.346786
H -3.046184 4.884012 -2.297765
C -4.046539 3.268679 -1.308520
C -4.888728 3.039638 2.419202
H -5.965563 2.843547 2.381523
H -4.704502 3.764273 3.214679
H -4.398965 2.100679 2.694510
C -2.587461 6.639586 -0.258635
H -1.829399 6.739238 0.524520
H -3.316891 7.441738 -0.105765
H -2.105536 6.803602 -1.224975
C -4.193365 2.434975 -2.555253
H -3.798240 2.966058 -3.422664
H -5.239816 2.183959 -2.757488
H -3.645654 1.491300 -2.461302

AlHF-NaBArF24-1.log

SCF (wB97x) = -4753.78065594
E(SCF)+ZPE(0 K)= -4752.888619
H(298 K)= -4752.805391
G(298 K)= -4753.023517
Lowest Frequency = 6.5484cm-1

Na -1.098252 0.179459 1.146769
F -1.163292 3.339602 2.480021
F 0.842022 3.884606 3.046518
F -0.336338 5.225241 1.834250
F -0.484749 2.949535 -3.609258
F 0.053471 4.932793 -2.952331
F 1.566176 3.597361 -3.715969
C 1.592703 1.304239 -0.187280
C 1.156824 2.041498 0.922393
H 1.349717 1.669527 1.926048
C 0.484811 3.259216 0.792676
C 0.242584 3.811634 -0.458074
H -0.275285 4.756374 -0.560957
C 0.698580 3.115516 -1.571795
C 1.349771 1.891883 -1.438964
H 1.687497 1.383834 -2.337608
C -0.034277 3.937801 2.032278
C 0.460824 3.654878 -2.957655
B 2.432096 -0.109336 -0.048857
F 5.028540 -2.576936 -4.312271
F 4.964877 -3.955230 -2.658617
F 3.839230 -4.370599 -4.452581
F -1.410387 -1.096718 -3.437068
F -0.674837 -2.492114 -4.910324
F -1.306436 -3.200362 -2.975240
C 2.222336 -1.034311 -1.388979
C 3.252765 -1.790482 -1.956568
H 4.255646 -1.722192 -1.546043
C 3.036103 -2.641564 -3.041491
C 1.776090 -2.774337 -3.608962

H	1.606824	-3.436863	-4.449433
C	0.737184	-2.024488	-3.065931
C	0.960293	-1.173474	-1.987848
H	0.122368	-0.593673	-1.612914
C	4.209280	-3.389849	-3.615694
C	-0.657899	-2.195488	-3.601020
F	6.228450	4.001459	0.141972
F	5.942997	3.363147	-1.895000
F	7.821599	2.988819	-0.900479
F	6.816242	-1.641104	2.785840
F	8.441516	-1.154338	1.452754
F	7.006497	-2.669675	0.903361
C	4.021136	0.241819	0.140947
C	4.592836	1.428097	-0.333810
H	3.961224	2.177376	-0.802265
C	5.959819	1.681788	-0.230163
C	6.817792	0.760401	0.359994
H	7.879243	0.958534	0.443975
C	6.269876	-0.425031	0.834541
C	4.902705	-0.675404	0.728290
H	4.514761	-1.615956	1.112208
C	6.492193	2.999885	-0.723384
C	7.138180	-1.464695	1.488986
F	-0.172643	-4.731129	1.509723
F	-1.656594	-3.178059	1.346939
F	-1.209507	-4.045925	3.272799
F	3.587866	-0.674210	5.115450
F	1.851052	-1.555963	6.044675
F	1.782737	0.481207	5.337048
C	1.878651	-0.937501	1.265724
C	0.931462	-1.966778	1.184069
H	0.588110	-2.302537	0.209125
C	0.396243	-2.580568	2.320928
C	0.813323	-2.217610	3.594652
H	0.406283	-2.698041	4.475855
C	1.775856	-1.217248	3.698617
C	2.287808	-0.591616	2.564961
H	3.041438	0.181115	2.694927
C	-0.657975	-3.637904	2.122054
C	2.250409	-0.751868	5.050727
Al	-4.368752	0.171809	0.622463
H	-3.886381	0.340694	2.133841
F	-2.836278	-0.087551	-0.193881
N	-5.620840	-1.171929	0.237158
C	-6.841425	-0.927841	-0.248006
C	-7.287843	0.355472	-0.593943
H	-8.304473	0.427660	-0.956136
C	-6.540719	1.541691	-0.600764
N	-5.282490	1.617525	-0.156330
C	-7.782207	-2.085196	-0.452865
H	-7.383699	-2.771234	-1.206052
H	-8.762614	-1.739840	-0.778609
H	-7.893880	-2.659408	0.470874
C	-7.189012	2.782952	-1.154008
H	-7.137534	3.601534	-0.431032
H	-8.231786	2.598978	-1.409532
H	-6.662113	3.119044	-2.052021
C	-5.180847	-2.525508	0.445715
C	-4.583961	-3.212077	-0.622080
C	-4.118745	-4.505624	-0.394713
H	-3.655138	-5.048371	-1.215070
C	-4.220751	-5.113937	0.857198
C	-4.820665	-4.402286	1.895045
H	-4.913970	-4.865417	2.874532
C	-5.305057	-3.106178	1.713823
C	-4.447390	-2.561457	-1.974653
H	-3.809962	-1.673670	-1.917476
H	-5.415256	-2.238681	-2.372287
H	-3.999769	-3.250938	-2.692059
C	-3.658602	-6.493016	1.088552
H	-3.774472	-7.124187	0.203333
H	-4.148248	-6.988157	1.931061
H	-2.588027	-6.437148	1.313315
C	-5.954505	-2.354536	2.847764
H	-5.402140	-1.439963	3.088675
H	-5.995972	-2.971371	3.747912
H	-6.976594	-2.051424	2.597811
C	-4.543345	2.842640	-0.299932
C	-4.518778	3.762741	0.758523
C	-3.782921	4.935767	0.596730
H	-3.759757	5.658041	1.409225
C	-3.087867	5.210704	-0.582013
C	-3.115741	4.263526	-1.605213
H	-2.575935	4.459242	-2.527970
C	-3.828776	3.069783	-1.484365
C	-5.284414	3.498079	2.029491
H	-6.354969	3.374198	1.834972
H	-5.162107	4.323566	2.733607

H -4.941560 2.580409 2.518630
C -2.347930 6.514158 -0.746906
H -1.700892 6.498585 -1.627446
H -1.733413 6.735865 0.130539
H -3.050771 7.345037 -0.867965
C -3.836358 2.055984 -2.599711
H -3.240667 2.406809 -3.444226
H -4.850901 1.856768 -2.960677
H -3.422134 1.100987 -2.261605

BDIAlF2.log

SCF (wB97x) = -1205.15905663
E(SCF)+ZPE(0 K)= -1204.682895
H(298 K)= -1204.651339
G(298 K)= -1204.746033
Lowest Frequency = 8.6035cm-1

C 1.257997 1.424859 1.295868
C 0.000997 1.911988 1.679682
C -1.256713 1.426360 1.296313
N -1.423862 0.381128 0.481179
N 1.423860 0.379394 0.480760
C 2.478946 2.123795 1.832692
C -2.476643 2.126619 1.833779
C 2.752267 -0.050281 0.129837
C -2.752539 -0.047456 0.130167
C 3.361125 0.481904 -1.014952
C 4.630473 0.019602 -1.360726
C 5.292263 -0.947521 -0.604133
C 4.652613 -1.459555 0.524627
C 3.383520 -1.028412 0.910062
C -3.362324 0.487951 -1.012490
C -4.635311 0.032315 -1.354012
C -5.297636 -0.934601 -0.597861
C -4.659196 -1.446168 0.531925
C -3.386664 -1.021724 0.913062
C 6.676252 -1.409973 -0.983386
C 2.707199 -1.599178 2.130084
C 2.660381 1.523635 -1.848910
C -2.712160 -1.590490 2.135022
C -6.658351 -1.437186 -1.008678
C -2.661623 1.530840 -1.845029
H 0.001633 2.764517 2.345439
H 3.090910 2.509588 1.012457
H 2.201187 2.950976 2.484626
H 3.105739 1.425093 2.393789
H -3.103686 1.428742 2.395622
H -2.197601 2.953718 2.485259
H -3.088982 2.512751 1.014010
H 5.111798 0.423243 -2.248417
H 5.151474 -2.219915 1.120893
H -5.120258 0.443190 -2.236372
H -5.163115 -2.198729 1.133862
H 6.829496 -1.360075 -2.064608
H 7.439079 -0.779337 -0.513260
H 6.854419 -2.438574 -0.659039
H 3.351447 -2.331504 2.621193
H 2.466862 -0.818652 2.860158
H 1.767239 -2.091500 1.862583
H 2.425805 2.418668 -1.262429
H 3.285272 1.828149 -2.691097
H 1.714716 1.138576 -2.242432
H -3.358080 -2.320226 2.627782
H -1.773333 -2.085631 1.868726
H -2.469939 -0.808868 2.863303
H -7.220457 -0.669478 -1.547005
H -6.566439 -2.304096 -1.672138
H -7.246276 -1.748596 -0.141139
H -1.716569 1.145468 -2.239721
H -3.286751 1.836920 -2.686465
H -2.425779 2.424932 -1.257625
Al -0.000784 -0.583022 -0.262208
F -0.001198 -0.541770 -1.978976
F -0.002182 -2.234236 0.204947

BDIAlH2.log

SCF (wB97x) = -1006.48061656
E(SCF)+ZPE(0 K)= -1005.996568
H(298 K)= -1005.966685
G(298 K)= -1006.058157
Lowest Frequency = 6.8622cm-1

C 1.258226 1.497681 1.221387
C 0.001435 1.997049 1.596681
C -1.256384 1.499114 1.222976
N -1.423716 0.488157 0.374964

N	1.423621	0.486353	0.373406
C	2.477763	2.139418	1.835737
C	-2.474449	2.141865	1.839199
C	2.740932	-0.005398	0.092246
C	-2.741464	-0.002558	0.094436
C	3.455451	0.511095	-0.998367
C	4.711136	-0.026040	-1.282342
C	5.262732	-1.053039	-0.516733
C	4.523810	-1.548007	0.557812
C	3.264702	-1.042039	0.879093
C	-3.457769	0.517277	-0.993335
C	-4.717112	-0.013989	-1.272342
C	-5.268440	-1.041526	-0.507371
C	-4.530017	-1.536137	0.567754
C	-3.267387	-1.036058	0.884059
C	6.634446	-1.597124	-0.827139
C	2.483780	-1.587499	2.046798
C	2.883978	1.628440	-1.832876
C	-2.487680	-1.579603	2.053473
C	-6.615855	-1.624236	-0.851908
C	-2.887200	1.636444	-1.826039
H	0.002355	2.830833	2.286266
H	3.159672	2.498862	1.060306
H	2.200092	2.975611	2.476766
H	3.033021	1.409677	2.432491
H	-3.029178	1.412671	2.437110
H	-2.195074	2.977965	2.479602
H	-3.157559	2.501645	1.065016
H	5.271077	0.367741	-2.127616
H	4.935650	-2.353705	1.161384
H	-5.281272	0.386845	-2.111466
H	-4.946395	-2.334929	1.177376
H	6.860690	-1.520327	-1.893997
H	7.406741	-1.038847	-0.286191
H	6.721840	-2.646562	-0.533400
H	3.019897	-2.412116	2.521718
H	2.307507	-0.818123	2.806502
H	1.502000	-1.951360	1.727458
H	2.742630	2.538755	-1.239868
H	3.548628	1.870274	-2.665178
H	1.904586	1.358250	-2.239109
H	-3.024759	-2.402742	2.529857
H	-1.506274	-1.945210	1.734968
H	-2.310567	-0.809002	2.811758
H	-7.258236	-0.883456	-1.335595
H	-6.509101	-2.468036	-1.542608
H	-7.129726	-1.993518	0.039754
H	-1.908205	1.366639	-2.233494
H	-3.552436	1.879639	-2.657472
H	-2.745132	2.545860	-1.231829
H	-0.002515	-0.113282	-2.115398
H	-0.002392	-2.003414	-0.180386
Al	-0.001288	-0.436788	-0.539383

BDIAlHF.log

SCF (wB97x) = -1105.82164086

E(SCF)+ZPE(0 K)= -1105.341452

H(298 K)= -1105.310775

G(298 K)= -1105.402520

Lowest Frequency = 12.2194cm-1

C	1.256722	1.545934	1.172767
C	0.001328	2.053054	1.537821
C	-1.255000	1.547979	1.173197
N	-1.417861	0.535071	0.321301
N	1.417896	0.532952	0.320623
C	2.475920	2.161036	1.810594
C	-2.473032	2.165361	1.811079
C	2.732956	0.009467	0.073006
C	-2.733330	0.012548	0.074261
C	3.496979	0.520202	-0.985704
C	4.749833	-0.044049	-1.226204
C	5.247817	-1.091638	-0.451667
C	4.456683	-1.582435	0.586993
C	3.198733	-1.049279	0.865538
C	-3.498380	0.525116	-0.982809
C	-4.754699	-0.033562	-1.218112
C	-5.253138	-1.080439	-0.442923
C	-4.463318	-1.569433	0.597570
C	-3.201942	-1.041783	0.870988
C	6.617826	-1.663299	-0.715950
C	2.354897	-1.596114	1.988205
C	2.978374	1.648924	-1.839115
C	-2.360381	-1.584952	1.997149
C	-6.599466	-1.689424	-0.742881
C	-2.979998	1.654351	-1.835676
H	0.002131	2.886212	2.228009

H 3.183799 2.501986 1.050627
H 2.203062 3.003938 2.444619
H 2.998727 1.418110 2.420577
H -2.996942 1.423792 2.421739
H -2.198592 3.008205 2.444492
H -3.180648 2.507012 1.051202
H 5.350366 0.344069 -2.045650
H 4.825055 -2.405413 1.195078
H -5.358864 0.360236 -2.032169
H -4.836697 -2.384715 1.212932
H 6.879704 -1.594348 -1.775123
H 7.382551 -1.117604 -0.151968
H 6.675327 -2.713107 -0.416500
H 2.871993 -2.408900 2.502582
H 2.116871 -0.824468 2.728117
H 1.401522 -1.982444 1.612024
H 2.792242 2.549514 -1.243690
H 3.698831 1.905436 -2.618868
H 2.030622 1.379296 -2.314704
H -2.877966 -2.396912 2.512326
H -1.405896 -1.971407 1.623977
H -2.124381 -0.811457 2.735810
H -7.285517 -0.950104 -1.164923
H -6.503765 -2.502293 -1.471184
H -7.057137 -2.107394 0.157724
H -2.032808 1.384185 -2.312080
H -3.700808 1.911570 -2.614864
H -2.792730 2.554639 -1.240158
H -0.002263 -1.898536 -0.532204
Al -0.000928 -0.313637 -0.616846
F -0.001387 0.187109 -2.282097

BDIGaF2.log

SCF (wB97x) = -1205.15030023
E(SCF)+ZPE(0 K)= -1204.675602
H(298 K)= -1204.643531
G(298 K)= -1204.739076
Lowest Frequency = 12.6963cm-1

C 1.266917 1.402753 1.295512
C 0.001122 1.877405 1.672601
C -1.265493 1.404488 1.296091
N -1.466103 0.363478 0.485785
Ga -0.001001 -0.628524 -0.266571
N 1.466019 0.361511 0.485098
C 2.475335 2.119733 1.839771
C -2.472711 2.123099 1.840912
C 2.796418 -0.052470 0.134407
C -2.796775 -0.049272 0.134935
C 3.397107 0.486959 -1.010878
C 4.672978 0.042319 -1.354861
C 5.346348 -0.916253 -0.597312
C 4.710914 -1.440474 0.528329
C 3.435633 -1.026831 0.912055
C -3.398283 0.493497 -1.008228
C -4.677598 0.055492 -1.348128
C -5.351476 -0.903076 -0.591227
C -4.717309 -1.426997 0.535362
C -3.438725 -1.020031 0.914954
C 6.737154 -1.359308 -0.974611
C 2.756054 -1.614466 2.121788
C 2.675789 1.511480 -1.847981
C -2.760967 -1.605989 2.126522
C -6.719521 -1.386517 -1.000643
C -2.677019 1.519284 -1.843820
F -0.001991 -0.627311 -2.078088
H 0.001870 2.729810 2.338326
H 3.084877 2.514437 1.022022
H 2.182069 2.942131 2.490737
H 3.108767 1.428799 2.402943
H -3.106879 1.433149 2.404440
H -2.178000 2.945102 2.491718
H -3.082194 2.518661 1.023554
H 5.149507 0.451523 -2.242543
H 5.217303 -2.197599 1.122255
H -5.157602 0.471993 -2.230507
H -5.228600 -2.176556 1.134728
H 6.890330 -1.309630 -2.055851
H 7.490234 -0.716344 -0.505495
H 6.930154 -2.384414 -0.647768
H 3.402394 -2.346018 2.611087
H 2.502703 -0.843166 2.857217
H 1.822667 -2.112696 1.841093
H 2.427269 2.405916 -1.266330
H 3.291194 1.821578 -2.694965
H 1.735875 1.105951 -2.235661
H -3.408453 -2.336054 2.616519

H	-1.828031	-2.105796	1.847099
H	-2.506990	-0.833956	2.860971
H	-7.275911	-0.606926	-1.527726
H	-6.639952	-2.246875	-1.674149
H	-7.306475	-1.701177	-0.133677
H	-1.737515	1.113595	-2.232328
H	-3.292490	1.830705	-2.690263
H	-2.427632	2.412905	-1.261292
F	-0.002795	-2.373492	0.219168

BDIGaH2.log

SCF (wB97x) = -1006.52403637

E(SCF)+ZPE(0 K)= -1006.041280

H(298 K)= -1006.010810

G(298 K)= -1006.103288

Lowest Frequency = 9.8352cm-1

C	1.265262	1.483457	1.199392
C	0.001262	1.976954	1.564890
C	-1.263643	1.484920	1.200599
N	-1.458819	0.477276	0.360884
Ga	-0.001241	-0.507018	-0.603928
N	1.458748	0.475418	0.359710
C	2.472305	2.142468	1.825970
C	-2.469407	2.144956	1.828559
C	2.778583	-0.004054	0.093277
C	-2.779003	-0.000996	0.094484
C	3.496022	0.509487	-0.997299
C	4.756754	-0.019988	-1.272431
C	5.311639	-1.039571	-0.499121
C	4.569488	-1.535063	0.572881
C	3.305255	-1.035830	0.884910
C	-3.497886	0.516566	-0.993240
C	-4.762038	-0.006841	-1.264127
C	-5.316889	-1.027529	-0.492217
C	-4.575647	-1.523189	0.580308
C	-3.308032	-1.029959	0.888147
C	6.688849	-1.575467	-0.799830
C	2.519140	-1.583253	2.048245
C	2.917775	1.615874	-1.841603
C	-2.523688	-1.575869	2.053391
C	-6.670100	-1.602037	-0.828319
C	-2.920271	1.625451	-1.834716
H	0.002080	2.813223	2.251597
H	3.160080	2.504192	1.056709
H	2.178738	2.979812	2.458495
H	3.027550	1.422151	2.434149
H	-3.024606	1.425068	2.437283
H	-2.174383	2.981937	2.460879
H	-3.157824	2.507414	1.060238
H	5.318059	0.373163	-2.117233
H	4.982648	-2.336368	1.181564
H	-5.327266	0.393924	-2.102721
H	-4.993426	-2.317907	1.194457
H	6.916848	-1.510019	-1.867161
H	7.455508	-1.004006	-0.264592
H	6.784895	-2.620464	-0.493074
H	3.050252	-2.411987	2.521740
H	2.343461	-0.816139	2.810367
H	1.536277	-1.940681	1.725475
H	2.771787	2.530853	-1.256973
H	3.579260	1.853734	-2.677634
H	1.938696	1.336956	-2.242532
H	-3.055714	-2.403673	2.527476
H	-1.540803	-1.934367	1.731862
H	-2.348086	-0.807899	2.814694
H	-7.318394	-0.851150	-1.288176
H	-6.575553	-2.432413	-1.536949
H	-7.171784	-1.987472	0.063437
H	-1.941606	1.347063	-2.237045
H	-3.582229	1.865589	-2.669715
H	-2.773423	2.538942	-1.247965
H	-0.002265	-0.124962	-2.150626
H	-0.002388	-2.041194	-0.179935

BDIGaHF.log

SCF (wB97x) = -1105.84254755

E(SCF)+ZPE(0 K)= -1105.363702

H(298 K)= -1105.332481

G(298 K)= -1105.425062

Lowest Frequency = 21.0437cm-1

C	1.264456	1.510534	1.168571
C	0.001379	2.008221	1.525694
C	-1.262621	1.512371	1.169348
N	-1.455779	0.494831	0.333947

Ga	-0.001050	-0.429424	-0.602420
N	1.455852	0.492840	0.332926
C	2.471933	2.154862	1.804106
C	-2.468864	2.158619	1.805296
C	2.777461	-0.004113	0.084125
C	-2.777836	-0.000920	0.085527
C	3.524641	0.515372	-0.982696
C	4.787185	-0.024510	-1.227456
C	5.311292	-1.057381	-0.450178
C	4.535827	-1.559503	0.594726
C	3.268553	-1.050890	0.877255
C	-3.526031	0.521219	-0.979243
C	-4.791966	-0.012823	-1.219279
C	-5.316427	-1.045651	-0.442213
C	-4.542258	-1.546667	0.604181
C	-3.271704	-1.043788	0.882093
C	6.691374	-1.601932	-0.719550
C	2.440840	-1.611788	2.005159
C	2.974272	1.626958	-1.837993
C	-2.446273	-1.601751	2.013125
C	-6.673275	-1.628305	-0.747357
C	-2.975842	1.633911	-1.833217
F	-0.001922	0.207143	-2.342362
H	0.002209	2.847918	2.207834
H	-0.002218	-1.991904	-0.587870
H	3.173582	2.503837	1.042050
H	2.182015	2.997325	2.431029
H	3.008192	1.426575	2.420007
H	-3.005886	1.431439	2.421820
H	-2.177409	3.000923	2.431711
H	-3.170488	2.508292	1.043552
H	5.374359	0.370545	-2.053316
H	4.923985	-2.372203	1.204395
H	-5.382684	0.388704	-2.039479
H	-4.935292	-2.352064	1.220397
H	6.942385	-1.540669	-1.781869
H	7.448819	-1.032265	-0.169607
H	6.775018	-2.646337	-0.407571
H	2.980805	-2.404997	2.526470
H	2.180412	-0.840936	2.738141
H	1.498556	-2.030193	1.635317
H	2.823496	2.542718	-1.255256
H	3.659296	1.860570	-2.656015
H	2.002454	1.352171	-2.259075
H	-2.987207	-2.393717	2.535311
H	-1.503416	-2.021049	1.645802
H	-2.186854	-0.829323	2.744829
H	-7.343510	-0.875457	-1.170932
H	-6.591218	-2.442183	-1.476255
H	-7.142098	-2.038069	0.151298
H	-2.004680	1.358622	-2.255485
H	-3.661320	1.869024	-2.650423
H	-2.823642	2.548886	-1.249622

C5F4NH.log

SCF (wB97x) = -645.182891291

E(SCF)+ZPE(0 K)= -645.125813

H(298 K)= -645.117515

G(298 K)= -645.158263

Lowest Frequency = 119.2327cm-1

C	-1.952989	0.470774	0.000122
C	-1.207462	1.645438	-0.000149
C	-1.873130	2.859766	-0.001106
C	-3.257581	2.829137	-0.001477
C	-3.902341	1.596159	-0.000711
F	0.120492	1.594645	0.000544
F	-3.965223	3.954012	-0.002425
F	-5.226185	1.551786	-0.001279
F	-1.329649	-0.697964	0.001815
N	-3.262163	0.454136	-0.000009
H	-1.331380	3.797970	-0.001499

C5F5N.log

SCF (wB97x) = -744.409035121

E(SCF)+ZPE(0 K)= -744.359947

H(298 K)= -744.350715

G(298 K)= -744.393599

Lowest Frequency = 122.4309cm-1

C	-1.949886	0.474689	0.000944
C	-1.191657	1.638775	0.000148
C	-1.879768	2.848058	-0.000974
C	-3.271214	2.839395	-0.001514
C	-3.900566	1.600849	-0.001075
F	-1.222692	3.986298	-0.001608

F 0.130144 1.613781 0.000362
F -3.953382 3.971859 -0.002515
F -5.221388 1.556503 -0.001283
F -1.328016 -0.691407 0.001148
N -3.259096 0.459493 0.000178

Et3SiF.log

SCF (wB97x) = -627.066447038
E(SCF)+ZPE(0 K)= -626.864491
H(298 K)= -626.851267
G(298 K)= -626.903122
Lowest Frequency = 46.6982cm-1

C -2.752599 -1.880314 0.130424
H -3.059254 -2.354045 1.071677
H -3.227963 -2.467047 -0.667713
C -5.346591 -0.169214 0.201930
H -5.691061 0.869138 0.288965
H -5.741888 -0.535477 -0.755582
C -2.870113 0.828470 -1.389900
H -1.776206 0.892672 -1.329572
H -3.085188 0.245612 -2.296076
C -1.224120 -1.938128 -0.013410
H -0.858669 -2.967739 0.043798
H -0.897151 -1.525761 -0.973464
H -0.728353 -1.367051 0.777816
C -5.909943 -1.008417 1.358927
H -7.002495 -0.960123 1.389114
H -5.629647 -2.062284 1.261707
H -5.536749 -0.654762 2.325171
C -3.472986 2.236006 -1.514413
H -3.064928 2.767673 -2.379134
H -4.560311 2.195691 -1.635835
H -3.261528 2.838889 -0.625713
Si -3.480180 -0.157962 0.077681
F -2.925877 0.626088 1.430947

Et3SiH.log

SCF (wB97x) = -527.745695610
E(SCF)+ZPE(0 K)= -527.538210
H(298 K)= -527.525931
G(298 K)= -527.575415
Lowest Frequency = 48.0012cm-1

C -2.752485 -1.890719 0.084375
H -3.098510 -2.414644 0.984539
H -3.181923 -2.437469 -0.765972
C -5.356862 -0.208450 0.174718
H -5.736137 0.821299 0.192570
H -5.728541 -0.649966 -0.759818
C -2.903887 0.816094 -1.422562
H -1.806577 0.836769 -1.428750
H -3.199521 0.255167 -2.319466
C -1.219315 -1.936376 0.014480
H -0.845529 -2.964849 0.041649
H -0.847372 -1.476752 -0.907211
H -0.766594 -1.399225 0.855152
C -5.911008 -0.986621 1.376760
H -7.005263 -0.968167 1.398507
H -5.601377 -2.036635 1.350181
H -5.555741 -0.563376 2.322731
C -3.450665 2.248840 -1.498695
H -3.070683 2.778644 -2.377975
H -4.544033 2.256523 -1.558571
H -3.166068 2.830768 -0.615200
Si -3.474273 -0.149264 0.093042
H -2.974771 0.558330 1.314244

GaF2-NaBArF24.log

SCF (wB97x) = -4853.11762452
E(SCF)+ZPE(0 K)= -4852.231883
H(298 K)= -4852.147160
G(298 K)= -4852.367982
Lowest Frequency = 5.7187cm-1

Na 5.464359 2.700463 6.844364
F 4.686501 5.685164 7.639303
F 6.331861 6.832148 8.426449
F 5.209254 7.582497 6.745386
F 6.971934 4.625222 1.988573
F 7.073587 6.745615 2.375555
F 8.882566 5.574671 2.277211
C 8.225963 3.882197 6.068598
C 7.414240 4.692201 6.873839
H 7.370298 4.514014 7.946354

C	6.651753	5.739196	6.352759
C	6.694707	6.050107	5.000507
H	6.107160	6.862973	4.594773
C	7.525091	5.285166	4.188720
C	8.263334	4.224607	4.707912
H	8.893435	3.652083	4.033997
C	5.728283	6.475641	7.283186
C	7.612204	5.563708	2.710891
B	9.146170	2.661479	6.690047
F	13.241999	0.387379	3.568591
F	13.079668	-0.613228	5.469136
F	12.541253	-1.649963	3.654211
F	6.890257	0.503866	2.365725
F	8.053663	-1.250082	1.893760
F	6.826312	-1.214578	3.667017
C	9.508978	1.572249	5.518809
C	10.788483	1.026686	5.362869
H	11.598622	1.372101	5.997302
C	11.062621	0.038810	4.417639
C	10.065281	-0.453504	3.584776
H	10.274157	-1.229953	2.859064
C	8.789505	0.081733	3.715533
C	8.521977	1.080092	4.650159
H	7.508051	1.473167	4.700629
C	12.474170	-0.464090	4.279386
C	7.648230	-0.465786	2.904811
F	12.021336	7.375801	7.425952
F	12.602680	6.495756	5.548416
F	14.016362	6.595736	7.176070
F	12.465140	1.996643	10.847449
F	14.326154	2.900667	10.233794
F	13.624917	1.103155	9.268148
C	10.517934	3.302154	7.310381
C	11.044379	4.523850	6.875919
H	10.505912	5.104664	6.132274
C	12.250799	5.023090	7.366086
C	12.982807	4.322526	8.317899
H	13.917835	4.711635	8.701384
C	12.477321	3.105751	8.760393
C	11.272393	2.609064	8.266543
H	10.911506	1.652135	8.636302
C	12.728974	6.364480	6.880416
C	13.226729	2.286188	9.775253
F	6.392778	-2.051137	8.272431
F	5.273634	-0.746066	6.972721
F	4.662101	-1.010886	9.029582
F	8.506016	2.852876	11.991295
F	6.603186	1.920880	12.401085
F	6.679409	3.829977	11.398485
C	8.274708	1.920672	7.880632
C	7.474508	0.793756	7.648370
H	7.506896	0.302368	6.681646
C	6.598304	0.286408	8.609703
C	6.518418	0.853337	9.875915
H	5.838357	0.463051	10.623279
C	7.341605	1.941844	10.147297
C	8.193559	2.462251	9.174273
H	8.811907	3.318687	9.431116
C	5.728419	-0.882716	8.227339
C	7.283256	2.626443	11.488129
F	3.295640	2.034998	6.861228
F	4.868723	1.717654	4.762284
N	2.120486	-0.027838	4.747701
C	0.951749	0.013934	4.093041
C	0.377312	1.182275	3.574408
H	-0.577092	1.052281	3.083012
C	0.895456	2.486619	3.551663
N	2.049467	2.838136	4.126597
C	0.214021	-1.282393	3.890152
H	0.820792	-1.977877	3.303475
H	-0.730424	-1.116413	3.374340
H	0.017050	-1.765873	4.850826
C	0.113435	3.544056	2.820164
H	-0.094557	4.393112	3.476503
H	-0.827290	3.144337	2.444772
H	0.695422	3.928525	1.977379
C	2.680605	-1.284295	5.169423
C	3.530338	-1.966118	4.290050
C	4.098948	-3.161834	4.725268
H	4.763367	-3.701212	4.054986
C	3.840156	-3.676533	5.996147
C	2.984051	-2.969622	6.840836
H	2.777101	-3.356699	7.835439
C	2.401762	-1.764037	6.453823
C	3.814500	-1.415822	2.916681
H	4.303253	-0.438156	2.976975
H	2.896787	-1.286135	2.333183
H	4.477630	-2.081821	2.362350

C 4.502003 -4.947706 6.462250
H 4.728614 -5.610630 5.623291
H 3.869087 -5.490168 7.169532
H 5.445860 -4.722208 6.970082
C 1.524896 -0.981580 7.395761
H 1.970531 -0.007008 7.621933
H 1.391801 -1.519119 8.336545
H 0.533879 -0.791560 6.970346
C 2.558460 4.175588 3.989853
C 2.238354 5.135120 4.959675
C 2.734431 6.427482 4.790718
H 2.492146 7.183820 5.532777
C 3.523232 6.774237 3.693180
C 3.848639 5.783054 2.766768
H 4.477837 6.032678 1.916793
C 3.386138 4.474116 2.899316
C 1.368444 4.783093 6.138604
H 0.365595 4.478784 5.819233
H 1.262539 5.638632 6.808423
H 1.790249 3.950047 6.709074
C 3.992302 8.194341 3.503340
H 4.882752 8.240480 2.871256
H 4.220648 8.669538 4.461131
H 3.214522 8.794150 3.018719
C 3.765637 3.409383 1.903225
H 4.365206 3.832403 1.095747
H 2.885794 2.932003 1.459515
H 4.356567 2.622186 2.383558
Ga 3.082163 1.566136 5.093851

GaHF-NaBArF24.log

SCF (wB97x) = -4753.80845466
E(SCF)+ZPE(0 K)= -4752.917694
H(298 K)= -4752.834108
G(298 K)= -4753.052761
Lowest Frequency = 4.9835cm-1

Na 5.416818 2.491392 7.482912
F 4.528502 5.803897 7.697127
F 6.306120 6.797646 8.396874
F 5.246636 7.573758 6.686639
F 6.425807 4.081993 2.136830
F 6.777622 6.202759 2.322535
F 8.440824 4.833586 2.224347
C 8.000094 3.656493 6.205316
C 7.254987 4.567121 6.966205
H 7.288133 4.519775 8.051910
C 6.474979 5.567928 6.384543
C 6.425174 5.722483 5.005701
H 5.812289 6.488821 4.549078
C 7.197303 4.863458 4.231908
C 7.955715 3.851928 4.816155
H 8.543169 3.207298 4.170025
C 5.646625 6.446444 7.281656
C 7.207734 5.001245 2.731123
B 8.969861 2.512330 6.895634
F 13.037941 0.350567 3.944675
F 12.842760 -0.991658 5.617114
F 12.366493 -1.674886 3.626364
F 7.126562 -0.235502 2.256762
F 7.558800 -2.173844 3.092653
F 6.225367 -0.824854 4.123819
C 9.313312 1.347387 5.800346
C 10.601178 0.861880 5.562463
H 11.443283 1.293105 6.096046
C 10.845078 -0.172256 4.655258
C 9.808835 -0.766182 3.949148
H 9.995881 -1.573237 3.251062
C 8.517534 -0.295946 4.172121
C 8.280841 0.741395 5.067060
H 7.257711 1.086728 5.201479
C 12.265952 -0.627074 4.456837
C 7.360677 -0.885240 3.415739
F 11.625455 7.344031 7.118534
F 12.343020 6.336006 5.356253
F 13.677413 6.686824 7.015711
F 12.497635 2.410470 11.013974
F 14.334136 3.089196 10.107104
F 13.447103 1.220968 9.490536
C 10.347004 3.236404 7.407501
C 10.811501 4.444858 6.877533
H 10.225665 4.957923 6.119797
C 12.013512 5.018577 7.292721
C 12.804150 4.407608 8.258473
H 13.734850 4.855205 8.584345
C 12.362950 3.201535 8.791135
C 11.161551 2.632033 8.374901

H	10.850899	1.688608	8.817620
C	12.421987	6.339255	6.698964
C	13.164961	2.488543	9.845409
F	6.769395	-2.133630	10.251209
F	6.603944	-1.988553	8.105548
F	5.007861	-1.265430	9.365753
F	8.601263	3.233936	12.191354
F	6.791163	2.223748	12.791210
F	6.686487	3.989167	11.554272
C	8.213067	1.831789	8.198505
C	7.634433	0.550090	8.189981
H	7.701243	-0.063117	7.298707
C	6.983405	0.022770	9.303632
C	6.878201	0.750630	10.486613
H	6.373356	0.340158	11.353700
C	7.457010	2.011496	10.525012
C	8.114514	2.532627	9.409607
H	8.591473	3.505285	9.501120
C	6.351122	-1.344404	9.247946
C	7.385001	2.855272	11.771149
F	4.159557	2.039612	5.823166
H	4.905192	1.586692	3.197862
N	2.403993	0.125577	4.200422
C	1.108766	0.191732	3.893283
C	0.454591	1.363462	3.484439
H	-0.593323	1.256167	3.238917
C	0.970628	2.664433	3.400525
N	2.241245	2.980743	3.650314
C	0.283002	-1.067271	3.986773
H	0.704740	-1.851772	3.352514
H	-0.746460	-0.882049	3.682911
H	0.284263	-1.452816	5.010135
C	0.018378	3.765249	3.003340
H	-0.089617	4.495440	3.810061
H	-0.964679	3.361719	2.763994
H	0.401117	4.307358	2.134187
C	2.964246	-1.112434	4.663508
C	3.532456	-1.997527	3.739360
C	4.093950	-3.181796	4.215624
H	4.538873	-3.874086	3.504914
C	4.120575	-3.486721	5.575530
C	3.547260	-2.581577	6.470017
H	3.554813	-2.806766	7.533491
C	2.964634	-1.390979	6.038026
C	3.558920	-1.662593	2.271146
H	4.134208	-0.748367	2.089812
H	2.553832	-1.493948	1.870783
H	4.021598	-2.468332	1.697568
C	4.784055	-4.744561	6.075341
H	4.800084	-5.520302	5.305480
H	4.270966	-5.143016	6.954902
H	5.821323	-4.543074	6.364085
C	2.329307	-0.437468	7.017607
H	2.732738	0.572353	6.897405
H	2.500141	-0.768931	8.044058
H	1.246330	-0.368794	6.864252
C	2.644390	4.356703	3.645928
C	2.489928	5.114064	4.814713
C	2.874752	6.455010	4.789355
H	2.748090	7.053883	5.687280
C	3.416871	7.041862	3.646577
C	3.572790	6.253002	2.505320
H	3.996465	6.694541	1.606917
C	3.195600	4.911517	2.482022
C	1.891521	4.501448	6.054679
H	0.827500	4.277097	5.918272
H	1.982317	5.181814	6.903581
H	2.387842	3.557835	6.299495
C	3.854327	8.484889	3.644298
H	4.947558	8.564600	3.649347
H	3.479564	9.015930	4.522340
H	3.499007	9.004181	2.749647
C	3.340369	4.084561	1.231454
H	3.803768	4.667445	0.433239
H	2.368173	3.728778	0.873586
H	3.961535	3.201184	1.407074
Ga	3.615782	1.653321	4.058847

NaBArF24.log

SCF (wB97x) = -3647.91998979

E(SCF)+ZPE(0 K)= -3647.511081

H(298 K)= -3647.458890

G(298 K)= -3647.611249

Lowest Frequency = 7.6896cm-1

Na	2.005846	1.402170	-1.494059
F	0.839003	4.019028	-1.660434

F	-0.991574	4.636346	-2.612712
F	-0.141403	5.840369	-1.039135
F	-1.864799	3.655463	4.020378
F	-2.626169	5.437376	3.073162
F	-3.912420	3.727266	3.353985
C	-2.124059	1.622882	0.184533
C	-1.439663	2.453324	-0.705875
H	-1.120760	2.054107	-1.665394
C	-1.151038	3.786838	-0.409464
C	-1.547570	4.356088	0.792729
H	-1.326590	5.390851	1.023876
C	-2.241514	3.550013	1.690773
C	-2.513454	2.216686	1.396370
H	-3.044632	1.617743	2.131853
C	-0.379321	4.584165	-1.420566
C	-2.664360	4.097706	3.028188
B	-2.508761	0.060578	-0.148106
F	-3.675761	-4.252955	4.287405
F	-5.090020	-3.484501	2.849672
F	-3.576105	-4.884813	2.228129
F	1.416069	-0.983013	3.228709
F	0.174201	0.103487	4.612152
F	0.551182	-1.997081	4.927175
C	-2.281629	-0.844603	1.203167
C	-3.123241	-1.897776	1.580057
H	-4.007867	-2.114440	0.988935
C	-2.869563	-2.679911	2.706575
C	-1.755544	-2.450678	3.506112
H	-1.558642	-3.059483	4.379946
C	-0.907808	-1.408465	3.150861
C	-1.172449	-0.623146	2.030693
H	-0.493038	0.193558	1.798865
C	-3.801032	-3.819055	3.023867
C	0.300630	-1.081744	3.983227
F	-7.245417	2.904989	-1.367552
F	-7.100250	2.491827	0.741483
F	-8.607895	1.508597	-0.449828
F	-5.631101	-2.639784	-3.660007
F	-7.571238	-2.617198	-2.715884
F	-5.929650	-3.671818	-1.793764
C	-4.071119	-0.036973	-0.638303
C	-5.030968	0.942905	-0.359965
H	-4.740203	1.844439	0.170227
C	-6.364339	0.800843	-0.742180
C	-6.797967	-0.326974	-1.430017
H	-7.833274	-0.436359	-1.728942
C	-5.859761	-1.310278	-1.719449
C	-4.528045	-1.164304	-1.335125
H	-3.824770	-1.954878	-1.586645
C	-7.329878	1.917446	-0.450873
C	-6.255567	-2.553171	-2.468580
F	1.334264	-3.586782	-1.094314
F	2.545918	-1.806011	-1.138086
F	2.279297	-2.972976	-2.933870
F	-2.588020	-0.197320	-5.416591
F	-0.614138	-0.776029	-6.069178
F	-0.962166	1.213273	-5.311496
C	-1.534864	-0.490045	-1.353897
C	-0.399152	-1.276039	-1.135287
H	-0.159533	-1.599190	-0.126004
C	0.431904	-1.687973	-2.181141
C	0.165060	-1.326700	-3.495898
H	0.799405	-1.657517	-4.309134
C	-0.961180	-0.543513	-3.735038
C	-1.790503	-0.142211	-2.691089
H	-2.669949	0.453293	-2.922975
C	1.638474	-2.523072	-1.848581
C	-1.283158	-0.087923	-5.133606

TS1-2.log

SCF (wB97x) = -1732.87046390

E(SCF)+ZPE(0 K)= -1732.184471

H(298 K)= -1732.141827

G(298 K)= -1732.258129

Lowest Frequency = -117.0370cm-1

Al	4.212864	0.297153	0.259231
F	4.324954	1.582136	-1.149911
F	2.539277	-0.070229	-0.004684
N	4.957695	-1.129411	1.324833
C	5.626686	-0.979037	2.461321
C	5.782973	0.265644	3.090527
H	6.369827	0.279244	3.999255
C	5.148017	1.459627	2.735423
N	4.415718	1.611297	1.625751
C	6.218058	-2.190743	3.133885
H	5.431727	-2.917150	3.359748

H	6.722530	-1.918399	4.060546
H	6.934140	-2.689271	2.475673
C	5.273711	2.621279	3.688633
H	5.639906	3.512005	3.171318
H	5.952117	2.380833	4.506748
H	4.295992	2.876549	4.108260
C	4.763085	-2.439569	0.768242
C	3.559788	-3.110627	1.044504
C	3.311002	-4.322192	0.404480
H	2.380053	-4.844272	0.613468
C	4.216210	-4.875630	-0.502637
C	5.413073	-4.202864	-0.730351
H	6.142084	-4.627908	-1.416953
C	5.711629	-2.991430	-0.102883
C	2.559987	-2.533722	2.012573
H	2.117347	-1.621976	1.601576
H	3.027682	-2.269456	2.966697
H	1.759329	-3.249411	2.212417
C	3.892628	-6.155623	-1.230083
H	3.288344	-5.950956	-2.120816
H	3.320428	-6.839708	-0.597216
H	4.800205	-6.669307	-1.557865
C	7.051202	-2.345363	-0.340784
H	7.025711	-1.271476	-0.152343
H	7.381655	-2.507868	-1.370102
H	7.813825	-2.780483	0.315831
C	3.594961	2.791391	1.511794
C	4.062399	3.943685	0.870940
C	3.204703	5.041829	0.769144
H	3.566695	5.940933	0.275067
C	1.910713	5.019013	1.280929
C	1.479918	3.861134	1.930860
H	0.478110	3.828478	2.353181
C	2.300260	2.742994	2.057834
C	5.445627	4.027072	0.284226
H	5.420853	3.727268	-0.767393
H	6.147720	3.362207	0.791858
H	5.829817	5.048854	0.341005
C	0.990370	6.203214	1.126815
H	1.551970	7.125466	0.956380
H	0.366788	6.340610	2.014830
H	0.317550	6.063017	0.273662
C	1.810035	1.512067	2.777000
H	0.816581	1.681887	3.197957
H	2.480090	1.224971	3.594655
H	1.753402	0.662617	2.090161
Si	5.121465	0.509157	-2.527042
C	6.998476	0.658730	-2.393039
C	4.532869	1.851019	-3.730258
C	4.576763	-1.119500	-3.341920
C	7.526017	1.446131	-1.188218
C	3.270996	-1.745114	-2.833627
C	3.018461	1.907701	-3.968126
H	5.074655	-0.402109	-1.075254
H	7.400812	-0.360362	-2.368655
H	7.370907	1.115899	-3.318872
H	4.896808	2.828837	-3.390002
H	5.046062	1.643719	-4.681533
H	5.394796	-1.833251	-3.169203
H	4.517152	-0.955683	-4.426772
H	8.614967	1.373328	-1.107177
H	7.267951	2.505304	-1.260888
H	7.105966	1.076471	-0.243159
H	3.042189	-2.671329	-3.371226
H	2.418487	-1.068894	-2.954165
H	3.344347	-1.992257	-1.770228
H	2.749196	2.682748	-4.693675
H	2.642068	0.954001	-4.355080
H	2.482282	2.121297	-3.038639

TS1.log

SCF (wB97x) = -5380.85358977

E(SCF)+ZPE(0 K)= -5379.757751

H(298 K)= -5379.662340

G(298 K)= -5379.904579

Lowest Frequency = -141.8722cm-1

Na	0.723629	1.109224	-0.947479
F	0.139856	4.095444	-2.456285
F	-1.868348	4.492482	-3.128072
F	-0.881914	5.881544	-1.803846
F	-1.656126	3.854114	3.653961
F	-2.458280	5.625255	2.718693
F	-3.760431	3.974938	3.207968
C	-2.214579	1.625752	0.035142
C	-1.787247	2.436865	-1.023145
H	-1.608519	2.000228	-2.004298

C	-1.587149	3.814103	-0.879673
C	-1.838926	4.452343	0.325597
H	-1.684591	5.517987	0.439639
C	-2.285883	3.670739	1.387609
C	-2.457487	2.298378	1.247230
H	-2.794342	1.728624	2.109125
C	-1.058603	4.581964	-2.061623
C	-2.543087	4.288732	2.737560
B	-2.514416	0.013535	-0.098728
F	-4.019327	-4.275240	2.515432
F	-2.081407	-5.104395	2.069580
F	-2.601958	-4.623518	4.105811
F	1.085301	0.478140	3.670763
F	-0.253048	0.023506	5.294082
F	1.207945	-1.430495	4.671471
C	-2.053923	-0.797119	1.258644
C	-2.587088	-2.059678	1.548447
H	-3.356086	-2.476689	0.905702
C	-2.161249	-2.813581	2.640076
C	-1.192923	-2.326896	3.509150
H	-0.869002	-2.905363	4.366752
C	-0.649343	-1.076207	3.241383
C	-1.060029	-0.338328	2.132781
H	-0.585605	0.619121	1.951462
C	-2.719264	-4.196312	2.838482
C	0.350642	-0.503444	4.206958
F	-6.993942	2.080723	1.699063
F	-8.634860	0.948477	0.871544
F	-7.661500	2.463846	-0.313031
F	-5.895904	-2.900512	-3.061979
F	-7.774779	-2.873501	-2.000181
F	-6.077809	-3.918383	-1.172967
C	-4.137230	-0.195742	-0.317187
C	-5.096625	0.689129	0.192492
H	-4.781089	1.588520	0.710814
C	-6.463990	0.458008	0.057910
C	-6.941094	-0.668927	-0.603641
H	-8.004098	-0.846758	-0.712337
C	-6.007616	-1.556551	-1.121164
C	-4.640244	-1.322246	-0.981626
H	-3.949827	-2.043368	-1.411347
C	-7.436287	1.479402	0.582927
C	-6.446258	-2.804800	-1.836862
F	0.573080	-4.212419	-1.293835

F	2.028456	-2.652547	-1.617766
F	1.340157	-3.844299	-3.275972
F	-3.451924	-0.662455	-5.256149
F	-1.648346	-1.451920	-6.140460
F	-1.731300	0.609319	-5.508378
C	-1.742110	-0.657122	-1.389262
C	-0.713284	-1.599571	-1.264187
H	-0.330531	-1.846910	-0.278890
C	-0.168523	-2.250618	-2.374071
C	-0.618370	-1.979498	-3.658090
H	-0.196386	-2.486431	-4.516811
C	-1.617285	-1.022879	-3.808656
C	-2.164613	-0.381238	-2.701519
H	-2.977874	0.323284	-2.859605
C	0.939982	-3.242256	-2.145807
C	-2.111754	-0.642838	-5.178572
F	2.970634	1.083130	-1.144513
N	3.948298	-1.529468	1.768929
C	4.224890	-1.314655	3.049357
C	4.231355	-0.040163	3.645685
H	4.416514	-0.026595	4.710983
C	4.047060	1.209561	3.050631
N	3.788817	1.405974	1.748380
C	4.565250	-2.495502	3.921262
H	3.758887	-3.233059	3.898809
H	4.732238	-2.185461	4.951920
H	5.466003	-2.993938	3.552323
C	4.133005	2.421240	3.943256
H	4.910901	3.109636	3.603468
H	4.343332	2.132373	4.972002
H	3.186455	2.969175	3.918006
C	4.068517	-2.842724	1.199471
C	2.951902	-3.691807	1.155214
C	3.095112	-4.933761	0.540692
H	2.236382	-5.599036	0.504475
C	4.294633	-5.335677	-0.047850
C	5.386228	-4.472829	0.021654
H	6.336230	-4.774444	-0.413490
C	5.298948	-3.229588	0.650175
C	1.637473	-3.282813	1.763239
H	1.309319	-2.314379	1.376633
H	1.715116	-3.179000	2.850545
H	0.864266	-4.024713	1.553222
C	4.391822	-6.662003	-0.757417

H 5.431899 -6.972518 -0.884883
H 3.937555 -6.601506 -1.752408
H 3.864416 -7.445630 -0.206199
C 6.521512 -2.361728 0.788410
H 6.275472 -1.300010 0.729109
H 7.255774 -2.588520 0.012309
H 7.004902 -2.521629 1.759475
C 3.759318 2.740486 1.219418
C 4.964097 3.323948 0.800812
C 4.926739 4.593359 0.229523
H 5.856787 5.046505 -0.105551
C 3.727235 5.285188 0.053368
C 2.549243 4.684551 0.491385
H 1.604664 5.211071 0.371875
C 2.543690 3.418580 1.079446
C 6.268600 2.590035 0.972816
H 6.212014 1.575909 0.564957
H 6.538520 2.485487 2.029368
H 7.082769 3.114967 0.468590
C 3.703667 6.621427 -0.641410
H 3.652196 6.483643 -1.727495
H 4.605605 7.200237 -0.425617
H 2.834678 7.212968 -0.342244
C 1.252829 2.827670 1.580615
H 0.410423 3.154725 0.964948
H 1.036194 3.153944 2.603251
H 1.287155 1.737393 1.608197
Si 4.499283 0.340548 -2.258462
C 6.061524 -0.734155 -2.487742
C 5.115528 2.095441 -2.512289
C 3.343112 -0.331569 -3.574520
C 7.336837 -0.191578 -1.833637
C 1.940504 0.250421 -3.745304
C 4.087638 3.191126 -2.804169
H 4.499257 -0.429853 -0.710945
H 5.849498 -1.748738 -2.126413
H 6.223271 -0.824037 -3.571945
H 5.727145 2.382643 -1.649806
H 5.824867 2.009699 -3.348693
H 3.292929 -1.421320 -3.470551
H 3.923417 -0.161106 -4.493734
H 8.166762 -0.899999 -1.921682
H 7.653802 0.747448 -2.297595
H 7.198047 0.006058 -0.765629
H 1.497272 -0.058152 -4.696774
H 1.939597 1.343161 -3.717521
H 1.275888 -0.126729 -2.965120
H 4.580823 4.158809 -2.942818
H 3.521885 2.977493 -3.716974
H 3.382236 3.292788 -1.977387
Ga 3.414520 -0.075076 0.565834
F 1.590867 -0.375350 0.442087

TS1'.log

SCF (wB97x) = -1732.87499728
E(SCF)+ZPE(0 K)= -1732.192405
H(298 K)= -1732.148552
G(298 K)= -1732.267861
Lowest Frequency = -113.3516cm-1

F 2.557763 0.834027 -1.112872
F 1.521387 -0.746274 0.873651
N 4.258469 -0.925792 2.007535
C 4.387964 -0.460342 3.242478
C 3.994021 0.835536 3.623125
H 4.121640 1.081243 4.669149
C 3.639216 1.900221 2.782849
N 3.363784 1.774352 1.481198
C 5.035663 -1.335855 4.286419
H 4.620915 -2.346353 4.251142
H 4.891632 -0.922930 5.284858
H 6.110575 -1.421575 4.101900
C 3.614289 3.282558 3.385658
H 4.409578 3.893497 2.947001
H 3.762780 3.240588 4.464203
H 2.669659 3.788953 3.172216
C 4.972420 -2.085995 1.570767
C 4.308918 -3.312467 1.425319
C 5.012300 -4.383060 0.871780
H 4.505153 -5.338549 0.758658
C 6.339500 -4.262586 0.460506
C 6.976936 -3.035392 0.638919
H 8.014388 -2.922694 0.332374
C 6.315548 -1.939207 1.189577
C 2.879384 -3.476640 1.869896
H 2.205775 -2.822848 1.308586
H 2.761189 -3.208339 2.925264

H	2.552609	-4.511265	1.743745
C	7.060302	-5.419908	-0.183105
H	6.975553	-5.370347	-1.274334
H	6.641576	-6.377826	0.136382
H	8.125625	-5.412086	0.063293
C	7.033296	-0.627779	1.377292
H	6.404324	0.216666	1.083216
H	7.952640	-0.598474	0.788472
H	7.306508	-0.464341	2.425978
C	3.171516	2.937983	0.664378
C	4.275224	3.488735	0.002974
C	4.069698	4.577499	-0.842604
H	4.922326	5.010316	-1.361056
C	2.798279	5.110425	-1.052340
C	1.718484	4.529520	-0.387443
H	0.718589	4.926159	-0.547987
C	1.880133	3.438338	0.465634
C	5.645340	2.885060	0.166389
H	5.679722	1.879896	-0.272323
H	5.930861	2.782748	1.218185
H	6.402970	3.489837	-0.336607
C	2.590725	6.249920	-2.017082
H	2.447096	5.869369	-3.034713
H	3.454757	6.919789	-2.036115
H	1.706226	6.837689	-1.757678
C	0.695655	2.781035	1.122710
H	-0.224364	3.326123	0.900776
H	0.806413	2.728828	2.210659
H	0.580249	1.752158	0.765474
Si	3.672280	-0.274039	-2.250331
C	5.175386	-1.420099	-2.559137
C	4.153499	1.286806	-3.191747
C	2.222431	-1.335407	-2.801809
C	6.532475	-0.790820	-2.233467
C	0.810687	-0.743400	-2.760263
C	3.044560	2.282382	-3.545639
H	4.148642	-0.586092	-0.627035
H	5.040748	-2.334677	-1.965972
H	5.151773	-1.722844	-3.616895
H	4.918762	1.798475	-2.590355
H	4.677022	0.951238	-4.098137
H	2.260011	-2.251502	-2.195456
H	2.473503	-1.659416	-3.822196
H	7.347049	-1.520903	-2.284498
H	6.771522	0.024479	-2.924593
H	6.540927	-0.375127	-1.220004
H	0.075880	-1.454575	-3.152233
H	0.741683	0.171323	-3.356696
H	0.531011	-0.488343	-1.735990
H	3.448360	3.142731	-4.090824
H	2.280272	1.820001	-4.179537
H	2.555201	2.652303	-2.642419
Ga	3.164383	0.030539	0.645112

TS2.log

SCF (wB97x) = -1750.91818332

E(SCF)+ZPE(0 K)= -1750.386204

H(298 K)= -1750.346268

G(298 K)= -1750.458873

Lowest Frequency = -712.8047cm-1

F	0.414750	-2.520136	-1.171323
N	-0.484038	1.929348	0.284136
C	-0.020841	2.822844	1.154850
C	1.330984	2.926761	1.515184
H	1.575754	3.719276	2.209722
C	2.386702	2.065347	1.172609
N	2.283371	1.065932	0.297639
C	-1.003550	3.766852	1.800109
H	-1.630920	4.239822	1.039638
H	-0.487177	4.539037	2.369563
H	-1.672778	3.226510	2.475577
C	3.716133	2.285784	1.849786
H	3.984614	1.412561	2.451560
H	3.684874	3.160758	2.497824
H	4.510786	2.417976	1.110631
C	-1.885689	1.662348	0.162679
C	-2.595975	2.160825	-0.934740
C	-3.937855	1.798889	-1.073040
H	-4.497554	2.177201	-1.924893
C	-4.572381	0.965637	-0.154628
C	-3.834112	0.489602	0.931173
H	-4.313090	-0.172427	1.648340
C	-2.491038	0.810554	1.102485
C	-1.925503	3.063144	-1.938349
H	-1.136952	2.535324	-2.485746
H	-1.456175	3.924150	-1.451802

H	-2.647150	3.435006	-2.668486
C	-6.010338	0.552649	-0.330282
H	-6.598000	0.775966	0.565533
H	-6.077349	-0.525818	-0.505993
H	-6.472516	1.063373	-1.178426
C	-1.703849	0.249922	2.259250
H	-1.445543	1.022071	2.992117
H	-0.758614	-0.189740	1.923776
H	-2.274773	-0.521830	2.779688
C	3.392361	0.193565	0.040510
C	3.537146	-0.970745	0.807612
C	4.589896	-1.833515	0.507154
H	4.712535	-2.737905	1.098384
C	5.482933	-1.568859	-0.531537
C	5.307097	-0.403368	-1.276991
H	5.995658	-0.181217	-2.088829
C	4.267709	0.488664	-1.013588
C	2.577180	-1.274040	1.928121
H	1.549755	-1.347223	1.559264
H	2.586523	-0.492026	2.694771
H	2.826575	-2.221902	2.409011
C	6.591276	-2.536764	-0.859437
H	6.237562	-3.310332	-1.549825
H	6.956987	-3.041609	0.038749
H	7.434539	-2.030266	-1.336186
C	4.093270	1.740578	-1.834547
H	4.874652	1.818307	-2.593213
H	4.134396	2.641579	-1.213535
H	3.124284	1.749179	-2.344332
H	-0.148487	-0.785345	-0.323117
H	0.779794	0.793329	-2.286328
C	-0.783240	-2.039177	-0.696956
C	-1.282975	-2.637015	0.491145
C	-1.795964	-1.779465	-1.662859
C	-2.638923	-2.789384	0.670081
C	-3.118599	-1.986910	-1.352478
N	-3.558619	-2.498984	-0.222137
F	-0.413633	-2.951312	1.460074
F	-3.070715	-3.296917	1.825502
F	-4.039408	-1.688682	-2.267126
F	-1.432655	-1.235037	-2.829910
Ga	0.660656	0.719388	-0.729326

10) References

[S1]; Barbon S. M.; Staroverov V. N.; Boyle P.D.; Gilroy J. B. *Dalton Trans.*, **2014**, *43*, 240–250.

[S2]: Yow. S.; Gates S. J.; White A. J. P.; Mark R. Crimmin M. R. *Angew. Chem. Int. Ed.*, **2012**, *51*, 12559 –12563.

[S3]: Singh S.; Ahn H.; Stasch A.; Jancik V.; Roesky H. W.; Pal A.; Biadene M.; Herbst-Irmer R.; Noltemeyer M.; Schmidt H. *Inorg. Chem.*, **2006**, *45*, 1853−1860.

[S4]: Zhi Y., Yafei Y., Mingdong Z., Sriman D., Totan M.,Debasis K., Xiaoli M., Dongxiang Z., Herbert W. R. *Chem. Eur. J*, **2019**, *7*, 1588–1593.

[S5]: Mulryan D.; White A. J. P.; Crimmin M. R. *Org. Lett.*, **2020**, *22*, 9351−9355.

[S6]: Rosemary C. H. Michael E. P., *Journal of Fluorine Chemistry*, **1986**, *31*, 129-133.

[S7]: Abdusalom A. S., Barbara M. K., Thibault D., Albert R., Euro S., Rosario S., Farzaneh F., Kay S. *Angew. Chem. Int. Ed.*, **2022**, e202213429.

[S8]: Nishino K.; Tsukahara S.; Ogiwara Y.; Sakai N. *Eur. J. Org. Chem.*, **2019**, *7*, 1588–1593.

[S9]: Christopher M. T., Matthew J. W., Jackie A. G., *J. Fluorine Chem.*, **2006**, *127*, 249-256.

[S10]: Pang Y.; Leutzsch M.; Nothling N.; Katzenburg F.; Cornella J. *J. Am. Chem. Soc.*, **2021**, *143*, 12487−12493.

[S11]: Kikushima K.; Grellier M.; Ohashi M.; Ogoshi S. *Angew. Chem. Int. Ed.*, **2017**, *56*, 16191 –16196.

[S12]: Lv H.; Zhan J.; Cai Y.; Yu Y.; Wang B.; Zhang J. *J. Am. Chem. Soc.*, **2012**, *134*, 16216−16227.

[S13]: Lv H.; Cai Y.; Zhang J. *Angew. Chem. Int. Ed.*, **2013**, *52*, 3203 –3207.

[S14]: D. J. Burton; T. D. Spawn; P. L. Heinze; A. R. Bailey; S. Shin-ya. *J. Fluorine Chem.*, **1989**, *44*, 167-174.

[S15]: Zhang J.; Wu J.; Xiong Y.; Cao S. *Chem. Commun.*, **2012**, *48*, 8553–8555.

[S16]: SHELXTL v5.1, Bruker AXS, Madison, WI, 1998.

[S17]: SHELX-2013, G.M. Sheldrick, *Acta Cryst.*, 2015, **C71**, 3-8.

[S18]: O.V. Dolomanov, L.J. Bourhis, R.J. Gildea, J.A.K. Howard, H. Puschmann, *J. Appl. Cryst.*, 2009, **42**, 339-341.

[S19] NBO 6.0. Glendening, E. D.; Badenhoop, J. K.; Reed, A. E.; Carpenter, J. E.; Bohmann, J. A.; Morales, C. M.; Landis, C. R.; Weinhold, F. Theoretical Chemistry Institute, University of Misconsin, Madison (2013).

[S20] AIMAll (Version 13.10.19), Keith, T. A. TK Gristmill Software, Overland Park, KS, USA (2013) (aim.tkgristmill.com).
